# Rotational Spectra of Unsaturated Carbon Chains Produced by Pyrolysis: The Case of Propadienone, Cyanovinylacetylene, and Allenylacetylene

**DOI:** 10.1021/acs.jpca.2c05018

**Published:** 2022-08-31

**Authors:** Alessio Melli, Mattia Melosso, Luca Bizzocchi, Silvia Alessandrini, Ningjing Jiang, Francesca Tonolo, Salvatore Boi, Giorgia Castellan, Carlotta Sapienza, Jean-Claude Guillemin, Luca Dore, Cristina Puzzarini

**Affiliations:** † Dipartimento di Chimica “Giacomo Ciamician”, Università di Bologna, Via F. Selmi 2, 40126 Bologna, Italy; ‡ Scuola Normale Superiore, Piazza dei Cavalieri 7, 56126 Pisa, Italy; ¶ Scuola Superiore Meridionale, Largo San Marcellino 10, 80138 Naples, Italy; § Univ Rennes, Ecole Nationale Supérieure de Chimie de Rennes, CNRS, ISCR-UMR6226, F-35000 Rennes, France

## Abstract

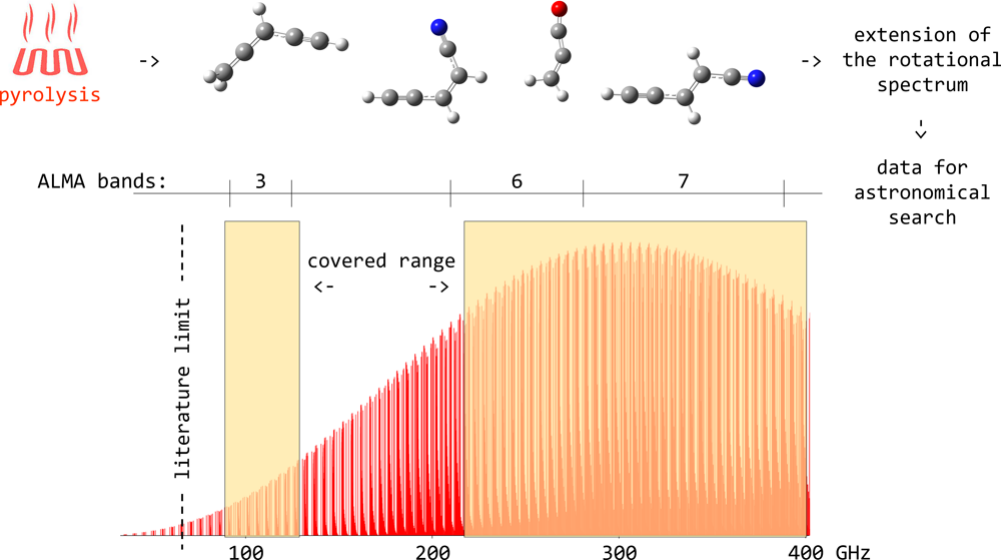

Several interstellar molecules are highly reactive unsaturated carbon chains, which are unstable under terrestrial conditions. Laboratory studies in support of their detection in space thus face the issue of how to produce these species and how to correctly model their rotational energy levels. In this work, we introduce a general approach for producing and investigating unsaturated carbon chains by means of selected test cases. We report a comprehensive theoretical/experimental spectroscopic characterization of three species, namely, propadienone, cyanovinylacetylene, and allenylacetylene, all of them being produced by means of flash vacuum pyrolysis of a suitable precursor. For each species, quantum-chemical calculations have been carried out with the aim of obtaining accurate predictions of the missing spectroscopic information required to guide spectral analysis and assignment. Rotational spectra of the title molecules have been investigated up to 400 GHz by using a frequency-modulation millimeter-/submillimeter-wave spectrometer, thus significantly extending spectral predictions over a wide range of frequency and quantum numbers. A comparison between our results and those available in the literature points out the clear need of the reported laboratory measurements at higher frequencies for setting up accurate line catalogs for astronomical searches.

## Introduction

Since the first detection of polyatomic molecules in space using radioastronomy, which dates back to the late 1960s,^1−3^ the molecular discovery has proceeded at a nearly constant pace.^4^ Very recently, this has tremendously accelerated with more than 40 different species identified in the interstellar medium (ISM) in 2021.^5^ Most of them belong to the family of the so-called complex organic molecules,^6^ namely, carbon-containing species with more than five atoms, thus confirming that the ISM features a substantial degree of chemical complexity. Its extreme conditions, which means very low temperature (10–100 K) and density (10^4^–10^8^ cm^–3^) together with the pervasive presence of ionizing radiation,^7^ put important constraints on the chemistry and allow the existence of exotic species which are unstable or highly reactive in the ordinary terrestrial conditions.

Most of the molecular detections in the ISM have been accomplished using radioastronomy, which implies the observation of molecular rotational transitions. In the past decade, the improved sensitivity of ground-based facilities such as ALMA^8^ and YEBES^9^ have revolutionized the field of astrochemistry, as addressed above. This has also greatly increased the number of lines to be assigned, thus necessitating a massive parallel laboratory effort. Indeed, while a few species have been identified in space on the basis of highly accurate quantum-chemical predictions of their rotational transitions (see, e.g., refs (10) and (11)), the detection of the vast majority of interstellar molecules has been guided by rotational spectra characterized in the laboratory. In addition, if spectral predictions are based on extrapolation from low-frequency measurements, ambiguities or discrepancies may arise in the millimeter- and submillimeter-wave regions.^12,13^ Figure 2 of ref (12) provides a clear example of disagreement in rest frequencies which may become troublesome when analyzing astronomical spectral surveys that contain hundreds of unknown features.

As suggested above, the chemical species of interest can be very short living on Earth and, in such cases, their spectroscopic characterization is a challenging task. Radical and ions are transient species which are typically generated in situ using plasma techniques.^14,15^ Neutral unsaturated species such as imines are stable molecules but tend to be highly reactive and their spectroscopic characterization as isolated system is problematic. For such compounds, flash vacuum pyrolysis has been proven to be a reliable methodology for production and has been already employed in the study of several interstellar members of the imine family.^16−19^

In this work, we demonstrate the validity of pyrolysis as the technique of choice for the production of isolated unsaturated carbon chains, spanning from pure hydrocarbons to oxygen or nitrogen-bearing molecules. In particular, we have considered three different species as test cases, namely, propadienone (1,2-propadien-1-one or methyleneketene), cyanovinylacetylene (2-penten-4-ynenitrile, both *E* and *Z* isomers), and allenylacetylene (3,4-pentadiene-1-yne). We report a comprehensive investigation of the ground-state rotational spectra of these three molecules, thus providing the astronomical community with a catalog of accurate rest frequencies up to 400 GHz. The study is complemented by high-level quantum-chemical computations of spectroscopic parameters and the energetics of the [H_2_C_3_O] and [H_3_C_5_N] families of isomers.

The astrochemical relevance of these molecules is undoubted. Allenylacetylene and the *E* isomer of cyanovinylacetylene have been recently identified in the cold core of the Taurus molecular cloud (TMC-1).^20,21^ Propadienone has so far escaped detection despite the fact that it is the lowest in energy form of the [H_2_C_3_O] family,^22^ with the less stable propynal^23^ and cyclopropenone isomers^24^ that have already been identified. Such apparent violation of the so-called minimum energy principle^25−27^ may be explained by a faster destruction chemistry, as pointed out by Shingledecker et al.^28^ However, the recent identification of its sulfur analog^29^ gives hope for a possible future discovery.

The manuscript is organized as follows. In the next two sections, Experimental Details and Computational Methods will be provided. In the Results and Discussion, the three test cases considered will be discussed starting from propadienone and moving to cyanovinylacetylene and then allenylacetylene. Finally, we draw our conclusions in the last section.

## Experimental Details

The rotational spectra of propadienone, cyanovinylacetylene, and allenylacetylene have been recorded using a frequency-modulation millimeter/submillimeter-wave (mm/sub-mm) spectrometer, described in detail elsewhere.^30,31^ Briefly, the mm/sub-mm radiation has been produced by a series of Gunn oscillators working in the 75–134 GHz range. The frequency and phase stability of these primary sources are ensured by a phase-lock loop; higher frequencies have been obtained by exploiting passive frequency multipliers (in detail, a tripler and a quadrupler). The detection system consists of Schottky barrier diodes, whose output signal is demodulated by a lock-in amplifier set at twice the modulation frequency, thus leading to second harmonic (2*f*) detection.

As briefly mentioned in the Introduction, the three title species have been produced in a flash vacuum pyrolysis apparatus. This system is constituted by a quartz tube surrounded by a 30 cm long tubular oven and is connected directly to one inlet of the glass-made absorption cell of the spectrometer (3.25 m long, 5 cm in diameter). The oven can reach temperatures as high as 1200 °C. Due to the unstable nature of the target molecules, the measurements have been performed in dynamical conditions, with a tenuous flow of fresh pyrolysis products continuously assured by the vacuum system.

Propadienone, cyanovinylacetylene, and allenylacetylene have been generated starting from three different precursors. For the first two molecules, the precursor compounds (acrylic anhydride and 2,3-pyridinedicarboxylic anhydride, respectively) were chosen on the basis of literature studies.^32,33^ Allenylacetylene was instead identified as side product of the pyrolysis of dipropargylamine during the spectroscopic investigation of propargylimine^19^ (see the Supporting Information for further details). For each species, the production conditions have been optimized by adjusting the temperature of the precursor (*T*_prec_, which alters its vapor pressure), the temperature of the pyrolysis apparatus (*T*_pyro_), and the pressure of the gaseous products inside the absorption cell (*P*_cell_). The best experimental conditions used are detailed in Table 1.

**Table 1:** Summary of the Experimental Conditions Employed in This Work^a^

	allenylacetylene	cyanovinylacetylene	propadienone
precursor	dipropargylamine	2,3-pyridinedicarboxylic anhydride	acrylic anhydride
physical state	solid	solid	liquid
*T*_prec_	25 °C	120 °C	0 °C
*T*_pyro_	800 °C	950–1000 °C	660 °C
*P*_cell_	9–12 mTorr	6–7 mTorr	7–10 mTorr

a *T*_prec_: temperature of the precursor. *T*_pyro_: temperature of the pyrolysis furnace. *P*_cell_: pressure in the absorption cell.

## Computational Methods

The rotational spectra of propadienone, cyanovinylacetylene, and allenylacetylene have been already experimentally investigated in the low-frequency regime, as addressed in the specific sections in the following. However, a complete characterization is not available, with crucial spectroscopic parameters still missing prior to this work. For these species, the centrifugal distortion characterization was limited to the quartic terms. It is instead crucial to understand how the rotational transitions shift and how the overall appearance of the spectrum modifies by including high-order terms. Accurate estimates are also important for guiding fitting procedures in order to avoid the derivation of unreliable parameters. For these reasons, to support the spectral analysis and guide the fitting procedures, state-of-the-art quantum-chemical computations have been carried out. Concerning cyanovinylacetylene and allenylacetylene, the so-called jun-ChS composite approach^34^ (the acronym ChS standing for “cheap” scheme) has been used to provide accurate equilibrium geometries and, straightforwardly, equilibrium rotational constants. The same level of theory has also been applied in the energetics characterization. While a detailed description of jun-ChS can be found in refs (34) and (35), we briefly recall that it is based on the frozen-core (fc) CCSD(T)/jun-cc-pVTZ^36,37^ level of theory (with CCSD(T) standing for coupled cluster singles, doubles, and a perturbative triple excitations^38^), which is improved by accounting for the extrapolation to the complete basis set (CBS) limit and core–valence (CV) correlation effects using the MP2 method^39^ (MP2 standing for Møller–Plesset perturbation theory to second order). Owing to its size, propadienone could be characterized using a composite scheme entirely based on CCSD(T) for both electronic energy evaluation and equilibrium geometry determination.^40,41^ In detail, the so-called CCSD(T)/CBS+CV approach has been employed, which accounts for the extrapolation to the CBS limit and CV correlation effects using CCSD(T).^42^

Focusing on the determination of spectroscopic parameters, the ground-state rotational constants have been determined by correcting their equilibrium values for vibrational contributions, the latter being obtained within vibrational perturbation theory to second order (VPT2).^43^ From a computational point of view, this requires anharmonic force-field computations, which have been performed using the double-hybrid B2PLYP-D3(BJ) density functional^44−46^ in conjunction with a triple-ζ basis set (jun-cc-pVTZ).^36,37,47^ As byproducts of the anharmonic calculations, the corresponding zero-point energy (ZPE) contribution as well as quartic and sextic centrifugal distortion constants have been derived. Furthermore, first-order properties, namely, electric dipole moment components and nuclear quadrupole coupling constants, have been determined at the same level of theory. For propadienone, first-order properties and harmonic force field (and thus harmonic ZPE and quartic centrifugal distortion constants) have been evaluated at the CCSD(T)/cc-pCVQZ level of theory, with all electrons being correlated.^47,48^

## Results and Discussion

**Figure 1:**
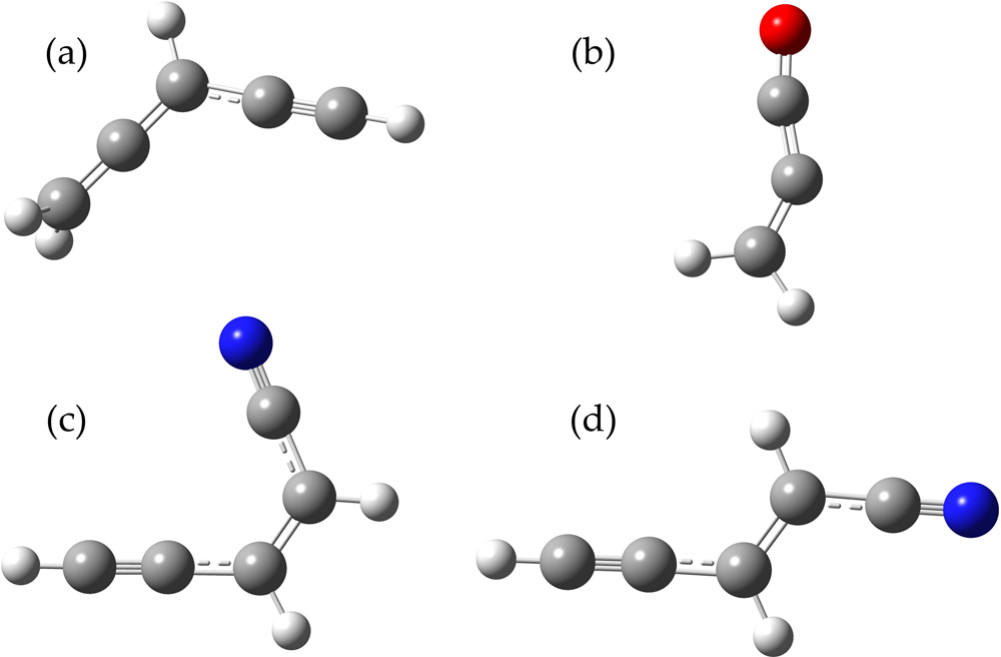
Molecular structure of the species considered in this work: (a) allenylacetylene; (b) propadienone; (c) (*Z*)-cyanovinylacetylene; (d) (*E*)-cyanovinylacetylene.

The three molecules considered in this work are depicted in Figure 1; for cyanovinylacetylene, both *Z* and *E* isomers were considered. In the following, the results for these species are presented and discussed in separated subsections. All spectral analyses have been performed using the SPFIT/SPCAT suite of programs^50^ and employing Watson’s *S*-reduced Hamiltonian^51^ in its *I*^*r*^ representation.^52^ In all cases, the analyzed line lists include data from earlier measurements, taken from the literature. Different statistical weights were thus assigned to the various subsets to take into account their measurement precision. This is detailed in each subsection.

### Propadienone

Propadienone (*l*-H_2_C_3_O, also known as 1,2-propadien-1-one or methyleneketene) is a near-prolate asymmetric rotor (κ = −0.998) which exhibits a tunneling motion between two equivalent planar-but-bent configurations, passing through a “linear” transition state. Its permutation-inversion symmetry group is *C*_2*v*_(*M*). Given its isomorphism with the *C*_2*v*_ point symmetry group, the spin statistics of *l*-H_2_C_3_O is analogous to that of formaldehyde (H_2_C=O) and ketene (H_2_C=C=O). The tunneling motion exchanges a pair of hydrogen nuclei along the *a* principal axis. The most evident consequence of this large amplitude motion is the splitting of each rotational energy level into two sublevels, one symmetric (0^+^) and one antisymmetric (0^–^) with the respect to the inversion. Since the overall molecular wave function must be antisymmetric (hydrogens being Fermions), in the 0^+^ state the symmetric rotational wave functions (even values of *K*_*a*_) only combine with antisymmetric spin functions (*I*_TOT_ = *I*_H_1__ + *I*_H_2__ = 0, *para* species), while antisymmetric rotational energy levels (odd values of *K*_*a*_) exist only with the symmetric spin functions (*I*_TOT_ = 1, *ortho* species). The opposite behavior applies to the 0^–^ state. As a result, each rotational transition appears as a doublet with a relative intensity of 3:1 between the *ortho* and *para* species. Moreover, the selection rules impose that *a*-type transitions (μ_*a*_ = 2.156(3) D) occur within each inversion state, while *b*-type transitions (μ_*b*_ = 0.7914(6) D) connect inversion states of different symmetry (see ref (32) for the dipole moment determination).

Together with cyclopropenone and propynal, propadienone is one of the three most stable isomers of the [H_2_C_3_O] family.^53^ Despite this, it is the only one of the three that has not been detected in the ISM,^22,54^ thus violating the minimum energy principle. Several astronomical searches have been carried out, spanning from starless cores to molecular clouds,^22,55^ but only upper limits on the molecular abundance of propadienone could be retrieved so far. These searches have been performed relying on the spectroscopic information collected in the Cologne Database for Molecular Spectroscopy (CDMS).^56,57^ Interestingly, while the rotational spectra of cyclopropenone and propynal have been studied up to 500 GHz,^58,59^ prior to the present work, the characterization of propadienone was limited to frequencies below 50 GHz.^32,49,60,61^ Spectral predictions extrapolated from such low-frequency data, though, do not meet the accuracy required for an effective identification of this molecule at higher frequencies, and this is particularly true for cold quiescent sources such as TMC-1, where molecular emissions exhibit line widths smaller than 1 km s^–1^.

The present work aims at extending the knowledge of the rotational spectrum of propadienone and providing reliable spectral predictions also in the 3–1 mm range, similarly to what has been done for the two other [H_2_C_3_O] isomers. Propadienone is a kinetically unstable compound which has never been isolated in the condensed phase, even when diluted in a solvent. Therefore, it has been produced directly in situ by exploiting the methodology described in the literature, i.e., through gas-phase pyrolysis of acrylic anhydride,^62,63^ and using the apparatus described in the Experimental Details. Given its relatively high vapor pressure, the precursor (acrylic anhydride) was kept in a water/ice bath at 0 °C to facilitate the flow regulation inside the pyrolysis system. The experimental conditions were adjusted by monitoring the signal-to-noise ratio (S/N) of the *J*_*K_a_*,*K_c_*_ = 10_1,10_–9_1,9_ transition pair, predicted around 85.8 GHz by the CDMS catalog. The best working conditions are those collected in Table 1 (oven temperature, 660 °C; pressure inside the absorption cell, 7–10 mTorr).

**Figure 2:**
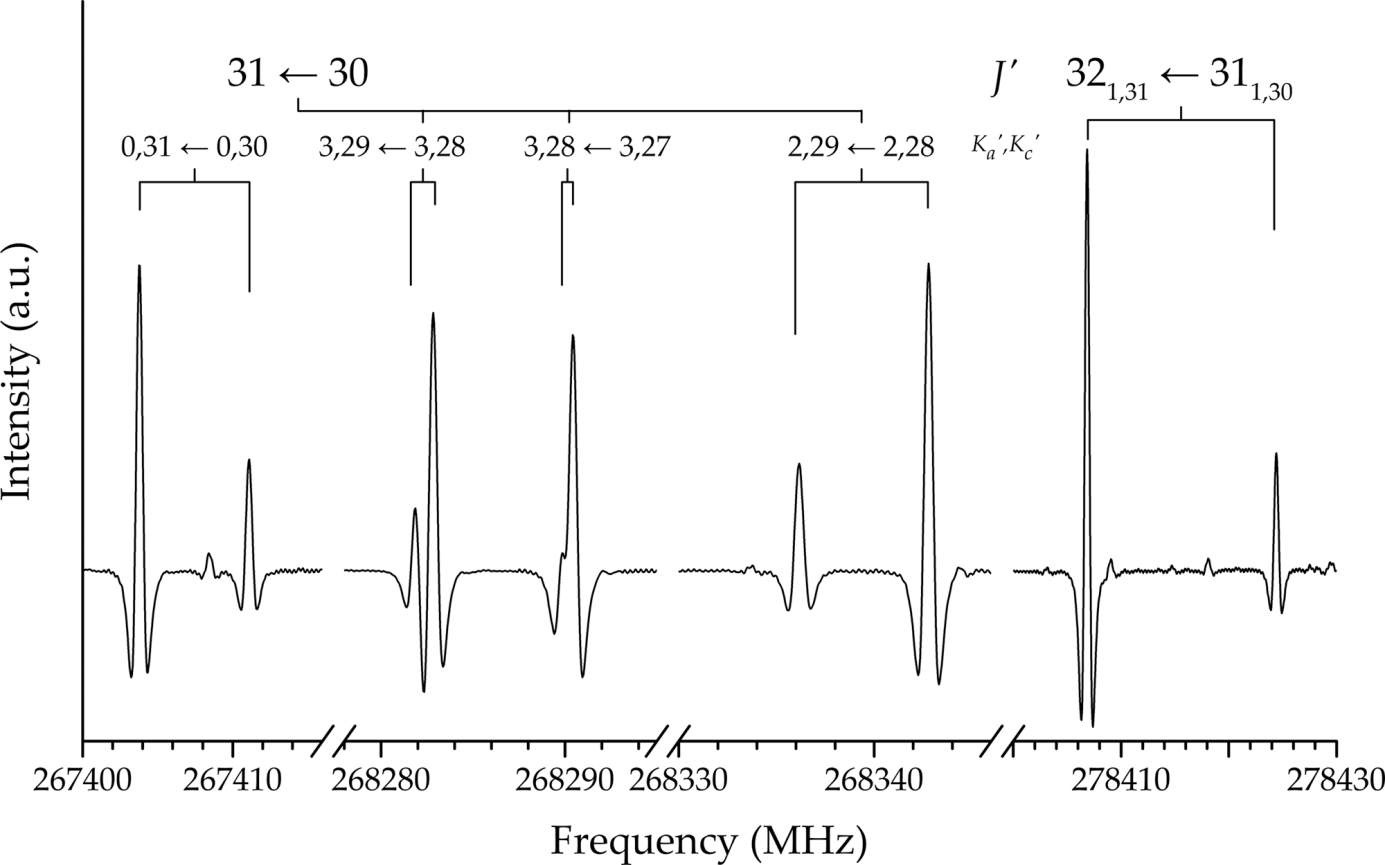
Portions of the experimental spectrum of propadienone showing the splitting due to the inversion motion.

Guided by the spectroscopic data available in the literature^32,49,60,61^ complemented by our computational study (see the Computational Methods), we successfully recorded the rotational spectrum of propadienone in selected ranges in the 80–115, 240–315, and 350–400 GHz frequency intervals. Figure 2 shows small portions of the experimental spectrum, where the tunneling splittings are evident. About 300 newly assigned transitions have been merged with the available data set and analyzed in a global fit whose results are reported in Table 2. To determine statistical weights, an estimated uncertainty ranging between 10 and 60 kHz has been assigned to the lines measured in the present work according to their S/N and the resolution of the inversion doublets. For the data taken from ref (49), we retained the uncertainties reported in the original study, which vary in the 5–50 kHz interval.

**Table 2:**
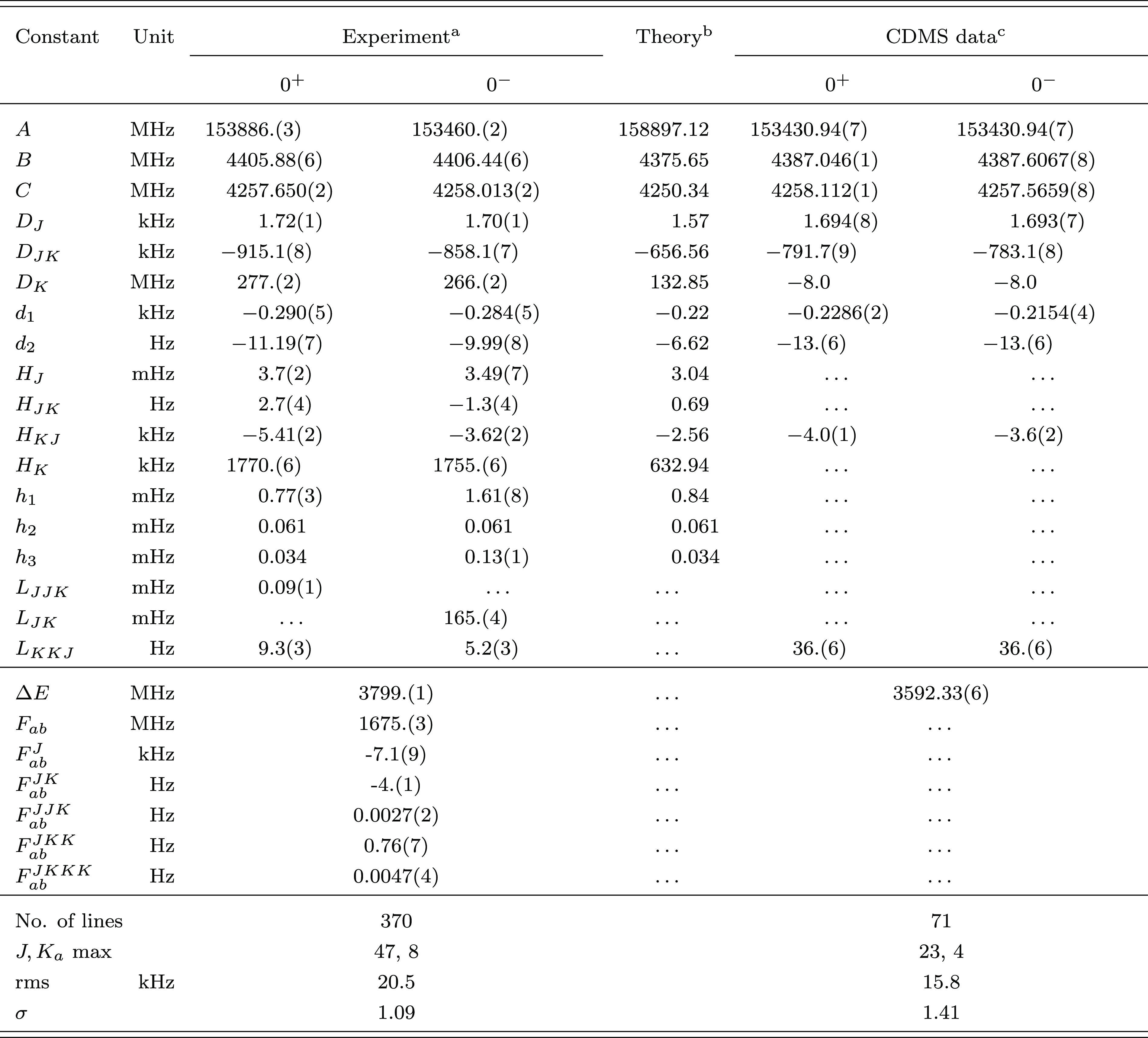
Ground-State Spectroscopic Parameters of Propadienone

a Values in parentheses are one standard deviation in units of the last quoted digit. Constants without error are fixed to the corresponding computed value.

b Equilibrium CCSD(T)/CBS+CV rotational constants augmented by vibrational corrections at the B2PLYP-D3(BJ)/aug-cc-pVTZ level. Quartic and sextic centrifugal distortion constants at the CCSD(T)/cc-pCVQZ and B2PLYP-D3(BJ)/jun-cc-pVTZ levels of theory, respectively.

c Based on ref (49).

**Figure 3:**
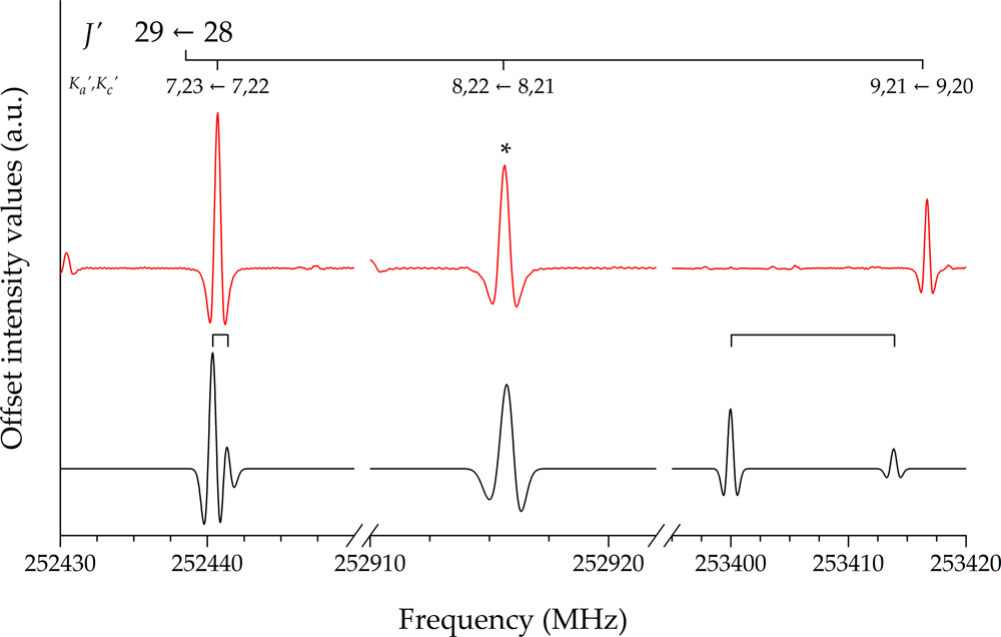
Portion of the experimental (black) and simulated (red) rotational spectra of propadienone. Only the transition marked with an asterisk was included in the fit.

The spectral analysis and fitting procedure were straightforward. The rotational-inversion Hamiltonian described by Brown et al.^49^ was able to reproduce—with some difficulties and a few exceptions—most of the *a*-type transitions between energy levels with *K*_*a*_ ≤ 8. At first, the tunneling splitting of several transitions was poorly predicted by our simulation, with the observed splittings being either underestimated or overestimated (in the former case resulting in blended features). This discrepancy has been overcome by including additional centrifugal distortion dependencies in the analysis. However, due to the limitations in the Hamiltonian, we can observe some discrepancies between the theoretical and experimental values of centrifugal distortion terms, and some of them are poorly determined. Furthermore, the use—for each state—of different higher order centrifugal distortion terms was required for reproducing the experimental data with proper accuracy. The final simulation provided reliable predictions only up to, as mentioned above, *K*_*a*_ = 8, with very few pathological cases such as the 29_7,23_ ← 28_7,22_ transition depicted in Figure 3. This was predicted to be partially resolved in contrast with the experimental evidence. In such cases, the lines were excluded from the analysis even though the assignment was undoubted. Figure 3 also provides a clear example of the unreliability of the prediction for transitions with *K*_*a*_ > 8: the simulation does not reproduce the experimental counterpart either on the frequency scale or in terms of tunneling splitting. However, the intensity and position of the line detected in the actual spectrum strongly support its identification as the 29_9,21_ ← 28_9,20_ transition. Therefore, our recommendation is to consider cautiously our spectral predictions beyond the maximum *J* and *K*_*a*_ values investigated in this work. Despite the difficulties encountered in the analysis, our results still represent a significant improvement in the characterization of the propadienone rotational spectrum.

Concerning the energetic characterization of the [H_2_C_3_O] family, our results are in agreement with previous findings,^53^ thus confirming propadienone as the most stable isomer. The relative ZPE-corrected CCSD(T)/CBS+CV energies (evaluated on top of B2PLYP-D3(BJ)/aug-cc-pVTZ reference geometries) of propynal and cyclopropenone, with respect to propadienone, are 2.1  and 30.0 kJ mol^–1^, respectively. The final ZPE correction has been obtained by correcting the CCSD(T)/cc-pCVQZ harmonic ZPE for the B2PLYP-D3(BJ)/aug-cc-pVTZ anharmonic contribution. The energy barrier for the inversion motion has been estimated at the CCSD(T)/CBS+CV level to be about 350 cm^–1^, in agreement with the value of 359(10) cm^–1^ reported by Brown et al.^49^

### Cyanovinylacetylene

Both isomers of cyanovinylacetylene (also known as 2-penten-4-ynenitrile) are planar asymmetric-top rotors, with asymmetry values of κ = −0.993 and −0.713 for the *E* and *Z* form, respectively. While the latter isomer is predicted to have *a*- and *b*-type spectra of similar intensity (μ_*a*_ = 2.60 D, μ_*b*_ = 2.72 D), according to B2PLYP-D3(BJ)/jun-cc-pVTZ calculations, the *E* isomer spectrum should be dominated by *a*-type transitions (μ_*a*_ = 4.06 D, μ_*b*_ = 0.61 D). In previous works, the rotational spectrum of the former has been characterized up to ∼40 GHz,^33,64^ while that of the latter only up to 15 GHz.^65^ As done in literature studies, we relied on the gas-phase pyrolysis of 2,3-pyridinedicarboxylic anhydride for the production of cyanovinylacetylene. In detail, the solid precursor was kept in a glass tube and heated in order to provide a suitable amount of vapors inside the pyrolysis apparatus. The optimal temperature for this heating stage was found to be ∼120 °C. The pyrolysis temperature was then optimized in order to have the maximum yield of cyanovinylacetylene, thus leading to working values of 950–1000 °C. The pyrolysis products were continuously flown into the absorption cell, with the pumping system maintaining a constant pressure of 6–7 mTorr inside it. The experimental conditions are summarized in Table 1.

To support our measurements, the available experimental data (taken from CDMS^56,57^) have been complemented with our computed spectroscopic parameters. Indeed, from the previous works on (*E*)-cyanovinylacetylene,^33,64^ only an incomplete set of quartic centrifugal distortion constants was determined together with the rotational constants. In addition, large uncertainties affected the experimental values of *A* and *d*_1_. Instead, all rotational and centrifugal distortion constants were already available for the *Z* isomer.^65^ Because of the presence of ^14^N, which is a quadrupolar nucleus (*I* = 1), the rotational transitions of both isomers show a hyperfine structure due to the coupling between the nitrogen quadrupole moment and the electric field gradient at the nucleus produced by the molecular rotation. The hyperfine structure, resolved for both isomers and analyzed in low-frequency studies,^64,65^ is completely collapsed in the spectral region investigated in our work and therefore its effect has been ignored in our analysis.

**Figure 4:**
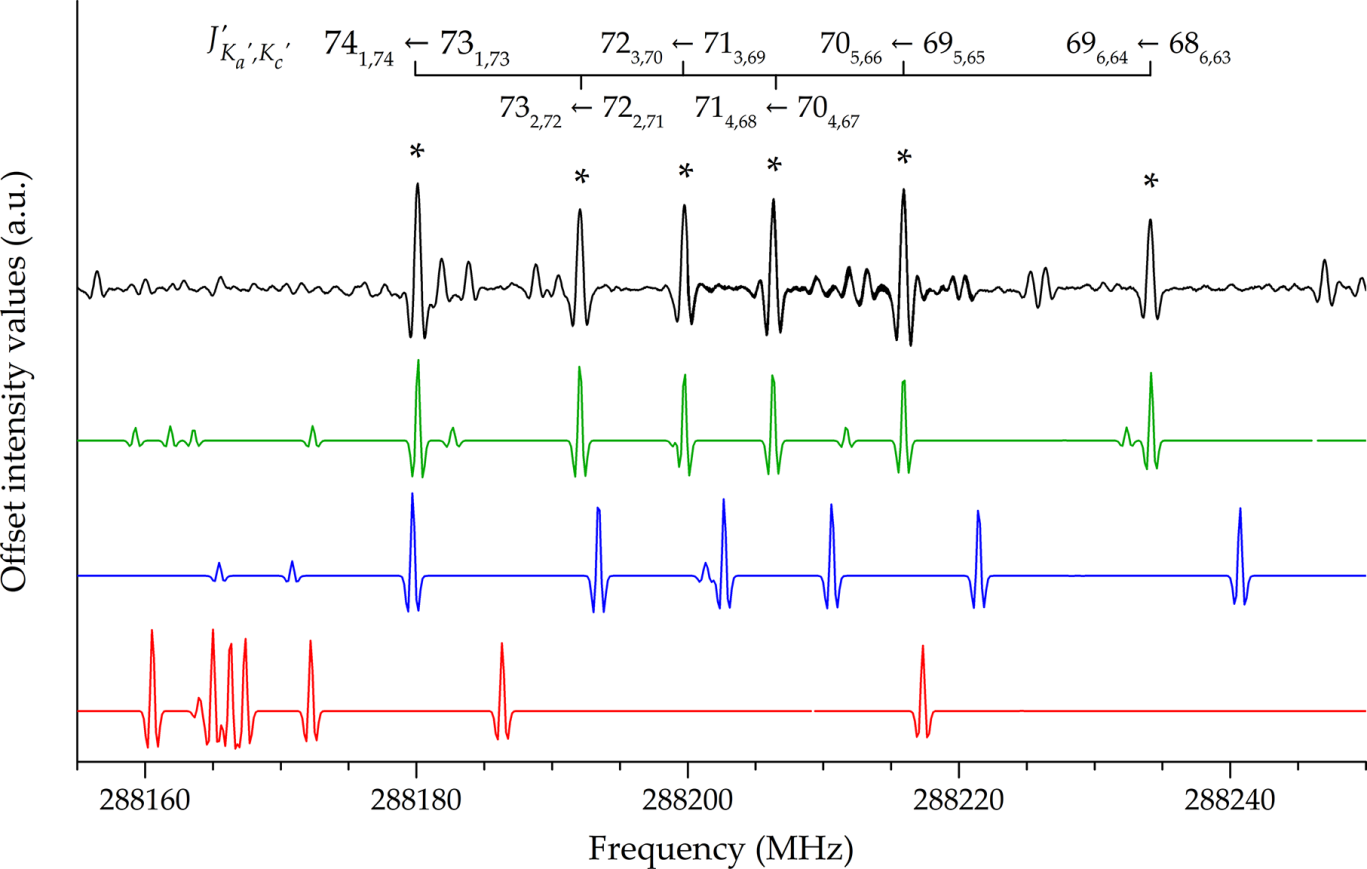
Comparison of different simulated spectra of (*Z*)-cyanovinylacetylene. In red: data from CDMS. In blue: data from CDMS augmented by computed sextic centrifugal distortion constants. In green: data from our final fit. In black: experimental spectrum. Only transitions marked with an asterisk have been assigned.

The importance of including additional centrifugal distortion parameters for the prediction of the rotational spectrum at higher frequencies is shown in Figure 4 for (*Z*)-cyanovinylacetylene. It is noted that the agreement of the simulated spectrum with the recorded counterpart noticeably improves when taking the computed sextic centrifugal distortion constants into account. About 1000 and 400 lines have been recorded and analyzed for (*E*)- and (*Z*)-cyanovinylacetylene, respectively, in the 80–115 and 245–400 GHz ranges. Our data have been merged with the rotational transitions available from literature in a global fit. For the literature data, statistical weights were derived using the uncertainties reported in the original papers,^33,64,65^ while an uncertainty of 10–30 kHz (depending on the line width) was assigned to the frequencies measured in the present work.

The resulting parameters are reported in Table 3 together with the corresponding theoretically computed values. An average discrepancy of 0.06% is found for the rotational constants of (*E*)-cyanovinylacetylene, while a larger deviation (close to 0.3%) is observed for the *Z* isomer. The agreement between ab initio and experimental centrifugal distortion constants is somewhat different for the *E* and *Z* isomers. For the former, quartic and sextic coefficients compare fairly well with their theoretically computed counterparts, with mean deviations of 7% and 9%, respectively. The quartic centrifugal distortion constants show a good agreement (∼8%) for (*Z*)-cyanovinylacetylene as well, while larger deviations are observed for the sextic coefficients (∼35%). The worst case is *H*_*JK*_, whose experimental value (not accurately determined) disagrees both in size and in sign with the corresponding ab initio result.

**Table 3:**
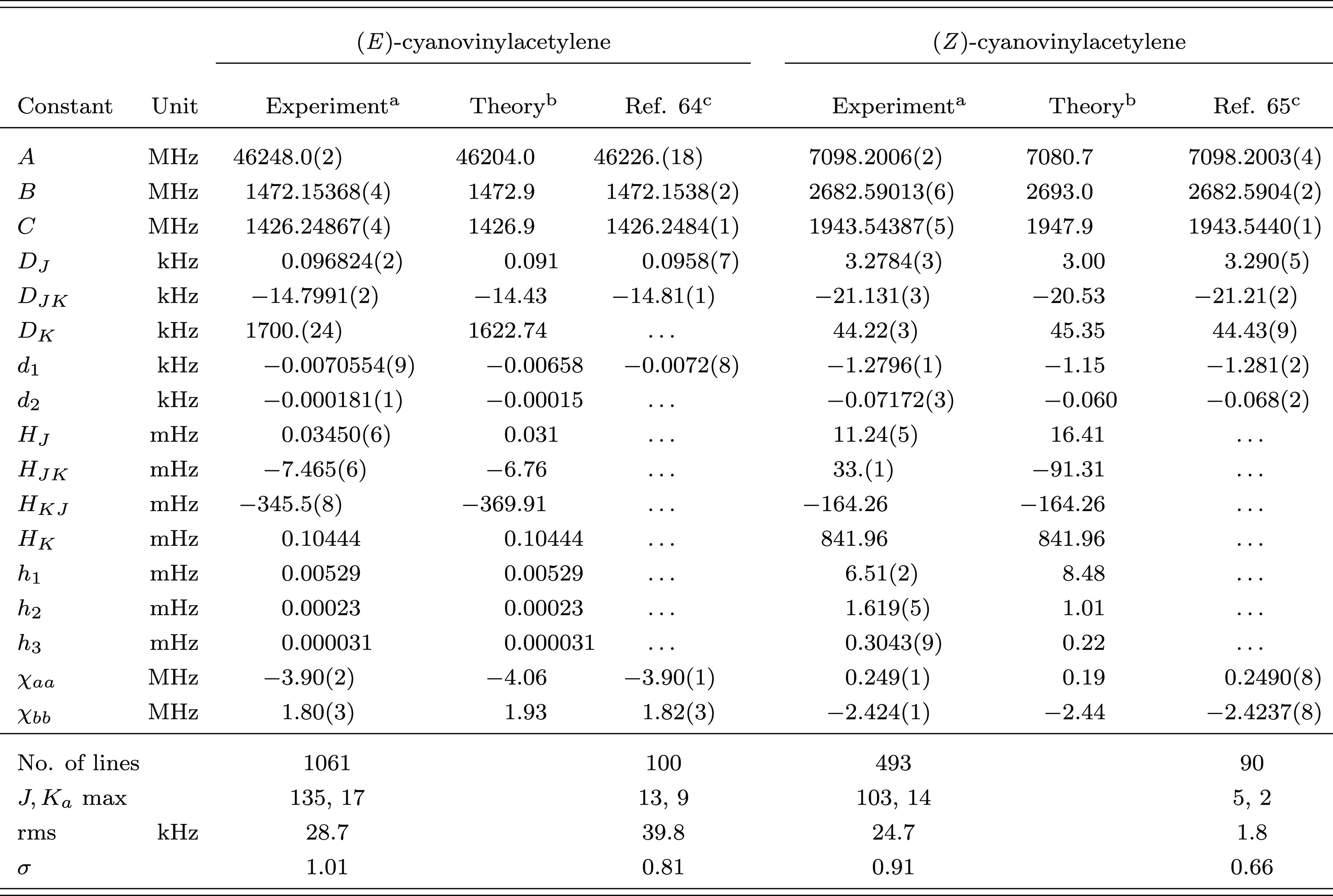
Ground-State Spectroscopic Parameters of (*E*)- and (*Z*)-Cyanovinylacetylene

a Values in parentheses are one standard deviation in units of the last quoted digit. Constants without error are fixed to the corresponding computed value.

b Equilibrium jun-ChS rotational constants augmented by vibrational corrections at the B2PLYP-D3(BJ)/jun-cc-pVTZ level. Quartic and sextic centrifugal distortion constants as well as nuclear quadrupole coupling constants at the B2PLYP-D3(BJ)/jun-cc-pVTZ level of theory.

c Reanalyzed with SPFIT(50) using the *S*-reduction.

The comparison with previous experimental results points out a clear improvement in the accuracy of the spectroscopic parameters as well as a more comprehensive characterization. The global fit for (*E*)-cyanovinylacetylene is well conditioned, with a final standard deviation (σ) ∼1 and a root-mean-square error (rms) of 28.7 kHz. Concerning (*Z*)-cyanovinylacetylene, the global fit has rms = 25.1 kHz and σ < 1. Based on these statistics, for both isomers, we can affirm that our global fits were able to well reproduce the experimental data.

For the sake of completeness, the energetic characterization of the cyanovinylacetylene isomers and vinylcyanoacetylene (4-penten-2-ynenitrile), all members of the [H_3_C_5_N] family, has been carried out. The results are in disagreement with those obtained in ref (21), where vinylcyanoacetylene is reported as the most stable species. We note that, if only the electronic energy is considered, vinylcyanoacetylene is the most stable isomer also at our level level of theory (jun-ChS). However, when the B2PLYP-D3(BJ)/jun-cc-pVTZ harmonic ZPE correction is added, the relative stability order changes. Vinylcyanoacetylene is found to be roughly 1 kJ mol^–1^ less stable than (*E*)-cyanovinylacetylene, while the *Z* isomer still remains higher in energy and lies 1.4 kJ mol^–1^ above the *E* isomer. If we consider the abundance of these species detected toward TMC-1,^21^ the column density values reported (namely, 2 × 10^11^ and 3 × 10^11^ cm^–2^ for vinylcyanoacetylene and *E*-cyanovinylacetylene, respectively) tend to suggest that the abundances of the [H_3_C_5_N] isomers are in agreement with their thermodynamic stability, thus following the minimum energy principle.

### Allenylacetylene

Allenylacetylene (H_2_CCCHCCH or 3,4-pentadiene-1-yne) is the simplest hydrocarbon featuring the C≡C triple bond and the allene C=C=C subunit, and its molecular structure is depicted in Figure 1. This species belongs to the *C*_*s*_ point group with the two hydrogen atoms of the CH_2_ moiety laying outside the symmetry plane. From the spectroscopic point of view, allenylacetylene is a nearly prolate asymmetric-top with κ = −0.983 and only one significant electric dipole component (μ_*a*_ = 0.72 D at the B2PLYP-D3(BJ)/jun-cc-pVTZ level of theory).

Rotational lines of allenylacetylene were first identified in the products of the plasma-induced decomposition of benzene by McCarthy et al.,^66^ who then comprehensively studied its centimeter-wave spectrum using Fourier-transform microwave (FTMW) spectroscopy. After this laboratory characterization, allenylacetylene was detected in the dark cloud TMC-1 by Cernicharo et al.,^67^ who observed 19 transitions in the *Q*-band (31–50 GHz), with the maximum value of *K*_*a*_ ≤ 3 and obtained improved values for the rotational constants and three quartic centrifugal distortion parameters.

**Figure 5:**
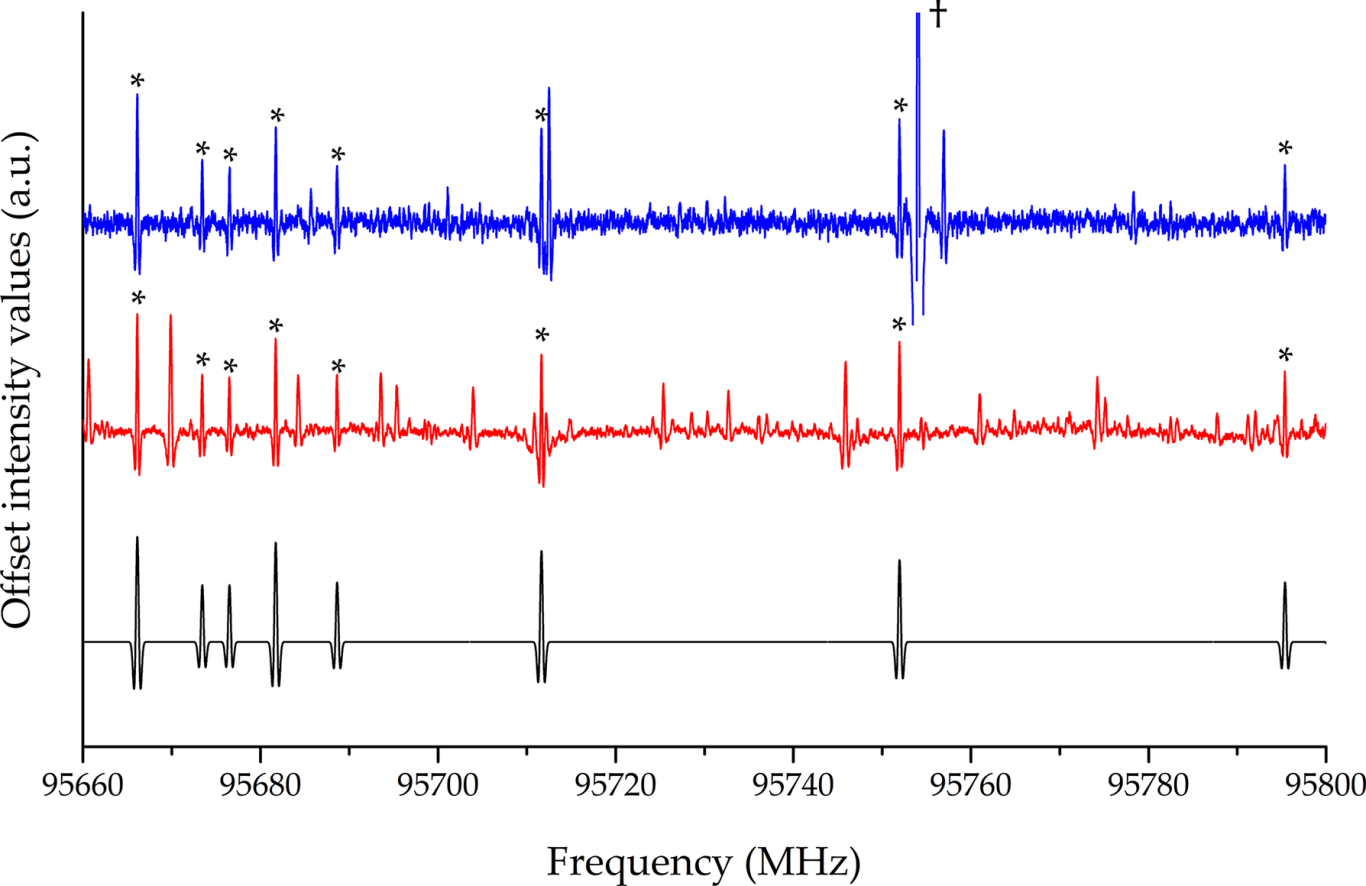
Portion of the rotational spectrum of allenylacetylene using dipropargylamine (blue) and tripropargylamine (red) as the pyrolysis precursor, compared to the simulated (black) spectrum. Transitions marked with an asterisk were assigned to allenylacetylene. The strong interfering feature marked with a dagger is out of scale.

In our experiments, allenylacetylene has been produced in the gas phase by flash vacuum pyrolysis of two different precursors, namely, dipropargylamine and tripropargylamine. Both species are commercially available and were used without further purification. Vapors of the precursors were flown through the pyrolysis system which was set at a temperature of 800 °C, and the products were continuously pumped through the absorption cell kept at a pressure of ∼10 mTorr. A portion of the rotational spectra obtained using the two precursors is depicted in Figure 5. Dipropargylamine, the same precursor employed in the pyrolytic production of propargylimine,^19^ was found to generate the smallest amount of interfering side products while providing comparably strong allenylacetylene signals as tripropargylamine. Therefore, the former was used for the spectral recordings.

Given the availability of the spectroscopic parameters from ref (67), the assignment of the rotational transitions of allenylacetylene in the 75–115 GHz range was straightforwardly accomplished. Due to a gap in the frequency coverage of our spectrometer, we had to skip the 2 mm band and the measurements were resumed at 225 GHz. In this spectral region, the line positions predicted using the spectroscopic parameters obtained from low-frequency analysis were found to be rather inaccurate, with deviations of ∼3 MHz for *K*_*a*_ = 0 transitions and rapidly increasing with the *J* value. The inclusion of these lines in the spectral analysis did not significantly improve the centrifugal distortion description, as no transitions with *K*_*a*_ ≥ 1 could be unambiguously assigned. To improve the accuracy of our predictions, additional higher-order centrifugal distortion constants were incorporated and kept fixed in the analysis. In detail, these are the full set of computed sextic centrifugal distortion constants (see the Computational Methods section), and three higher-order terms (up to *J*^10^) experimentally determined for the isoelectronic cyanoallene molecule.^68,69^ This approach allowed us to identify almost 200 lines in the 225–315 GHz range with *J*_max_ = 67 and *K*_*a*_ as high as 13.

The final least-squares analysis was performed on a set of 313 transition frequencies, which includes all available literature data. For the determination of their statistical weights, we assigned uncertainties of 2 kHz and 10–20 kHz to the previous FTMW^66^ and astronomical^67^ measurements, respectively. For our data, we assumed an average uncertainty of 35 kHz. The resulting spectroscopic parameters are reported in Table 4, where they are compared with the quantum-chemical counterparts and the constants from ref (67). From this table, one sees that all rotational constants were improved (*A* by about 1 order of magnitude), and the full set of quartic centrifugal distortion constants were obtained together with four sextic and one (*L*_*KKJ*_) octic centrifugal distortion constants. *H*_*J*_, *h*_2_, and *h*_3_, could not be determined using the available data set and were constrained at their computed values. It is also noted that the centrifugal distortion terms taken from cyanoallene were no longer needed in the final analysis. A rms error of about 30 kHz together with a standard deviation smaller than 1 confirm that the derived spectroscopic parameters are able to reproduce recorded rotational transitions within their experimental uncertainty.

**Table 4:**
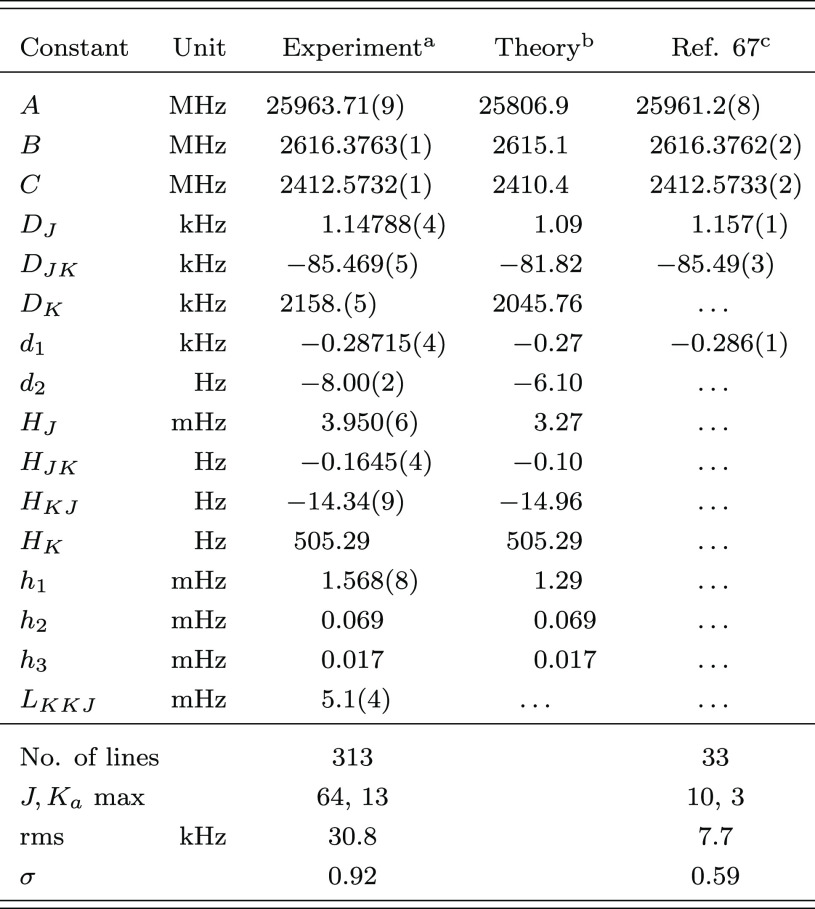
Ground-State Spectroscopic Parameters of Allenylacetylene

a Values in parentheses are one standard deviation in units of the last quoted digit. Constants without error are fixed to the corresponding computed value.

b Equilibrium jun-ChS rotational constants augmented by vibrational corrections at the B2PLYP-D3(BJ)/jun-cc-pVTZ level. Quartic and sextic centrifugal distortion constants at the B2PLYP-D3(BJ)/jun-cc-pVTZ level of theory.

c Reanalyzed with SPFIT(50) using the *S*-reduction.

As expected for hydrocarbons, the electric dipole moment of allenylacetylene is rather small, with the μ_*a*_ and μ_*b*_ components being about 0.72 and 0.01 D respectively, at the B2PLYP-D3(BJ)/jun-cc-pVTZ level of theory. These are in agreement with the values determined in previous works^67,70^ and explain why only the *a*-type spectrum could be observed.

As a last note, good agreement has been found between experimental and theoretical spectroscopic parameters, with an average deviation on the rotational and quartic centrifugal distortion constants of about 0.3% and 9%, respectively. Larger discrepancies are noted for sextic centrifugal distortion constants, this being mostly related to the small magnitude of these parameters. A comment on the sextic terms kept fixed in the fitting procedure is warranted. It has to be noted that their inclusion does not significantly affect the fit.

## Conclusions

This manuscript reports a thorough spectroscopic characterization of three unsaturated carbon chains important for astrochemistry: propadienone, cyanovinylacetylene, and allenylacetylene. The last two species have already been identified in the interstellar medium, while propadienone—the most stable isomer of the [H_2_C_3_O] family—is still undetected in space. These molecules have been produced in laboratory using gas-phase pyrolysis. Their rotational spectra have been investigated in the millimeter-/submillimeter-wave regime, with about 2000 new rotational transitions being recorded. The spectral analyses and line assignments have been guided by the available spectroscopic parameters and high-level quantum-chemical calculations for missing information. The latter also provided suitable constraints for those spectroscopic parameters that could not be directly determined from the fit of the experimental data.

The rotational spectrum of propadienone is complicated by a large amplitude motion that exchanges two equivalent structural configurations. Following earlier studies,^49^ the analysis has been performed by assigning the components of the tunneling doublets to separate states, each modeled with a conventional Watson-type Hamiltonian together with a set of suitable interaction parameters. However, such a simple approach presents some limitations and a few lines recorded in this work could not be satisfactorily reproduced. The inherent nonrigid nature of the propadienone suggests that the analysis would benefit from the use of a more sophisticated model explicitly accounting for the tunnelling motion together with the end-over-end molecular rotation. In this respect, an example is provided by the recent revision of the spectroscopy of the CH_2_OH radical.^71^

Both isomers (*E*/*Z*) of cyanovinylacetylene behave as semirigid molecules. Thus, their rotational spectra can be accurately predicted over an extensive frequency range and quantum number values from the spectroscopic parameters determined in this work. The present investigation then provides a well-sounded base for astronomical searches of the *Z* isomer in the ISM. Indeed, despite being less stable than the *E* form, its presence in the ISM cannot be ruled out. Recent findings on imines^72^ have demonstrated that both geometrical isomers can coexist in the cold interstellar gas and that the *E*/*Z* ratio might be enhanced by quantum tunneling through the isomerization barrier.

Concerning allenylacetylene, the extension of its spectral investigation well into the millimeter-wave region has proven to be essential for the set up of a reliable catalog of rest frequencies. For this molecule, centrifugal distortion effects have been found to be particularly prominent, as highlighted by the large value of the *D*_*K*_ constant (∼2 MHz, also confirmed by our accurate ab initio calculations). This seems to indicate some degree of floppiness along the *a* principal axis, with the rigid allenyl and ethynyl moieties becoming more and more aligned as the centrifugal force increases. In such a pathological case, extrapolation to higher frequency from microwave measurements leads to completely unreliable predictions, thus pointing out the importance of the present work.

## Supporting Information Available

Optimized geometries of all stationary points; harmonic frequencies of all minima; NMR spectrum of allenylacetylene; list of recorded transitions and their residuals from the final fit (PDF)
