# Supplementary material for: Rotational Spectra of Unsaturated Carbon Chains Produced by Pyrolysis: The Case of Propadienone, Cyanovinylacetylene, and Allenylacetylene
Source: J Phys Chem A. 2022 Aug 31;126(36):6210–20. doi: 10.1021/acs.jpca.2c05018 (PMC9483987; doi:10.1021/acs.jpca.2c05018)
Supplement: Supplementary file 1 — jp2c05018_si_001.pdf [file jp2c05018_si_001.pdf]

Supporting Information: Rotational Spectra of Unsaturated Carbon-Chains Produced by Pyrolysis: The Case of Propadienone, Cyanovinylacetylene, and Allenylacetylene

Alessio Melli,[†,‡] Mattia Melosso,[*,†,¶] Luca Bizzocchi,[†] Silvia Alessandrini,[†,‡] Ningjing Jiang,[†] Francesca Tonolo,[†,‡] Salvatore Boi,[†] Giorgia Castellan,[†] Carlotta Sapienza,[†] Jean-Claude Guillemin,[§] Luca Dore,[†] and Cristina Puzzarini[*,†]

†*Dipartimento di Chimica "Giacomo Ciamician", Università di Bologna, Via F. Selmi 2, 40126 Bologna, Italy*

‡*Scuola Normale Superiore, Piazza dei Cavalieri 7, 56126 Pisa, Italy*

¶*Scuola Superiore Meridionale, Largo San Marcellino 10, 80138 Naples, Italy*

§*Univ Rennes, Ecole Nationale Supérieure de Chimie de Rennes, CNRS, ISCR–UMR6226, F-35000 Rennes, France*

E-mail: mattia.melosso@unina.it; cristina.puzzarini@unibo.it

Optimized geometries of stationary points

In this section, the optimized geometries of the investigated species are collected, with internal coordinates being employed. The atom numbering is shown in Figure S1. Valence and dihedral angles that have been kept fixed in the geometry optimization (0 or 180 degrees) are not reported. Therefore, to avoid ambiguities in *quasi*-linear moieties, the convex angles have been highlighted in Figure S1. The levels of theory employed are detailed in the manuscript and references therein.

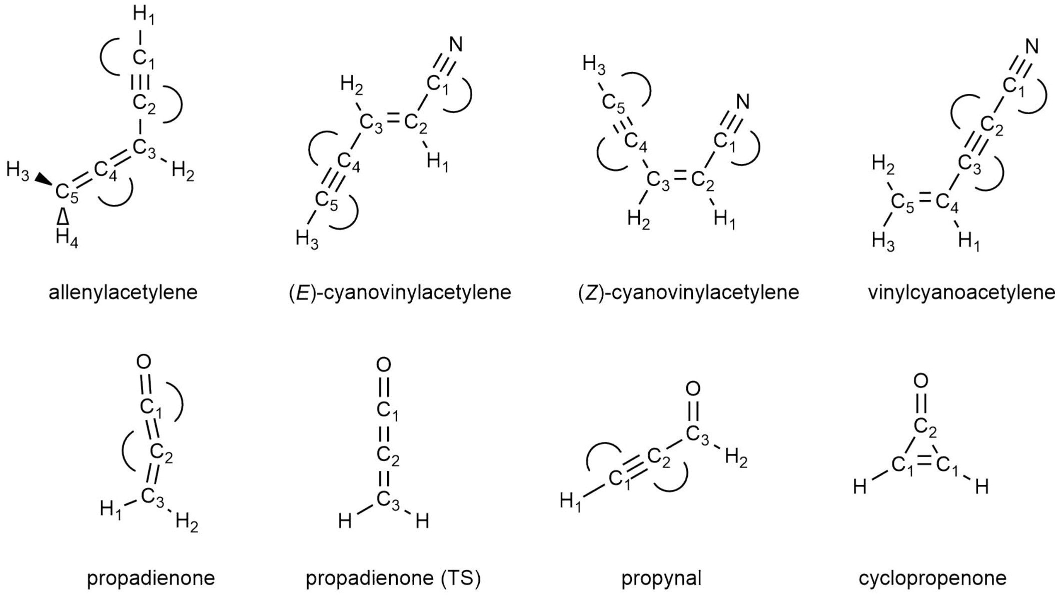

Figure S1: Atom numbering of the species studied in this work. The highlighted angles are convex.

Table S1: Computed molecule structure of allenylacetylene. Bonds in Å, angles in degrees.

Parameter	jun-ChS	B2PLYP-D3(BJ)/ jun-cc-pVTZ
r(H2-C3)	1.0829	1.0843
r(C2-C3)	1.4275	1.4229
r(C4-C3)	1.3147	1.3133
r(C5-C4)	1.3039	1.3002
r(H3/4-C5)	1.0812	1.0820
r(C1-C2)	1.2064	1.2076
r(H1-C1)	1.0615	1.0604
∠(C2-C3-C4)	117.70	123.63
∠(C4-C3-H2)	119.19	118.87
∠(C5-C4-C3)	179.30	179.36
∠(H3/4-C5-C4)	120.87	121.11
∠(C1-C2-C3)	178.06	178.19
∠(H1-C1-C2)	179.31	179.40
∠(H3-C5-C4-C3)	90.21	90.19
∠(H4-C5-C4-C3)	−90.21	−90.19

Table S2: Computed molecule structure of (*E*)-cyanovinylacetylene. Bonds in Å, angles in degrees.

Parameter	jun-ChS	B2PLYP-D3(BJ)/ jun-cc-pVTZ
r(C1-N)	1.1580	1.1611
r(C2-C1)	1.4278	1.4221
r(H1-C2)	1.0797	1.0805
r(C3-C2)	1.3434	1.3447
r(H2-C3)	1.0817	1.0825
r(C4-C3)	1.4210	1.4148
r(C5-C4)	1.2065	1.2080
r(H3-C5)	1.0620	1.0612
∠(C2-C1-N)	179.30	178.88
∠(H1-C2-C1)	117.28	117.02
∠(C3-C2-C1)	121.35	121.94
∠(H2-C3-C2)	119.81	119.50
∠(C4-C3-C2)	122.58	123.24
∠(C5-C4-C3)	178.65	178.37
∠(H3-C5-C4)	179.50	179.55

Table S3: Computed molecule structure of (*Z*)-cyanovinylacetylene. Bonds in Å, angles in degrees.

Parameter	jun-ChS	B2PLYP-D3(BJ)/ jun-cc-pVTZ
r(C1-N)	1.1576	1.1609
r(C2-C1)	1.4278	1.4221
r(H1-C2)	1.0791	1.0798
r(C3-C2)	1.3440	1.3458
r(H2-C3)	1.0811	1.0818
r(C4-C3)	1.4198	1.4137
r(C5-C4)	1.2064	1.2078
r(H3-C5)	1.0621	1.0612
∠(C2-C1-N)	178.76	178.56
∠(H1-C2-C1)	116.87	116.71
∠(C3-C2-C1)	122.51	123.16
∠(H2-C3-C2)	118.70	118.26
∠(C4-C3-C2)	123.81	124.50
∠(C5-C4-C3)	179.46	179.17
∠(H3-C5-C4)	179.19	179.19

Table S4: Computed molecule structure of vinylcyanoacetylene. Bonds in Å, angles in degrees.

Parameter	B2PLYP-D3(BJ)/ jun-cc-pVTZ
r(C1-N)	1.1652
r(C1-C2)	1.3647
r(C2-C3)	1.2141
r(C3-C4)	1.4163
r(C4-C5)	1.3377
r(H1-C4)	1.0826
r(H2-C5)	1.0797
r(H3-C5)	1.0791
∠(N-C1-C2)	180.00
∠(C1-C2-C3)	179.50
∠(C2-C3-C4)	178.63
∠(C5-C4-C3)	123.34
∠(H1-C4-C3)	116.31
∠(H2-C5-C4)	121.47
∠(H3-C5-C4)	120.53

Table S5: Computed molecule structure of propadienone. Bonds in Å, angles in degrees.

Parameter	B2PLYP-D3(BJ)/		CCSD(T)/	CCSD(T)/
	jun-cc-pVTZ	aug-cc-pVTZ	CBS+CV	cc-pCVQZ
r(C1-O)	1.1704	1.1704	1.1657	1.1661
r(C2-C1)	1.3007	1.3003	1.3046	1.3064
r(C3-C2)	1.3266	1.3263	1.3290	1.3301
r(H2-C3)	1.0838	1.0838	1.0829	1.0831
r(H1-C3)	1.0858	1.0859	1.0845	1.0846
∠(C2-C1-O)	169.08	169.17	168.28	168.01
∠(C3-C2-C1)	144.61	144.80	143.31	142.75
∠(H2-C3-C2)	121.13	121.15	120.81	120.80
∠(H1-C3-C2)	122.74	122.72	122.52	122.60

Table S6: Computed molecule structure of propadienone inversion transition state. Bonds in Å, angles in degrees.

Parameter	B2PLYP-D3(BJ)/ aug-cc-pVTZ	CCSD(T)/ CBS+CV
r(C1-O)	1.1776	1.1736
r(C1-C2)	1.2740	1.2756
r(C3-C2)	1.3166	1.3194
r(C3-H)	1.0861	1.0847
∠(H-C3-C2)	121.97	121.64

Table S7: Computed molecule structure of propynal. Bonds in Å, angles in degrees.

Parameter	B2PLYP-D3(BJ)/ aug-cc-pVTZ	CCSD(T)/ CBS+CV	CCSD(T)/ cc-pCVQZ
r(H1-C1)	1.0615	1.0631	1.0629
r(C1-C2)	1.2071	1.2065	1.2072
r(C3-C2)	1.4452	1.4502	1.4518
r(O-C3)	1.2121	1.2077	1.2079
r(H2-C3)	1.1002	1.0984	1.0986
∠(H1-C1-C2)	178.89	178.89	178.78
∠(C1-C2-C3)	177.68	177.73	177.58
∠(O-C3-C2)	123.70	123.32	123.39
∠(H2-C3-C2)	114.84	115.11	114.97

Table S8: Computed molecule structure of cyclopropenone. Bonds in Å, angles in degrees.

Parameter	B2PLYP-D3(BJ)/ aug-cc-pVTZ	CCSD(T)/ CBS+CV	CCSD(T)/ cc-pCVQZ
r(C2-O)	1.2045	1.2002	1.2000
r(C1-C2)	1.4289	1.4279	1.4302
r(H-C1)	1.0790	1.0785	1.0787
∠(C1-C2-O)	151.94	151.92	151.94
∠(H-C1-C2)	153.89	153.70	153.78

Harmonic frequency vibrations of minima

In this section, the computed harmonic frequencies of the investigated species are reported. Fundamentals are ordered according to their symmetry. The levels of theory employed are detailed in the manuscript and references therein.

Table S9: Fundamental vibrational modes (cm^{-1}) of allenylacetylene, (*E*)-cyanovinylacetylene, (*Z*)-cyanovinylacetylene and vinylcyanoacetylene at the B2PLYP-D3(BJ)/jun-cc-pVTZ level of theory.

	allenylacetylene		(*E*)-cyanovinylacetylene		(*Z*)-cyanovinylacetylene		vinylcyanoacetylene	
Mode	Sym.	Freq.	Sym.	Freq.	Sym.	Freq.	Sym.	Freq.
ν_1	A'	3481.13	A'	3475.12	A'	3475.70	A'	3271.94
ν_2		3153.66		3210.14		3218.01		3181.89
ν_3		3143.53		3185.66		3189.04		3173.41
ν_4		2170.66		2259.81		2260.14		2309.30
ν_5		2030.19		2167.35		2169.51		2183.64
ν_6		1473.05		1652.00		1637.08		1658.63
ν_7		1354.76		1329.40		1420.99		1460.53
ν_8		1136.58		1295.93		1239.27		1325.81
ν_9		952.03		1039.13		1034.66		1202.67
ν_{10}		884.08		1023.78		894.19		1050.55
ν_{11}		672.84		677.85		735.34		683.81
ν_{12}		602.02		538.60		675.19		546.51
ν_{13}		351.99		512.19		456.06		500.47
ν_{14}		137.58		246.75		253.64		257.74
ν_{15}	A''	3222.24		130.60		102.58		106.78
ν_{16}		1009.85	A''	984.48	A''	987.91	A''	1008.80
ν_{17}		886.57		864.95		775.93		980.03
ν_{18}		638.22		666.03		673.24		695.37
ν_{19}		621.85		535.93		598.37		519.67
ν_{20}		345.58		359.56		362.09		338.60
ν_{21}		296.16		121.90		224.64		137.14

Table S10: Computed fundamental vibrational modes (in cm^{-1}) for the three most stable members of the [H_2C_3O] family.

		propadienone			cyclopropenone		propynal
Mode	Sym.	B2PLYP-D3(BJ)/ jun-cc-pVTZ	CCSD(T)/ cc-pCVQZ	Sym.	B2PLYP-D3(BJ)/ jun-cc-pVTZ	Sym.	B2PLYP-D3(BJ)/ jun-cc-pVTZ
ν_1	A'	3187.12	3200.53	A_1	3242.27	A'	3474.04
ν_2		3106.89	3114.97		1908.94		2984.53
ν_3		2189.30	2180.76		1542.85		2159.25
ν_4		1736.73	1722.77		1045.42		1719.04
ν_5		1496.58	1486.90		853.55		1419.36
ν_6		1084.73	1077.28	A_2	981.40		961.03
ν_7		943.53	937.97	B_1	760.85		677.31
ν_8		508.72	500.53		468.51		626.48
ν_9		171.38	179.75	B_2	3209.34		209.10
ν_{10}	A''	1034.68	1008.02		1160.48	A''	1010.28
ν_{11}		703.13	700.12		830.24		726.52
ν_{12}		276.86	270.67		520.27		269.73

NMR spectrum of allenylacetylene

Allenylacetylene was observed together with propargylimine in the pyrolysis of dipropargylamine at 800 °C , as already mentioned in Ref. 19. Allenylacetylene is formed with a yield of about 5%. The pyrolysis products have been condensed in a trap immersed in a liquid nitrogen bath, then heated up to room temperature for 10 min, and finally distilled in order to remove the low boiling point compounds. In this way, allenylacetylene has been obtained with a purity greater than 95%. In a similar process, a yield of 30% in allenylacetylene was obtained starting from tripropargylamine, but with much lower purity.

^{1}H NMR ($CDCl_3$, 400 MHz) δ 2.89 (dt, 1H, $^{4}J_{HH}$ = 2.6 Hz, $^{6}J_{HH}$ = 1.6 Hz, C≡C−H); 5.06 (dd, 2H, $^{4}J_{HH}$= 6.9 Hz, $^{6}J_{HH}$ = 1.6 Hz, CH_2); 5.37 (td, 1H, $^{4}J_{HH}$ = 6.9 Hz, $^{4}J_{HH}$ = 2.6 Hz, C=CH). ^{13}C NMR ($CDCl_3$, 100 MHz) δ 74.4 (d, $^{1}J_{CH}$ = 173.0 Hz, C=CH); 76.0 (d, $^{2}J_{CH}$ = 51.6 Hz, C≡CH); 77.4 (t, $^{1}J_{CH}$= 169.9 Hz, CH_2); 78.8 (d, $^{1}J_{CH}$ = 254.3 Hz, C≡C−H) ; 217.2 (s, C=C=C).

List of recorded transitions

In this section, the lists containing all analyzed transitions for each species are reported. In the following tables, "obs" refers to the experimental frequency (in MHz), "o-c" refers to the difference between the observed frequency and that calculated from the fitted parameters (in MHz), "err" refers to the experimental uncertainty (in MHz), "wt" refers to the weight for blended transitions. The quantum numbers section uses the following notations: J', K_a', K_c', J, K_a, K_c for allenylacetylene; J', K_a', K_c', v, F', J, K_a, K_c, v, F for cyanovinylacetylene (where $v = 0, 1$ denotes transitions which hyperfine structure is resolved or collapsed, respectively); J', K_a', K_c', v, J, K_a, K_c, v for propadienone ($v = 0, 1$ refer to the 0^+ and 0^- states, respectively).

propadienone (methyleneketene) Wed Jul 13 14:43:18 2022

/ instead of : below denotes (o-c)>3*err

									obs	o-c	error	blends o-c	wt	Notes
1:	16	0	16	1	15	1	15	0	111.6900	0.0068	0.050			Brown-87
2:	8	1	7	0	8	1	8	0	4639.2330	0.0093	0.016			Brown-87
3:	8	1	7	1	8	1	8	1	4679.2180	-0.0052	0.015			Brown-87
4:	9	1	8	0	9	1	9	0	5798.2860	0.0081	0.015			Brown-87
5:	15	1	15	1	16	0	16	0	7002.3970	-0.0374	0.045			Brown-87
6:	11	1	10	0	11	1	11	0	8501.5570	0.0037	0.005			Brown-87
7:	11	1	10	1	11	1	11	1	8575.0820	-0.0066	0.010			Brown-87
8:	1	0	1	0	0	0	0	0	8645.1590	0.0221	0.008	0.0094	0.25	Brown-87
9:	1	0	1	1	0	0	0	1	8645.1590	0.0050	0.008	0.0094	0.75	Brown-87
10:	14	1	14	0	15	0	15	1	9423.1000	-0.0036	0.008			Brown-87
11:	17	0	17	1	16	1	16	0	9699.0960	-0.0004	0.009			Brown-87
12:	12	1	11	0	12	1	12	0	10045.5330	0.0015	0.006			Brown-87
13:	12	1	11	1	12	1	12	1	10132.5340	0.0013	0.050			Brown-87
14:	13	1	12	0	13	1	13	0	11717.5550	0.0018	0.005			Brown-87
15:	18	0	18	0	17	1	17	1	12245.2030	0.0017	0.019			Brown-87
16:	14	1	13	0	14	1	14	0	13517.4640	0.0003	0.007			Brown-87
17:	14	1	13	1	14	1	14	1	13634.8760	0.0053	0.011			Brown-87
18:	15	1	14	0	15	1	15	0	15445.1000	0.0051	0.007			Brown-87
19:	15	1	14	1	15	1	15	1	15579.4580	-0.0001	0.006			Brown-87
20:	14	1	14	1	15	0	15	0	16546.6270	-0.0393	0.037			Brown-87
21:	2	1	2	1	1	1	1	1	17163.3910	0.0182	0.010			Brown-87
22:	2	1	2	0	1	1	1	0	17164.4850	0.0005	0.010			Brown-87
23:	2	0	2	0	1	0	1	0	17290.1790	0.0270	0.006	0.0026	0.25	Brown-87
24:	2	0	2	1	1	0	1	1	17290.1790	-0.0056	0.006	0.0026	0.75	Brown-87
25:	2	1	1	0	1	1	0	0	17422.3310	-0.0058	0.007			Brown-87
26:	2	1	1	1	1	1	0	1	17423.4350	-0.0058	0.010			Brown-87
27:	16	1	15	0	16	1	16	0	17500.2720	0.0081	0.007			Brown-87
28:	16	1	15	1	16	1	16	1	17652.7660	0.0014	0.005			Brown-87
29:	13	1	13	0	14	0	14	1	18904.1260	0.0108	0.009			Brown-87
30:	18	0	18	1	17	1	17	0	19337.9320	0.0092	0.009			Brown-87
31:	19	0	19	0	18	1	18	1	21946.1630	-0.0273	0.010			Brown-87
32:	18	1	17	0	18	1	18	0	21992.4100	-0.0001	0.007			Brown-87
33:	19	1	18	0	19	1	19	0	24428.9350	-0.0109	0.008			Brown-87
34:	3	1	3	1	2	1	2	1	25744.9500	0.0257	0.019			Brown-87
35:	3	1	3	0	2	1	2	0	25746.5910	-0.0022	0.008			Brown-87
36:	3	0	3	0	2	0	2	0	25934.9680	0.0442	0.007	0.0104	0.25	Brown-87
37:	3	0	3	1	2	0	2	1	25934.9680	-0.0008	0.007	0.0104	0.75	Brown-87
38:	3	2	2	1	2	2	1	1	25953.7640	-0.0022	0.010			Brown-87
39:	3	2	2	0	2	2	1	0	25953.8930	-0.0060	0.025			Brown-87
40:	3	2	1	1	2	2	0	1	25954.0970	-0.0089	0.010			Brown-87
41:	3	2	1	0	2	2	0	0	25954.2450	0.0137	0.025			Brown-87
42:	13	1	13	1	14	0	14	0	26036.2980	-0.0364	0.020			Brown-87
43:	3	1	2	0	2	1	1	0	26133.3410	-0.0034	0.007			Brown-87
44:	3	1	2	1	2	1	1	1	26135.0150	0.0144	0.016			Brown-87
45:	20	1	19	0	20	1	20	0	26992.1290	-0.0068	0.007			Brown-87
46:	12	1	12	0	13	0	13	1	28330.2650	-0.0028	0.008			Brown-87
47:	19	0	19	1	18	1	18	0	29026.8820	-0.0025	0.008			Brown-87
48:	21	1	20	0	21	1	21	0	29681.7140	-0.0038	0.011			Brown-87
49:	20	0	20	0	19	1	19	1	31696.8790	-0.0368	0.026			Brown-87
50:	22	1	21	0	22	1	22	0	32497.4170	0.0043	0.010			Brown-87
51:	4	1	4	1	3	1	3	1	34326.3160	0.0020	0.013			Brown-87
52:	4	1	4	0	3	1	3	0	34328.5450	0.0029	0.014			Brown-87
53:	4	0	4	0	3	0	3	0	34579.3770	0.0464	0.008	0.0066	0.25	Brown-87
54:	4	0	4	1	3	0	3	1	34579.3770	-0.0066	0.008	0.0066	0.75	Brown-87
55:	4	2	3	1	3	2	2	1	34604.7830	0.0031	0.013			Brown-87
56:	4	2	3	0	3	2	2	0	34604.9500	-0.0082	0.025			Brown-87
57:	4	2	2	1	3	2	1	1	34605.6260	-0.0030	0.013			Brown-87
58:	4	2	2	0	3	2	1	0	34605.7840	-0.0046	0.025			Brown-87
59:	4	3	2	1	3	3	1	1	34634.6670	0.0039	0.009	0.0038	0.50	Brown-87
60:	4	3	1	1	3	3	0	1	34634.6670	0.0036	0.009	0.0038	0.50	Brown-87
61:	4	3	2	0	3	3	1	0	34634.9450	-0.0070	0.010	-0.0071	0.50	Brown-87
62:	4	3	1	0	3	3	0	0	34634.9450	-0.0072	0.010	-0.0071	0.50	Brown-87

63:	4	1	3	0	3	1	2	0	34844.1640	0.0049	0.017			Brown-87
64:	4	1	3	1	3	1	2	1	34846.3640	-0.0032	0.016			Brown-87
65:	23	1	22	0	23	1	23	0	35438.9210	-0.0020	0.009			Brown-87
66/	12	1	12	1	13	0	13	0	35470.3200	-0.0677	0.022			Brown-87
67:	11	1	11	0	12	0	12	1	37700.5430	-0.0016	0.008			Brown-87
68:	20	0	20	1	19	1	19	0	38764.6350	-0.0069	0.009			Brown-87
69:	5	1	5	0	4	1	4	0	42910.2810	0.0034	0.011			Brown-87
70:	5	0	5	0	4	0	4	0	43223.3020	0.0509	0.014	0.0098	0.25	Brown-87
71:	5	0	5	1	4	0	4	1	43223.3020	-0.0039	0.014	0.0098	0.75	Brown-87
72:	5	3	3	1	4	3	2	1	43293.1360	0.0101	0.010	0.0097	0.50	Brown-87
73:	5	3	2	1	4	3	1	1	43293.1360	0.0093	0.010	0.0097	0.50	Brown-87
74:	5	3	3	0	4	3	2	0	43293.4790	-0.0058	0.010	-0.0062	0.50	Brown-87
75:	5	3	2	0	4	3	1	0	43293.4790	-0.0065	0.010	-0.0062	0.50	Brown-87
76:	5	4	1	1	4	4	0	1	43342.6750	0.0127	0.010	0.0128	0.50	Brown-87
77:	5	4	2	1	4	4	1	1	43342.6750	0.0127	0.010	0.0128	0.50	Brown-87
78:	5	4	1	0	4	4	0	0	43342.9470	0.0100	0.008	0.0100	0.50	Brown-87
79:	5	4	2	0	4	4	1	0	43342.9470	0.0100	0.008	0.0100	0.50	Brown-87
80:	5	1	4	0	4	1	3	0	43554.7160	-0.0001	0.010			Brown-87
81:	10	1	10	0	11	0	11	1	47013.9700	-0.0250	0.015			Brown-87
82:	21	0	21	1	20	1	20	0	48549.7760	-0.0171	0.010			Brown-87
83:	10	1	10	1	9	1	9	1	85808.2490	-0.0041	0.010			Melli+22
84:	10	1	10	0	9	1	9	0	85813.9070	0.0008	0.010			Melli+22
85:	10	0	10	1	9	0	9	1	86431.2620	0.0162	0.010	-0.0043	0.75	Melli+22
86:	10	0	10	0	9	0	9	0	86431.2620	-0.0659	0.010	-0.0043	0.25	Melli+22
87:	10	2	9	1	9	2	8	1	86504.6940	-0.0096	0.010			Melli+22
88:	10	2	9	0	9	2	8	0	86505.2090	0.0205	0.010			Melli+22
89:	10	2	8	1	9	2	7	1	86518.6810	0.0003	0.010			Melli+22
90:	10	3	8	1	9	3	7	1	86582.8710	0.0137	0.020	-0.0001	0.50	Melli+22
91:	10	3	7	1	9	3	6	1	86582.8710	-0.0139	0.020	-0.0001	0.50	Melli+22
92:	10	3	8	0	9	3	7	0	86583.5200	-0.0195	0.020	-0.0327	0.50	Melli+22
93:	10	3	7	0	9	3	6	0	86583.5200	-0.0458	0.020	-0.0327	0.50	Melli+22
94:	10	4	6	1	9	4	5	1	86681.3380	0.0003	0.020	0.0004	0.50	Melli+22
95:	10	4	7	1	9	4	6	1	86681.3380	0.0003	0.020	0.0004	0.50	Melli+22
96:	10	4	6	0	9	4	5	0	86681.8700	0.0082	0.020	0.0083	0.50	Melli+22
97:	10	4	7	0	9	4	6	0	86681.8700	0.0083	0.020	0.0083	0.50	Melli+22
98:	10	6	4	1	9	6	3	1	86940.1040	-0.1396	0.010	0.0050	0.37	Melli+22
99:	10	6	5	1	9	6	4	1	86940.1040	-0.1396	0.010	0.0050	0.37	Melli+22
100:	10	6	4	0	9	6	3	0	86940.1040	0.4385	0.010	0.0050	0.13	Melli+22
101:	10	6	5	0	9	6	4	0	86940.1040	0.4385	0.010	0.0050	0.13	Melli+22
102:									0.0000	0.2504	0.100			Melli+22
103:	10	7	3	1	9	7	2	1	87093.1470	-0.2631	0.010	-0.0177	0.12	Melli+22
104:	10	7	4	1	9	7	3	1	87093.1470	-0.2631	0.010	-0.0177	0.12	Melli+22
105:	10	7	3	0	9	7	2	0	87093.1470	0.0639	0.010	-0.0177	0.38	Melli+22
106:	10	7	4	0	9	7	3	0	87093.1470	0.0639	0.010	-0.0177	0.38	Melli+22
107:									0.0000	0.1415	0.100			Melli+22
108:	10	1	9	0	9	1	8	0	87101.3890	0.0013	0.010			Melli+22
109:	10	8	2	1	9	8	1	1	87258.1490	0.0363	0.010	0.0179	0.37	Melli+22
110:	10	8	3	1	9	8	2	1	87258.1490	0.0363	0.010	0.0179	0.37	Melli+22
111:	10	8	2	0	9	8	1	0	87258.1490	-0.0373	0.010	0.0179	0.13	Melli+22
112:	10	8	3	0	9	8	2	0	87258.1490	-0.0373	0.010	0.0179	0.13	Melli+22
113:									0.0000	0.0319	0.100			Melli+22
114:	11	1	11	1	10	1	10	1	94387.0030	-0.0102	0.020			Melli+22
115:	11	1	11	0	10	1	10	0	94393.2550	0.0005	0.020			Melli+22
116:	11	0	11	1	10	0	10	1	95069.6710	0.0236	0.020	-0.0138	0.75	Melli+22
117:	11	0	11	0	10	0	10	0	95069.6710	-0.1261	0.020	-0.0138	0.25	Melli+22
118:	11	2	10	1	10	2	9	1	95153.1720	-0.0113	0.020			Melli+22
119:	11	2	10	0	10	2	9	0	95153.7470	0.0196	0.020			Melli+22
120:	11	2	9	1	10	2	8	1	95171.8150	0.0075	0.020	-0.0247	0.75	Melli+22
121:	11	2	9	0	10	2	8	0	95171.8150	-0.1213	0.020	-0.0247	0.25	Melli+22
122:	11	3	9	1	10	3	8	1	95240.1180	0.0244	0.020	0.0020	0.50	Melli+22
123:	11	3	8	1	10	3	7	1	95240.1180	-0.0204	0.020	0.0020	0.50	Melli+22
124:	11	3	9	0	10	3	8	0	95240.8220	-0.0112	0.020	-0.0326	0.50	Melli+22
125:	11	3	8	0	10	3	7	0	95240.8220	-0.0539	0.020	-0.0326	0.50	Melli+22
126:	11	4	8	1	10	4	7	1	95348.2440	0.0009	0.020	0.0009	0.50	Melli+22
127:	11	4	7	1	10	4	6	1	95348.2440	0.0008	0.020	0.0009	0.50	Melli+22
128:	11	4	7	0	10	4	6	0	95348.8220	0.0099	0.020	0.0099	0.50	Melli+22
129:	11	4	8	0	10	4	7	0	95348.8220	0.0099	0.020	0.0099	0.50	Melli+22
130:	11	6	5	0	10	6	4	0	95632.7570	0.4891	0.020	0.0016	0.12	Melli+22
131:	11	6	6	0	10	6	5	0	95632.7570	0.4891	0.020	0.0016	0.12	Melli+22

132:	11	6	5	1	10	6	4	1	95632.7570	-0.1609	0.020	0.0016	0.38	Melli+22
133:	11	6	6	1	10	6	5	1	95632.7570	-0.1609	0.020	0.0016	0.38	Melli+22
134:									0.0000	0.2815	0.100			Melli+22
135:	11	7	4	0	10	7	3	0	95801.0690	0.0879	0.010	-0.0090	0.38	Melli+22
136:	11	7	5	0	10	7	4	0	95801.0690	0.0879	0.010	-0.0090	0.38	Melli+22
137:	11	7	4	1	10	7	3	1	95801.0690	-0.3000	0.010	-0.0090	0.12	Melli+22
138:	11	7	5	1	10	7	4	1	95801.0690	-0.3000	0.010	-0.0090	0.12	Melli+22
139:									0.0000	0.1679	0.100			Melli+22
140:	11	1	10	0	10	1	9	0	95809.0540	0.0056	0.010			Melli+22
141:	11	1	10	1	10	1	9	1	95815.1250	0.0000	0.010			Melli+22
142:	11	8	3	0	10	8	2	0	95982.5330	-0.0078	0.010	0.0077	0.13	Melli+22
143:	11	8	4	0	10	8	3	0	95982.5330	-0.0078	0.010	0.0077	0.13	Melli+22
144:	11	8	3	1	10	8	2	1	95982.5330	0.0128	0.010	0.0077	0.37	Melli+22
145:	11	8	4	1	10	8	3	1	95982.5330	0.0128	0.010	0.0077	0.37	Melli+22
146:									0.0000	0.0089	0.100			Melli+22
147:	12	1	12	1	11	1	11	1	102965.1820	-0.0043	0.010			Melli+22
148:	12	1	12	0	11	1	11	0	102972.0270	0.0045	0.010			Melli+22
149:	12	0	12	1	11	0	11	1	103706.7120	0.0070	0.010			Melli+22
150:	12	2	11	1	11	2	10	1	103801.0910	-0.0042	0.010			Melli+22
151:	12	2	11	0	11	2	10	0	103801.7310	0.0293	0.010			Melli+22
152:	12	2	10	1	11	2	9	1	103825.3040	0.0143	0.010	-0.0022	0.75	Melli+22
153:	12	2	10	0	11	2	9	0	103825.3040	-0.0518	0.010	-0.0022	0.25	Melli+22
154:	12	3	10	1	11	3	9	1	103897.0540	0.0262	0.010	-0.0087	0.50	Melli+22
155:	12	3	9	1	11	3	8	1	103897.0540	-0.0436	0.010	-0.0087	0.50	Melli+22
156/	12	3	10	0	11	3	9	0	103897.8070	-0.0149	0.010	-0.0482	0.50	Melli+22
157:	12	3	9	0	11	3	8	0	103897.8070	-0.0814	0.010	-0.0482	0.50	Melli+22
158:	12	4	9	1	11	4	8	1	104014.7990	0.0015	0.010	0.0015	0.50	Melli+22
159:	12	4	8	1	11	4	7	1	104014.7990	0.0014	0.010	0.0015	0.50	Melli+22
160:	12	4	9	0	11	4	8	0	104015.4230	0.0136	0.010	0.0136	0.50	Melli+22
161:	12	4	8	0	11	4	7	0	104015.4230	0.0136	0.010	0.0136	0.50	Melli+22
162:	12	6	6	0	11	6	5	0	104325.0280	0.5468	0.010	0.0031	0.12	Melli+22
163:	12	6	7	0	11	6	6	0	104325.0280	0.5468	0.010	0.0031	0.12	Melli+22
164:	12	6	6	1	11	6	5	1	104325.0280	-0.1781	0.010	0.0031	0.38	Melli+22
165:	12	6	7	1	11	6	6	1	104325.0280	-0.1781	0.010	0.0031	0.38	Melli+22
166/									0.0000	0.3139	0.100			Melli+22
167:	12	7	5	0	11	7	4	0	104508.5950	0.1174	0.010	0.0039	0.38	Melli+22
168:	12	7	6	0	11	7	5	0	104508.5950	0.1174	0.010	0.0039	0.38	Melli+22
169:	12	7	5	1	11	7	4	1	104508.5950	-0.3371	0.010	0.0039	0.12	Melli+22
170:	12	7	6	1	11	7	5	1	104508.5950	-0.3371	0.010	0.0039	0.12	Melli+22
171:									0.0000	0.1968	0.100			Melli+22
172:	12	1	11	0	11	1	10	0	104516.0030	0.0023	0.010			Melli+22
173:	12	1	11	1	11	1	10	1	104522.6310	0.0005	0.010			Melli+22
174:	12	8	4	0	11	8	3	0	104706.5100	0.0305	0.010	-0.0033	0.13	Melli+22
175:	12	8	5	0	11	8	4	0	104706.5100	0.0305	0.010	-0.0033	0.13	Melli+22
176:	12	8	4	1	11	8	3	1	104706.5100	-0.0146	0.010	-0.0033	0.37	Melli+22
177:	12	8	5	1	11	8	4	1	104706.5100	-0.0146	0.010	-0.0033	0.37	Melli+22
178:									0.0000	0.0195	0.100			Melli+22
179:	13	1	13	1	12	1	12	1	111542.7100	-0.0104	0.010			Melli+22
180:	13	1	13	0	12	1	12	0	111550.1600	0.0013	0.010			Melli+22
181:	13	0	13	1	12	0	12	1	112342.3020	0.0027	0.010			Melli+22
182:	13	0	13	0	12	0	12	0	112342.6490	0.0114	0.010			Melli+22
183:	13	2	12	1	12	2	11	1	112448.3740	-0.0141	0.010			Melli+22
184:	13	2	12	0	12	2	11	0	112449.0790	0.0186	0.010			Melli+22
185:	13	2	11	1	12	2	10	1	112479.1630	0.0058	0.010	0.0098	0.75	Melli+22
186:	13	2	11	0	12	2	10	0	112479.1630	0.0217	0.010	0.0098	0.25	Melli+22
187:	13	3	11	1	12	3	10	1	112553.6950	0.0633	0.010	0.0110	0.50	Melli+22
188:	13	3	10	1	12	3	9	1	112553.6950	-0.0413	0.010	0.0110	0.50	Melli+22
189/	13	3	11	0	12	3	10	0	112554.4960	0.0188	0.010	-0.0309	0.50	Melli+22
190:	13	3	10	0	12	3	9	0	112554.4960	-0.0807	0.010	-0.0309	0.50	Melli+22
191:	13	4	10	1	12	4	9	1	112680.9520	-0.0167	0.010	-0.0168	0.50	Melli+22
192:	13	4	9	1	12	4	8	1	112680.9520	-0.0168	0.010	-0.0168	0.50	Melli+22
193:	13	4	10	0	12	4	9	0	112681.6190	-0.0023	0.010	-0.0024	0.50	Melli+22
194:	13	4	9	0	12	4	8	0	112681.6190	-0.0024	0.010	-0.0024	0.50	Melli+22
195:	13	5	8	0	12	5	7	0	112837.1100	0.0097	0.010	0.0097	0.50	Melli+22
196:	13	5	9	0	12	5	8	0	112837.1100	0.0097	0.010	0.0097	0.50	Melli+22
197:	13	6	7	0	12	6	6	0	113016.8830	0.6126	0.010	0.0108	0.12	Melli+22
198:	13	6	8	0	12	6	7	0	113016.8830	0.6126	0.010	0.0108	0.12	Melli+22
199:	13	6	7	1	12	6	6	1	113016.8830	-0.1898	0.010	0.0108	0.38	Melli+22
200:	13	6	8	1	12	6	7	1	113016.8830	-0.1898	0.010	0.0108	0.38	Melli+22

201:	13	7	6	0	12	7	5	0	113215.6700	0.1334	0.010	0.0019	0.38	Melli+22
202:	13	7	7	0	12	7	6	0	113215.6700	0.1334	0.010	0.0019	0.38	Melli+22
203:	13	7	6	1	12	7	5	1	113215.6700	-0.3931	0.010	0.0019	0.12	Melli+22
204:	13	7	7	1	12	7	6	1	113215.6700	-0.3931	0.010	0.0019	0.12	Melli+22
205:	13	1	12	0	12	1	11	0	113222.2040	0.0236	0.010			Melli+22
206:	13	1	12	1	12	1	11	1	113229.3800	0.0167	0.010			Melli+22
207:	13	8	5	0	12	8	4	0	113430.0440	0.0787	0.010	-0.0138	0.13	Melli+22
208:	13	8	6	0	12	8	5	0	113430.0440	0.0787	0.010	-0.0138	0.13	Melli+22
209:	13	8	5	1	12	8	4	1	113430.0440	-0.0447	0.010	-0.0138	0.37	Melli+22
210:	13	8	6	1	12	8	5	1	113430.0440	-0.0447	0.010	-0.0138	0.37	Melli+22
211:									0.0000	0.0534	0.100			Melli+22
212:	29	1	29	1	28	1	28	1	248655.5630	-0.0363	0.020			Melli+22
213:	29	1	29	0	28	1	28	0	248674.2010	0.0123	0.020			Melli+22
214:	29	0	29	1	28	0	28	1	250219.7360	-0.0018	0.020			Melli+22
215:	29	0	29	0	28	0	28	0	250225.6220	-0.0251	0.020			Melli+22
216:	29	2	28	1	28	2	27	1	250679.6900	-0.0185	0.020			Melli+22
217:	29	2	28	0	28	2	27	0	250682.1920	0.0203	0.020			Melli+22
218:	29	3	27	1	28	3	26	1	250990.0930	0.0727	0.040			Melli+22
219:	29	3	27	0	28	3	26	0	250991.0870	-0.0354	0.020			Melli+22
220:	29	3	26	1	28	3	25	1	250995.8000	0.0180	0.060			Melli+22
221:	29	3	26	0	28	3	25	0	250996.5780	-0.0144	0.020			Melli+22
222:	29	2	27	0	28	2	26	0	251008.9320	0.0399	0.020			Melli+22
223:	29	2	27	1	28	2	26	1	251014.2010	-0.0219	0.020			Melli+22
224:	29	4	25	1	28	4	24	1	251261.4210	-0.0239	0.020	-0.0033	0.50	Melli+22
225:	29	4	26	1	28	4	25	1	251261.4210	0.0173	0.020	-0.0033	0.50	Melli+22
226:	29	4	25	0	28	4	24	0	251262.4310	0.0067	0.040	0.0240	0.50	Melli+22
227:	29	4	26	0	28	4	25	0	251262.4310	0.0413	0.040	0.0240	0.50	Melli+22
228:	29	6	24	1	28	6	23	1	252000.2120	-0.4493	0.020	-0.0284	0.38	Melli+22
229:	29	6	23	0	28	6	22	0	252000.2120	1.2346	0.020	-0.0284	0.12	Melli+22
230:	29	6	24	0	28	6	23	0	252000.2120	1.2346	0.020	-0.0284	0.12	Melli+22
231:	29	6	23	1	28	6	22	1	252000.2120	-0.4493	0.020	-0.0284	0.38	Melli+22
232:	29	1	28	0	28	1	27	0	252363.2500	-0.0058	0.020			Melli+22
233:	29	1	28	1	28	1	27	1	252379.2170	-0.0164	0.020			Melli+22
234/	29	7	22	1	28	7	21	1	252440.7000	-0.5542	0.020	0.0767	0.12	Melli+22
235:	29	7	23	1	28	7	22	1	252440.7000	-0.5542	0.020	0.0767	0.12	Melli+22
236:	29	7	22	0	28	7	21	0	252440.7000	0.2868	0.020	0.0767	0.38	Melli+22
237:	29	7	23	0	28	7	22	0	252440.7000	0.2868	0.020	0.0767	0.38	Melli+22
238:									0.0000	0.3641	0.150			Melli+22
239:	29	8	21	0	28	8	20	0	252915.6340	0.2030	0.020	-0.0353	0.13	Melli+22
240:	29	8	22	0	28	8	21	0	252915.6340	0.2030	0.020	-0.0353	0.13	Melli+22
241:	29	8	21	1	28	8	20	1	252915.6340	-0.1150	0.020	-0.0353	0.37	Melli+22
242:	29	8	22	1	28	8	21	1	252915.6340	-0.1150	0.020	-0.0353	0.37	Melli+22
243:									0.0000	0.1378	0.100			Melli+22
244:	30	1	30	1	29	1	29	1	257214.7460	-0.0093	0.020			Melli+22
245:	30	1	30	0	29	1	29	0	257234.1500	-0.0196	0.020			Melli+22
246:	30	0	30	1	29	0	29	1	258813.4390	0.0114	0.020			Melli+22
247:	30	0	30	0	29	0	29	0	258820.0260	0.0128	0.020			Melli+22
248:	30	2	29	1	29	2	28	1	259308.8000	-0.0020	0.020			Melli+22
249:	30	2	29	0	29	2	28	0	259311.4720	0.0310	0.020			Melli+22
250:	30	3	28	1	29	3	27	1	259636.4380	0.0794	0.060			Melli+22
251:	30	3	28	0	29	3	27	0	259637.4130	-0.0230	0.020			Melli+22
252:	30	3	27	1	29	3	26	1	259643.1830	0.0130	0.060			Melli+22
253:	30	3	27	0	29	3	26	0	259643.8930	-0.0086	0.020			Melli+22
254:	30	2	28	0	29	2	27	0	259672.2010	0.0290	0.020			Melli+22
255:	30	2	28	1	29	2	27	1	259678.1520	-0.0159	0.020			Melli+22
256:	30	4	26	1	29	4	25	1	259916.1630	-0.0314	0.020	-0.0053	0.50	Melli+22
257:	30	4	27	1	29	4	26	1	259916.1630	0.0208	0.020	-0.0053	0.50	Melli+22
258:	30	4	26	0	29	4	25	0	259917.1860	0.0115	0.040	0.0334	0.50	Melli+22
259:	30	4	27	0	29	4	26	0	259917.1860	0.0553	0.040	0.0334	0.50	Melli+22
260:	30	6	24	1	29	6	23	1	260679.4930	-0.4282	0.020	-0.0198	0.38	Melli+22
261:	30	6	25	1	29	6	24	1	260679.4930	-0.4282	0.020	-0.0198	0.38	Melli+22
262:	30	6	24	0	29	6	23	0	260679.4930	1.2052	0.020	-0.0198	0.12	Melli+22
263:	30	6	25	0	29	6	24	0	260679.4930	1.2052	0.020	-0.0198	0.12	Melli+22
264:									0.0000	0.7073	0.250			Melli+22
265:	30	1	29	0	29	1	28	0	261046.3930	-0.0182	0.020			Melli+22
266:	30	1	29	1	29	1	28	1	261062.9190	-0.0079	0.020			Melli+22
267:	30	7	23	1	29	7	22	1	261134.8890	-0.4700	0.020	0.0023	0.12	Melli+22
268:	30	7	24	1	29	7	23	1	261134.8890	-0.4700	0.020	0.0023	0.12	Melli+22
269:	30	7	23	0	29	7	22	0	261134.8890	0.1596	0.020	0.0023	0.38	Melli+22

270:	30	7	24	0	29	7	23	0	261134.8890	0.1596	0.020	0.0023	0.38	Melli+22
271:									0.0000	0.2725	0.250			Melli+22
272:	30	8	22	0	29	8	21	0	261625.9110	-0.0112	0.020	0.0358	0.13	Melli+22
273:	30	8	23	0	29	8	22	0	261625.9110	-0.0112	0.020	0.0358	0.13	Melli+22
274:	30	8	22	1	29	8	21	1	261625.9110	0.0515	0.020	0.0358	0.37	Melli+22
275:	30	8	23	1	29	8	22	1	261625.9110	0.0515	0.020	0.0358	0.37	Melli+22
276:									0.0000	0.0271	0.100			Melli+22
277:	31	1	31	1	30	1	30	1	265772.4090	-0.0117	0.020			Melli+22
278:	31	1	31	0	30	1	30	0	265792.6620	-0.0168	0.020			Melli+22
279:	31	0	31	1	30	0	30	1	267403.7790	0.0096	0.020			Melli+22
280:	31	0	31	0	30	0	30	0	267411.0760	-0.0029	0.020			Melli+22
281:	31	2	30	1	30	2	29	1	267936.3810	-0.0042	0.020			Melli+22
282:	31	2	30	1	30	2	29	1	267936.3830	-0.0022	0.020			Melli+22
283:	31	2	30	0	30	2	29	0	267939.2300	0.0203	0.020			Melli+22
284:	31	2	30	0	30	2	29	0	267939.2320	0.0223	0.020			Melli+22
285:	31	3	29	1	30	3	28	1	268281.8590	0.0608	0.060			Melli+22
286:	31	3	29	0	30	3	28	0	268282.8290	-0.0166	0.020			Melli+22
287:	31	3	29	0	30	3	28	0	268282.8320	-0.0136	0.020			Melli+22
288:	31	3	28	0	30	3	27	0	268290.4320	-0.0128	0.020			Melli+22
289:	31	3	28	0	30	3	27	0	268290.4330	-0.0118	0.020			Melli+22
290/	31	2	29	0	30	2	28	0	268336.1600	0.0616	0.020			Melli+22
291/	31	2	29	0	30	2	28	0	268336.1630	0.0646	0.020			Melli+22
292:	31	2	29	1	30	2	28	1	268342.7890	-0.0186	0.020			Melli+22
293:	31	2	29	1	30	2	28	1	268342.7900	-0.0176	0.020			Melli+22
294:	31	4	28	1	30	4	27	1	268569.9480	0.0308	0.020	-0.0019	0.50	Melli+22
295:	31	4	27	1	30	4	26	1	268569.9480	-0.0347	0.020	-0.0019	0.50	Melli+22
296:	31	4	28	1	30	4	27	1	268569.9500	0.0328	0.020	0.0001	0.50	Melli+22
297:	31	4	27	1	30	4	26	1	268569.9500	-0.0327	0.020	0.0001	0.50	Melli+22
298:	31	4	28	0	30	4	27	0	268570.9600	0.0536	0.060	0.0262	0.50	Melli+22
299:	31	4	27	0	30	4	26	0	268570.9600	-0.0013	0.060	0.0262	0.50	Melli+22
300:	31	4	28	0	30	4	27	0	268570.9640	0.0576	0.040	0.0302	0.50	Melli+22
301:	31	4	27	0	30	4	26	0	268570.9640	0.0026	0.040	0.0302	0.50	Melli+22
302:	31	6	26	1	30	6	25	1	269357.7190	-0.3976	0.020	-0.0083	0.37	Melli+22
303:	31	6	25	1	30	6	24	1	269357.7190	-0.3976	0.020	-0.0083	0.37	Melli+22
304:	31	6	25	0	30	6	24	0	269357.7190	1.1584	0.020	-0.0083	0.13	Melli+22
305:	31	6	26	0	30	6	25	0	269357.7190	1.1584	0.020	-0.0083	0.13	Melli+22
306:									0.0000	0.6740	0.250			Melli+22
307:	31	1	30	0	30	1	29	0	269727.6440	0.0143	0.020			Melli+22
308:	31	1	30	1	30	1	29	1	269744.6780	-0.0026	0.020			Melli+22
309/	31	7	24	1	30	7	23	1	269828.0030	-0.3534	0.020	-0.0835	0.12	Melli+22
310:	31	7	25	1	30	7	24	1	269828.0030	-0.3534	0.020	-0.0835	0.12	Melli+22
311:	31	7	24	0	30	7	23	0	269828.0030	0.0064	0.020	-0.0835	0.38	Melli+22
312:	31	7	25	0	30	7	24	0	269828.0030	0.0064	0.020	-0.0835	0.38	Melli+22
313:									0.0000	0.1557	0.250			Melli+22
314:	32	1	32	1	31	1	31	1	274328.5130	-0.0412	0.020			Melli+22
315:	32	1	32	0	31	1	31	0	274349.6690	-0.0064	0.020			Melli+22
316:	32	0	32	1	31	0	31	1	275990.6980	0.0139	0.020			Melli+22
317:	32	0	32	0	31	0	31	0	275998.7880	0.0215	0.020			Melli+22
318:	32	2	31	1	31	2	30	1	276562.4290	0.0179	0.020			Melli+22
319:	32	2	31	0	31	2	30	0	276565.4680	0.0372	0.020			Melli+22
320:	32	3	30	1	31	3	29	1	276926.3630	0.0592	0.060			Melli+22
321:	32	3	30	0	31	3	29	0	276927.2960	-0.0199	0.020	-0.0200	0.50	Melli+22
322:	32	3	30	0	31	3	29	0	276927.2960	-0.0199	0.020	-0.0200	0.50	Melli+22
323:	32	3	29	0	31	3	28	0	276936.1810	-0.0187	0.020			Melli+22
324:	32	3	29	0	31	3	28	0	276936.1830	-0.0167	0.020			Melli+22
325:	32	2	30	0	31	2	29	0	277000.6810	0.0171	0.020			Melli+22
326:	32	2	30	0	31	2	29	0	277000.6820	0.0181	0.020			Melli+22
327:	32	2	30	1	31	2	29	1	277008.1140	-0.0221	0.020			Melli+22
328:	32	2	30	1	31	2	29	1	277008.1150	-0.0211	0.020			Melli+22
329:	32	4	28	1	31	4	27	1	277222.7400	-0.0376	0.020	0.0033	0.50	Melli+22
330:	32	4	29	1	31	4	28	1	277222.7400	0.0441	0.020	0.0033	0.50	Melli+22
331:	32	4	29	1	31	4	28	1	277222.7410	0.0451	0.020	0.0043	0.50	Melli+22
332:	32	4	28	1	31	4	27	1	277222.7410	-0.0366	0.020	0.0043	0.50	Melli+22
333:	32	4	29	0	31	4	28	0	277223.7350	0.0507	0.020	0.0165	0.50	Melli+22
334:	32	4	28	0	31	4	27	0	277223.7350	-0.0177	0.020	0.0165	0.50	Melli+22
335:	32	4	28	0	31	4	27	0	277223.7420	-0.0107	0.040	0.0235	0.50	Melli+22
336:	32	4	29	0	31	4	28	0	277223.7420	0.0577	0.040	0.0235	0.50	Melli+22
337:	32	6	26	1	31	6	25	1	278034.8530	-0.3587	0.020	0.0038	0.19	Melli+22
338:	32	6	27	1	31	6	26	1	278034.8530	-0.3587	0.020	0.0038	0.19	Melli+22

339:	32	6	26	0	31	6	25	0	278034.8530	1.0901	0.020	0.0038	0.06	Melli+22
340:	32	6	27	0	31	6	26	0	278034.8530	1.0901	0.020	0.0038	0.06	Melli+22
341:	32	6	26	1	31	6	25	1	278034.8530	-0.3587	0.020	0.0038	0.19	Melli+22
342:	32	6	27	1	31	6	26	1	278034.8530	-0.3587	0.020	0.0038	0.19	Melli+22
343:	32	6	26	0	31	6	25	0	278034.8530	1.0901	0.020	0.0038	0.06	Melli+22
344:	32	6	27	0	31	6	26	0	278034.8530	1.0901	0.020	0.0038	0.06	Melli+22
345:									0.0000	0.6275	0.250			Melli+22
346:	32	1	31	0	31	1	30	0	278406.8530	0.0066	0.020			Melli+22
347:	32	1	31	1	31	1	30	1	278424.4190	-0.0104	0.020			Melli+22
348:	33	1	33	1	32	1	32	1	282883.1300	0.0146	0.020			Melli+22
349:	33	1	33	0	32	1	32	0	282905.0760	-0.0436	0.020			Melli+22
350:	33	0	33	1	32	0	32	1	284574.1180	0.0211	0.020			Melli+22
351:	33	0	33	0	32	0	32	0	284583.0150	0.0128	0.020			Melli+22
352:	33	2	32	1	32	2	31	1	285186.8330	0.0005	0.020			Melli+22
353:	33	2	32	0	32	2	31	0	285190.0700	0.0120	0.020			Melli+22
354:	33	3	31	1	32	3	30	1	285569.9090	0.0696	0.060			Melli+22
355:	33	3	31	0	32	3	30	0	285570.8010	-0.0099	0.020	-0.0100	0.50	Melli+22
356:	33	3	31	0	32	3	30	0	285570.8010	-0.0099	0.020	-0.0100	0.50	Melli+22
357:	33	3	30	0	32	3	29	0	285581.0900	-0.0545	0.040	0.0490	0.75	Melli+22
358:	33	3	30	1	32	3	29	1	285581.0900	0.3597	0.040	0.0490	0.25	Melli+22
359:	33	3	30	0	32	3	29	0	285581.0910	-0.0535	0.020			Melli+22
360:	33	2	31	0	32	2	30	0	285665.8800	0.0224	0.020	0.0224	0.50	Melli+22
361:	33	2	31	0	32	2	30	0	285665.8800	0.0224	0.020	0.0224	0.50	Melli+22
362:	33	2	31	1	32	2	30	1	285674.1290	-0.0147	0.020			Melli+22
363:	33	2	31	1	32	2	30	1	285674.1300	-0.0137	0.020			Melli+22
364:	33	4	29	1	32	4	28	1	285874.5000	-0.0469	0.020	0.0037	0.50	Melli+22
365:	33	4	30	1	32	4	29	1	285874.5000	0.0542	0.020	0.0037	0.50	Melli+22
366:	33	4	29	0	32	4	28	0	285875.4760	-0.0405	0.060	0.0018	0.50	Melli+22
367:	33	4	30	0	32	4	29	0	285875.4760	0.0441	0.060	0.0018	0.50	Melli+22
368:	33	1	32	0	32	1	31	0	287084.0000	0.0035	0.020			Melli+22
369:	33	1	32	1	32	1	31	1	287102.0960	-0.0121	0.020			Melli+22
370:	34	1	34	1	33	1	33	1	291436.0580	-0.0062	0.020			Melli+22
371:	34	1	34	0	33	1	33	0	291458.9560	-0.0160	0.020			Melli+22
372:	34	0	34	1	33	0	33	1	293153.9310	-0.0060	0.020			Melli+22
373:	34	0	34	0	33	0	33	0	293163.7220	0.0056	0.020			Melli+22
374:	34	2	33	1	33	2	32	1	293809.6000	-0.0030	0.020			Melli+22
375:	34	2	33	0	33	2	32	0	293813.0590	0.0142	0.020			Melli+22
376:	34	3	32	0	33	3	31	0	294213.2850	-0.0092	0.020			Melli+22
377:	34	3	31	0	33	3	30	0	294225.2250	-0.0326	0.020			Melli+22
378:	34	2	32	1	33	2	31	1	294340.8500	0.0332	0.020			Melli+22
379:	34	1	33	0	33	1	32	0	295759.0240	0.0086	0.020			Melli+22
380:	34	1	33	1	33	1	32	1	295777.6340	-0.0177	0.020			Melli+22
381:	35	1	35	1	34	1	34	1	299987.3580	-0.0041	0.020			Melli+22
382:	35	1	35	0	34	1	34	0	300011.1710	-0.0231	0.020			Melli+22
383:	35	0	35	1	34	0	34	1	301730.1510	0.0126	0.020			Melli+22
384:	35	0	35	0	34	0	34	0	301740.8630	0.0193	0.020			Melli+22
385:	35	2	34	1	34	2	33	1	302430.6750	-0.0014	0.020			Melli+22
386:	35	2	34	0	34	2	33	0	302434.3320	-0.0133	0.020			Melli+22
387:	35	3	33	0	34	3	32	0	302854.7140	-0.0150	0.020			Melli+22
388:	35	3	32	0	34	3	31	0	302868.5060	-0.0116	0.020	0.0198	0.75	Melli+22
389:	35	3	32	1	34	3	31	1	302868.5060	0.1141	0.020	0.0198	0.25	Melli+22
390:	35	2	33	0	34	2	32	0	302998.0910	0.0249	0.020			Melli+22
391:	35	2	33	1	34	2	32	1	303008.1270	-0.0103	0.020			Melli+22
392:	35	4	31	1	34	4	30	1	303174.8130	-0.0674	0.020	0.0084	0.50	Melli+22
393:	35	4	32	1	34	4	31	1	303174.8130	0.0843	0.020	0.0084	0.50	Melli+22
394:	35	4	31	0	34	4	30	0	303175.7330	-0.1000	0.040	-0.0365	0.50	Melli+22
395:	35	4	32	0	34	4	31	0	303175.7330	0.0270	0.040	-0.0365	0.50	Melli+22
396/	35	6	29	1	34	6	28	1	304059.3650	-0.1679	0.020	0.0607	0.37	Melli+22
397:	35	6	30	1	34	6	29	1	304059.3650	-0.1679	0.020	0.0607	0.37	Melli+22
398:	35	6	29	0	34	6	28	0	304059.3650	0.7458	0.020	0.0607	0.13	Melli+22
399:	35	6	30	0	34	6	29	0	304059.3650	0.7458	0.020	0.0607	0.13	Melli+22
400:									0.0000	0.3957	0.250			Melli+22
401:	35	1	34	0	34	1	33	0	304431.8490	0.0105	0.020			Melli+22
402:	35	1	34	1	34	1	33	1	304450.9790	-0.0161	0.020			Melli+22
403:	20	1	20	0	19	0	19	1	305551.1890	0.0024	0.030			Melli+22
404:	36	0	36	1	35	0	35	1	310302.6550	0.0156	0.020			Melli+22
405:	36	0	36	0	35	0	35	0	310314.3350	0.0117	0.020			Melli+22
406:	36	2	35	1	35	2	34	1	311050.0010	-0.0058	0.020			Melli+22
407:	36	2	35	0	35	2	34	0	311053.9230	0.0088	0.020			Melli+22

408:	36	3	34	0	35	3	33	0	311495.0670	-0.0111	0.020			Melli+22
409:	36	3	33	1	35	3	32	1	311510.9250	-0.0231	0.020	0.0104	0.25	Melli+22
410:	36	3	33	0	35	3	32	0	311510.9250	0.0215	0.020	0.0104	0.75	Melli+22
411:	41	1	41	1	40	1	40	1	351258.3980	0.0065	0.020			Melli+22
412:	41	1	41	0	40	1	40	0	351288.1920	-0.0350	0.020			Melli+22
413:	41	0	41	1	40	0	40	1	353107.8420	0.0092	0.020			Melli+22
414:	41	0	41	0	40	0	40	0	353125.1440	-0.0751	0.060			Melli+22
415:	41	2	40	1	40	2	39	1	354118.9280	-0.0055	0.020			Melli+22
416:	41	2	40	0	40	2	39	0	354124.2170	0.0069	0.020			Melli+22
417:	41	2	39	0	40	2	38	0	355007.3580	0.0080	0.020			Melli+22
418:	41	2	39	1	40	2	38	1	355024.1220	0.0163	0.020			Melli+22
419:	26	1	26	1	25	0	25	0	356227.4430	-0.0146	0.020			Melli+22
420:	27	1	27	0	26	0	26	1	356402.5310	-0.0123	0.020			Melli+22
421:	41	1	40	0	40	1	39	0	356419.0350	-0.0137	0.020			Melli+22
422:	41	1	40	1	40	1	39	1	356441.2180	0.0072	0.020			Melli+22
423:	42	1	42	1	41	1	41	1	359797.1150	0.0088	0.020			Melli+22
424:	42	1	42	0	41	1	41	0	359827.9670	-0.0539	0.020			Melli+22
425:	42	0	42	1	41	0	41	1	361657.1420	0.0206	0.020			Melli+22
426:	42	0	42	0	41	0	41	0	361675.8210	0.0099	0.020			Melli+22
427:	42	2	41	1	41	2	40	1	362726.8620	-0.0012	0.020			Melli+22
428:	42	2	41	0	41	2	40	0	362732.4630	0.0125	0.020			Melli+22
429:	27	1	27	1	26	0	26	0	363336.8550	-0.0074	0.030			Melli+22
430:	42	3	39	0	41	3	38	0	363345.7240	0.0080	0.020			Melli+22
431/	42	3	39	1	41	3	38	1	363347.2900	0.0609	0.020			Melli+22
432:	28	1	28	0	27	0	27	1	363492.6640	-0.0073	0.020			Melli+22
433:	42	2	40	0	41	2	39	0	363677.0170	0.0065	0.020			Melli+22
434:	42	4	38	1	41	4	37	1	363688.9330	-0.0995	0.030	-0.0503	0.38	Melli+22
435:	42	4	39	0	41	4	38	0	363688.9330	-0.4756	0.030	-0.0503	0.12	Melli+22
436:	42	4	38	0	41	4	37	0	363688.9330	-0.9190	0.030	-0.0503	0.12	Melli+22
437:	42	4	39	1	41	4	38	1	363688.9330	0.4304	0.030	-0.0503	0.38	Melli+22
438:	42	2	40	1	41	2	39	1	363695.0860	0.0043	0.020			Melli+22
439:	42	1	41	0	41	1	40	0	365074.6840	-0.0113	0.020			Melli+22
440:	43	1	43	1	42	1	42	1	368333.9050	0.0184	0.020			Melli+22
441:	43	1	43	0	42	1	42	0	368365.8610	-0.0421	0.020			Melli+22
442:	43	0	43	1	42	0	42	1	370202.4200	-0.0026	0.020			Melli+22
443/	43	0	43	0	42	0	42	0	370222.5390	0.0694	0.020			Melli+22
444:	29	1	29	0	28	0	28	1	370544.0460	-0.0310	0.030			Melli+22
445:	43	2	42	1	42	2	41	1	371332.7310	-0.0083	0.020			Melli+22
446:	43	2	42	0	42	2	41	0	371338.6550	0.0051	0.020			Melli+22
447:	43	3	41	0	42	3	40	0	371943.8560	-0.0372	0.020	0.0260	0.75	Melli+22
448:	43	3	41	1	42	3	40	1	371943.8560	0.2156	0.020	0.0260	0.25	Melli+22
449:	43	3	40	0	42	3	39	0	371981.3970	-0.0181	0.020			Melli+22
450:	43	3	40	1	42	3	39	1	371983.2880	0.0268	0.030			Melli+22
451:	43	4	39	1	42	4	38	1	372328.6420	-0.1438	0.040	-0.0426	0.38	Melli+22
452:	43	4	40	0	42	4	39	0	372328.6420	-0.4119	0.040	-0.0426	0.12	Melli+22
453:	43	4	40	1	42	4	39	1	372328.6420	0.4783	0.040	-0.0426	0.38	Melli+22
454:	43	4	39	0	42	4	38	0	372328.6420	-0.9323	0.040	-0.0426	0.12	Melli+22
455:	43	2	41	0	42	2	40	0	372346.9440	-0.0063	0.020			Melli+22
456:	43	2	41	1	42	2	40	1	372366.4130	0.0170	0.020			Melli+22
457:	43	1	42	0	42	1	41	0	373727.6260	-0.0053	0.020			Melli+22
458:	43	1	42	1	42	1	41	1	373750.7440	0.0059	0.020			Melli+22
459:	44	1	44	1	43	1	43	1	376868.7410	0.0403	0.020			Melli+22
460:	44	1	44	0	43	1	43	0	376901.8240	-0.0179	0.020			Melli+22
461:	44	0	44	1	43	0	43	1	378743.6990	-0.0187	0.020			Melli+22
462:	44	0	44	0	43	0	43	0	378765.2010	0.0259	0.020			Melli+22
463:	44	2	43	1	43	2	42	1	379936.5220	0.0027	0.020			Melli+22
464:	44	2	43	0	43	2	42	0	379942.7860	0.0203	0.020			Melli+22
465:	44	3	42	1	43	3	41	1	380574.1550	0.1413	0.020	0.0314	0.25	Melli+22
466:	44	3	42	0	43	3	41	0	380574.1550	-0.0053	0.020	0.0314	0.75	Melli+22
467:	44	3	41	0	43	3	40	0	380616.0890	0.0081	0.020			Melli+22
468:	44	3	41	1	43	3	40	1	380618.3070	0.0176	0.030			Melli+22
469:	44	4	40	1	43	4	39	1	380966.9450	-0.2176	0.040	-0.0571	0.38	Melli+22
470:	44	4	41	0	43	4	40	0	380966.9450	-0.3625	0.040	-0.0571	0.12	Melli+22
471:	44	4	40	0	43	4	39	0	380966.9450	-0.9710	0.040	-0.0571	0.12	Melli+22
472:	44	4	41	1	43	4	40	1	380966.9450	0.5099	0.040	-0.0571	0.38	Melli+22
473:	44	2	42	0	43	2	41	0	381017.1290	0.0206	0.020			Melli+22
474:	44	2	42	1	43	2	41	1	381037.9860	-0.0012	0.020			Melli+22
475:	44	1	43	0	43	1	42	0	382377.7690	-0.0242	0.020			Melli+22
476:	44	1	43	1	43	1	42	1	382401.3770	0.0180	0.020			Melli+22

477:	45	1	45	1	44	1	44	1	385401.5440	0.0263	0.020			Melli+22
478:	45	1	45	0	44	1	44	0	385435.7980	-0.0080	0.020			Melli+22
479:	45	0	45	1	44	0	44	1	387280.9670	-0.0260	0.020			Melli+22
480:	45	0	45	0	44	0	44	0	387303.9300	0.0167	0.020			Melli+22
481:	45	2	44	1	44	2	43	1	388538.1570	-0.0035	0.020			Melli+22
482:	45	2	44	0	44	2	43	0	388544.7810	0.0252	0.020			Melli+22
483:	45	3	43	1	44	3	42	1	389202.9900	0.0371	0.020	0.0121	0.25	Melli+22
484:	45	3	43	0	44	3	42	0	389202.9900	0.0037	0.020	0.0121	0.75	Melli+22
485:	45	3	42	0	44	3	41	0	389249.6810	-0.0133	0.020			Melli+22
486:	45	3	42	1	44	3	41	1	389252.2870	-0.0098	0.020			Melli+22
487:	45	2	43	0	44	2	42	0	389687.4180	0.0004	0.020			Melli+22
488:	45	2	43	1	44	2	42	1	389709.7660	-0.0229	0.020			Melli+22
489:	45	1	44	0	44	1	43	0	391025.0810	-0.0361	0.020			Melli+22
490:	45	1	44	1	44	1	43	1	391049.1650	0.0327	0.020			Melli+22
491:	46	1	46	1	45	1	45	1	393932.3220	0.0146	0.020			Melli+22
492:	46	1	46	0	45	1	45	0	393967.7650	-0.0000	0.020			Melli+22
493:	46	0	46	1	45	0	45	1	395814.1830	-0.0579	0.020			Melli+22
494:	46	0	46	0	45	0	45	0	395838.7010	0.0257	0.020			Melli+22
495:	46	2	45	1	45	2	44	1	397137.5890	-0.0325	0.020			Melli+22
496:	46	2	45	0	45	2	44	0	397144.5850	0.0067	0.020			Melli+22
497:	46	3	44	1	45	3	43	1	397830.3290	-0.0877	0.020	-0.0223	0.25	Melli+22
498:	46	3	44	0	45	3	43	0	397830.3290	-0.0005	0.020	-0.0223	0.75	Melli+22
499:	46	3	43	0	45	3	42	0	397882.2350	-0.0016	0.020			Melli+22
500:	46	3	43	1	45	3	42	1	397885.2380	-0.0288	0.020			Melli+22
501:	46	2	44	0	45	2	43	0	398357.8180	0.0114	0.020			Melli+22
502:	46	2	44	1	45	2	43	1	398381.7080	-0.0214	0.020			Melli+22
503:	46	1	45	0	45	1	44	0	399669.4950	-0.0450	0.020			Melli+22
504:	46	1	45	1	45	1	44	1	399694.0310	0.0370	0.020			Melli+22
505:	47	1	47	1	46	1	46	1	402461.0640	0.0235	0.020			Melli+22
506:	47	1	47	0	46	1	46	0	402497.6960	0.0065	0.020			Melli+22
507:	47	0	47	1	46	0	46	1	404343.4030	-0.0562	0.020			Melli+22
508:	47	0	47	0	46	0	46	0	404369.4620	0.0042	0.020			Melli+22

(E)-cyanovinylacetylene   Wed Jul 13 14:41:09 2022

/ instead of : below denotes (o-c)>3*err

											obs	o-c	error	blends o-c	blends wt	Notes
1:	3	1	3	0	3	2	1	2	0	2	8626.1631	0.0001	0.002			Thorwirth+04
2:	3	1	3	0	2	2	1	2	0	1	8626.4961	-0.0004	0.002			Thorwirth+04
3:	3	1	3	0	4	2	1	2	0	3	8626.5127	-0.0000	0.002			Thorwirth+04
4:	3	0	3	0	2	2	0	2	0	1	8694.8604	-0.0000	0.002			Thorwirth+04
5:	3	0	3	0	3	2	0	2	0	2	8695.0562	0.0008	0.002			Thorwirth+04
6:	3	0	3	0	4	2	0	2	0	3	8695.1026	0.0007	0.002			Thorwirth+04
7:	3	1	2	0	3	2	1	1	0	2	8763.8760	-0.0004	0.002			Thorwirth+04
8:	3	1	2	0	4	2	1	1	0	3	8764.2217	-0.0010	0.002	-0.0017	0.68	Thorwirth+04
9:	3	1	2	0	2	2	1	1	0	1	8764.2217	-0.0029	0.002	-0.0017	0.32	Thorwirth+04
10:	4	1	4	0	4	3	1	3	0	3	11501.7276	0.0008	0.002			Thorwirth+04
11:	4	1	4	0	3	3	1	3	0	2	11501.8291	0.0010	0.002			Thorwirth+04
12:	4	1	4	0	5	3	1	3	0	4	11501.8819	-0.0010	0.002			Thorwirth+04
13:	4	0	4	0	3	3	0	3	0	2	11593.1475	-0.0007	0.002			Thorwirth+04
14:	4	0	4	0	4	3	0	3	0	3	11593.2315	-0.0002	0.002			Thorwirth+04
15:	4	0	4	0	5	3	0	3	0	4	11593.2613	-0.0000	0.002			Thorwirth+04
16:	4	1	3	0	4	3	1	2	0	3	11685.3438	0.0007	0.002			Thorwirth+04
17:	4	1	3	0	3	3	1	2	0	2	11685.4522	0.0015	0.002			Thorwirth+04
18:	4	1	3	0	5	3	1	2	0	4	11685.4986	0.0015	0.002			Thorwirth+04
19:	5	1	5	0	5	4	1	4	0	4	14377.1480	-0.0013	0.002			Thorwirth+04
20:	5	1	5	0	4	4	1	4	0	3	14377.1900	0.0006	0.002			Thorwirth+04
21:	5	1	5	0	6	4	1	4	0	5	14377.2359	0.0009	0.002			Thorwirth+04
22:	5	0	5	0	4	4	0	4	0	3	14491.2120	0.0006	0.002			Thorwirth+04
23:	5	0	5	0	5	4	0	4	0	4	14491.2579	0.0003	0.002			Thorwirth+04
24:	5	0	5	0	6	4	0	4	0	5	14491.2774	-0.0007	0.002			Thorwirth+04
25:	5	1	4	0	5	4	1	3	0	4	14606.6680	0.0010	0.002			Thorwirth+04
26:	5	1	4	0	4	4	1	3	0	3	14606.7120	0.0015	0.002			Thorwirth+04
27:	5	1	4	0	6	4	1	3	0	5	14606.7539	0.0027	0.002			Thorwirth+04
28:	6	1	6	0	6	5	1	5	0	5	17252.4883	-0.0010	0.002			Thorwirth+04
29:	6	1	6	0	5	5	1	5	0	4	17252.5078	0.0005	0.002			Thorwirth+04
30:	6	1	6	0	7	5	1	5	0	6	17252.5420	-0.0004	0.002			Thorwirth+04
31:	6	0	6	0	5	5	0	5	0	4	17389.0655	-0.0006	0.002			Thorwirth+04
32:	6	0	6	0	6	5	0	5	0	5	17389.0938	-0.0017	0.002			Thorwirth+04
33:	6	0	6	0	7	5	0	5	0	6	17389.1114	0.0007	0.002			Thorwirth+04
34:	6	1	5	0	6	5	1	4	0	5	17527.9073	0.0010	0.002			Thorwirth+04
35:	6	1	5	0	5	5	1	4	0	4	17527.9278	0.0014	0.002			Thorwirth+04
36:	6	1	5	0	7	5	1	4	0	6	17527.9600	0.0017	0.002			Thorwirth+04
37:	7	1	7	0	7	6	1	6	0	6	20127.7520	-0.0013	0.002			Thorwirth+04
38:	7	1	7	0	6	6	1	6	0	5	20127.7637	0.0019	0.002			Thorwirth+04
39:	7	1	7	0	8	6	1	6	0	7	20127.7871	-0.0019	0.002			Thorwirth+04
40:	7	0	7	0	6	6	0	6	0	5	20286.6905	0.0029	0.002			Thorwirth+04
41:	7	0	7	0	7	6	0	6	0	6	20286.7071	-0.0007	0.002			Thorwirth+04
42:	7	0	7	0	8	6	0	6	0	7	20286.7227	0.0032	0.002			Thorwirth+04
43:	7	1	6	0	7	6	1	5	0	6	20449.0664	-0.0005	0.002			Thorwirth+04
44:	7	1	6	0	6	6	1	5	0	5	20449.0801	0.0032	0.002			Thorwirth+04
45:	7	1	6	0	8	6	1	5	0	7	20449.1026	0.0008	0.002			Thorwirth+04
46:	8	1	8	0	8	7	1	7	0	7	23002.9385	0.0020	0.002	0.0003	0.53	Thorwirth+04
47:	8	1	8	0	7	7	1	7	0	6	23002.9385	-0.0017	0.002	0.0003	0.47	Thorwirth+04
48:	8	1	8	0	9	7	1	7	0	8	23002.9629	0.0010	0.002			Thorwirth+04
49:	8	0	8	0	7	7	0	7	0	6	23184.0410	-0.0013	0.002			Thorwirth+04
50:	8	0	8	0	8	7	0	7	0	7	23184.0566	-0.0004	0.002			Thorwirth+04
51:	8	0	8	0	9	7	0	7	0	8	23184.0684	0.0020	0.002			Thorwirth+04
52:	8	1	7	0	8	7	1	6	0	7	23370.1465	0.0031	0.003	0.0008	0.53	Thorwirth+04
53:	8	1	7	0	7	7	1	6	0	6	23370.1465	-0.0018	0.003	0.0008	0.47	Thorwirth+04
54:	8	1	7	0	9	7	1	6	0	8	23370.1700	0.0019	0.002			Thorwirth+04
55:	10	0	10	0	9	9	0	9	0	8	28977.8037	-0.0038	0.004			Thorwirth+04
56:	10	0	10	0	10	9	0	9	0	9	28977.8174	0.0010	0.004			Thorwirth+04
57:	10	0	10	0	11	9	0	9	0	10	28977.8262	0.0035	0.004			Thorwirth+04
58:	12	0	12	0	11	11	0	11	0	10	34770.0684	0.0002	0.004			Thorwirth+04
59:	12	0	12	0	12	11	0	11	0	11	34770.0840	0.0101	0.004	0.0077	0.48	Thorwirth+04
60:	12	0	12	0	13	11	0	11	0	12	34770.0840	0.0054	0.004	0.0077	0.52	Thorwirth+04
61:	13	0	13	1	13	12	0	12	1	12	37665.5488	0.0022	0.004			Thorwirth+04
62:	10	1	10	1	10	9	1	9	1	9	28753.0900	0.0593	0.060			August-88

63:	10	0	10	1	10	9	0	9	1	9	28977.8700	0.0535	0.060			August-88
64:	10	2	9	1	10	9	2	8	1	9	28984.0500	0.1216	0.060			August-88
65:	10	3	8	1	10	9	3	7	1	9	28987.2100	0.1241	0.060	0.1175	0.50	August-88
66:	10	3	7	1	10	9	3	6	1	9	28987.2100	0.1107	0.060	0.1175	0.50	August-88
67:	10	4	6	1	10	9	4	5	1	9	28988.8800	0.0598	0.060	0.0599	0.50	August-88
68:	10	4	7	1	10	9	4	6	1	9	28988.8800	0.0598	0.060	0.0599	0.50	August-88
69:	10	2	8	1	10	9	2	7	1	9	28989.7700	0.0233	0.060			August-88
70:	10	5	5	1	10	9	5	4	1	9	28991.3700	0.0357	0.060	0.0357	0.50	August-88
71:	10	5	6	1	10	9	5	5	1	9	28991.3700	0.0357	0.060	0.0357	0.50	August-88
72:	10	6	4	1	10	9	6	3	1	9	28994.5700	0.0617	0.060	0.0617	0.50	August-88
73:	10	6	5	1	10	9	6	4	1	9	28994.5700	0.0617	0.060	0.0617	0.50	August-88
74:	10	7	3	1	10	9	7	2	1	9	28998.3900	0.0872	0.060	0.0873	0.50	August-88
75:	10	7	4	1	10	9	7	3	1	9	28998.3900	0.0872	0.060	0.0873	0.50	August-88
76:	10	1	9	1	10	9	1	8	1	9	29212.1100	0.0991	0.060			August-88
77:	11	1	11	1	11	10	1	10	1	10	31628.0100	0.0952	0.060			August-88
78:	11	0	11	1	11	10	0	10	1	10	31874.2000	0.0483	0.060			August-88
79:	11	2	10	1	11	10	2	9	1	10	31882.1200	0.1145	0.060			August-88
80:	11	3	9	1	11	10	3	8	1	10	31885.9600	0.0890	0.060	0.0781	0.50	August-88
81:	11	3	8	1	11	10	3	7	1	10	31885.9600	0.0672	0.060	0.0781	0.50	August-88
82:	11	4	7	1	11	10	4	6	1	10	31887.7700	0.0670	0.060	0.0670	0.50	August-88
83:	11	4	8	1	11	10	4	7	1	10	31887.7700	0.0670	0.060	0.0670	0.50	August-88
84:	11	2	9	1	11	10	2	8	1	10	31889.8500	0.0877	0.060			August-88
85:	11	5	6	1	11	10	5	5	1	10	31890.4600	0.0254	0.060	0.0254	0.50	August-88
86:	11	5	7	1	11	10	5	6	1	10	31890.4600	0.0254	0.060	0.0254	0.50	August-88
87:	11	6	5	1	11	10	6	4	1	10	31893.9600	0.0518	0.060	0.0519	0.50	August-88
88:	11	6	6	1	11	10	6	5	1	10	31893.9600	0.0518	0.060	0.0519	0.50	August-88
89:	11	7	4	1	11	10	7	3	1	10	31898.1300	0.0586	0.060	0.0586	0.50	August-88
90:	11	7	5	1	11	10	7	4	1	10	31898.1300	0.0586	0.060	0.0586	0.50	August-88
91:	11	1	10	1	11	10	1	9	1	10	32132.8100	0.0370	0.060			August-88
92:	12	1	12	1	12	11	1	11	1	11	34502.7500	0.0699	0.060			August-88
93:	12	0	12	1	12	11	0	11	1	11	34770.1200	0.0459	0.060			August-88
94:	12	2	11	1	12	11	2	10	1	11	34780.0500	0.0576	0.060			August-88
95:	12	3	10	1	12	11	3	9	1	11	34784.7500	0.0728	0.060	0.0559	0.50	August-88
96:	12	3	9	1	12	11	3	8	1	11	34784.7500	0.0389	0.060	0.0559	0.50	August-88
97:	12	4	8	1	12	11	4	7	1	11	34786.6200	0.0339	0.060	0.0339	0.50	August-88
98:	12	4	9	1	12	11	4	8	1	11	34786.6200	0.0339	0.060	0.0339	0.50	August-88
99:	12	5	7	1	12	11	5	6	1	11	34789.4900	-0.0353	0.060	-0.0353	0.50	August-88
100:	12	5	8	1	12	11	5	7	1	11	34789.4900	-0.0353	0.060	-0.0353	0.50	August-88
101:	12	2	10	1	12	11	2	9	1	11	34790.0400	-0.0347	0.060			August-88
102:	12	6	6	1	12	11	6	5	1	11	34793.2900	-0.0033	0.060	-0.0033	0.50	August-88
103:	12	6	7	1	12	11	6	6	1	11	34793.2900	-0.0033	0.060	-0.0033	0.50	August-88
104:	12	7	5	1	12	11	7	4	1	11	34797.8300	0.0075	0.060	0.0076	0.50	August-88
105:	12	7	6	1	12	11	7	5	1	11	34797.8300	0.0075	0.060	0.0076	0.50	August-88
106:	12	8	4	1	12	11	8	3	1	11	34803.1600	0.0748	0.060	0.0749	0.50	August-88
107:	12	8	5	1	12	11	8	4	1	11	34803.1600	0.0748	0.060	0.0749	0.50	August-88
108:	12	9	3	1	12	11	9	2	1	11	34809.1000	0.0321	0.060	0.0321	0.50	August-88
109:	12	9	4	1	12	11	9	3	1	11	34809.1000	0.0321	0.060	0.0321	0.50	August-88
110:	12	1	11	1	12	11	1	10	1	11	35053.5000	0.0912	0.060			August-88
111:	13	1	13	1	13	12	1	12	1	12	37377.3700	0.0538	0.060			August-88
112:	13	0	13	1	13	12	0	12	1	12	37665.6900	0.1434	0.060			August-88
113:	13	2	12	1	13	12	2	11	1	12	37677.9700	0.0890	0.060			August-88
114:	13	3	11	1	13	12	3	10	1	12	37683.5700	0.0641	0.060	0.0388	0.50	August-88
115:	13	3	10	1	13	12	3	9	1	12	37683.5700	0.0133	0.060	0.0388	0.50	August-88
116:	13	4	10	1	13	12	4	9	1	12	37685.4800	0.0106	0.060	0.0106	0.50	August-88
117:	13	4	9	1	13	12	4	8	1	12	37685.4800	0.0105	0.060	0.0106	0.50	August-88
118:	13	5	8	1	13	12	5	7	1	12	37688.6600	0.0542	0.060	0.0542	0.50	August-88
119:	13	5	9	1	13	12	5	8	1	12	37688.6600	0.0542	0.060	0.0542	0.50	August-88
120:	13	2	11	1	13	12	2	10	1	12	37690.7000	-0.0107	0.060			August-88
121:	13	6	7	1	13	12	6	6	1	12	37692.7800	0.1173	0.060	0.1173	0.50	August-88
122:	13	6	8	1	13	12	6	7	1	12	37692.7800	0.1173	0.060	0.1173	0.50	August-88
123:	13	7	6	1	13	12	7	5	1	12	37697.6200	0.0657	0.060	0.0657	0.50	August-88
124:	13	7	7	1	13	12	7	6	1	12	37697.6200	0.0657	0.060	0.0657	0.50	August-88
125:	13	8	5	1	13	12	8	4	1	12	37703.2500	0.0040	0.060	0.0041	0.50	August-88
126:	13	8	6	1	13	12	8	5	1	12	37703.2500	0.0040	0.060	0.0041	0.50	August-88
127:	13	1	12	1	13	12	1	11	1	12	37973.9500	0.0435	0.060			August-88
128:	28	0	28	1	28	27	0	27	1	27	81019.1890	-0.0052	0.010			Melli+22
129:	28	2	27	1	28	27	2	26	1	27	81128.8601	0.0000	0.010			Melli+22
130:	28	4	24	1	28	27	4	23	1	27	81168.7505	-0.0164	0.010	-0.0076	0.50	Melli+22
131:	28	4	25	1	28	27	4	24	1	27	81168.7505	0.0012	0.010	-0.0076	0.50	Melli+22

132:	28	3	26	1	28	27	3	25	1	27	81169.2970	0.0072	0.010			Melli+22
133:	28	3	25	1	28	27	3	24	1	27	81171.6890	-0.0055	0.010			Melli+22
134:	28	5	23	1	28	27	5	22	1	27	81172.9800	-0.0019	0.010	-0.0019	0.50	Melli+22
135:	28	5	24	1	28	27	5	23	1	27	81172.9800	-0.0018	0.010	-0.0019	0.50	Melli+22
136:	28	6	22	1	28	27	6	21	1	27	81180.3920	0.0011	0.010	0.0012	0.50	Melli+22
137:	28	6	23	1	28	27	6	22	1	27	81180.3920	0.0011	0.010	0.0012	0.50	Melli+22
138:	28	7	22	1	28	27	7	21	1	27	81190.1324	-0.0056	0.010	-0.0057	0.50	Melli+22
139:	28	7	21	1	28	27	7	20	1	27	81190.1324	-0.0056	0.010	-0.0057	0.50	Melli+22
140:	28	8	21	1	28	27	8	20	1	27	81201.8820	-0.0060	0.010	-0.0061	0.50	Melli+22
141:	28	8	20	1	28	27	8	19	1	27	81201.8820	-0.0060	0.010	-0.0061	0.50	Melli+22
142:	28	9	20	1	28	27	9	19	1	27	81215.4810	-0.0029	0.010	-0.0030	0.50	Melli+22
143:	28	9	19	1	28	27	9	18	1	27	81215.4810	-0.0029	0.010	-0.0030	0.50	Melli+22
144:	28	10	18	1	28	27	10	17	1	27	81230.8420	-0.0007	0.010	-0.0008	0.50	Melli+22
145:	28	10	19	1	28	27	10	18	1	27	81230.8420	-0.0007	0.010	-0.0008	0.50	Melli+22
146:	28	11	18	1	28	27	11	17	1	27	81247.9160	0.0000	0.010	0.0000	0.50	Melli+22
147:	28	11	17	1	28	27	11	16	1	27	81247.9160	0.0000	0.010	0.0000	0.50	Melli+22
148:	28	2	26	1	28	27	2	25	1	27	81256.3820	-0.0017	0.010			Melli+22
149:	28	2	26	1	28	27	2	25	1	27	81256.3858	0.0020	0.010			Melli+22
150:	28	12	16	1	28	27	12	15	1	27	81266.6760	0.0036	0.010	0.0037	0.50	Melli+22
151:	28	12	17	1	28	27	12	16	1	27	81266.6760	0.0036	0.010	0.0037	0.50	Melli+22
152:	28	1	27	1	28	27	1	26	1	27	81756.3710	-0.0027	0.010			Melli+22
153:	29	2	28	1	29	28	2	27	1	28	84024.0680	0.0005	0.010			Melli+22
154:	29	4	25	1	29	28	4	24	1	28	84067.6387	-0.0196	0.010	-0.0083	0.50	Melli+22
155:	29	4	26	1	29	28	4	25	1	28	84067.6387	0.0030	0.010	-0.0083	0.50	Melli+22
156:	29	3	27	1	29	28	3	26	1	28	84068.5710	-0.0069	0.010			Melli+22
157:	29	3	26	1	29	28	3	25	1	28	84071.4192	-0.0248	0.010			Melli+22
158:	29	5	24	1	29	28	5	23	1	28	84071.7790	0.0001	0.010	0.0002	0.50	Melli+22
159:	29	5	25	1	29	28	5	24	1	28	84071.7790	0.0002	0.010	0.0002	0.50	Melli+22
160:	29	6	23	1	29	28	6	22	1	28	84079.3180	-0.0070	0.010	-0.0070	0.50	Melli+22
161:	29	6	24	1	29	28	6	23	1	28	84079.3180	-0.0070	0.010	-0.0070	0.50	Melli+22
162:	29	7	23	1	29	28	7	22	1	28	84089.3543	0.0095	0.010	0.0095	0.50	Melli+22
163:	29	7	22	1	29	28	7	21	1	28	84089.3543	0.0095	0.010	0.0095	0.50	Melli+22
164:	29	8	21	1	29	28	8	20	1	28	84101.4480	-0.0176	0.010	-0.0176	0.50	Melli+22
165:	29	8	22	1	29	28	8	21	1	28	84101.4480	-0.0176	0.010	-0.0176	0.50	Melli+22
166:	29	9	20	1	29	28	9	19	1	28	84115.5100	-0.0035	0.010	-0.0036	0.50	Melli+22
167:	29	9	21	1	29	28	9	20	1	28	84115.5100	-0.0035	0.010	-0.0036	0.50	Melli+22
168:	29	10	19	1	29	28	10	18	1	28	84131.3930	-0.0037	0.010	-0.0038	0.50	Melli+22
169:	29	10	20	1	29	28	10	19	1	28	84131.3930	-0.0037	0.010	-0.0038	0.50	Melli+22
170:	29	11	18	1	29	28	11	17	1	28	84149.0580	-0.0035	0.010	-0.0035	0.50	Melli+22
171:	29	11	19	1	29	28	11	18	1	28	84149.0580	-0.0035	0.010	-0.0035	0.50	Melli+22
172:	29	2	27	1	29	28	2	26	1	28	84165.5537	-0.0007	0.010			Melli+22
173:	29	2	27	1	29	28	2	26	1	28	84165.5546	0.0001	0.010			Melli+22
174:	29	12	18	1	29	28	12	17	1	28	84168.4749	0.0012	0.010	0.0012	0.50	Melli+22
175:	29	12	17	1	29	28	12	16	1	28	84168.4749	0.0012	0.010	0.0012	0.50	Melli+22
176:	29	13	16	1	29	28	13	15	1	28	84189.6210	0.0116	0.010	0.0117	0.50	Melli+22
177:	29	13	17	1	29	28	13	16	1	28	84189.6210	0.0116	0.010	0.0117	0.50	Melli+22
178:	29	1	28	1	29	28	1	27	1	28	84672.9451	-0.0004	0.010			Melli+22
179:	30	2	29	1	30	29	2	28	1	29	86919.0253	-0.0133	0.010			Melli+22
180:	30	4	26	1	30	29	4	25	1	29	86966.5290	-0.0216	0.010	-0.0073	0.50	Melli+22
181:	30	4	27	1	30	29	4	26	1	29	86966.5290	0.0070	0.010	-0.0073	0.50	Melli+22
182:	30	3	28	1	30	29	3	27	1	29	86967.8900	-0.0025	0.010			Melli+22
183:	30	5	25	1	30	29	5	24	1	29	86970.5440	-0.0067	0.010	-0.0067	0.50	Melli+22
184:	30	5	26	1	30	29	5	25	1	29	86970.5440	-0.0065	0.010	-0.0067	0.50	Melli+22
185:	30	3	27	1	30	29	3	26	1	29	86971.2884	0.0003	0.010			Melli+22
186:	30	6	24	1	30	29	6	23	1	29	86978.2128	-0.0078	0.010	-0.0078	0.50	Melli+22
187:	30	6	25	1	30	29	6	24	1	29	86978.2128	-0.0078	0.010	-0.0078	0.50	Melli+22
188:	30	7	24	1	30	29	7	23	1	29	86988.4940	-0.0110	0.010	-0.0110	0.50	Melli+22
189:	30	7	23	1	30	29	7	22	1	29	86988.4940	-0.0110	0.010	-0.0110	0.50	Melli+22
190:	30	8	23	1	30	29	8	22	1	29	87000.9768	-0.0148	0.010	-0.0148	0.50	Melli+22
191:	30	8	22	1	30	29	8	21	1	29	87000.9768	-0.0148	0.010	-0.0148	0.50	Melli+22
192:	30	9	22	1	30	29	9	21	1	29	87015.4760	-0.0121	0.010	-0.0121	0.50	Melli+22
193:	30	9	21	1	30	29	9	20	1	29	87015.4760	-0.0121	0.010	-0.0121	0.50	Melli+22
194:	30	10	20	1	30	29	10	19	1	29	87031.8910	-0.0021	0.010	-0.0021	0.50	Melli+22
195:	30	10	21	1	30	29	10	20	1	29	87031.8910	-0.0021	0.010	-0.0021	0.50	Melli+22
196:	30	11	20	1	30	29	11	19	1	29	87050.1210	-0.0265	0.010	-0.0266	0.50	Melli+22
197:	30	11	19	1	30	29	11	18	1	29	87050.1210	-0.0265	0.010	-0.0266	0.50	Melli+22
198:	30	12	18	1	30	29	12	17	1	29	87070.2080	-0.0059	0.010	-0.0060	0.50	Melli+22
199:	30	12	19	1	30	29	12	18	1	29	87070.2080	-0.0059	0.010	-0.0060	0.50	Melli+22
200:	30	2	28	1	30	29	2	27	1	29	87075.4238	-0.0107	0.010			Melli+22

201:	30	13	17	1	30	29	13	16	1	29	87092.0640	-0.0023	0.010	-0.0024	0.50	Melli+22
202:	30	13	18	1	30	29	13	17	1	29	87092.0640	-0.0023	0.010	-0.0024	0.50	Melli+22
203:	30	14	16	1	30	29	14	15	1	29	87115.6796	-0.0057	0.010	-0.0057	0.50	Melli+22
204:	30	14	17	1	30	29	14	16	1	29	87115.6796	-0.0057	0.010	-0.0057	0.50	Melli+22
205:	30	15	15	1	30	29	15	14	1	29	87141.0630	0.0077	0.010	0.0078	0.50	Melli+22
206:	30	15	16	1	30	29	15	15	1	29	87141.0630	0.0077	0.010	0.0078	0.50	Melli+22
207:	30	1	29	1	30	29	1	28	1	29	87589.1520	-0.0040	0.010			Melli+22
208/	31	0	31	1	31	30	0	30	1	30	89666.6281	0.0391	0.010			Melli+22
209:	31	2	30	1	31	30	2	29	1	30	89813.7640	-0.0016	0.010			Melli+22
210:	31	4	27	1	31	30	4	26	1	30	89865.4280	-0.0160	0.010	0.0020	0.50	Melli+22
211:	31	4	28	1	31	30	4	27	1	30	89865.4280	0.0200	0.010	0.0020	0.50	Melli+22
212:	31	3	29	1	31	30	3	28	1	30	89867.2375	0.0053	0.010			Melli+22
213:	31	5	26	1	31	30	5	25	1	30	89869.2930	-0.0035	0.010	-0.0035	0.50	Melli+22
214:	31	5	27	1	31	30	5	26	1	30	89869.2930	-0.0034	0.010	-0.0035	0.50	Melli+22
215:	31	3	28	1	31	30	3	27	1	30	89871.2320	-0.0001	0.010			Melli+22
216:	31	6	25	1	31	30	6	24	1	30	89877.0729	-0.0035	0.010	-0.0036	0.50	Melli+22
217:	31	6	26	1	31	30	6	25	1	30	89877.0729	-0.0035	0.010	-0.0036	0.50	Melli+22
218:	31	7	24	1	31	30	7	23	1	30	89887.6079	-0.0092	0.010	-0.0093	0.50	Melli+22
219:	31	7	25	1	31	30	7	24	1	30	89887.6079	-0.0092	0.010	-0.0093	0.50	Melli+22
220:	31	8	23	1	31	30	8	22	1	30	89900.4580	-0.0062	0.010	-0.0063	0.25	Melli+22
221:	31	8	24	1	31	30	8	23	1	30	89900.4580	-0.0062	0.010	-0.0063	0.25	Melli+22
222:	31	8	23	1	31	30	8	22	1	30	89900.4580	-0.0062	0.010	-0.0063	0.25	Melli+22
223:	31	8	24	1	31	30	8	23	1	30	89900.4580	-0.0062	0.010	-0.0063	0.25	Melli+22
224:	31	9	22	1	31	30	9	21	1	30	89915.3952	-0.0104	0.010	-0.0105	0.50	Melli+22
225:	31	9	23	1	31	30	9	22	1	30	89915.3952	-0.0104	0.010	-0.0105	0.50	Melli+22
226:	31	10	22	1	31	30	10	21	1	30	89932.3350	0.0051	0.010	0.0051	0.50	Melli+22
227:	31	10	21	1	31	30	10	20	1	30	89932.3350	0.0051	0.010	0.0051	0.50	Melli+22
228:	31	11	20	1	31	30	11	19	1	30	89951.1790	0.0069	0.010	0.0069	0.50	Melli+22
229:	31	11	21	1	31	30	11	20	1	30	89951.1790	0.0069	0.010	0.0069	0.50	Melli+22
230:	31	12	20	1	31	30	12	19	1	30	89971.8920	0.0008	0.010	0.0008	0.50	Melli+22
231:	31	12	19	1	31	30	12	18	1	30	89971.8920	0.0008	0.010	0.0008	0.50	Melli+22
232:	31	2	29	1	31	30	2	28	1	30	89986.0370	-0.0012	0.010			Melli+22
233:	31	2	29	1	31	30	2	28	1	30	89986.0380	-0.0002	0.010			Melli+22
234:	31	13	19	1	31	30	13	18	1	30	89994.4650	0.0059	0.010	0.0060	0.50	Melli+22
235:	31	13	18	1	31	30	13	17	1	30	89994.4650	0.0059	0.010	0.0060	0.50	Melli+22
236:	31	14	18	1	31	30	14	17	1	30	90018.8590	0.0043	0.010	0.0043	0.50	Melli+22
237:	31	14	17	1	31	30	14	16	1	30	90018.8590	0.0043	0.010	0.0043	0.50	Melli+22
238:	31	15	17	1	31	30	15	16	1	30	90045.0721	0.0108	0.010	0.0109	0.50	Melli+22
239:	31	15	16	1	31	30	15	15	1	30	90045.0721	0.0108	0.010	0.0109	0.50	Melli+22
240:	31	1	30	1	31	30	1	29	1	30	90504.9878	-0.0026	0.010			Melli+22
241:	32	0	32	1	32	31	0	31	1	31	92546.9570	-0.0042	0.010			Melli+22
242:	32	2	31	1	32	31	2	30	1	31	92708.2370	-0.0034	0.010			Melli+22
243:	32	4	28	1	32	31	4	27	1	31	92764.3140	-0.0246	0.010	-0.0021	0.50	Melli+22
244:	32	4	29	1	32	31	4	28	1	31	92764.3140	0.0205	0.010	-0.0021	0.50	Melli+22
245:	32	3	30	1	32	31	3	29	1	31	92766.5939	-0.0011	0.010			Melli+22
246:	32	5	27	1	32	31	5	26	1	31	92768.0111	-0.0044	0.010	-0.0043	0.50	Melli+22
247:	32	5	28	1	32	31	5	27	1	31	92768.0111	-0.0042	0.010	-0.0043	0.50	Melli+22
248:	32	3	29	1	32	31	3	28	1	31	92771.2844	0.0018	0.010			Melli+22
249:	32	6	26	1	32	31	6	25	1	31	92775.8860	-0.0051	0.010	-0.0051	0.50	Melli+22
250:	32	6	27	1	32	31	6	26	1	31	92775.8860	-0.0051	0.010	-0.0051	0.50	Melli+22
251:	32	7	25	1	32	31	7	24	1	31	92786.6730	-0.0067	0.010	-0.0067	0.50	Melli+22
252:	32	7	26	1	32	31	7	25	1	31	92786.6730	-0.0067	0.010	-0.0067	0.50	Melli+22
253:	32	8	25	1	32	31	8	24	1	31	92799.8762	-0.0055	0.010	-0.0056	0.50	Melli+22
254:	32	8	24	1	32	31	8	23	1	31	92799.8762	-0.0055	0.010	-0.0056	0.50	Melli+22
255:	32	9	24	1	32	31	9	23	1	31	92815.2585	-0.0058	0.010	-0.0058	0.50	Melli+22
256:	32	9	23	1	32	31	9	22	1	31	92815.2585	-0.0058	0.010	-0.0058	0.50	Melli+22
257:	32	10	22	1	32	31	10	21	1	31	92832.6981	-0.0069	0.010	-0.0069	0.50	Melli+22
258:	32	10	23	1	32	31	10	22	1	31	92832.6981	-0.0069	0.010	-0.0069	0.50	Melli+22
259:	32	11	22	1	32	31	11	21	1	31	92852.1348	0.0019	0.010	0.0019	0.50	Melli+22
260:	32	11	21	1	32	31	11	20	1	31	92852.1348	0.0019	0.010	0.0019	0.50	Melli+22
261:	32	12	20	1	32	31	12	19	1	31	92873.5089	0.0057	0.010	0.0057	0.50	Melli+22
262:	32	12	21	1	32	31	12	20	1	31	92873.5089	0.0057	0.010	0.0057	0.50	Melli+22
263:	32	13	20	1	32	31	13	19	1	31	92896.7910	0.0057	0.010	0.0057	0.50	Melli+22
264:	32	13	19	1	32	31	13	18	1	31	92896.7910	0.0057	0.010	0.0057	0.50	Melli+22
265:	32	2	30	1	32	31	2	29	1	31	92897.3806	0.0027	0.010			Melli+22
266:	32	2	30	1	32	31	2	29	1	31	92897.3826	0.0047	0.010			Melli+22
267:	32	14	18	1	32	31	14	17	1	31	92921.9699	0.0134	0.010	0.0135	0.50	Melli+22
268:	32	14	19	1	32	31	14	18	1	31	92921.9699	0.0134	0.010	0.0135	0.50	Melli+22
269/	32	15	18	1	32	31	15	17	1	31	92948.9560	-0.0428	0.010	-0.0428	0.50	Melli+22

270:	32	15	17	1	32	31	15	16	1	31	92948.9560	-0.0428	0.010	-0.0428	0.50	Melli+22
271:	32	1	31	1	32	31	1	30	1	31	93420.4405	0.0069	0.010			Melli+22
272:	33	0	33	1	33	32	0	32	1	32	95426.2420	-0.0083	0.010			Melli+22
273:	33	2	32	1	33	32	2	31	1	32	95602.4585	0.0034	0.010			Melli+22
274:	33	4	29	1	33	32	4	28	1	32	95663.2020	-0.0326	0.010	-0.0046	0.50	Melli+22
275:	33	4	30	1	33	32	4	29	1	32	95663.2020	0.0234	0.010	-0.0046	0.50	Melli+22
276:	33	3	31	1	33	32	3	30	1	32	95665.9770	-0.0024	0.010			Melli+22
277:	33	5	28	1	33	32	5	27	1	32	95666.6978	-0.0089	0.010	-0.0088	0.50	Melli+22
278:	33	5	29	1	33	32	5	28	1	32	95666.6978	-0.0086	0.010	-0.0088	0.50	Melli+22
279:	33	3	30	1	33	32	3	29	1	32	95671.4400	-0.0053	0.010			Melli+22
280:	33	6	27	1	33	32	6	26	1	32	95674.6570	-0.0062	0.010	-0.0062	0.50	Melli+22
281:	33	6	28	1	33	32	6	27	1	32	95674.6570	-0.0062	0.010	-0.0062	0.50	Melli+22
282:	33	7	26	1	33	32	7	25	1	32	95685.6864	-0.0046	0.010	-0.0046	0.50	Melli+22
283:	33	7	27	1	33	32	7	26	1	32	95685.6864	-0.0046	0.010	-0.0046	0.50	Melli+22
284:	33	8	25	1	33	32	8	24	1	32	95699.2360	-0.0063	0.010	-0.0064	0.50	Melli+22
285:	33	8	26	1	33	32	8	25	1	32	95699.2360	-0.0063	0.010	-0.0064	0.50	Melli+22
286:	33	8	25	1	33	32	8	24	1	32	95699.2363	-0.0060	0.010	-0.0061	0.50	Melli+22
287:	33	8	26	1	33	32	8	25	1	32	95699.2363	-0.0060	0.010	-0.0061	0.50	Melli+22
288:	33	9	25	1	33	32	9	24	1	32	95715.0540	-0.0082	0.010	-0.0082	0.50	Melli+22
289:	33	9	24	1	33	32	9	23	1	32	95715.0540	-0.0082	0.010	-0.0082	0.50	Melli+22
290:	33	9	25	1	33	32	9	24	1	32	95715.0542	-0.0080	0.010	-0.0080	0.50	Melli+22
291:	33	9	24	1	33	32	9	23	1	32	95715.0542	-0.0080	0.010	-0.0080	0.50	Melli+22
292:	33	10	23	1	33	32	10	22	1	32	95733.0169	0.0002	0.010	0.0003	0.50	Melli+22
293:	33	10	24	1	33	32	10	23	1	32	95733.0169	0.0002	0.010	0.0003	0.50	Melli+22
294:	33	11	22	1	33	32	11	21	1	32	95753.0240	-0.0040	0.010	-0.0041	0.50	Melli+22
295:	33	11	23	1	33	32	11	22	1	32	95753.0240	-0.0040	0.010	-0.0041	0.50	Melli+22
296:	33	12	22	1	33	32	12	21	1	32	95775.0528	0.0049	0.010	0.0049	0.50	Melli+22
297:	33	12	21	1	33	32	12	20	1	32	95775.0528	0.0049	0.010	0.0049	0.50	Melli+22
298:	33	13	21	1	33	32	13	20	1	32	95799.0550	0.0121	0.010	0.0122	0.50	Melli+22
299:	33	13	20	1	33	32	13	19	1	32	95799.0550	0.0121	0.010	0.0122	0.50	Melli+22
300:	33	2	31	1	33	32	2	30	1	32	95809.4704	0.0063	0.010			Melli+22
301:	33	15	18	1	33	32	15	17	1	32	95852.8813	0.0155	0.010	0.0155	0.50	Melli+22
302:	33	15	19	1	33	32	15	18	1	32	95852.8813	0.0155	0.010	0.0155	0.50	Melli+22
303:	33	1	32	1	33	32	1	31	1	32	96335.4780	0.0078	0.010			Melli+22
304:	34	1	34	1	34	33	1	33	1	33	97697.8700	-0.0041	0.010			Melli+22
305:	34	0	34	1	34	33	0	33	1	33	98304.4323	-0.0047	0.010			Melli+22
306:	34	2	33	1	34	33	2	32	1	33	98496.4040	0.0023	0.010			Melli+22
307:	34	4	30	1	34	33	4	29	1	33	98562.0970	-0.0350	0.010	-0.0005	0.50	Melli+22
308:	34	4	31	1	34	33	4	30	1	33	98562.0970	0.0340	0.010	-0.0005	0.50	Melli+22
309:	34	3	32	1	34	33	3	31	1	33	98565.3749	-0.0082	0.010	0.0011	0.33	Melli+22
310:	34	5	29	1	34	33	5	28	1	33	98565.3749	0.0055	0.010	0.0011	0.33	Melli+22
311:	34	5	30	1	34	33	5	29	1	33	98565.3749	0.0059	0.010	0.0011	0.33	Melli+22
312:	34	3	31	1	34	33	3	30	1	33	98571.7287	0.0014	0.010			Melli+22
313:	34	6	28	1	34	33	6	27	1	33	98573.3848	-0.0067	0.010	-0.0067	0.50	Melli+22
314:	34	6	29	1	34	33	6	28	1	33	98573.3848	-0.0067	0.010	-0.0067	0.50	Melli+22
315:	34	7	27	1	34	33	7	26	1	33	98584.6464	-0.0030	0.010	-0.0030	0.50	Melli+22
316:	34	7	28	1	34	33	7	27	1	33	98584.6464	-0.0030	0.010	-0.0030	0.50	Melli+22
317:	34	8	27	1	34	33	8	26	1	33	98598.5392	-0.0051	0.010	-0.0051	0.50	Melli+22
318:	34	8	26	1	34	33	8	25	1	33	98598.5392	-0.0051	0.010	-0.0051	0.50	Melli+22
319:	34	9	25	1	34	33	9	24	1	33	98614.7951	-0.0023	0.010	-0.0024	0.50	Melli+22
320:	34	9	26	1	34	33	9	25	1	33	98614.7951	-0.0023	0.010	-0.0024	0.50	Melli+22
321:	34	10	25	1	34	33	10	24	1	33	98633.2870	0.0243	0.010	0.0244	0.50	Melli+22
322:	34	10	24	1	34	33	10	23	1	33	98633.2870	0.0243	0.010	0.0244	0.50	Melli+22
323:	34	11	24	1	34	33	11	23	1	33	98653.8560	0.0004	0.010	0.0005	0.50	Melli+22
324:	34	11	23	1	34	33	11	22	1	33	98653.8560	0.0004	0.010	0.0005	0.50	Melli+22
325:	34	12	22	1	34	33	12	21	1	33	98676.5490	0.0258	0.010	0.0259	0.50	Melli+22
326:	34	12	23	1	34	33	12	22	1	33	98676.5490	0.0258	0.010	0.0259	0.50	Melli+22
327:	34	13	21	1	34	33	13	20	1	33	98701.2333	0.0036	0.010	0.0037	0.50	Melli+22
328:	34	13	22	1	34	33	13	21	1	33	98701.2333	0.0036	0.010	0.0037	0.50	Melli+22
329:	34	2	32	1	34	33	2	31	1	33	98722.3072	0.0015	0.010			Melli+22
330:	34	14	20	1	34	33	14	19	1	33	98727.9655	0.0167	0.010	0.0167	0.50	Melli+22
331:	34	14	21	1	34	33	14	20	1	33	98727.9655	0.0167	0.010	0.0167	0.50	Melli+22
332:	34	15	19	1	34	33	15	18	1	33	98756.6450	-0.0149	0.010	-0.0150	0.50	Melli+22
333:	34	15	20	1	34	33	15	19	1	33	98756.6450	-0.0149	0.010	-0.0150	0.50	Melli+22
334:	34	1	33	1	34	33	1	32	1	33	99250.0849	0.0003	0.010			Melli+22
335:	35	1	35	1	35	34	1	34	1	34	100567.3340	0.0004	0.010			Melli+22
336:	35	0	35	1	35	34	0	34	1	34	101181.5050	0.0010	0.010			Melli+22
337:	35	2	34	1	35	34	2	33	1	34	101390.0759	0.0036	0.010			Melli+22
338:	35	4	31	1	35	34	4	30	1	34	101461.0150	-0.0161	0.010			Melli+22

339:	35	5	30	1	35	34	5	29	1	34	101464.0183	0.0158	0.010	0.0161	0.50	Melli+22
340:	35	5	31	1	35	34	5	30	1	34	101464.0183	0.0163	0.010	0.0161	0.50	Melli+22
341:	35	3	33	1	35	34	3	32	1	34	101464.8219	0.0180	0.010			Melli+22
342:	35	3	32	1	35	34	3	31	1	34	101472.1140	-0.0208	0.010	0.0193	0.33	Melli+22
343:	35	6	29	1	35	34	6	28	1	34	101472.1140	0.0394	0.010	0.0193	0.33	Melli+22
344:	35	6	30	1	35	34	6	29	1	34	101472.1140	0.0394	0.010	0.0193	0.33	Melli+22
345:	35	7	28	1	35	34	7	27	1	34	101483.5672	0.0138	0.010	0.0138	0.50	Melli+22
346:	35	7	29	1	35	34	7	28	1	34	101483.5672	0.0138	0.010	0.0138	0.50	Melli+22
347:	35	8	27	1	35	34	8	26	1	34	101497.7995	0.0136	0.010	0.0137	0.25	Melli+22
348:	35	8	28	1	35	34	8	27	1	34	101497.7995	0.0136	0.010	0.0137	0.25	Melli+22
349:	35	8	27	1	35	34	8	26	1	34	101497.7995	0.0136	0.010	0.0137	0.25	Melli+22
350:	35	8	28	1	35	34	8	27	1	34	101497.7995	0.0136	0.010	0.0137	0.25	Melli+22
351:	35	9	27	1	35	34	9	26	1	34	101514.4660	-0.0021	0.010	-0.0021	0.50	Melli+22
352:	35	9	26	1	35	34	9	25	1	34	101514.4660	-0.0021	0.010	-0.0021	0.50	Melli+22
353:	35	10	25	1	35	34	10	24	1	34	101533.4412	0.0000	0.010	0.0001	0.50	Melli+22
354:	35	10	26	1	35	34	10	25	1	34	101533.4412	0.0000	0.010	0.0001	0.50	Melli+22
355:	35	11	24	1	35	34	11	23	1	34	101554.6160	0.0028	0.010	0.0028	0.50	Melli+22
356:	35	11	25	1	35	34	11	24	1	34	101554.6160	0.0028	0.010	0.0028	0.50	Melli+22
357:	35	12	24	1	35	34	12	23	1	34	101577.9368	0.0098	0.010	0.0099	0.50	Melli+22
358:	35	12	23	1	35	34	12	22	1	34	101577.9368	0.0098	0.010	0.0099	0.50	Melli+22
359:	35	13	23	1	35	34	13	22	1	34	101603.3580	0.0144	0.010	0.0144	0.50	Melli+22
360:	35	13	22	1	35	34	13	21	1	34	101603.3580	0.0144	0.010	0.0144	0.50	Melli+22
361:	35	14	21	1	35	34	14	20	1	34	101630.8370	0.0020	0.010	0.0020	0.50	Melli+22
362:	35	14	22	1	35	34	14	21	1	34	101630.8370	0.0020	0.010	0.0020	0.50	Melli+22
363:	35	2	33	1	35	34	2	32	1	34	101635.9158	0.0063	0.010			Melli+22
364:	35	1	34	1	35	34	1	33	1	34	102164.2630	0.0022	0.010			Melli+22
365:	36	1	36	1	36	35	1	35	1	35	103436.4701	0.0010	0.010			Melli+22
366:	36	0	36	1	36	35	0	35	1	35	104057.4282	-0.0077	0.010			Melli+22
367:	36	2	35	1	36	35	2	34	1	35	104283.4437	-0.0153	0.010			Melli+22
368/	36	4	33	1	36	35	4	32	1	35	104359.8640	0.0349	0.010			Melli+22
369:	36	5	31	1	36	35	5	30	1	35	104362.5880	-0.0171	0.010	-0.0168	0.50	Melli+22
370:	36	5	32	1	36	35	5	31	1	35	104362.5880	-0.0164	0.010	-0.0168	0.50	Melli+22
371:	36	3	34	1	36	35	3	33	1	35	104364.2231	-0.0157	0.010			Melli+22
372:	36	6	30	1	36	35	6	29	1	35	104370.6908	-0.0203	0.010	-0.0204	0.50	Melli+22
373:	36	6	31	1	36	35	6	30	1	35	104370.6908	-0.0203	0.010	-0.0204	0.50	Melli+22
374:	36	3	33	1	36	35	3	32	1	35	104372.6615	-0.0138	0.010			Melli+22
375:	36	7	30	1	36	35	7	29	1	35	104382.3820	-0.0192	0.010	-0.0193	0.50	Melli+22
376:	36	7	29	1	36	35	7	28	1	35	104382.3820	-0.0192	0.010	-0.0193	0.50	Melli+22
377:	36	8	29	1	36	35	8	28	1	35	104396.9460	-0.0190	0.010	-0.0191	0.50	Melli+22
378:	36	8	28	1	36	35	8	27	1	35	104396.9460	-0.0190	0.010	-0.0191	0.50	Melli+22
379:	36	9	27	1	36	35	9	26	1	35	104414.0860	0.0137	0.010	0.0137	0.50	Melli+22
380:	36	9	28	1	36	35	9	27	1	35	104414.0860	0.0137	0.010	0.0137	0.50	Melli+22
381:	36	10	27	1	36	35	10	26	1	35	104433.5652	0.0151	0.010	0.0151	0.50	Melli+22
382:	36	10	26	1	36	35	10	25	1	35	104433.5652	0.0151	0.010	0.0151	0.50	Melli+22
383:	36	11	26	1	36	35	11	25	1	35	104455.2998	0.0007	0.010	0.0008	0.50	Melli+22
384:	36	11	25	1	36	35	11	24	1	35	104455.2998	0.0007	0.010	0.0008	0.50	Melli+22
385:	36	12	24	1	36	35	12	23	1	35	104479.2688	0.0117	0.010	0.0117	0.50	Melli+22
386:	36	12	25	1	36	35	12	24	1	35	104479.2688	0.0117	0.010	0.0117	0.50	Melli+22
387:	36	13	23	1	36	35	13	22	1	35	104505.3579	-0.0245	0.010	-0.0246	0.50	Melli+22
388:	36	13	24	1	36	35	13	23	1	35	104505.3579	-0.0245	0.010	-0.0246	0.50	Melli+22
389:	36	14	23	1	36	35	14	22	1	35	104533.6463	0.0013	0.010	0.0014	0.50	Melli+22
390:	36	14	22	1	36	35	14	21	1	35	104533.6463	0.0013	0.010	0.0014	0.50	Melli+22
391:	36	2	34	1	36	35	2	33	1	35	104550.3050	0.0250	0.010			Melli+22
392:	36	15	21	1	36	35	15	20	1	35	104564.0160	-0.0050	0.010	-0.0051	0.50	Melli+22
393:	36	15	22	1	36	35	15	21	1	35	104564.0160	-0.0050	0.010	-0.0051	0.50	Melli+22
394:	36	1	35	1	36	35	1	34	1	35	105077.9901	0.0074	0.010			Melli+22
395:	37	1	37	1	37	36	1	36	1	36	106305.2730	-0.0017	0.010			Melli+22
396:	37	0	37	1	37	36	0	36	1	36	106932.2147	-0.0053	0.010			Melli+22
397:	37	2	36	1	37	36	2	35	1	36	107176.5512	-0.0030	0.010			Melli+22
398:	37	5	32	1	37	36	5	31	1	36	107261.1716	-0.0049	0.010	-0.0046	0.50	Melli+22
399:	37	5	33	1	37	36	5	32	1	36	107261.1716	-0.0041	0.010	-0.0046	0.50	Melli+22
400:	37	3	35	1	37	36	3	34	1	36	107263.6827	-0.0025	0.010			Melli+22
401:	37	6	31	1	37	36	6	30	1	36	107269.2958	-0.0041	0.010	-0.0041	0.50	Melli+22
402:	37	6	32	1	37	36	6	31	1	36	107269.2958	-0.0041	0.010	-0.0041	0.50	Melli+22
403:	37	3	34	1	37	36	3	33	1	36	107273.3561	0.0000	0.010			Melli+22
404:	37	7	30	1	37	36	7	29	1	36	107281.1867	-0.0047	0.010	-0.0048	0.50	Melli+22
405:	37	7	31	1	37	36	7	30	1	36	107281.1867	-0.0047	0.010	-0.0048	0.50	Melli+22
406:	37	8	30	1	37	36	8	29	1	36	107296.0731	-0.0071	0.010	-0.0072	0.50	Melli+22
407:	37	8	29	1	37	36	8	28	1	36	107296.0731	-0.0071	0.010	-0.0072	0.50	Melli+22

408:	37	9	28	1	37	36	9	27	1	36	107313.6036	-0.0044	0.010	-0.0045	0.50	Melli+22
409:	37	9	29	1	37	36	9	28	1	36	107313.6036	-0.0044	0.010	-0.0045	0.50	Melli+22
410:	37	10	27	1	37	36	10	26	1	36	107333.5850	-0.0025	0.010	-0.0025	0.50	Melli+22
411:	37	10	28	1	37	36	10	27	1	36	107333.5850	-0.0025	0.010	-0.0025	0.50	Melli+22
412:	37	11	26	1	37	36	11	25	1	36	107355.9100	-0.0010	0.010	-0.0010	0.50	Melli+22
413:	37	11	27	1	37	36	11	26	1	36	107355.9100	-0.0010	0.010	-0.0010	0.50	Melli+22
414:	37	12	26	1	37	36	12	25	1	36	107380.5086	-0.0029	0.010	-0.0030	0.50	Melli+22
415:	37	12	25	1	37	36	12	24	1	36	107380.5086	-0.0029	0.010	-0.0030	0.50	Melli+22
416:	37	13	24	1	37	36	13	23	1	36	107407.3480	0.0038	0.010	0.0038	0.50	Melli+22
417:	37	13	25	1	37	36	13	24	1	36	107407.3480	0.0038	0.010	0.0038	0.50	Melli+22
418:	37	2	35	1	37	36	2	34	1	36	107465.4210	0.0014	0.010			Melli+22
419:	37	16	21	1	37	36	16	20	1	36	107500.9490	0.0040	0.010	0.0040	0.50	Melli+22
420:	37	16	22	1	37	36	16	21	1	36	107500.9490	0.0040	0.010	0.0040	0.50	Melli+22
421:	37	1	36	1	37	36	1	35	1	36	107991.2337	-0.0001	0.010			Melli+22
422:	38	0	38	1	38	37	0	37	1	37	109805.8535	0.0080	0.010			Melli+22
423:	38	2	37	1	38	37	2	36	1	37	110069.3470	-0.0029	0.010			Melli+22
424:	38	5	33	1	38	37	5	32	1	37	110159.7138	-0.0020	0.010	-0.0015	0.50	Melli+22
425:	38	5	34	1	38	37	5	33	1	37	110159.7138	-0.0010	0.010	-0.0015	0.50	Melli+22
426:	38	3	36	1	38	37	3	35	1	37	110163.1380	-0.0021	0.010			Melli+22
427:	38	6	32	1	38	37	6	31	1	37	110167.8340	-0.0054	0.010	-0.0054	0.50	Melli+22
428:	38	6	33	1	38	37	6	32	1	37	110167.8340	-0.0054	0.010	-0.0054	0.50	Melli+22
429:	38	3	35	1	38	37	3	34	1	37	110174.1829	-0.0018	0.010			Melli+22
430:	38	7	31	1	38	37	7	30	1	37	110179.9149	-0.0075	0.010	-0.0075	0.50	Melli+22
431:	38	7	32	1	38	37	7	31	1	37	110179.9149	-0.0075	0.010	-0.0075	0.50	Melli+22
432:	38	8	30	1	38	37	8	29	1	37	110195.1250	-0.0047	0.010	-0.0047	0.50	Melli+22
433:	38	8	31	1	38	37	8	30	1	37	110195.1250	-0.0047	0.010	-0.0047	0.50	Melli+22
434:	38	9	30	1	38	37	9	29	1	37	110213.0810	0.0073	0.010	0.0074	0.50	Melli+22
435:	38	9	29	1	38	37	9	28	1	37	110213.0810	0.0073	0.010	0.0074	0.50	Melli+22
436:	38	10	29	1	38	37	10	28	1	37	110233.5490	-0.0024	0.010	-0.0025	0.50	Melli+22
437:	38	10	28	1	38	37	10	27	1	37	110233.5490	-0.0024	0.010	-0.0025	0.50	Melli+22
438:	38	11	28	1	38	37	11	27	1	37	110256.4540	0.0069	0.010	0.0069	0.50	Melli+22
439:	38	11	27	1	38	37	11	26	1	37	110256.4540	0.0069	0.010	0.0069	0.50	Melli+22
440:	38	12	27	1	38	37	12	26	1	37	110281.6970	0.0087	0.010	0.0088	0.50	Melli+22
441:	38	12	26	1	38	37	12	25	1	37	110281.6970	0.0087	0.010	0.0088	0.50	Melli+22
442:	38	13	26	1	38	37	13	25	1	37	110309.2302	0.0035	0.010	0.0036	0.50	Melli+22
443:	38	13	25	1	38	37	13	24	1	37	110309.2302	0.0035	0.010	0.0036	0.50	Melli+22
444:	38	14	24	1	38	37	14	23	1	37	110339.0372	0.0096	0.010	0.0096	0.50	Melli+22
445:	38	14	25	1	38	37	14	24	1	37	110339.0372	0.0096	0.010	0.0096	0.50	Melli+22
446:	38	15	24	1	38	37	15	23	1	37	110371.0814	0.0168	0.010	0.0168	0.50	Melli+22
447:	38	15	23	1	38	37	15	22	1	37	110371.0814	0.0168	0.010	0.0168	0.50	Melli+22
448/	38	2	36	1	38	37	2	35	1	37	110381.3660	0.0374	0.010			Melli+22
449:	38	1	37	1	38	37	1	36	1	37	110904.0030	0.0054	0.010			Melli+22
450:	39	1	39	1	39	38	1	38	1	38	112041.8560	-0.0182	0.010			Melli+22
451:	39	0	39	1	39	38	0	38	1	38	112678.3199	0.0158	0.010			Melli+22
452:	39	2	38	1	39	38	2	37	1	38	112961.8630	0.0245	0.010			Melli+22
453:	41	1	41	1	41	40	1	40	1	40	117777.0880	-0.0021	0.010			Melli+22
454:	41	0	41	1	41	40	0	40	1	40	118419.6970	-0.0029	0.010			Melli+22
455:	41	2	40	1	41	40	2	39	1	40	118745.8630	0.0001	0.010			Melli+22
456:	41	4	38	1	41	40	4	37	1	40	118854.2160	-0.0015	0.010			Melli+22
457:	41	4	37	1	41	40	4	36	1	40	118854.4682	-0.0056	0.010			Melli+22
458:	41	5	36	1	41	40	5	35	1	40	118855.1250	-0.0071	0.010	-0.0061	0.50	Melli+22
459:	41	5	37	1	41	40	5	36	1	40	118855.1250	-0.0050	0.010	-0.0061	0.50	Melli+22
460:	41	3	39	1	41	40	3	38	1	40	118861.5140	-0.0065	0.010			Melli+22
461:	41	6	35	1	41	40	6	34	1	40	118863.1410	-0.0085	0.010	-0.0085	0.50	Melli+22
462:	41	6	36	1	41	40	6	35	1	40	118863.1410	-0.0085	0.010	-0.0085	0.50	Melli+22
463:	41	7	34	1	41	40	7	33	1	40	118875.7337	-0.0096	0.010	-0.0097	0.50	Melli+22
464:	41	7	35	1	41	40	7	34	1	40	118875.7337	-0.0096	0.010	-0.0097	0.50	Melli+22
465:	41	3	38	1	41	40	3	37	1	40	118877.6380	-0.0011	0.010			Melli+22
466:	41	8	34	1	41	40	8	33	1	40	118891.8540	-0.0114	0.010	-0.0115	0.50	Melli+22
467:	41	8	33	1	41	40	8	32	1	40	118891.8540	-0.0114	0.010	-0.0115	0.50	Melli+22
468:	41	9	32	1	41	40	9	31	1	40	118911.0280	-0.0016	0.010	-0.0017	0.50	Melli+22
469:	41	9	33	1	41	40	9	32	1	40	118911.0280	-0.0016	0.010	-0.0017	0.50	Melli+22
470:	41	10	32	1	41	40	10	31	1	40	118932.9860	0.0034	0.010	0.0034	0.50	Melli+22
471:	41	10	31	1	41	40	10	30	1	40	118932.9860	0.0034	0.010	0.0034	0.50	Melli+22
472:	41	11	30	1	41	40	11	29	1	40	118957.5782	-0.0011	0.010	-0.0011	0.50	Melli+22
473:	41	11	31	1	41	40	11	30	1	40	118957.5782	-0.0011	0.010	-0.0011	0.50	Melli+22
474:	41	12	30	1	41	40	12	29	1	40	118984.7381	0.0076	0.010	0.0076	0.50	Melli+22
475:	41	12	29	1	41	40	12	28	1	40	118984.7381	0.0076	0.010	0.0076	0.50	Melli+22
476:	41	13	29	1	41	40	13	28	1	40	119014.3910	0.0143	0.010	0.0143	0.50	Melli+22

477:	41	13	28	1	41	40	13	27	1	40	119014.3910	0.0143	0.010	0.0143	0.50	Melli+22
478:	41	14	28	1	41	40	14	27	1	40	119046.4880	0.0122	0.010	0.0123	0.50	Melli+22
479:	41	14	27	1	41	40	14	26	1	40	119046.4880	0.0122	0.010	0.0123	0.50	Melli+22
480:	41	15	27	1	41	40	15	26	1	40	119081.0024	0.0065	0.010	0.0066	0.50	Melli+22
481:	41	15	26	1	41	40	15	25	1	40	119081.0024	0.0065	0.010	0.0066	0.50	Melli+22
482:	41	16	25	1	41	40	16	24	1	40	119117.9267	0.0152	0.010	0.0153	0.50	Melli+22
483:	41	16	26	1	41	40	16	25	1	40	119117.9267	0.0152	0.010	0.0153	0.50	Melli+22
484:	41	2	39	1	41	40	2	38	1	40	119133.6330	-0.0015	0.010			Melli+22
485:	41	1	40	1	41	40	1	39	1	40	119639.1931	0.0009	0.010			Melli+22
486:	42	1	42	1	42	41	1	41	1	41	120644.1620	-0.0051	0.010			Melli+22
487:	42	0	42	1	42	41	0	41	1	41	121288.6231	-0.0102	0.010			Melli+22
488:	42	2	41	1	42	41	2	40	1	41	121637.3890	0.0055	0.010			Melli+22
489:	42	4	39	1	42	41	4	38	1	41	121753.0624	-0.0259	0.010			Melli+22
490:	42	3	40	1	42	41	3	39	1	41	121760.9769	0.0036	0.010			Melli+22
491:	42	6	36	1	42	41	6	35	1	41	121761.4780	-0.0009	0.010	-0.0009	0.50	Melli+22
492:	42	6	37	1	42	41	6	36	1	41	121761.4780	-0.0008	0.010	-0.0009	0.50	Melli+22
493:	42	7	35	1	42	41	7	34	1	41	121774.2115	-0.0096	0.010	-0.0096	0.50	Melli+22
494:	42	7	36	1	42	41	7	35	1	41	121774.2115	-0.0096	0.010	-0.0096	0.50	Melli+22
495:	42	3	39	1	42	41	3	38	1	41	121779.1369	-0.0050	0.010			Melli+22
496:	42	8	34	1	42	41	8	33	1	41	121790.6275	-0.0064	0.010	-0.0064	0.50	Melli+22
497:	42	8	35	1	42	41	8	34	1	41	121790.6275	-0.0064	0.010	-0.0064	0.50	Melli+22
498:	42	9	34	1	42	41	9	33	1	41	121810.1880	-0.0071	0.010	-0.0072	0.50	Melli+22
499:	42	9	33	1	42	41	9	32	1	41	121810.1880	-0.0071	0.010	-0.0072	0.50	Melli+22
500:	42	10	32	1	42	41	10	31	1	41	121832.6310	-0.0017	0.010	-0.0018	0.50	Melli+22
501:	42	10	33	1	42	41	10	32	1	41	121832.6310	-0.0017	0.010	-0.0018	0.50	Melli+22
502:	42	11	32	1	42	41	11	31	1	41	121857.7895	-0.0018	0.010	-0.0018	0.50	Melli+22
503:	42	11	31	1	42	41	11	30	1	41	121857.7895	-0.0018	0.010	-0.0018	0.50	Melli+22
504:	42	12	30	1	42	41	12	29	1	41	121885.5851	0.0100	0.010	0.0101	0.50	Melli+22
505:	42	12	31	1	42	41	12	30	1	41	121885.5851	0.0100	0.010	0.0101	0.50	Melli+22
506:	42	13	30	1	42	41	13	29	1	41	121915.9470	0.0265	0.010	0.0265	0.50	Melli+22
507:	42	13	29	1	42	41	13	28	1	41	121915.9470	0.0265	0.010	0.0265	0.50	Melli+22
508:	42	14	28	1	42	41	14	27	1	41	121948.7910	0.0081	0.010	0.0081	0.50	Melli+22
509:	42	14	29	1	42	41	14	28	1	41	121948.7910	0.0081	0.010	0.0081	0.50	Melli+22
510:	42	15	27	1	42	41	15	26	1	41	121984.1190	-0.0094	0.010	-0.0094	0.50	Melli+22
511:	42	15	28	1	42	41	15	27	1	41	121984.1190	-0.0094	0.010	-0.0094	0.50	Melli+22
512:	42	16	27	1	42	41	16	26	1	41	122021.9397	0.0096	0.010	0.0096	0.50	Melli+22
513:	42	16	26	1	42	41	16	25	1	41	122021.9397	0.0096	0.010	0.0096	0.50	Melli+22
514:	42	2	40	1	42	41	2	39	1	41	122052.5727	0.0011	0.010			Melli+22
515/	42	17	26	1	42	41	17	25	1	41	122062.1317	-0.0332	0.010			Melli+22
516/	42	17	25	1	42	41	17	24	1	41	122062.1340	-0.0309	0.010			Melli+22
517:	42	1	41	1	42	41	1	40	1	41	122549.8270	-0.0057	0.010			Melli+22
518:	43	1	43	1	43	42	1	42	1	42	123510.8789	-0.0054	0.010			Melli+22
519:	87	6	81	1	87	86	6	80	1	86	252108.0740	-0.0145	0.030	0.0064	0.50	Melli+22
520:	87	6	82	1	87	86	6	81	1	86	252108.0740	0.0273	0.030	0.0064	0.50	Melli+22
521:	87	7	80	1	87	86	7	79	1	86	252111.6220	-0.0098	0.030	-0.0095	0.50	Melli+22
522:	87	7	81	1	87	86	7	80	1	86	252111.6220	-0.0091	0.030	-0.0095	0.50	Melli+22
523:	88	1	88	1	88	87	1	87	1	87	252115.9960	0.0524	0.030			Melli+22
524:	87	3	85	1	87	86	3	84	1	86	252119.6510	0.0028	0.030			Melli+22
525:	87	5	83	1	87	86	5	82	1	86	252129.4500	-0.0370	0.030			Melli+22
526:	87	8	79	1	87	86	8	78	1	86	252130.9890	0.0497	0.030	0.0498	0.50	Melli+22
527:	87	8	80	1	87	86	8	79	1	86	252130.9890	0.0497	0.030	0.0498	0.50	Melli+22
528:	87	9	78	1	87	86	9	77	1	86	252161.3920	0.0109	0.030	0.0109	0.50	Melli+22
529:	87	9	79	1	87	86	9	78	1	86	252161.3920	0.0109	0.030	0.0109	0.50	Melli+22
530:	87	4	84	1	87	86	4	83	1	86	252182.1550	-0.0056	0.030			Melli+22
531:	87	10	78	1	87	86	10	77	1	86	252200.5610	-0.0309	0.030	-0.0309	0.50	Melli+22
532:	87	10	77	1	87	86	10	76	1	86	252200.5610	-0.0309	0.030	-0.0309	0.50	Melli+22
533:	87	4	83	1	87	86	4	82	1	86	252229.1900	0.0353	0.030			Melli+22
534:	87	11	76	1	87	86	11	75	1	86	252247.2160	-0.0317	0.030	-0.0318	0.50	Melli+22
535:	87	11	77	1	87	86	11	76	1	86	252247.2160	-0.0317	0.030	-0.0318	0.50	Melli+22
536:	87	12	75	1	87	86	12	74	1	86	252300.5210	-0.0338	0.030	-0.0339	0.50	Melli+22
537:	87	12	76	1	87	86	12	75	1	86	252300.5210	-0.0338	0.030	-0.0339	0.50	Melli+22
538:	87	13	75	1	87	86	13	74	1	86	252360.0030	-0.0070	0.030	-0.0070	0.50	Melli+22
539:	87	13	74	1	87	86	13	73	1	86	252360.0030	-0.0070	0.030	-0.0070	0.50	Melli+22
540:	88	0	88	1	88	87	0	87	1	87	252363.6260	-0.0259	0.030			Melli+22
541:	87	15	72	1	87	86	15	71	1	86	252496.0730	-0.0522	0.030	-0.0523	0.50	Melli+22
542:	87	15	73	1	87	86	15	72	1	86	252496.0730	-0.0522	0.030	-0.0523	0.50	Melli+22
543:	87	1	86	1	87	86	1	85	1	86	252622.0430	0.0353	0.030			Melli+22
544:	87	3	84	1	87	86	3	83	1	86	252718.9740	-0.0519	0.030			Melli+22
545:	87	2	85	1	87	86	2	84	1	86	253638.8230	-0.0115	0.030			Melli+22

546:	88	2	87	1	88	87	2	86	1	87	254172.5850	-0.0127	0.030			Melli+22
547:	89	1	89	1	89	88	1	88	1	88	254965.2810	0.0418	0.030			Melli+22
548:	88	6	82	1	88	87	6	81	1	87	255002.4740	0.0143	0.030	0.0380	0.50	Melli+22
549:	88	6	83	1	88	87	6	82	1	87	255002.4740	0.0617	0.030	0.0380	0.50	Melli+22
550:	88	7	81	1	88	87	7	80	1	87	255005.3160	-0.0322	0.030	-0.0319	0.50	Melli+22
551:	88	7	82	1	88	87	7	81	1	87	255005.3160	-0.0315	0.030	-0.0319	0.50	Melli+22
552:	88	3	86	1	88	87	3	85	1	87	255010.7280	-0.0116	0.030			Melli+22
553:	88	8	80	1	88	87	8	79	1	87	255024.4140	-0.0196	0.030	-0.0196	0.50	Melli+22
554:	88	8	81	1	88	87	8	80	1	87	255024.4140	-0.0196	0.030	-0.0196	0.50	Melli+22
555:	88	5	84	1	88	87	5	83	1	87	255025.2630	0.0567	0.030			Melli+22
556:	88	5	83	1	88	87	5	82	1	87	255027.2230	0.0218	0.030			Melli+22
557:	88	9	79	1	88	87	9	78	1	87	255054.8810	-0.0404	0.030	-0.0404	0.50	Melli+22
558:	88	9	80	1	88	87	9	79	1	87	255054.8810	-0.0404	0.030	-0.0404	0.50	Melli+22
559:	88	4	85	1	88	87	4	84	1	87	255079.3610	-0.0027	0.030			Melli+22
560:	88	10	78	1	88	87	10	77	1	87	255094.3590	-0.0049	0.030	-0.0050	0.50	Melli+22
561:	88	10	79	1	88	87	10	78	1	87	255094.3590	-0.0049	0.030	-0.0050	0.50	Melli+22
562:	88	4	84	1	88	87	4	83	1	87	255130.1270	-0.0161	0.030			Melli+22
563:	88	11	78	1	88	87	11	77	1	87	255141.4010	0.0093	0.030	0.0094	0.50	Melli+22
564:	88	11	77	1	88	87	11	76	1	87	255141.4010	0.0093	0.030	0.0094	0.50	Melli+22
565:	88	12	77	1	88	87	12	76	1	87	255195.2020	0.0184	0.030	0.0184	0.50	Melli+22
566:	88	12	76	1	88	87	12	75	1	87	255195.2020	0.0184	0.030	0.0184	0.50	Melli+22
567:	89	0	89	1	89	88	0	88	1	88	255203.8040	0.0160	0.030			Melli+22
568:	88	13	76	1	88	87	13	75	1	87	255255.2470	0.0274	0.030	0.0274	0.50	Melli+22
569:	88	13	75	1	88	87	13	74	1	87	255255.2470	0.0274	0.030	0.0274	0.50	Melli+22
570:	88	14	75	1	88	87	14	74	1	87	255321.2120	0.0587	0.030	0.0587	0.50	Melli+22
571:	88	14	74	1	88	87	14	73	1	87	255321.2120	0.0587	0.030	0.0587	0.50	Melli+22
572:	88	16	72	1	88	87	16	71	1	87	255469.7860	-0.0292	0.030	-0.0293	0.50	Melli+22
573:	88	16	73	1	88	87	16	72	1	87	255469.7860	-0.0292	0.030	-0.0293	0.50	Melli+22
574:	88	1	87	1	88	87	1	86	1	87	255485.4080	-0.0126	0.030			Melli+22
575:	88	3	85	1	88	87	3	84	1	87	255640.8630	0.0828	0.030			Melli+22
576:	88	2	86	1	88	87	2	85	1	87	256553.7260	0.0116	0.030			Melli+22
577:	89	2	88	1	89	88	2	87	1	88	257041.3560	-0.0365	0.030			Melli+22
578:	90	1	90	1	90	89	1	89	1	89	257814.1920	-0.0001	0.030			Melli+22
579:	89	6	83	1	89	88	6	82	1	88	257896.6670	-0.0465	0.030	-0.0197	0.50	Melli+22
580:	89	6	84	1	89	88	6	83	1	88	257896.6670	0.0070	0.030	-0.0197	0.50	Melli+22
581:	89	7	82	1	89	88	7	81	1	88	257898.9530	0.0295	0.030	0.0300	0.50	Melli+22
582:	89	7	83	1	89	88	7	82	1	88	257898.9530	0.0304	0.030	0.0300	0.50	Melli+22
583:	89	3	87	1	89	88	3	86	1	88	257901.4960	0.0710	0.030			Melli+22
584:	89	8	81	1	89	88	8	80	1	88	257917.7140	-0.0577	0.030	-0.0577	0.50	Melli+22
585:	89	8	82	1	89	88	8	81	1	88	257917.7140	-0.0577	0.030	-0.0577	0.50	Melli+22
586:	89	5	84	1	89	88	5	83	1	88	257923.0300	-0.0157	0.030			Melli+22
587:	89	9	80	1	89	88	9	79	1	88	257948.2770	-0.0182	0.030	-0.0182	0.50	Melli+22
588:	89	9	81	1	89	88	9	80	1	88	257948.2770	-0.0182	0.030	-0.0182	0.50	Melli+22
589:	89	4	86	1	89	88	4	85	1	88	257976.3940	-0.0633	0.030			Melli+22
590:	89	10	79	1	89	88	10	78	1	88	257987.9060	-0.0561	0.030	-0.0561	0.50	Melli+22
591:	89	10	80	1	89	88	10	79	1	88	257987.9060	-0.0561	0.030	-0.0561	0.50	Melli+22
592:	89	4	85	1	89	88	4	84	1	88	258031.2600	-0.0131	0.030			Melli+22
593:	89	11	78	1	89	88	11	77	1	88	258035.3910	0.0349	0.030	0.0349	0.50	Melli+22
594:	89	11	79	1	89	88	11	78	1	88	258035.3910	0.0349	0.030	0.0349	0.50	Melli+22
595:	90	0	90	1	90	89	0	89	1	89	258043.7920	-0.0264	0.030			Melli+22
596:	89	12	78	1	89	88	12	77	1	88	258089.6240	-0.0046	0.030	-0.0046	0.50	Melli+22
597:	89	12	77	1	89	88	12	76	1	88	258089.6240	-0.0046	0.030	-0.0046	0.50	Melli+22
598:	89	13	77	1	89	88	13	76	1	88	258150.2210	-0.0210	0.030	-0.0210	0.50	Melli+22
599:	89	13	76	1	89	88	13	75	1	88	258150.2210	-0.0210	0.030	-0.0210	0.50	Melli+22
600:	89	1	88	1	89	88	1	87	1	88	258347.4640	-0.0066	0.030			Melli+22
601:	89	16	73	1	89	88	16	72	1	88	258367.0620	0.0082	0.030	0.0083	0.50	Melli+22
602:	89	16	74	1	89	88	16	73	1	88	258367.0620	0.0082	0.030	0.0083	0.50	Melli+22
603:	89	3	86	1	89	88	3	85	1	88	258563.0650	-0.0342	0.030			Melli+22
604:	89	2	87	1	89	88	2	86	1	88	259467.6760	0.0130	0.030			Melli+22
605:	90	2	89	1	90	89	2	88	1	89	259909.5480	-0.0494	0.030			Melli+22
606:	91	1	91	1	91	90	1	90	1	90	260662.7760	-0.0288	0.030			Melli+22
607:	90	6	84	1	90	89	6	83	1	89	260790.7820	-0.0668	0.030	-0.0366	0.50	Melli+22
608:	90	6	85	1	90	89	6	84	1	89	260790.7820	-0.0063	0.030	-0.0366	0.50	Melli+22
609:	90	3	88	1	90	89	3	87	1	89	260791.6580	-0.0359	0.030			Melli+22
610:	90	7	83	1	90	89	7	82	1	89	260792.3430	-0.0127	0.030	-0.0122	0.50	Melli+22
611:	90	7	84	1	90	89	7	83	1	89	260792.3430	-0.0117	0.030	-0.0122	0.50	Melli+22
612:	90	8	82	1	90	89	8	81	1	89	260810.9640	0.0122	0.030	0.0123	0.50	Melli+22
613:	90	8	83	1	90	89	8	82	1	89	260810.9640	0.0122	0.030	0.0123	0.50	Melli+22
614:	90	5	86	1	90	89	5	85	1	89	260816.3800	-0.0092	0.030			Melli+22

615:	90 5 85 1 90	89 5 84 1 89	260818.8710	0.0476	0.030			Melli+22
616:	90 4 87 1 90	89 4 86 1 89	260873.4660	0.0300	0.030			Melli+22
617:	90 10 81 1 90	89 10 80 1 89	260881.4010	0.0166	0.030	0.0166	0.50	Melli+22
618:	90 10 80 1 90	89 10 79 1 89	260881.4010	0.0166	0.030	0.0166	0.50	Melli+22
619:	91 0 91 1 91	90 0 90 1 90	260883.7710	0.0233	0.030			Melli+22
620:	90 11 80 1 90	89 11 79 1 89	260929.1900	0.0509	0.030	0.0509	0.50	Melli+22
621:	90 11 79 1 90	89 11 78 1 89	260929.1900	0.0509	0.030	0.0509	0.50	Melli+22
622:	90 4 86 1 90	89 4 85 1 89	260932.6110	0.0589	0.030			Melli+22
623:	90 12 79 1 90	89 12 78 1 89	260983.9200	0.0321	0.030	0.0322	0.50	Melli+22
624:	90 12 78 1 90	89 12 77 1 89	260983.9200	0.0321	0.030	0.0322	0.50	Melli+22
625:	90 13 77 1 90	89 13 76 1 89	261045.1090	0.0338	0.030	0.0338	0.50	Melli+22
626:	90 13 78 1 90	89 13 77 1 89	261045.1090	0.0338	0.030	0.0338	0.50	Melli+22
627:	90 14 76 1 90	89 14 75 1 89	261112.3300	-0.0016	0.030	-0.0017	0.50	Melli+22
628:	90 14 77 1 90	89 14 76 1 89	261112.3300	-0.0016	0.030	-0.0017	0.50	Melli+22
629:	90 1 89 1 90	89 1 88 1 89	261208.1610	-0.0004	0.030			Melli+22
630:	90 16 74 1 90	89 16 73 1 89	261264.1370	0.0414	0.030	0.0415	0.50	Melli+22
631:	90 16 75 1 90	89 16 74 1 89	261264.1370	0.0414	0.030	0.0415	0.50	Melli+22
632:	90 3 87 1 90	89 3 86 1 89	261485.9300	-0.0401	0.030			Melli+22
633:	90 2 88 1 90	89 2 87 1 89	262380.7170	0.0590	0.030			Melli+22
634:	91 2 90 1 91	90 2 89 1 90	262777.2180	0.0073	0.030			Melli+22
635:	91 3 89 1 91	90 3 88 1 90	263681.5800	0.0432	0.030			Melli+22
636:	91 6 85 1 91	90 6 84 1 90	263684.7990	-0.0653	0.030	-0.0312	0.50	Melli+22
637:	91 6 86 1 91	90 6 85 1 90	263684.7990	0.0029	0.030	-0.0312	0.50	Melli+22
638:	91 8 83 1 91	90 8 82 1 90	263703.9020	-0.0699	0.030	-0.0699	0.50	Melli+22
639:	91 8 84 1 91	90 8 83 1 90	263703.9020	-0.0699	0.030	-0.0699	0.50	Melli+22
640:	91 5 86 1 91	90 5 85 1 90	263714.4820	-0.0522	0.030			Melli+22
641:	92 0 92 1 92	91 0 91 1 91	263723.5850	0.0058	0.030			Melli+22
642:	91 9 82 1 91	90 9 81 1 90	263734.5630	0.0273	0.030	0.0273	0.50	Melli+22
643:	91 9 83 1 91	90 9 82 1 90	263734.5630	0.0273	0.030	0.0273	0.50	Melli+22
644:	91 4 88 1 91	90 4 87 1 90	263770.2340	-0.0599	0.030			Melli+22
645:	91 10 82 1 91	90 10 81 1 90	263774.6060	-0.0228	0.030	-0.0229	0.50	Melli+22
646:	91 10 81 1 91	90 10 80 1 90	263774.6060	-0.0228	0.030	-0.0229	0.50	Melli+22
647:	91 4 87 1 91	90 4 86 1 90	263834.0130	0.0256	0.030			Melli+22
648:	91 12 80 1 91	90 12 79 1 90	263877.9300	-0.0292	0.030	-0.0292	0.50	Melli+22
649:	91 12 79 1 91	90 12 78 1 90	263877.9300	-0.0292	0.030	-0.0292	0.50	Melli+22
650:	91 13 79 1 91	90 13 78 1 90	263939.7040	-0.0129	0.030	-0.0130	0.50	Melli+22
651:	91 13 78 1 91	90 13 77 1 90	263939.7040	-0.0129	0.030	-0.0130	0.50	Melli+22
652:	91 15 76 1 91	90 15 75 1 90	264081.4260	-0.0090	0.030	-0.0090	0.50	Melli+22
653:	91 15 77 1 91	90 15 76 1 90	264081.4260	-0.0090	0.030	-0.0090	0.50	Melli+22
654:	91 17 75 1 91	90 17 74 1 90	264245.9750	-0.0217	0.030	-0.0217	0.50	Melli+22
655:	91 17 74 1 91	90 17 73 1 90	264245.9750	-0.0217	0.030	-0.0217	0.50	Melli+22
656:	91 2 89 1 91	90 2 88 1 90	265292.6590	-0.0186	0.030			Melli+22
657:	92 2 91 1 92	91 2 90 1 91	265644.2110	-0.0193	0.030			Melli+22
658:	93 1 93 1 93	92 1 92 1 92	266359.0610	0.0417	0.030			Melli+22
659:	93 0 93 1 93	92 0 92 1 92	266563.3370	0.0212	0.030			Melli+22
660:	92 6 86 1 92	91 6 85 1 91	266578.7070	-0.0517	0.030	-0.0133	0.50	Melli+22
661:	92 6 87 1 92	91 6 86 1 91	266578.7070	0.0251	0.030	-0.0133	0.50	Melli+22
662:	92 8 84 1 92	91 8 83 1 91	266596.8790	0.0485	0.030	0.0485	0.50	Melli+22
663:	92 8 85 1 92	91 8 84 1 91	266596.8790	0.0485	0.030	0.0485	0.50	Melli+22
664:	92 5 88 1 92	91 5 87 1 91	266607.2050	-0.0169	0.030			Melli+22
665:	92 5 87 1 92	91 5 86 1 91	266610.2170	0.0386	0.030			Melli+22
666:	92 9 83 1 92	91 9 82 1 91	266627.3880	-0.0105	0.030	-0.0106	0.50	Melli+22
667:	92 9 84 1 92	91 9 83 1 91	266627.3880	-0.0105	0.030	-0.0106	0.50	Melli+22
668:	92 12 80 1 92	91 12 79 1 91	266771.8420	0.0012	0.030	0.0012	0.50	Melli+22
669:	92 12 81 1 92	91 12 80 1 91	266771.8420	0.0012	0.030	0.0012	0.50	Melli+22
670:	92 14 79 1 92	91 14 78 1 91	266902.6920	-0.0411	0.030	-0.0411	0.50	Melli+22
671:	92 14 78 1 92	91 14 77 1 91	266902.6920	-0.0411	0.030	-0.0411	0.50	Melli+22
672:	92 1 91 1 92	91 1 90 1 91	266925.4640	-0.0272	0.030			Melli+22
673:	92 15 78 1 92	91 15 77 1 91	266977.2180	-0.0527	0.030	-0.0527	0.50	Melli+22
674:	92 15 77 1 92	91 15 76 1 91	266977.2180	-0.0527	0.030	-0.0527	0.50	Melli+22
675:	92 3 89 1 92	91 3 88 1 91	267333.2530	-0.0513	0.030			Melli+22
676:	93 2 92 1 93	92 2 91 1 92	268510.6860	0.0307	0.030			Melli+22
677:	94 1 94 1 94	93 1 93 1 93	269206.6370	0.0113	0.030			Melli+22
678:	94 0 94 1 94	93 0 93 1 93	269402.9590	-0.0004	0.030			Melli+22
679:	93 3 91 1 93	92 3 90 1 92	269459.9060	0.0014	0.030			Melli+22
680:	93 3 91 1 93	92 3 90 1 92	269459.9240	0.0194	0.030			Melli+22
681:	93 7 86 1 93	92 7 85 1 92	269471.7440	-0.0354	0.030	-0.0346	0.50	Melli+22
682:	93 7 87 1 93	92 7 86 1 92	269471.7440	-0.0338	0.030	-0.0346	0.50	Melli+22
683:	93 8 85 1 93	92 8 84 1 92	269489.4550	-0.0707	0.030	-0.0707	0.50	Melli+22

684:	93 8 86 1 93	92 8 85 1 92	269489.4550	-0.0707	0.030	-0.0707 0.50	Melli+22
685:	93 5 89 1 93	92 5 88 1 92	269502.5340	0.0309	0.030		Melli+22
686:	93 5 88 1 93	92 5 87 1 92	269505.7590	0.0032	0.030		Melli+22
687:	93 9 84 1 93	92 9 83 1 92	269520.1020	0.0145	0.030	0.0146 0.50	Melli+22
688:	93 9 85 1 93	92 9 84 1 92	269520.1020	0.0145	0.030	0.0146 0.50	Melli+22
689:	93 10 83 1 93	92 10 82 1 92	269560.5850	0.0084	0.030	0.0084 0.50	Melli+22
690:	93 10 84 1 93	92 10 83 1 92	269560.5850	0.0084	0.030	0.0084 0.50	Melli+22
691:	93 4 90 1 93	92 4 89 1 92	269563.6670	0.0418	0.030		Melli+22
692:	93 11 83 1 93	92 11 82 1 92	269609.4090	0.0295	0.030	0.0295 0.50	Melli+22
693:	93 11 82 1 93	92 11 81 1 92	269609.4090	0.0295	0.030	0.0295 0.50	Melli+22
694:	93 4 89 1 93	92 4 88 1 92	269637.3900	0.0305	0.030		Melli+22
695:	93 12 81 1 93	92 12 80 1 92	269665.5280	-0.0024	0.030	-0.0024 0.50	Melli+22
696:	93 12 82 1 93	92 12 81 1 92	269665.5280	-0.0024	0.030	-0.0024 0.50	Melli+22
697:	93 13 81 1 93	92 13 80 1 92	269728.4430	0.0247	0.030	0.0247 0.50	Melli+22
698:	93 13 80 1 93	92 13 79 1 92	269728.4430	0.0247	0.030	0.0247 0.50	Melli+22
699:	93 1 92 1 93	92 1 91 1 92	269782.1420	-0.0059	0.030		Melli+22
700:	93 16 77 1 93	92 16 76 1 92	269954.0650	0.0454	0.030	0.0455 0.50	Melli+22
701:	93 16 78 1 93	92 16 77 1 92	269954.0650	0.0454	0.030	0.0455 0.50	Melli+22
702:	93 3 90 1 93	92 3 89 1 92	270257.7020	-0.0290	0.030		Melli+22
703:	93 2 91 1 93	92 2 90 1 92	271113.7270	0.0197	0.030		Melli+22
704:	94 2 93 1 94	93 2 92 1 93	271376.5260	0.0420	0.030		Melli+22
705:	95 1 95 1 95	94 1 94 1 94	272053.9100	0.0087	0.030		Melli+22
706:	95 0 95 1 95	94 0 94 1 94	272242.5180	0.0066	0.030		Melli+22
707:	94 3 92 1 94	93 3 91 1 93	272348.4190	0.0088	0.030		Melli+22
708:	94 7 87 1 94	93 7 86 1 93	272364.6110	-0.0132	0.030	-0.0123 0.50	Melli+22
709:	94 7 88 1 94	93 7 87 1 93	272364.6110	-0.0114	0.030	-0.0123 0.50	Melli+22
710:	94 6 88 1 94	93 6 87 1 93	272366.1230	-0.0560	0.030	-0.0076 0.50	Melli+22
711:	94 6 89 1 94	93 6 88 1 93	272366.1230	0.0409	0.030	-0.0076 0.50	Melli+22
712:	94 8 86 1 94	93 8 85 1 93	272382.0370	-0.0188	0.030	-0.0188 0.50	Melli+22
713:	94 8 87 1 94	93 8 86 1 93	272382.0370	-0.0187	0.030	-0.0188 0.50	Melli+22
714:	94 5 90 1 94	93 5 89 1 93	272397.6870	-0.0049	0.030		Melli+22
715:	94 5 89 1 94	93 5 88 1 93	272401.2570	-0.0098	0.030		Melli+22
716:	94 9 85 1 94	93 9 84 1 93	272412.5940	-0.0064	0.030	-0.0064 0.50	Melli+22
717:	94 9 86 1 94	93 9 85 1 93	272412.5940	-0.0064	0.030	-0.0064 0.50	Melli+22
718:	94 10 85 1 94	93 10 84 1 93	272453.2680	-0.0079	0.030	-0.0080 0.50	Melli+22
719:	94 10 84 1 94	93 10 83 1 93	272453.2680	-0.0079	0.030	-0.0080 0.50	Melli+22
720:	94 4 91 1 94	93 4 90 1 93	272460.0860	-0.0003	0.030		Melli+22
721:	94 11 83 1 94	93 11 82 1 93	272502.4060	-0.0106	0.030		Melli+22
722:	94 4 90 1 94	93 4 89 1 93	272539.3050	-0.0076	0.030		Melli+22
723:	94 12 83 1 94	93 12 82 1 93	272559.0270	0.0009	0.030	0.0009 0.50	Melli+22
724:	94 12 82 1 94	93 12 81 1 93	272559.0270	0.0009	0.030	0.0009 0.50	Melli+22
725:	94 13 81 1 94	93 13 80 1 93	272622.4830	0.0093	0.030	0.0094 0.50	Melli+22
726:	94 13 82 1 94	93 13 81 1 93	272622.4830	0.0093	0.030	0.0094 0.50	Melli+22
727:	94 1 93 1 94	93 1 92 1 93	272637.4800	-0.0012	0.030		Melli+22
728:	94 14 81 1 94	93 14 80 1 93	272692.3040	-0.0367	0.030	-0.0368 0.50	Melli+22
729:	94 14 80 1 94	93 14 79 1 93	272692.3040	-0.0367	0.030	-0.0368 0.50	Melli+22
730:	94 16 79 1 94	93 16 78 1 93	272850.2960	0.0428	0.030	0.0428 0.50	Melli+22
731:	94 16 78 1 94	93 16 77 1 93	272850.2960	0.0428	0.030	0.0428 0.50	Melli+22
732:	94 3 91 1 94	93 3 90 1 93	273182.6360	-0.0004	0.030		Melli+22
733:	94 2 92 1 94	93 2 91 1 93	274022.6610	-0.0155	0.030		Melli+22
734:	95 2 94 1 95	94 2 93 1 94	274241.7240	0.0086	0.030		Melli+22
735:	96 1 96 1 96	95 1 95 1 95	274900.8550	0.0066	0.030		Melli+22
736:	96 0 96 1 96	95 0 95 1 95	275081.9840	0.0116	0.030		Melli+22
737:	95 3 93 1 95	94 3 92 1 94	275236.4100	-0.0405	0.030		Melli+22
738:	95 7 88 1 95	94 7 87 1 94	275257.3110	-0.0071	0.030	-0.0061 0.50	Melli+22
739:	95 7 89 1 95	94 7 88 1 94	275257.3110	-0.0051	0.030	-0.0061 0.50	Melli+22
740:	95 6 89 1 95	94 6 88 1 94	275259.6400	-0.0625	0.030	-0.0081 0.50	Melli+22
741:	95 6 90 1 95	94 6 89 1 94	275259.6400	0.0463	0.030	-0.0081 0.50	Melli+22
742:	95 5 91 1 95	94 5 90 1 94	275292.7810	-0.0060	0.030		Melli+22
743:	95 5 90 1 95	94 5 89 1 94	275296.7260	0.0141	0.030		Melli+22
744:	95 9 86 1 95	94 9 85 1 94	275304.9440	0.0084	0.030	0.0084 0.50	Melli+22
745:	95 9 87 1 95	94 9 86 1 94	275304.9440	0.0084	0.030	0.0084 0.50	Melli+22
746:	95 10 85 1 95	94 10 84 1 94	275345.7970	0.0072	0.030	0.0073 0.50	Melli+22
747:	95 10 86 1 95	94 10 85 1 94	275345.7970	0.0072	0.030	0.0073 0.50	Melli+22
748:	95 4 92 1 95	94 4 91 1 94	275356.4390	0.0359	0.030		Melli+22
749:	95 4 91 1 95	94 4 90 1 94	275441.4650	0.0096	0.030		Melli+22
750:	95 12 83 1 95	94 12 82 1 94	275452.3270	0.0012	0.030	0.0013 0.50	Melli+22
751:	95 12 84 1 95	94 12 83 1 94	275452.3270	0.0012	0.030	0.0013 0.50	Melli+22
752:	95 1 94 1 95	94 1 93 1 94	275491.4750	-0.0299	0.030		Melli+22

753:	95	13	83	1	95	94	13	82	1	94	275516.2990	-0.0302	0.030	-0.0303	0.50	Melli+22
754:	95	13	82	1	95	94	13	81	1	94	275516.2990	-0.0302	0.030	-0.0303	0.50	Melli+22
755:	95	14	81	1	95	94	14	80	1	94	275586.7920	-0.0496	0.030	-0.0496	0.50	Melli+22
756:	95	14	82	1	95	94	14	81	1	94	275586.7920	-0.0496	0.030	-0.0496	0.50	Melli+22
757:	95	3	92	1	95	94	3	91	1	94	276107.9670	-0.0298	0.030			Melli+22
758:	95	2	93	1	95	94	2	92	1	94	276930.6250	0.0357	0.030			Melli+22
759:	96	2	95	1	96	95	2	94	1	95	277106.3360	-0.0125	0.030			Melli+22
760:	97	1	97	1	97	96	1	96	1	96	277747.4790	0.0099	0.030			Melli+22
761:	97	0	97	1	97	96	0	96	1	96	277921.3390	-0.0035	0.030			Melli+22
762:	96	3	94	1	96	95	3	93	1	95	278124.0000	-0.0164	0.030			Melli+22
763:	96	6	90	1	96	95	6	89	1	95	278153.0430	-0.0568	0.030	0.0041	0.50	Melli+22
764:	96	6	91	1	96	95	6	90	1	95	278153.0430	0.0651	0.030	0.0041	0.50	Melli+22
765:	96	8	88	1	96	95	8	87	1	95	278166.5910	-0.0225	0.030	-0.0225	0.50	Melli+22
766:	96	8	89	1	96	95	8	88	1	95	278166.5910	-0.0225	0.030	-0.0225	0.50	Melli+22
767:	96	5	92	1	96	95	5	91	1	95	278187.7690	-0.0174	0.030			Melli+22
768:	96	5	91	1	96	95	5	90	1	95	278192.0870	-0.0040	0.030			Melli+22
769:	96	9	87	1	96	95	9	86	1	95	278197.1010	0.0098	0.030	0.0099	0.50	Melli+22
770:	96	9	88	1	96	95	9	87	1	95	278197.1010	0.0098	0.030	0.0099	0.50	Melli+22
771:	96	10	86	1	96	95	10	85	1	95	278238.1090	-0.0069	0.030	-0.0070	0.50	Melli+22
772:	96	10	87	1	96	95	10	86	1	95	278238.1090	-0.0069	0.030	-0.0070	0.50	Melli+22
773:	96	4	93	1	96	95	4	92	1	95	278252.5630	-0.0057	0.030			Melli+22
774:	96	11	86	1	96	95	11	85	1	95	278287.9380	0.0232	0.030	0.0232	0.50	Melli+22
775:	96	11	85	1	96	95	11	84	1	95	278287.9380	0.0232	0.030	0.0232	0.50	Melli+22
776:	96	12	85	1	96	95	12	84	1	95	278345.4050	-0.0223	0.030	-0.0223	0.50	Melli+22
777:	96	12	84	1	96	95	12	83	1	95	278345.4050	-0.0223	0.030	-0.0223	0.50	Melli+22
778:	96	13	83	1	96	95	13	82	1	95	278409.9770	-0.0062	0.030	-0.0062	0.50	Melli+22
779:	96	13	84	1	96	95	13	83	1	95	278409.9770	-0.0062	0.030	-0.0062	0.50	Melli+22
780:	96	17	79	1	96	95	17	78	1	95	278731.5120	0.0007	0.030	0.0007	0.50	Melli+22
781:	96	17	80	1	96	95	17	79	1	95	278731.5120	0.0007	0.030	0.0007	0.50	Melli+22
782:	96	3	93	1	96	95	3	92	1	95	279033.7900	0.0030	0.030			Melli+22
783:	96	2	94	1	96	95	2	93	1	95	279837.4250	-0.0013	0.030			Melli+22
784:	97	2	96	1	97	96	2	95	1	96	279970.3940	0.0111	0.030			Melli+22
785:	98	1	98	1	98	97	1	97	1	97	280593.7480	-0.0175	0.030			Melli+22
786:	98	0	98	1	98	97	0	97	1	97	280760.6380	0.0166	0.030			Melli+22
787:	97	3	95	1	97	96	3	94	1	96	281011.1170	0.0185	0.030			Melli+22
788:	97	8	89	1	97	96	8	88	1	96	281058.6010	-0.0366	0.030	-0.0367	0.50	Melli+22
789:	97	8	90	1	97	96	8	89	1	96	281058.6010	-0.0366	0.030	-0.0367	0.50	Melli+22
790:	97	10	88	1	97	96	10	87	1	96	281130.2320	-0.0207	0.030	-0.0207	0.50	Melli+22
791:	97	10	87	1	97	96	10	86	1	96	281130.2320	-0.0207	0.030	-0.0207	0.50	Melli+22
792:	97	4	94	1	97	96	4	93	1	96	281148.5980	0.0210	0.030			Melli+22
793:	97	11	87	1	97	96	11	86	1	96	281180.3940	0.0223	0.030	0.0223	0.50	Melli+22
794:	97	11	86	1	97	96	11	85	1	96	281180.3940	0.0223	0.030	0.0223	0.50	Melli+22
795:	97	1	96	1	97	96	1	95	1	96	281195.6880	-0.0015	0.030			Melli+22
796:	97	4	93	1	97	96	4	92	1	96	281246.3540	0.0093	0.030			Melli+22
797:	97	14	83	1	97	96	14	82	1	96	281375.2350	0.0080	0.030	0.0081	0.50	Melli+22
798:	97	14	84	1	97	96	14	83	1	96	281375.2350	0.0080	0.030	0.0081	0.50	Melli+22
799:	97	16	82	1	97	96	16	81	1	96	281537.7320	0.0309	0.030	0.0309	0.50	Melli+22
800:	97	16	81	1	97	96	16	80	1	96	281537.7320	0.0309	0.030	0.0309	0.50	Melli+22
801:	97	3	94	1	97	96	3	93	1	96	281959.9710	-0.0081	0.030			Melli+22
802:	97	2	95	1	97	96	2	94	1	96	282743.1730	0.0042	0.030			Melli+22
803:	98	2	97	1	98	97	2	96	1	97	282833.8240	0.0064	0.030			Melli+22
804:	99	1	99	1	99	98	1	98	1	98	283439.7500	0.0101	0.030			Melli+22
805:	99	0	99	1	99	98	0	98	1	98	283599.8100	0.0018	0.030			Melli+22
806:	98	3	96	1	98	97	3	95	1	97	283897.6980	0.0106	0.030			Melli+22
807:	98	7	91	1	98	97	7	90	1	97	283934.4930	0.0141	0.030	0.0157	0.50	Melli+22
808:	98	7	92	1	98	97	7	91	1	97	283934.4930	0.0171	0.030	0.0157	0.50	Melli+22
809:	98	8	90	1	98	97	8	89	1	97	283950.4680	-0.0216	0.030	-0.0216	0.50	Melli+22
810:	98	8	91	1	98	97	8	90	1	97	283950.4680	-0.0215	0.030	-0.0216	0.50	Melli+22
811:	98	5	94	1	98	97	5	93	1	97	283977.4900	-0.0018	0.030			Melli+22
812:	98	9	89	1	98	97	9	88	1	97	283980.8450	-0.0108	0.030	-0.0108	0.50	Melli+22
813:	98	9	90	1	98	97	9	89	1	97	283980.8450	-0.0108	0.030	-0.0108	0.50	Melli+22
814:	98	5	93	1	98	97	5	92	1	97	283982.6650	0.0112	0.030			Melli+22
815:	98	10	89	1	98	97	10	88	1	97	284022.1850	-0.0130	0.030	-0.0131	0.50	Melli+22
816:	98	10	88	1	98	97	10	87	1	97	284022.1850	-0.0130	0.030	-0.0131	0.50	Melli+22
817:	98	4	95	1	98	97	4	94	1	97	284044.4260	0.0051	0.030			Melli+22
818:	98	1	97	1	98	97	1	96	1	97	284045.8760	-0.0122	0.030			Melli+22
819:	98	11	87	1	98	97	11	86	1	97	284072.6200	-0.0110	0.030	-0.0111	0.50	Melli+22
820:	98	11	88	1	98	97	11	87	1	97	284072.6200	-0.0110	0.030	-0.0111	0.50	Melli+22
821:	98	12	86	1	98	97	12	85	1	97	284131.0300	0.0017	0.030	0.0018	0.50	Melli+22

822: 98 12 87	1 98	97 12 86	1 97	284131.0300	0.0017	0.030	0.0018	0.50	Melli+22
823: 98 4 94	1 98	97 4 93	1 97	284149.1100	0.0004	0.030			Melli+22
824: 98 13 86	1 98	97 13 85	1 97	284196.6910	0.0134	0.030	0.0134	0.50	Melli+22
825: 98 13 85	1 98	97 13 84	1 97	284196.6910	0.0134	0.030	0.0134	0.50	Melli+22
826: 98 14 85	1 98	97 14 84	1 97	284269.0750	-0.0321	0.030	-0.0322	0.50	Melli+22
827: 98 14 84	1 98	97 14 83	1 97	284269.0750	-0.0321	0.030	-0.0322	0.50	Melli+22
828: 98 16 82	1 98	97 16 81	1 97	284433.1330	0.0408	0.030			Melli+22
829: 98 3 95	1 98	97 3 94	1 97	284886.5440	0.0002	0.030			Melli+22
830: 98 2 96	1 98	97 2 95	1 97	285647.7940	-0.0042	0.030			Melli+22
831: 99 2 98	1 99	98 2 97	1 98	285696.6600	0.0075	0.030			Melli+22
832:100 1100	1100	99 1 99	1 99	286285.4020	0.0078	0.030			Melli+22
833:100 0100	1100	99 0 99	1 99	286438.9070	0.0056	0.030			Melli+22
834: 99 3 97	1 99	98 3 96	1 98	286783.7900	0.0160	0.030			Melli+22
835: 99 7 92	1 99	98 7 91	1 98	286826.5540	0.0006	0.030	0.0023	0.50	Melli+22
836: 99 7 93	1 99	98 7 92	1 98	286826.5540	0.0040	0.030	0.0023	0.50	Melli+22
837: 99 8 91	1 99	98 8 90	1 98	286842.1510	-0.0166	0.030	-0.0166	0.50	Melli+22
838: 99 8 92	1 99	98 8 91	1 98	286842.1510	-0.0165	0.030	-0.0166	0.50	Melli+22
839: 99 5 94	1 99	98 5 93	1 98	286877.8640	0.0256	0.030			Melli+22
840: 99 1 98	1 99	98 1 97	1 98	286894.8630	0.0101	0.030			Melli+22
841: 99 10 89	1 99	98 10 88	1 98	286913.9440	-0.0060	0.030	-0.0061	0.50	Melli+22
842: 99 10 90	1 99	98 10 89	1 98	286913.9440	-0.0060	0.030	-0.0061	0.50	Melli+22
843: 99 4 96	1 99	98 4 95	1 98	286940.0990	0.0053	0.030			Melli+22
844: 99 11 88	1 99	98 11 87	1 98	286964.6820	-0.0090	0.030	-0.0091	0.50	Melli+22
845: 99 11 89	1 99	98 11 88	1 98	286964.6820	-0.0090	0.030	-0.0091	0.50	Melli+22
846: 99 12 88	1 99	98 12 87	1 98	287023.5200	-0.0034	0.030	-0.0034	0.50	Melli+22
847: 99 12 87	1 99	98 12 86	1 98	287023.5200	-0.0034	0.030	-0.0034	0.50	Melli+22
848: 99 4 95	1 99	98 4 94	1 98	287052.0580	-0.0424	0.030			Melli+22
849: 99 13 86	1 99	98 13 85	1 98	287089.7480	0.0341	0.030	0.0341	0.50	Melli+22
850: 99 13 87	1 99	98 13 86	1 98	287089.7480	0.0341	0.030	0.0341	0.50	Melli+22
851: 99 14 86	1 99	98 14 85	1 98	287162.8130	0.0366	0.030	0.0367	0.50	Melli+22
852: 99 14 85	1 99	98 14 84	1 98	287162.8130	0.0366	0.030	0.0367	0.50	Melli+22
853: 99 17 82	1 99	98 17 81	1 98	287420.2750	0.0021	0.030	0.0021	0.50	Melli+22
854: 99 17 83	1 99	98 17 82	1 98	287420.2750	0.0021	0.030	0.0021	0.50	Melli+22
855: 99 3 96	1 99	98 3 95	1 98	287813.4390	-0.0104	0.030			Melli+22
856: 99 2 97	1 99	98 2 96	1 98	288551.2920	-0.0044	0.030			Melli+22
857:100 2 99	1100	99 2 98	1 99	288558.9100	0.0226	0.030			Melli+22
858:101 1101	1101	100 1100	1100	289130.7790	0.0486	0.030			Melli+22
859:101 0101	1101	100 0100	1100	289277.8980	-0.0014	0.030			Melli+22
860:100 3 98	1100	99 3 97	1 99	289669.3500	0.0006	0.030			Melli+22
861:100 7 93	1100	99 7 92	1 99	289718.5060	0.0368	0.030	0.0388	0.50	Melli+22
862:100 7 94	1100	99 7 93	1 99	289718.5060	0.0407	0.030	0.0388	0.50	Melli+22
863:100 6 94	1100	99 6 93	1 99	289725.3540	-0.0488	0.030			Melli+22
864:100 8 92	1100	99 8 91	1 99	289733.7020	0.0320	0.030	0.0321	0.50	Melli+22
865:100 8 93	1100	99 8 92	1 99	289733.7020	0.0320	0.030	0.0321	0.50	Melli+22
866:100 1 99	1100	99 1 98	1 99	289742.6290	0.0220	0.030			Melli+22
867:100 9 91	1100	99 9 90	1 99	289763.8550	-0.0245	0.030	-0.0245	0.50	Melli+22
868:100 9 92	1100	99 9 91	1 99	289763.8550	-0.0245	0.030	-0.0245	0.50	Melli+22
869:100 5 96	1100	99 5 95	1 99	289766.7680	-0.0256	0.030			Melli+22
870:100 5 95	1100	99 5 94	1 99	289773.0250	0.0656	0.030			Melli+22
871:100 10 91	1100	99 10 90	1 99	289805.4850	-0.0217	0.030	-0.0217	0.50	Melli+22
872:100 10 90	1100	99 10 89	1 99	289805.4850	-0.0217	0.030	-0.0217	0.50	Melli+22
873:100 4 97	1100	99 4 96	1 99	289835.6370	0.0488	0.030			Melli+22
874:100 11 89	1100	99 11 88	1 99	289856.5320	-0.0176	0.030	-0.0176	0.50	Melli+22
875:100 11 90	1100	99 11 89	1 99	289856.5320	-0.0176	0.030	-0.0176	0.50	Melli+22
876:100 12 89	1100	99 12 88	1 99	289915.8630	0.0505	0.030	0.0505	0.50	Melli+22
877:100 12 88	1100	99 12 87	1 99	289915.8630	0.0505	0.030	0.0505	0.50	Melli+22
878:100 4 96	1100	99 4 95	1 99	289955.3680	0.0411	0.030			Melli+22
879:100 13 87	1100	99 13 86	1 99	289982.4840	-0.0561	0.030	-0.0561	0.50	Melli+22
880:100 13 88	1100	99 13 87	1 99	289982.4840	-0.0561	0.030	-0.0561	0.50	Melli+22
881:100 14 87	1100	99 14 86	1 99	290056.2040	-0.0283	0.030	-0.0283	0.50	Melli+22
882:100 14 86	1100	99 14 85	1 99	290056.2040	-0.0283	0.030	-0.0283	0.50	Melli+22
883:100 16 84	1100	99 16 83	1 99	290223.2740	0.0499	0.030	0.0499	0.50	Melli+22
884:100 16 85	1100	99 16 84	1 99	290223.2740	0.0499	0.030	0.0499	0.50	Melli+22
885:100 17 84	1100	99 17 83	1 99	290316.0510	-0.0382	0.030			Melli+22
886:100 17 83	1100	99 17 82	1 99	290316.1270	0.0377	0.030			Melli+22
887:100 3 97	1100	99 3 96	1 99	290740.6350	-0.0278	0.030			Melli+22
888:100 3 97	1100	99 3 96	1 99	290740.6630	0.0001	0.030			Melli+22
889:100 2 98	1100	99 2 97	1 99	291453.6390	-0.0066	0.030			Melli+22
890:102 1102	1102	101 1101	1101	291975.7340	-0.0163	0.030			Melli+22

891:102	0102	1102	101	0101	1101	292116.8580	0.0577	0.030			Melli+22
892:101	3 99	1101	100	3 98	1100	292554.3370	-0.0675	0.030			Melli+22
893:101	7 94	1101	100	7 93	1100	292610.1640	-0.0604	0.030	-0.0582	0.50	Melli+22
894:101	7 95	1101	100	7 94	1100	292610.1640	-0.0559	0.030	-0.0582	0.50	Melli+22
895:101	9 92	1101	100	9 91	1100	292655.1230	0.0141	0.030	0.0141	0.50	Melli+22
896:101	9 93	1101	100	9 92	1100	292655.1230	0.0141	0.030	0.0141	0.50	Melli+22
897:101	5 97	1101	100	5 96	1100	292661.2800	-0.0084	0.030			Melli+22
898:101	5 96	1101	100	5 95	1100	292667.9960	-0.0215	0.030			Melli+22
899:101	10 91	1101	100	10 90	1100	292696.8780	0.0118	0.030	0.0118	0.50	Melli+22
900:101	10 92	1101	100	10 91	1100	292696.8780	0.0118	0.030	0.0118	0.50	Melli+22
901:101	4 98	1101	100	4 97	1100	292730.8810	-0.0163	0.030			Melli+22
902:101	11 90	1101	100	11 89	1100	292748.1620	-0.0427	0.030	-0.0428	0.50	Melli+22
903:101	11 91	1101	100	11 90	1100	292748.1620	-0.0427	0.030	-0.0428	0.50	Melli+22
904:101	13 89	1101	100	13 88	1100	292875.1400	-0.0142	0.030	-0.0143	0.50	Melli+22
905:101	13 88	1101	100	13 87	1100	292875.1400	-0.0142	0.030	-0.0143	0.50	Melli+22
906:101	3 98	1101	100	3 97	1100	293668.1260	-0.0229	0.030			Melli+22
907:101	2 99	1101	100	2 98	1100	294354.8770	0.0489	0.030			Melli+22
908:103	1103	1103	102	1102	1102	294820.4620	0.0060	0.030			Melli+22
909:103	0103	1103	102	0102	1102	294955.5700	-0.0314	0.030			Melli+22
910:102	1101	1102	101	1100	1101	295434.5890	0.0029	0.030			Melli+22
911:102	3100	1102	101	3 99	1101	295438.9290	-0.0019	0.030			Melli+22
912:102	7 95	1102	101	7 94	1101	295501.8250	0.0072	0.030	0.0098	0.50	Melli+22
913:102	7 96	1102	101	7 95	1101	295501.8250	0.0123	0.030	0.0098	0.50	Melli+22
914:102	6 97	1102	101	6 96	1101	295510.5710	0.0408	0.030			Melli+22
915:102	6 96	1102	101	6 95	1101	295510.7110	-0.0544	0.030			Melli+22
916:102	8 94	1102	101	8 93	1101	295516.1570	0.0164	0.030	0.0165	0.50	Melli+22
917:102	8 95	1102	101	8 94	1101	295516.1570	0.0165	0.030	0.0165	0.50	Melli+22
918:102	9 93	1102	101	9 92	1101	295546.0980	-0.0493	0.030	-0.0494	0.50	Melli+22
919:102	9 94	1102	101	9 93	1101	295546.0980	-0.0493	0.030	-0.0494	0.50	Melli+22
920:102	10 92	1102	101	10 91	1101	295588.0500	0.0235	0.030	0.0235	0.50	Melli+22
921:102	10 93	1102	101	10 92	1101	295588.0500	0.0235	0.030	0.0235	0.50	Melli+22
922:102	4 99	1102	101	4 98	1101	295626.0310	0.0174	0.030			Melli+22
923:102	12 90	1102	101	12 89	1101	295699.7330	-0.0308	0.030	-0.0308	0.50	Melli+22
924:102	12 91	1102	101	12 90	1101	295699.7330	-0.0308	0.030	-0.0308	0.50	Melli+22
925:102	4 98	1102	101	4 97	1101	295762.4830	-0.0428	0.030			Melli+22
926:102	13 90	1102	101	13 89	1101	295767.5090	-0.0453	0.030	-0.0453	0.50	Melli+22
927:102	13 89	1102	101	13 88	1101	295767.5090	-0.0453	0.030	-0.0453	0.50	Melli+22
928:102	16 87	1102	101	16 86	1101	296012.5280	0.0531	0.030	0.0531	0.50	Melli+22
929:102	16 86	1102	101	16 85	1101	296012.5280	0.0531	0.030	0.0531	0.50	Melli+22
930:102	3 99	1102	101	3 98	1101	296595.8390	-0.0318	0.030			Melli+22
931:104	1104	1104	103	1103	1103	297664.8800	0.0310	0.030			Melli+22
932:104	0104	1104	103	0103	1103	297794.3630	0.0627	0.030			Melli+22
933:103	1102	1103	102	1101	1102	298278.8730	0.0066	0.030			Melli+22
934:103	3101	1103	102	3100	1102	298322.9220	0.0019	0.030			Melli+22
935:103	7 96	1103	102	7 95	1102	298393.2680	0.0205	0.030	0.0234	0.50	Melli+22
936:103	7 97	1103	102	7 96	1102	298393.2680	0.0262	0.030	0.0234	0.50	Melli+22
937:103	8 95	1103	102	8 94	1102	298407.0950	-0.0102	0.030	-0.0102	0.50	Melli+22
938:103	8 96	1103	102	8 95	1102	298407.0950	-0.0101	0.030	-0.0102	0.50	Melli+22
939:103	9 94	1103	102	9 93	1102	298437.0040	0.0108	0.030	0.0108	0.50	Melli+22
940:103	9 95	1103	102	9 94	1102	298437.0040	0.0108	0.030	0.0108	0.50	Melli+22
941:103	5 99	1103	102	5 98	1102	298449.9690	0.0131	0.030			Melli+22
942:103	5 98	1103	102	5 97	1102	298457.9220	-0.0265	0.030			Melli+22
943:103	10 94	1103	102	10 93	1102	298478.9580	-0.0276	0.030	-0.0277	0.50	Melli+22
944:103	10 93	1103	102	10 92	1102	298478.9580	-0.0276	0.030	-0.0277	0.50	Melli+22
945:103	4100	1103	102	4 99	1102	298520.9100	-0.0195	0.030			Melli+22
946:103	11 92	1103	102	11 91	1102	298530.9040	0.0071	0.030	0.0072	0.50	Melli+22
947:103	11 93	1103	102	11 92	1102	298530.9040	0.0071	0.030	0.0072	0.50	Melli+22
948:103	12 92	1103	102	12 91	1102	298591.4130	-0.0090	0.030	-0.0090	0.50	Melli+22
949:103	12 91	1103	102	12 90	1102	298591.4130	-0.0090	0.030	-0.0090	0.50	Melli+22
950:103	13 90	1103	102	13 89	1102	298659.7180	-0.0201	0.030	-0.0201	0.50	Melli+22
951:103	13 91	1103	102	13 90	1102	298659.7180	-0.0201	0.030	-0.0201	0.50	Melli+22
952:103	4 99	1103	102	4 98	1102	298666.5600	0.0416	0.030			Melli+22
953:103	3100	1103	102	3 99	1102	299523.8010	0.0108	0.030			Melli+22
954:104	2103	1104	103	2102	1103	300001.8810	0.0524	0.030			Melli+22
955:103	2101	1103	102	2100	1102	300153.5460	-0.0768	0.030			Melli+22
956:105	1105	1105	104	1104	1104	300508.9540	0.0229	0.030			Melli+22
957:104	3102	1104	103	3101	1103	301206.3420	-0.0212	0.030			Melli+22
958:104	7 97	1104	103	7 96	1103	301284.5030	-0.0090	0.030	-0.0058	0.50	Melli+22
959:104	7 98	1104	103	7 97	1103	301284.5030	-0.0025	0.030	-0.0058	0.50	Melli+22

960:104	8 96	1104	103	8 95	1103	301297.8750	-0.0122	0.030	-0.0122	0.50	Melli+22
961:104	8 97	1104	103	8 96	1103	301297.8750	-0.0121	0.030	-0.0122	0.50	Melli+22
962:104	5100	1104	103	5 99	1103	301344.1290	0.0045	0.030			Melli+22
963:104	5 99	1104	103	5 98	1103	301352.8130	-0.0105	0.030			Melli+22
964:104	4101	1104	103	4100	1103	301415.7030	0.0656	0.030			Melli+22
965:104	11 93	1104	103	11 92	1103	301421.9410	0.0112	0.030	0.0113	0.50	Melli+22
966:104	11 94	1104	103	11 93	1103	301421.9410	0.0112	0.030	0.0113	0.50	Melli+22
967:104	12 93	1104	103	12 92	1103	301482.8650	-0.0009	0.030	-0.0009	0.50	Melli+22
968:104	12 92	1104	103	12 91	1103	301482.8650	-0.0009	0.030	-0.0009	0.50	Melli+22
969:104	13 91	1104	103	13 90	1103	301551.6490	-0.0546	0.030	-0.0546	0.50	Melli+22
970:104	13 92	1104	103	13 91	1103	301551.6490	-0.0546	0.030	-0.0546	0.50	Melli+22
971:104	4100	1104	103	4 99	1103	301570.8350	0.0485	0.030			Melli+22
972:104	14 91	1104	103	14 90	1103	301627.8300	-0.0525	0.030	-0.0526	0.50	Melli+22
973:104	14 90	1104	103	14 89	1103	301627.8300	-0.0525	0.030	-0.0526	0.50	Melli+22
974:104	16 88	1104	103	16 87	1103	301800.8630	0.0356	0.030	0.0356	0.50	Melli+22
975:104	16 89	1104	103	16 88	1103	301800.8630	0.0356	0.030	0.0356	0.50	Melli+22
976:104	3101	1104	103	3100	1103	302451.9000	0.0330	0.030			Melli+22
977:105	2104	1105	104	2103	1104	302861.1220	0.0555	0.030			Melli+22
978:104	2102	1104	103	2101	1103	303051.1260	-0.0748	0.030			Melli+22
979:106	1106	1106	105	1105	1105	303352.7290	0.0251	0.030			Melli+22
980:106	0106	1106	105	0105	1105	303471.3350	-0.0442	0.030			Melli+22
981:105	7 98	1105	104	7 97	1104	304175.5610	-0.0488	0.030	-0.0452	0.50	Melli+22
982:105	7 99	1105	104	7 98	1104	304175.5610	-0.0414	0.030	-0.0452	0.50	Melli+22
983:105	8 97	1105	104	8 96	1104	304188.5140	0.0291	0.030	0.0292	0.50	Melli+22
984:105	8 98	1105	104	8 97	1104	304188.5140	0.0292	0.030	0.0292	0.50	Melli+22
985:105	9 96	1105	104	9 95	1104	304218.0990	-0.0002	0.030	-0.0003	0.50	Melli+22
986:105	9 97	1105	104	9 96	1104	304218.0990	-0.0002	0.030	-0.0003	0.50	Melli+22
987:105	5101	1105	104	5100	1104	304238.1190	-0.0609	0.030			Melli+22
988:105	5100	1105	104	5 99	1104	304247.5880	-0.0514	0.030			Melli+22
989:105	10 95	1105	104	10 94	1104	304260.3180	0.0250	0.030	0.0250	0.50	Melli+22
990:105	10 96	1105	104	10 95	1104	304260.3180	0.0250	0.030	0.0250	0.50	Melli+22
991:105	11 94	1105	104	11 93	1104	304312.7300	-0.0212	0.030	-0.0213	0.50	Melli+22
992:105	11 95	1105	104	11 94	1104	304312.7300	-0.0212	0.030	-0.0213	0.50	Melli+22
993:105	13 93	1105	104	13 92	1104	304443.4710	0.0222	0.030	0.0222	0.50	Melli+22
994:105	13 92	1105	104	13 91	1104	304443.4710	0.0222	0.030	0.0222	0.50	Melli+22
995:105	3102	1105	104	3101	1104	305380.1000	0.0398	0.030			Melli+22
996:105	2103	1105	104	2102	1104	305947.5410	-0.0022	0.030			Melli+22
997:106	3104	1106	105	3103	1105	306971.5660	-0.0132	0.030			Melli+22
998:106	7 99	1106	105	7 98	1105	307066.5820	0.0427	0.030	0.0468	0.50	Melli+22
999:106	7100	1106	105	7 99	1105	307066.5820	0.0509	0.030	0.0468	0.50	Melli+22
1000:106	8 98	1106	105	8 97	1105	307078.8870	-0.0091	0.030	-0.0091	0.50	Melli+22
1001:106	8 99	1106	105	8 98	1105	307078.8870	-0.0090	0.030	-0.0091	0.50	Melli+22
1002:106	9 97	1106	105	9 96	1105	307108.3350	-0.0208	0.030	-0.0208	0.50	Melli+22
1003:106	9 98	1106	105	9 97	1105	307108.3350	-0.0208	0.030	-0.0208	0.50	Melli+22
1004:106	5102	1106	105	5101	1105	307132.1560	0.0359	0.030			Melli+22
1005:106	5101	1106	105	5100	1105	307142.4010	0.0032	0.030			Melli+22
1006:106	10 96	1106	105	10 95	1105	307150.6520	0.0147	0.030	0.0148	0.50	Melli+22
1007:106	10 97	1106	105	10 96	1105	307150.6520	0.0147	0.030	0.0148	0.50	Melli+22
1008:106	11 96	1106	105	11 95	1105	307203.3060	-0.0534	0.030	-0.0535	0.50	Melli+22
1009:106	11 95	1106	105	11 94	1105	307203.3060	-0.0534	0.030	-0.0535	0.50	Melli+22
1010:106	4103	1106	105	4102	1105	307204.4320	0.0344	0.030			Melli+22
1011:106	12 95	1106	105	12 94	1105	307265.0850	-0.0176	0.030	-0.0177	0.50	Melli+22
1012:106	12 94	1106	105	12 93	1105	307265.0850	-0.0176	0.030	-0.0177	0.50	Melli+22
1013:106	4102	1106	105	4101	1105	307380.1680	-0.0225	0.030			Melli+22
1014:106	14 92	1106	105	14 91	1105	307412.4270	0.0527	0.030	0.0528	0.50	Melli+22
1015:106	14 93	1106	105	14 92	1105	307412.4270	0.0527	0.030	0.0528	0.50	Melli+22
1016:106	17 89	1106	105	17 88	1105	307686.2200	-0.0224	0.030	-0.0224	0.50	Melli+22
1017:106	17 90	1106	105	17 89	1105	307686.2200	-0.0224	0.030	-0.0224	0.50	Melli+22
1018:106	3103	1106	105	3102	1105	308308.3630	0.0357	0.030			Melli+22
1019:107	2106	1107	106	2105	1106	308577.7050	-0.0456	0.030			Melli+22
1020:106	2104	1106	105	2103	1105	308842.5680	-0.0652	0.030			Melli+22
1021:107	1106	1107	106	1105	1106	309645.3120	0.0119	0.030			Melli+22
1022:107	3105	1107	106	3104	1106	309853.2610	-0.0750	0.030			Melli+22
1023:107	8 99	1107	106	8 98	1106	309969.0610	-0.0585	0.030	-0.0585	0.50	Melli+22
1024:107	8100	1107	106	8 99	1106	309969.0610	-0.0583	0.030	-0.0585	0.50	Melli+22
1025:107	5103	1107	106	5102	1106	310025.8980	-0.0447	0.030			Melli+22
1026:107	5102	1107	106	5101	1106	310037.0510	-0.0486	0.030			Melli+22
1027:107	5102	1107	106	5101	1106	310037.0830	-0.0166	0.030			Melli+22
1028:107	10 97	1107	106	10 96	1106	310040.8220	0.0493	0.030	0.0493	0.50	Melli+22

1029:	107 10 98	1107	106 10 97	1106	310040.8220	0.0493	0.030	0.0493	0.50	Melli+22
1030:	107 11 97	1107	106 11 96	1106	310093.7060	-0.0463	0.030	-0.0463	0.50	Melli+22
1031:	107 11 96	1107	106 11 95	1106	310093.7060	-0.0463	0.030	-0.0463	0.50	Melli+22
1032:	107 4104	1107	106 4103	1106	310098.3780	-0.0557	0.030			Melli+22
1033:	107 12 95	1107	106 12 94	1106	310155.8820	-0.0094	0.030	-0.0094	0.50	Melli+22
1034:	107 12 96	1107	106 12 95	1106	310155.8820	-0.0094	0.030	-0.0094	0.50	Melli+22
1035:	107 4103	1107	106 4102	1106	310285.3050	-0.0423	0.030			Melli+22
1036:	107 14 94	1107	106 14 93	1106	310304.2720	-0.0073	0.030	-0.0074	0.50	Melli+22
1037:	107 14 93	1107	106 14 92	1106	310304.2720	-0.0073	0.030	-0.0074	0.50	Melli+22
1038:	107 14 94	1107	106 14 93	1106	310304.3220	0.0426	0.030	0.0426	0.50	Melli+22
1039:	107 14 93	1107	106 14 92	1106	310304.3220	0.0426	0.030	0.0426	0.50	Melli+22
1040:	107 16 91	1107	106 16 90	1106	310481.6910	0.0564	0.030	0.0564	0.50	Melli+22
1041:	107 16 92	1107	106 16 91	1106	310481.6910	0.0564	0.030	0.0564	0.50	Melli+22
1042:	107 3104	1107	106 3103	1106	311236.6290	0.0043	0.030			Melli+22
1043:	108 2107	1108	107 2106	1107	311435.2040	0.0049	0.030			Melli+22
1044:	107 2105	1107	106 2104	1106	311736.4590	0.0046	0.030			Melli+22
1045:	109 1109	1109	108 1108	1108	311882.2490	0.0669	0.030			Melli+22
1046:	109 0109	1109	108 0108	1108	311986.1510	0.0002	0.030			Melli+22
1047:	108 1107	1108	107 1106	1107	312484.3440	-0.0558	0.030			Melli+22
1048:	108 3106	1108	107 3105	1107	312734.4990	-0.0157	0.030			Melli+22
1049:	108 7101	1108	107 7100	1107	312847.9260	0.0390	0.030	0.0443	0.50	Melli+22
1050:	108 7102	1108	107 7101	1107	312847.9260	0.0495	0.030	0.0443	0.50	Melli+22
1051:	108 8100	1108	107 8 99	1107	312859.1710	0.0178	0.030	0.0179	0.50	Melli+22
1052:	108 8101	1108	107 8100	1107	312859.1710	0.0179	0.030	0.0179	0.50	Melli+22
1053:	108 9 99	1108	107 9 98	1107	312888.3130	0.0463	0.030	0.0463	0.50	Melli+22
1054:	108 9100	1108	107 9 99	1107	312888.3130	0.0463	0.030	0.0463	0.50	Melli+22
1055:	108 10 99	1108	107 10 98	1107	312930.7230	0.0256	0.030			Melli+22
1056:	108 5103	1108	107 5102	1107	312931.8000	0.0533	0.030			Melli+22
1057:	108 11 98	1108	107 11 97	1107	312983.9120	-0.0158	0.030	-0.0158	0.50	Melli+22
1058:	108 11 97	1108	107 11 96	1107	312983.9120	-0.0158	0.030	-0.0158	0.50	Melli+22
1059:	108 4105	1108	107 4104	1107	312992.2490	0.0192	0.030			Melli+22
1060:	108 12 97	1108	107 12 96	1107	313046.4940	0.0362	0.030	0.0362	0.50	Melli+22
1061:	108 12 96	1108	107 12 95	1107	313046.4940	0.0362	0.030	0.0362	0.50	Melli+22
1062:	108 13 96	1108	107 13 95	1107	313117.3520	0.0104	0.030	0.0104	0.50	Melli+22
1063:	108 13 95	1108	107 13 94	1107	313117.3520	0.0104	0.030	0.0104	0.50	Melli+22
1064:	108 4104	1108	107 4103	1107	313190.8060	-0.0151	0.030			Melli+22
1065:	108 14 94	1108	107 14 93	1107	313195.9390	-0.0156	0.030	-0.0156	0.50	Melli+22
1066:	108 14 95	1108	107 14 94	1107	313195.9390	-0.0156	0.030	-0.0156	0.50	Melli+22
1067:	108 17 91	1108	107 17 90	1107	313474.4010	-0.0332	0.030	-0.0333	0.50	Melli+22
1068:	108 17 92	1108	107 17 91	1107	313474.4010	-0.0332	0.030	-0.0333	0.50	Melli+22
1069:	109 2108	1109	108 2107	1108	314292.0510	-0.0025	0.030			Melli+22
1070:	108 2106	1108	107 2105	1107	314629.0270	0.0371	0.030			Melli+22
1071:	110 1110	1110	109 1109	1109	314724.7510	0.0182	0.030			Melli+22
1072:	110 0110	1110	109 0109	1109	314824.2060	0.0382	0.030			Melli+22
1073:	109 1108	1109	108 1107	1108	315322.5720	0.0076	0.030			Melli+22
1074:	109 3107	1109	108 3106	1108	315615.1600	0.0520	0.030			Melli+22
1075:	109 7102	1109	108 7101	1108	315738.3460	0.0440	0.030	0.0499	0.50	Melli+22
1076:	109 7103	1109	108 7102	1108	315738.3460	0.0558	0.030	0.0499	0.50	Melli+22
1077:	109 8101	1109	108 8100	1108	315749.0340	0.0386	0.030	0.0387	0.50	Melli+22
1078:	109 8102	1109	108 8101	1108	315749.0340	0.0388	0.030	0.0387	0.50	Melli+22
1079:	109 9100	1109	108 9 99	1108	315777.9350	0.0177	0.030	0.0177	0.50	Melli+22
1080:	109 9101	1109	108 9100	1108	315777.9350	0.0177	0.030	0.0177	0.50	Melli+22
1081:	109 5105	1109	108 5104	1108	315813.2700	0.0444	0.030			Melli+22
1082:	109 10 99	1109	108 10 98	1108	315820.4080	-0.0013	0.030	-0.0013	0.50	Melli+22
1083:	109 10100	1109	108 10 99	1108	315820.4080	-0.0013	0.030	-0.0013	0.50	Melli+22
1084:	109 5104	1109	108 5103	1108	315826.3130	-0.0273	0.030			Melli+22
1085:	109 11 98	1109	108 11 97	1108	315873.8900	0.0059	0.030	0.0060	0.50	Melli+22
1086:	109 11 99	1109	108 11 98	1108	315873.8900	0.0059	0.030	0.0060	0.50	Melli+22
1087:	109 4106	1109	108 4105	1108	315885.7940	0.0169	0.030			Melli+22
1088:	109 12 98	1109	108 12 97	1108	315936.8170	0.0173	0.030	0.0174	0.50	Melli+22
1089:	109 12 97	1109	108 12 96	1108	315936.8170	0.0173	0.030	0.0174	0.50	Melli+22
1090:	109 14 96	1109	108 14 95	1108	316087.3650	-0.0329	0.030	-0.0329	0.50	Melli+22
1091:	109 14 95	1109	108 14 94	1108	316087.3650	-0.0329	0.030	-0.0329	0.50	Melli+22
1092:	109 16 94	1109	108 16 93	1108	316267.6600	-0.0067	0.030	-0.0068	0.50	Melli+22
1093:	109 16 93	1109	108 16 92	1108	316267.6600	-0.0067	0.030	-0.0068	0.50	Melli+22
1094:	109 3106	1109	108 3105	1108	317093.1320	0.0003	0.030			Melli+22
1095:	110 2109	1110	109 2108	1109	317148.3130	-0.0023	0.030			Melli+22
1096:	109 2107	1109	108 2106	1108	317520.2080	-0.0156	0.030			Melli+22
1097:	111 1111	1111	110 1110	1110	317566.9790	-0.0021	0.030			Melli+22

1098:111	0111	1111	110	0110	1110	317662.0840	0.0257	0.030			Melli+22
1099:110	1109	1110	109	1108	1109	318159.8500	0.0210	0.030			Melli+22
1100:110	3108	1110	109	3107	1109	318495.1310	0.0229	0.030			Melli+22
1101:110	7103	1110	109	7102	1109	318628.5240	-0.0184	0.030	-0.0118	0.50	Melli+22
1102:110	7104	1110	109	7103	1109	318628.5240	-0.0051	0.030	-0.0118	0.50	Melli+22
1103:110	8102	1110	109	8101	1109	318638.6150	-0.0293	0.030	-0.0292	0.50	Melli+22
1104:110	8103	1110	109	8102	1109	318638.6150	-0.0291	0.030	-0.0292	0.50	Melli+22
1105:110	6105	1110	109	6104	1109	318646.2260	0.0212	0.030			Melli+22
1106:110	6104	1110	109	6103	1109	318646.7320	-0.0045	0.030			Melli+22
1107:110	9101	1110	109	9100	1109	318667.3480	-0.0141	0.030	-0.0142	0.50	Melli+22
1108:110	9102	1110	109	9101	1109	318667.3480	-0.0141	0.030	-0.0142	0.50	Melli+22
1109:110	5106	1110	109	5105	1109	318706.6620	-0.0188	0.030			Melli+22
1110:110	10101	1110	109	10100	1109	318709.8790	-0.0276	0.030	-0.0277	0.50	Melli+22
1111:110	10100	1110	109	10 99	1109	318709.8790	-0.0276	0.030	-0.0277	0.50	Melli+22
1112:110	5105	1110	109	5104	1109	318720.8720	-0.0104	0.030			Melli+22
1113:110	11100	1110	109	11 99	1109	318763.6250	0.0060	0.030	0.0061	0.50	Melli+22
1114:110	11 99	1110	109	11 98	1109	318763.6250	0.0060	0.030	0.0061	0.50	Melli+22
1115:110	4107	1110	109	4106	1109	318779.0900	0.0226	0.030			Melli+22
1116:110	12 98	1110	109	12 97	1109	318826.9160	0.0009	0.030	0.0010	0.50	Melli+22
1117:110	12 99	1110	109	12 98	1109	318826.9160	0.0009	0.030	0.0010	0.50	Melli+22
1118:110	13 98	1110	109	13 97	1109	318898.7990	0.0016	0.030	0.0016	0.50	Melli+22
1119:110	13 97	1110	109	13 96	1109	318898.7990	0.0016	0.030	0.0016	0.50	Melli+22
1120:110	4106	1110	109	4105	1109	319002.7900	0.0286	0.030			Melli+22
1121:110	17 94	1110	109	17 93	1109	319261.6330	-0.0349	0.030	-0.0349	0.50	Melli+22
1122:110	17 93	1110	109	17 92	1109	319261.6330	-0.0349	0.030	-0.0349	0.50	Melli+22
1123:111	2110	1111	110	2109	1110	320004.0220	0.0357	0.030			Melli+22
1124:110	3107	1110	109	3106	1109	320021.2190	-0.0309	0.030			Melli+22
1125:112	1112	1112	111	1111	1111	320408.9580	0.0294	0.030			Melli+22
1126:110	2108	1110	109	2107	1109	320410.1410	0.0014	0.030			Melli+22
1127:111	1110	1111	110	1109	1110	320996.2290	-0.0004	0.030			Melli+22
1128:111	3109	1111	110	3108	1110	321374.5270	0.0192	0.030			Melli+22
1129:111	7104	1111	110	7103	1110	321518.6040	-0.0026	0.030	0.0048	0.50	Melli+22
1130:111	7105	1111	110	7104	1110	321518.6040	0.0122	0.030	0.0048	0.50	Melli+22
1131:111	8103	1111	110	8102	1110	321528.0850	-0.0133	0.030	-0.0132	0.50	Melli+22
1132:111	8104	1111	110	8103	1110	321528.0850	-0.0131	0.030	-0.0132	0.50	Melli+22
1133:111	6106	1111	110	6105	1110	321537.4970	-0.0145	0.030			Melli+22
1134:111	6105	1111	110	6104	1110	321538.1110	0.0131	0.030			Melli+22
1135:111	9102	1111	110	9101	1110	321556.6120	0.0124	0.030	0.0124	0.50	Melli+22
1136:111	9103	1111	110	9102	1110	321556.6120	0.0124	0.030	0.0124	0.50	Melli+22
1137:111	10101	1111	110	10100	1110	321599.1580	-0.0294	0.030	-0.0295	0.50	Melli+22
1138:111	10102	1111	110	10101	1110	321599.1580	-0.0294	0.030	-0.0295	0.50	Melli+22
1139:111	5107	1111	110	5106	1110	321600.0270	0.0185	0.030			Melli+22
1140:111	5106	1111	110	5105	1110	321615.3340	-0.0407	0.030			Melli+22
1141:111	11100	1111	110	11 99	1110	321653.1610	0.0304	0.030	0.0304	0.50	Melli+22
1142:111	11101	1111	110	11100	1110	321653.1610	0.0304	0.030	0.0304	0.50	Melli+22
1143:111	4108	1111	110	4107	1110	321672.1050	0.0131	0.030			Melli+22
1144:111	12 99	1111	110	12 98	1110	321716.7830	-0.0189	0.030	-0.0190	0.50	Melli+22
1145:111	12100	1111	110	12 99	1110	321716.7830	-0.0189	0.030	-0.0190	0.50	Melli+22
1146:111	13 98	1111	110	13 97	1110	321789.1620	-0.0152	0.030	-0.0152	0.50	Melli+22
1147:111	13 99	1111	110	13 98	1110	321789.1620	-0.0152	0.030	-0.0152	0.50	Melli+22
1148:111	4107	1111	110	4106	1110	321909.2600	0.0115	0.030			Melli+22
1149:112	2111	1112	111	2110	1111	322859.0880	0.0199	0.030			Melli+22
1150:111	3108	1111	110	3107	1110	322949.2250	0.0085	0.030			Melli+22
1151:113	1113	1113	112	1112	1112	323250.5850	0.0089	0.030			Melli+22
1152:111	2109	1111	110	2108	1110	323298.7230	0.0015	0.030			Melli+22
1153:113	0113	1113	112	0112	1112	323337.4540	0.0099	0.030			Melli+22
1154:112	1111	1112	111	1110	1111	323831.8220	0.0204	0.030			Melli+22
1155:112	3110	1112	111	3109	1111	324253.3090	0.0089	0.030			Melli+22
1156:112	7105	1112	111	7104	1111	324408.4750	-0.0181	0.030	-0.0097	0.50	Melli+22
1157:112	7106	1112	111	7105	1111	324408.4750	-0.0013	0.030	-0.0097	0.50	Melli+22
1158:112	8104	1112	111	8103	1111	324417.3650	0.0093	0.030	0.0095	0.50	Melli+22
1159:112	8105	1112	111	8104	1111	324417.3650	0.0096	0.030	0.0095	0.50	Melli+22
1160:112	6107	1112	111	6106	1111	324428.6690	0.0004	0.030			Melli+22
1161:112	6106	1112	111	6105	1111	324429.2690	-0.0453	0.030			Melli+22
1162:112	9103	1112	111	9102	1111	324445.6400	0.0124	0.030	0.0125	0.50	Melli+22
1163:112	9104	1112	111	9103	1111	324445.6400	0.0124	0.030	0.0125	0.50	Melli+22
1164:112	10102	1112	111	10101	1111	324488.2290	-0.0208	0.030	-0.0209	0.50	Melli+22
1165:112	10103	1112	111	10102	1111	324488.2290	-0.0208	0.030	-0.0209	0.50	Melli+22
1166:112	5108	1112	111	5107	1111	324493.1870	-0.0188	0.030			Melli+22

1167:112	5107	1112	111	5106	1111	324509.8030	-0.0163	0.030			Melli+22
1168:112	11101	1112	111	11100	1111	324542.4180	0.0010	0.030	0.0010	0.50	Melli+22
1169:112	11102	1112	111	11101	1111	324542.4180	0.0010	0.030	0.0010	0.50	Melli+22
1170:112	4109	1112	111	4108	1111	324564.8100	-0.0318	0.030			Melli+22
1171:112	12100	1112	111	12 99	1111	324606.4570	-0.0013	0.030	-0.0014	0.50	Melli+22
1172:112	12101	1112	111	12100	1111	324606.4570	-0.0013	0.030	-0.0014	0.50	Melli+22
1173:112	13100	1112	111	13 99	1111	324679.3170	-0.0054	0.030	-0.0054	0.50	Melli+22
1174:112	13 99	1112	111	13 98	1111	324679.3170	-0.0054	0.030	-0.0054	0.50	Melli+22
1175:112	14 99	1112	111	14 98	1111	324760.3130	-0.0021	0.030	-0.0021	0.50	Melli+22
1176:112	14 98	1112	111	14 97	1111	324760.3130	-0.0021	0.030	-0.0021	0.50	Melli+22
1177:112	4108	1112	111	4107	1111	324816.0650	-0.0286	0.030			Melli+22
1178:113	2112	1113	112	2111	1112	325713.6230	0.0605	0.030			Melli+22
1179:112	3109	1112	111	3108	1111	325876.9710	-0.0132	0.030			Melli+22
1180:114	1114	1114	113	1113	1113	326091.9320	0.0074	0.030			Melli+22
1181:114	0114	1114	113	0113	1113	326174.9420	0.0106	0.030			Melli+22
1182:112	2110	1112	111	2109	1111	326185.9430	-0.0107	0.030			Melli+22
1183:113	1112	1113	112	1111	1112	326666.5690	-0.0123	0.030			Melli+22
1184:113	3111	1113	112	3110	1112	327131.4910	0.0130	0.030			Melli+22
1185:113	7106	1113	112	7105	1112	327298.1760	-0.0242	0.030	-0.0149	0.50	Melli+22
1186:113	7107	1113	112	7106	1112	327298.1760	-0.0055	0.030	-0.0149	0.50	Melli+22
1187:113	8105	1113	112	8104	1112	327306.4170	0.0024	0.030	0.0026	0.50	Melli+22
1188:113	8106	1113	112	8105	1112	327306.4170	0.0028	0.030	0.0026	0.50	Melli+22
1189:113	6108	1113	112	6107	1112	327319.6340	-0.0404	0.030			Melli+22
1190:113	6107	1113	112	6106	1112	327320.3710	-0.0141	0.030			Melli+22
1191:113	9104	1113	112	9103	1112	327334.4390	-0.0052	0.030	-0.0053	0.50	Melli+22
1192:113	9105	1113	112	9104	1112	327334.4390	-0.0052	0.030	-0.0053	0.50	Melli+22
1193:113	10103	1113	112	10102	1112	327377.0790	-0.0128	0.030	-0.0128	0.50	Melli+22
1194:113	10104	1113	112	10103	1112	327377.0790	-0.0128	0.030	-0.0128	0.50	Melli+22
1195:113	5109	1113	112	5108	1112	327386.2720	0.0018	0.030			Melli+22
1196:113	5108	1113	112	5107	1112	327404.2520	0.0338	0.030			Melli+22
1197:113	12102	1113	112	12101	1112	327495.8790	-0.0032	0.030	-0.0032	0.50	Melli+22
1198:113	12101	1113	112	12100	1112	327495.8790	-0.0032	0.030	-0.0032	0.50	Melli+22
1199:113	4109	1113	112	4108	1112	327723.3290	0.0219	0.030			Melli+22
1200:114	2113	1114	113	2112	1113	328567.4880	0.0162	0.030			Melli+22
1201:113	3110	1113	112	3109	1112	328804.4880	-0.0182	0.030			Melli+22
1202:115	1115	1115	114	1114	1114	328932.9690	-0.0062	0.030			Melli+22
1203:115	0115	1115	114	0114	1114	329012.2860	0.0100	0.030			Melli+22
1204:113	2111	1113	112	2110	1112	329071.8150	-0.0059	0.030			Melli+22
1205:114	3112	1114	113	3111	1113	330009.0540	0.0193	0.030			Melli+22
1206:114	7107	1114	113	7106	1113	330187.7000	-0.0265	0.030	-0.0160	0.50	Melli+22
1207:114	7108	1114	113	7107	1113	330187.7000	-0.0055	0.030	-0.0160	0.50	Melli+22
1208:114	8106	1114	113	8105	1113	330195.2340	-0.0392	0.030	-0.0390	0.50	Melli+22
1209:114	8107	1114	113	8106	1113	330195.2340	-0.0388	0.030	-0.0390	0.50	Melli+22
1210:114	6109	1114	113	6108	1113	330210.5070	-0.0207	0.030			Melli+22
1211:114	6108	1114	113	6107	1113	330211.3600	0.0510	0.030			Melli+22
1212:114	9105	1114	113	9104	1113	330223.0380	-0.0098	0.030	-0.0099	0.50	Melli+22
1213:114	9106	1114	113	9105	1113	330223.0380	-0.0098	0.030	-0.0099	0.50	Melli+22
1214:114	10104	1114	113	10103	1113	330265.7230	0.0114	0.030	0.0115	0.50	Melli+22
1215:114	10105	1114	113	10104	1113	330265.7230	0.0114	0.030	0.0115	0.50	Melli+22
1216:114	5110	1114	113	5109	1113	330279.1760	-0.0226	0.030			Melli+22
1217:114	5109	1114	113	5108	1113	330298.5700	-0.0034	0.030			Melli+22
1218:114	11104	1114	113	11103	1113	330320.2450	-0.0611	0.030	-0.0611	0.50	Melli+22
1219:114	11103	1114	113	11102	1113	330320.2450	-0.0611	0.030	-0.0611	0.50	Melli+22
1220:114	4111	1114	113	4110	1113	330349.4890	0.0063	0.030			Melli+22
1221:114	12103	1114	113	12102	1113	330385.0940	0.0224	0.030	0.0224	0.50	Melli+22
1222:114	12102	1114	113	12101	1113	330385.0940	0.0224	0.030	0.0224	0.50	Melli+22
1223:114	13101	1114	113	13100	1113	330458.9160	0.0156	0.030	0.0157	0.50	Melli+22
1224:114	13102	1114	113	13101	1113	330458.9160	0.0156	0.030	0.0157	0.50	Melli+22
1225:114	4110	1114	113	4109	1113	330630.8910	-0.0074	0.030			Melli+22
1226:116	10106	1116	115	10105	1115	336042.2750	-0.0012	0.030	-0.0013	0.50	Melli+22
1227:116	10107	1116	115	10106	1115	336042.2750	-0.0012	0.030	-0.0013	0.50	Melli+22
1228:116	5112	1116	115	5111	1115	336064.6420	0.0062	0.030			Melli+22
1229:116	11106	1116	115	11105	1115	336097.3230	0.0523	0.030	0.0523	0.50	Melli+22
1230:116	11105	1116	115	11104	1115	336097.3230	0.0523	0.030	0.0523	0.50	Melli+22
1231:116	4113	1116	115	4112	1115	336132.9540	0.0363	0.030			Melli+22
1232:116	4112	1116	115	4111	1115	336447.2009	-0.0518	0.030			Melli+22
1233:117	2116	1117	116	2115	1116	337125.6820	-0.0282	0.030			Melli+22
1234:118	1118	1118	117	1117	1117	337454.3369	-0.0111	0.030			Melli+22
1235:116	3113	1116	115	3112	1115	337585.1780	0.0499	0.030			Melli+22

1236:116	2114	1116	115	2113	1115	337721.0878	0.0070	0.030			Melli+22
1237:117	3115	1117	116	3114	1116	338637.9079	-0.0051	0.030			Melli+22
1238:117	8109	1117	116	8108	1116	338860.6220	-0.0087	0.030	-0.0084	0.50	Melli+22
1239:117	8110	1117	116	8109	1116	338860.6220	-0.0081	0.030	-0.0084	0.50	Melli+22
1240:117	6111	1117	116	6110	1116	338883.1490	-0.0409	0.030			Melli+22
1241:117	9108	1117	116	9107	1116	338887.5856	0.0238	0.030	0.0239	0.50	Melli+22
1242:117	9109	1117	116	9108	1116	338887.5856	0.0238	0.030	0.0239	0.50	Melli+22
1243:117	10107	1117	116	10106	1116	338930.1788	-0.0387	0.030	-0.0387	0.50	Melli+22
1244:117	10108	1117	116	10107	1116	338930.1788	-0.0387	0.030	-0.0387	0.50	Melli+22
1245:117	5113	1117	116	5112	1116	338957.1210	-0.0174	0.030			Melli+22
1246:117	5112	1117	116	5111	1116	338981.3604	-0.0427	0.030			Melli+22
1247:117	11106	1117	116	11105	1116	338985.3700	-0.0312	0.030	-0.0313	0.50	Melli+22
1248:117	11107	1117	116	11106	1116	338985.3700	-0.0312	0.030	-0.0313	0.50	Melli+22
1249:117	4114	1117	116	4113	1116	339024.0940	-0.0660	0.030			Melli+22
1250:118	2117	1118	117	2116	1117	339977.3349	0.0335	0.030			Melli+22
1251:117	3114	1117	116	3113	1116	340511.2346	0.0366	0.030			Melli+22
1252:117	2115	1117	116	2114	1116	340601.3385	0.0003	0.030			Melli+22
1253:118	1117	1118	117	1116	1117	340829.8082	-0.0359	0.030			Melli+22
1254:118	3116	1118	117	3115	1117	341512.9238	0.0024	0.030			Melli+22
1255:118	7111	1118	117	7110	1117	341743.9479	-0.0438	0.030	-0.0276	0.50	Melli+22
1256:118	7112	1118	117	7111	1117	341743.9479	-0.0112	0.030	-0.0276	0.50	Melli+22
1257:118	8110	1118	117	8109	1117	341748.6379	-0.0333	0.030	-0.0330	0.50	Melli+22
1258:118	8111	1118	117	8110	1117	341748.6379	-0.0326	0.030	-0.0330	0.50	Melli+22
1259:118	9109	1118	117	9108	1117	341775.2502	-0.0443	0.030	-0.0443	0.50	Melli+22
1260:118	9110	1118	117	9109	1117	341775.2502	-0.0443	0.030	-0.0443	0.50	Melli+22
1261:118	10108	1118	117	10107	1117	341817.9350	0.0062	0.030	0.0062	0.50	Melli+22
1262:118	10109	1118	117	10108	1117	341817.9350	0.0062	0.030	0.0062	0.50	Melli+22
1263:118	5114	1118	117	5113	1117	341849.4350	-0.0577	0.030			Melli+22
1264:118	11108	1118	117	11107	1117	341873.3458	0.0509	0.030	0.0510	0.50	Melli+22
1265:118	11107	1118	117	11106	1117	341873.3458	0.0509	0.030	0.0510	0.50	Melli+22
1266:118	5113	1118	117	5112	1117	341875.6270	0.0171	0.030			Melli+22
1267:118	4115	1118	117	4114	1117	341915.1030	0.0298	0.030			Melli+22
1268:119	2118	1119	118	2117	1118	342828.3070	-0.0123	0.030			Melli+22
1269:120	0120	1120	119	0119	1119	343196.6941	-0.0214	0.030			Melli+22
1270:118	3115	1118	117	3114	1117	343436.7690	-0.0199	0.030			Melli+22
1271:118	2116	1118	117	2115	1117	343480.1810	0.0235	0.030			Melli+22
1272:119	1118	1119	118	1117	1118	343660.6140	0.0008	0.030			Melli+22
1273:119	3117	1119	118	3116	1118	344387.2580	-0.0194	0.030			Melli+22
1274:119	7112	1119	118	7111	1118	344632.5791	-0.0112	0.030			Melli+22
1275:119	8111	1119	118	8110	1118	344636.5060	0.0030	0.030	0.0035	0.50	Melli+22
1276:119	8112	1119	118	8111	1118	344636.5060	0.0038	0.030	0.0035	0.50	Melli+22
1277:119	5114	1119	118	5113	1118	344769.8356	0.0491	0.030			Melli+22
1278:119	4116	1119	118	4115	1118	344805.6951	0.0474	0.030			Melli+22
1279:119	12108	1119	118	12107	1118	344827.4100	-0.0200	0.030	-0.0200	0.50	Melli+22
1280:119	12107	1119	118	12106	1118	344827.4100	-0.0200	0.030	-0.0200	0.50	Melli+22
1281:120	2119	1120	119	2118	1119	345678.7720	0.0052	0.030			Melli+22
1282:121	0121	1121	120	0120	1120	346033.1737	0.0559	0.030			Melli+22
1283:119	2117	1119	118	2116	1118	346357.5240	-0.0014	0.030			Melli+22
1284:120	3118	1120	119	3117	1119	347260.9658	-0.0099	0.030			Melli+22
1285:120	8112	1120	119	8111	1119	347524.1797	0.0557	0.030	0.0562	0.50	Melli+22
1286:120	8113	1120	119	8112	1119	347524.1797	0.0566	0.030	0.0562	0.50	Melli+22
1287:120	9111	1120	119	9110	1119	347550.1480	0.0566	0.030	0.0566	0.50	Melli+22
1288:120	9112	1120	119	9111	1119	347550.1480	0.0566	0.030	0.0566	0.50	Melli+22
1289:120	6115	1120	119	6114	1119	347552.3980	0.0379	0.030			Melli+22
1290:120	10110	1120	119	10109	1119	347592.6151	-0.0387	0.030	-0.0387	0.50	Melli+22
1291:120	10111	1120	119	10110	1119	347592.6151	-0.0387	0.030	-0.0387	0.50	Melli+22
1292:120	4116	1120	119	4115	1119	348084.9650	0.0733	0.030			Melli+22
1293:122	1122	1122	121	1121	1121	348812.0620	0.0098	0.030			Melli+22
1294:122	0122	1122	121	0121	1121	348869.3640	0.0155	0.030			Melli+22
1295:121	7114	1121	120	7113	1120	350409.2162	0.0006	0.030	0.0231	0.50	Melli+22
1296:121	7115	1121	120	7114	1120	350409.2162	0.0455	0.030	0.0231	0.50	Melli+22
1297:121	8113	1121	120	8112	1120	350411.5360	0.0033	0.030	0.0038	0.50	Melli+22
1298:121	8114	1121	120	8113	1120	350411.5360	0.0043	0.030	0.0038	0.50	Melli+22
1299:121	9112	1121	120	9111	1120	350437.1560	0.0043	0.030	0.0043	0.50	Melli+22
1300:121	9113	1121	120	9112	1120	350437.1560	0.0043	0.030	0.0043	0.50	Melli+22
1301:121	6116	1121	120	6115	1120	350442.0828	-0.0208	0.030			Melli+22
1302:121	10111	1121	120	10110	1120	350479.6644	0.0006	0.030	0.0007	0.50	Melli+22
1303:121	10112	1121	120	10111	1120	350479.6644	0.0006	0.030	0.0007	0.50	Melli+22
1304:121	5117	1121	120	5116	1120	350525.6479	0.0156	0.030			Melli+22

1305:121	11110	1121	120	11109	1120	350535.5656	0.0321	0.030	0.0322	0.50	Melli+22
1306:121	11111	1121	120	11110	1120	350535.5656	0.0321	0.030	0.0322	0.50	Melli+22
1307:121	4118	1121	120	4117	1120	350585.7230	-0.0197	0.030			Melli+22
1308:121	12109	1121	120	12108	1120	350602.7040	0.0425	0.030	0.0426	0.50	Melli+22
1309:121	12110	1121	120	12109	1120	350602.7040	0.0425	0.030	0.0426	0.50	Melli+22
1310:121	4117	1121	120	4116	1120	350995.4440	0.0699	0.030			Melli+22
1311:123	1123	1123	122	1122	1122	351650.7090	-0.0357	0.030			Melli+22
1312:122	1121	1122	121	1120	1121	352149.6980	-0.0504	0.030			Melli+22
1313:122	7115	1122	121	7114	1121	353297.2000	-0.0390	0.030	-0.0141	0.50	Melli+22
1314:122	7116	1122	121	7115	1121	353297.2000	0.0107	0.030	-0.0141	0.50	Melli+22
1315:122	8114	1122	121	8113	1121	353298.7070	-0.0202	0.030	-0.0198	0.50	Melli+22
1316:122	8115	1122	121	8114	1121	353298.7070	-0.0192	0.030	-0.0198	0.50	Melli+22
1317:122	9113	1122	121	9112	1121	353324.0200	0.0358	0.030	0.0358	0.50	Melli+22
1318:122	9114	1122	121	9113	1121	353324.0200	0.0358	0.030	0.0358	0.50	Melli+22
1319:122	6116	1122	121	6115	1121	353333.3194	0.0194	0.030			Melli+22
1320:122	5118	1122	121	5117	1121	353417.3700	0.0107	0.030			Melli+22
1321:122	4119	1122	121	4118	1121	353475.2400	-0.0041	0.030			Melli+22
1322:122	12110	1122	121	12109	1121	353489.9360	0.0337	0.030	0.0338	0.50	Melli+22
1323:122	12111	1122	121	12110	1121	353489.9360	0.0337	0.030	0.0338	0.50	Melli+22
1324:122	4118	1122	121	4117	1121	353906.3054	0.0066	0.030			Melli+22
1325:123	2122	1123	122	2121	1122	354226.7821	0.0675	0.030			Melli+22
1326:122	2120	1122	121	2119	1121	354980.7967	0.0013	0.030			Melli+22
1327:122	3119	1122	121	3118	1121	355133.3990	-0.0675	0.030			Melli+22
1328:123	9114	1123	122	9113	1122	356210.5580	-0.0290	0.030	-0.0290	0.50	Melli+22
1329:123	9115	1123	122	9114	1122	356210.5580	-0.0290	0.030	-0.0290	0.50	Melli+22
1330:123	6118	1123	122	6117	1122	356221.0445	-0.0510	0.030			Melli+22
1331:123	5119	1123	122	5118	1122	356308.9786	0.0583	0.030			Melli+22
1332:123	5118	1123	122	5117	1122	356346.2600	0.0022	0.030			Melli+22
1333:123	12112	1123	122	12111	1122	356376.8835	-0.0070	0.030	-0.0070	0.50	Melli+22
1334:123	12111	1123	122	12110	1122	356376.8835	-0.0070	0.030	-0.0070	0.50	Melli+22
1335:123	2121	1123	122	2120	1122	357852.2605	0.0253	0.030			Melli+22
1336:124	3122	1124	123	3121	1123	358749.0857	0.0001	0.030			Melli+22
1337:124	6118	1124	123	6117	1123	359112.3140	0.0488	0.030			Melli+22
1338:124	10114	1124	123	10113	1123	359139.2148	-0.0453	0.030	-0.0453	0.50	Melli+22
1339:124	10115	1124	123	10114	1123	359139.2148	-0.0453	0.030	-0.0453	0.50	Melli+22
1340:124	5119	1124	123	5118	1123	359240.3129	-0.0218	0.030			Melli+22
1341:124	4121	1124	123	4120	1123	359253.1100	0.0035	0.030			Melli+22
1342:124	4120	1124	123	4119	1123	359729.4630	-0.0333	0.030			Melli+22
1343:126	1126	1126	125	1125	1125	360165.1220	0.0544	0.030			Melli+22
1344:126	0126	1126	125	0125	1125	360212.5227	0.0469	0.030			Melli+22
1345:125	1124	1125	124	1123	1124	360634.7310	-0.0297	0.030			Melli+22
1346:124	2122	1124	123	2121	1123	360722.0930	-0.0726	0.030			Melli+22
1347:125	3123	1125	124	3122	1124	361619.3937	-0.0237	0.030			Melli+22
1348:125	7118	1125	124	7117	1124	361960.1360	0.0010	0.030	0.0350	0.50	Melli+22
1349:125	7119	1125	124	7118	1124	361960.1360	0.0689	0.030	0.0350	0.50	Melli+22
1350:125	6120	1125	124	6119	1124	361999.3840	-0.0327	0.030			Melli+22
1351:125	12114	1125	124	12113	1124	362150.0650	-0.0363	0.030	-0.0364	0.50	Melli+22
1352:125	12113	1125	124	12112	1124	362150.0650	-0.0363	0.030	-0.0364	0.50	Melli+22
1353:125	4121	1125	124	4120	1124	362641.7750	-0.0027	0.030			Melli+22
1354:127	1127	1127	126	1126	1126	363002.6100	0.0192	0.030			Melli+22
1355:127	0127	1127	126	0126	1126	363047.7680	-0.0208	0.030			Melli+22
1356:125	2123	1125	124	2122	1124	363590.6200	0.0426	0.030			Melli+22
1357:126	3124	1126	125	3123	1125	364489.0710	0.0091	0.030			Melli+22
1358:126	8118	1126	125	8117	1125	364845.2930	-0.0376	0.030	-0.0367	0.50	Melli+22
1359:126	8119	1126	125	8118	1125	364845.2930	-0.0358	0.030	-0.0367	0.50	Melli+22
1360:126	7119	1126	125	7118	1125	364847.3350	-0.0352	0.030	0.0023	0.50	Melli+22
1361:126	7120	1126	125	7119	1125	364847.3350	0.0398	0.030	0.0023	0.50	Melli+22
1362:126	9117	1126	125	9116	1125	364868.9970	-0.0022	0.030	-0.0022	0.50	Melli+22
1363:126	9118	1126	125	9117	1125	364868.9970	-0.0022	0.030	-0.0022	0.50	Melli+22
1364:126	6121	1126	125	6120	1125	364888.3200	-0.0019	0.030			Melli+22
1365:126	6120	1126	125	6119	1125	364890.5800	-0.0249	0.030			Melli+22
1366:126	10116	1126	125	10115	1125	364911.1390	0.0280	0.030	0.0281	0.50	Melli+22
1367:126	10117	1126	125	10116	1125	364911.1390	0.0280	0.030	0.0281	0.50	Melli+22
1368:126	5122	1126	125	5121	1125	364982.5940	0.0253	0.030			Melli+22
1369:126	17109	1126	125	17108	1125	365522.9560	-0.0394	0.030	-0.0395	0.50	Melli+22
1370:126	17110	1126	125	17109	1125	365522.9560	-0.0394	0.030	-0.0395	0.50	Melli+22
1371:126	17110	1126	125	17109	1125	365522.9630	-0.0324	0.030	-0.0325	0.50	Melli+22
1372:126	17109	1126	125	17108	1125	365522.9630	-0.0324	0.030	-0.0325	0.50	Melli+22
1373:126	4122	1126	125	4121	1125	365554.5640	0.0456	0.030			Melli+22

1374:127	2126	1127	126	2125	1126	365616.1560	-0.0162	0.030			Melli+22
1375:128	1128	1128	127	1127	1127	365839.8030	-0.0187	0.030			Melli+22
1376:128	0128	1128	127	0127	1127	365882.9340	0.0273	0.030			Melli+22
1377:127	1126	1127	126	1125	1126	366289.4790	-0.0337	0.030			Melli+22
1378:126	2124	1126	125	2123	1125	366457.4410	-0.0207	0.030			Melli+22
1379:126	3123	1126	125	3122	1125	366819.0290	0.0344	0.030			Melli+22
1380:127	3125	1127	126	3124	1126	367358.0550	0.0403	0.030			Melli+22
1381:127	8119	1127	126	8118	1126	367731.4340	0.0049	0.030	0.0060	0.50	Melli+22
1382:127	8120	1127	126	8119	1126	367731.4340	0.0069	0.030	0.0060	0.50	Melli+22
1383:127	7120	1127	126	7119	1126	367734.3690	-0.0358	0.030	0.0057	0.50	Melli+22
1384:127	7121	1127	126	7120	1126	367734.3690	0.0472	0.030	0.0057	0.50	Melli+22
1385:127	9118	1127	126	9117	1126	367754.6400	-0.0251	0.030	-0.0251	0.50	Melli+22
1386:127	9119	1127	126	9118	1126	367754.6400	-0.0250	0.030	-0.0251	0.50	Melli+22
1387:127	6122	1127	126	6121	1126	367777.0240	-0.0306	0.030			Melli+22
1388:127	10117	1127	126	10116	1126	367796.7080	0.0413	0.030	0.0414	0.50	Melli+22
1389:127	10118	1127	126	10117	1126	367796.7080	0.0413	0.030	0.0414	0.50	Melli+22
1390:127	4124	1127	126	4123	1126	367916.9280	0.0259	0.030			Melli+22
1391:127	14114	1127	126	14113	1126	368091.3480	-0.0002	0.030	-0.0003	0.50	Melli+22
1392:127	14113	1127	126	14112	1126	368091.3480	-0.0002	0.030	-0.0003	0.50	Melli+22
1393:127	4123	1127	126	4122	1126	368467.7480	0.0280	0.030			Melli+22
1394:129	1129	1129	128	1128	1128	368676.7380	-0.0227	0.030			Melli+22
1395:129	0129	1129	128	0128	1128	368717.8090	-0.0163	0.030			Melli+22
1396:127	2125	1127	126	2124	1126	369322.8250	0.0138	0.030			Melli+22
1397:127	3124	1127	126	3123	1126	369738.3780	0.0454	0.030			Melli+22
1398:128	8120	1128	127	8119	1127	370617.3060	0.0029	0.030	0.0041	0.50	Melli+22
1399:128	8121	1128	127	8120	1127	370617.3060	0.0052	0.030	0.0041	0.50	Melli+22
1400:128	9119	1128	127	9118	1127	370640.0900	-0.0021	0.030	-0.0022	0.50	Melli+22
1401:128	9120	1128	127	9119	1127	370640.0900	-0.0021	0.030	-0.0022	0.50	Melli+22
1402:128	6123	1128	127	6122	1127	370665.6110	-0.0022	0.030			Melli+22
1403:128	10118	1128	127	10117	1127	370682.0150	0.0417	0.030	0.0417	0.50	Melli+22
1404:128	10119	1128	127	10118	1127	370682.0150	0.0417	0.030	0.0417	0.50	Melli+22
1405:128	11118	1128	127	11117	1127	370738.8180	0.0575	0.030	0.0576	0.50	Melli+22
1406:128	11117	1128	127	11116	1127	370738.8180	0.0575	0.030	0.0576	0.50	Melli+22
1407:128	4125	1128	127	4124	1127	370803.9880	-0.0074	0.030			Melli+22
1408:128	12116	1128	127	12115	1127	370807.9720	-0.0016	0.030	-0.0016	0.50	Melli+22
1409:128	12117	1128	127	12116	1127	370807.9720	-0.0016	0.030	-0.0016	0.50	Melli+22
1410:128	5123	1128	127	5122	1127	370816.6010	0.0010	0.030			Melli+22
1411:128	14114	1128	127	14113	1127	370977.9840	-0.0072	0.030	-0.0073	0.50	Melli+22
1412:128	14115	1128	127	14114	1127	370977.9840	-0.0072	0.030	-0.0073	0.50	Melli+22
1413:129	2128	1129	128	2127	1128	371307.5980	-0.0156	0.030			Melli+22
1414:128	4124	1128	127	4123	1127	371381.3610	-0.0225	0.030			Melli+22
1415:130	0130	1130	129	0129	1129	371552.5810	0.0397	0.030			Melli+22
1416:129	1128	1129	128	1127	1128	371942.9760	0.0218	0.030			Melli+22
1417:128	2126	1128	127	2125	1127	372186.6750	0.0559	0.030			Melli+22
1418:129	3127	1129	128	3126	1128	373093.8400	0.0108	0.030			Melli+22
1419:129	8121	1129	128	8120	1128	373502.9430	-0.0078	0.030	-0.0066	0.50	Melli+22
1420:129	8122	1129	128	8121	1128	373502.9430	-0.0053	0.030	-0.0066	0.50	Melli+22
1421:129	7123	1129	128	7122	1128	373507.7770	0.0128	0.030			Melli+22
1422:129	9120	1129	128	9119	1128	373525.3060	0.0274	0.030	0.0274	0.50	Melli+22
1423:129	9121	1129	128	9120	1128	373525.3060	0.0274	0.030	0.0274	0.50	Melli+22
1424:129	6124	1129	128	6123	1128	373553.9870	-0.0090	0.030			Melli+22
1425:129	6123	1129	128	6122	1128	373556.9040	-0.0258	0.030			Melli+22
1426:129	10119	1129	128	10118	1128	373567.0120	-0.0170	0.030	-0.0170	0.50	Melli+22
1427:129	10120	1129	128	10119	1128	373567.0120	-0.0170	0.030	-0.0170	0.50	Melli+22
1428:129	11118	1129	128	11117	1128	373623.9490	0.0319	0.030	0.0320	0.50	Melli+22
1429:129	11119	1129	128	11118	1128	373623.9490	0.0319	0.030	0.0320	0.50	Melli+22
1430:129	5125	1129	128	5124	1128	373654.5660	-0.0123	0.030			Melli+22
1431:129	4126	1129	128	4125	1128	373690.6220	-0.0311	0.030			Melli+22
1432:129	5124	1129	128	5123	1128	373710.6100	-0.0680	0.030			Melli+22
1433:129	14116	1129	128	14115	1128	373864.3480	-0.0130	0.030	-0.0131	0.50	Melli+22
1434:129	14115	1129	128	14114	1128	373864.3480	-0.0130	0.030	-0.0131	0.50	Melli+22
1435:129	15115	1129	128	15114	1128	373964.0310	-0.0387	0.030	-0.0387	0.50	Melli+22
1436:129	15114	1129	128	15113	1128	373964.0310	-0.0387	0.030	-0.0387	0.50	Melli+22
1437:130	2129	1130	129	2128	1129	374152.5080	-0.0162	0.030			Melli+22
1438:129	17113	1129	128	17112	1128	374189.3320	-0.0077	0.030	-0.0077	0.50	Melli+22
1439:129	17112	1129	128	17111	1128	374189.3320	-0.0077	0.030	-0.0077	0.50	Melli+22
1440:129	17112	1129	128	17111	1128	374189.3500	0.0102	0.030	0.0103	0.50	Melli+22
1441:129	17113	1129	128	17112	1128	374189.3500	0.0102	0.030	0.0103	0.50	Melli+22
1442:129	4125	1129	128	4124	1128	374295.4930	-0.0156	0.030			Melli+22

1443:131	1131	1131	130	1130	1130	374349.7320	-0.0297	0.030			Melli+22
1444:131	0131	1131	130	0130	1130	374387.0150	-0.0355	0.030			Melli+22
1445:130	1129	1130	129	1128	1129	374769.2320	-0.0054	0.030			Melli+22
1446:129	2127	1129	128	2126	1128	375048.8800	0.0000	0.030			Melli+22
1447:129	3126	1129	128	3125	1128	375574.3270	0.0316	0.030			Melli+22
1448:130	3128	1130	129	3127	1129	375960.6800	-0.0034	0.030			Melli+22
1449:130	8122	1130	129	8121	1129	376388.3950	0.0240	0.030	0.0255	0.50	Melli+22
1450:130	8123	1130	129	8122	1129	376388.3950	0.0268	0.030	0.0255	0.50	Melli+22
1451:130	7123	1130	129	7122	1129	376394.2570	-0.0315	0.030	0.0243	0.50	Melli+22
1452:130	7124	1130	129	7123	1129	376394.2570	0.0801	0.030	0.0243	0.50	Melli+22
1453:130	9121	1130	129	9120	1129	376410.2170	-0.0055	0.030	-0.0055	0.50	Melli+22
1454:130	9122	1130	129	9121	1129	376410.2170	-0.0054	0.030	-0.0055	0.50	Melli+22
1455:130	6125	1130	129	6124	1129	376442.1950	-0.0065	0.030			Melli+22
1456:130	6124	1130	129	6123	1129	376445.3960	0.0094	0.030			Melli+22
1457:130	10120	1130	129	10119	1129	376451.7830	-0.0489	0.030	-0.0489	0.50	Melli+22
1458:130	10121	1130	129	10120	1129	376451.7830	-0.0489	0.030	-0.0489	0.50	Melli+22
1459:130	11120	1130	129	11119	1129	376508.8710	0.0579	0.030	0.0579	0.50	Melli+22
1460:130	11119	1130	129	11118	1129	376508.8710	0.0579	0.030	0.0579	0.50	Melli+22
1461:130	5126	1130	129	5125	1129	376544.8320	-0.0316	0.030			Melli+22
1462:130	12119	1130	129	12118	1129	376578.6090	0.0397	0.030	0.0398	0.50	Melli+22
1463:130	12118	1130	129	12117	1129	376578.6090	0.0397	0.030	0.0398	0.50	Melli+22
1464:130	5125	1130	129	5124	1129	376604.7830	0.0123	0.030			Melli+22
1465:130	13118	1130	129	13117	1129	376659.5260	0.0529	0.030	0.0530	0.50	Melli+22
1466:130	13117	1130	129	13116	1129	376659.5260	0.0529	0.030	0.0530	0.50	Melli+22
1467:130	15115	1130	129	15114	1129	376850.8380	0.0518	0.030	0.0518	0.50	Melli+22
1468:130	15116	1130	129	15115	1129	376850.8380	0.0518	0.030	0.0518	0.50	Melli+22
1469:131	2130	1131	130	2129	1130	376996.9040	0.0047	0.030			Melli+22
1470:132	1132	1132	131	1131	1131	377185.7590	-0.0644	0.030			Melli+22
1471:132	0132	1132	131	0131	1131	377221.3470	-0.0027	0.030			Melli+22
1472:130	3127	1130	129	3126	1129	378490.8090	-0.0414	0.030			Melli+22
1473:131	3129	1131	130	3128	1130	378826.8140	-0.0170	0.030			Melli+22
1474:131	8123	1131	130	8122	1130	379273.5530	-0.0083	0.030	-0.0068	0.50	Melli+22
1475:131	8124	1131	130	8123	1130	379273.5530	-0.0052	0.030	-0.0068	0.50	Melli+22
1476:131	7124	1131	130	7123	1130	379280.4390	-0.0658	0.030	-0.0043	0.50	Melli+22
1477:131	7125	1131	130	7124	1130	379280.4390	0.0572	0.030	-0.0043	0.50	Melli+22
1478:131	9122	1131	130	9121	1130	379294.9370	0.0148	0.030	0.0149	0.50	Melli+22
1479:131	9123	1131	130	9122	1130	379294.9370	0.0148	0.030	0.0149	0.50	Melli+22
1480:131	10121	1131	130	10120	1130	379336.3900	0.0098	0.030	0.0099	0.50	Melli+22
1481:131	10122	1131	130	10121	1130	379336.3900	0.0098	0.030	0.0099	0.50	Melli+22
1482:131	11120	1131	130	11119	1130	379393.4470	0.0003	0.030	0.0003	0.50	Melli+22
1483:131	11121	1131	130	11120	1130	379393.4470	0.0003	0.030	0.0003	0.50	Melli+22
1484:131	5127	1131	130	5126	1130	379434.9270	-0.0221	0.030			Melli+22
1485:131	4128	1131	130	4127	1130	379462.5930	-0.0294	0.030			Melli+22
1486:131	5126	1131	130	5125	1130	379498.9100	0.0273	0.030			Melli+22
1487:131	13119	1131	130	13118	1130	379544.7440	-0.0277	0.030	-0.0277	0.50	Melli+22
1488:131	13118	1131	130	13117	1130	379544.7440	-0.0277	0.030	-0.0277	0.50	Melli+22
1489:131	14117	1131	130	14116	1130	379636.2810	0.0080	0.030	0.0080	0.50	Melli+22
1490:131	14118	1131	130	14117	1130	379636.2810	0.0080	0.030	0.0080	0.50	Melli+22
1491:132	2131	1132	131	2130	1131	379840.7840	0.0416	0.030			Melli+22
1492:133	1133	1133	132	1132	1132	380021.5570	-0.0353	0.030			Melli+22
1493:133	0133	1133	132	0132	1132	380055.4490	0.0138	0.030			Melli+22
1494:131	4127	1131	130	4126	1130	380125.1530	0.0168	0.030			Melli+22
1495:131	2129	1131	130	2128	1130	380768.7500	0.0058	0.030			Melli+22
1496:132	3130	1132	131	3129	1131	381692.2960	0.0271	0.030			Melli+22
1497:132	8124	1132	131	8123	1131	382158.5120	-0.0086	0.030	-0.0069	0.50	Melli+22
1498:132	8125	1132	131	8124	1131	382158.5120	-0.0051	0.030	-0.0069	0.50	Melli+22
1499:132	7125	1132	131	7124	1131	382166.4600	-0.0528	0.030			Melli+22
1500:132	9123	1132	131	9122	1131	382179.3450	-0.0307	0.030	-0.0307	0.50	Melli+22
1501:132	9124	1132	131	9123	1131	382179.3450	-0.0306	0.030	-0.0307	0.50	Melli+22
1502:132	4129	1132	131	4128	1131	382347.9300	0.0156	0.030			Melli+22
1503:132	5127	1132	131	5126	1131	382393.0100	-0.0097	0.030			Melli+22
1504:132	13120	1132	131	13119	1131	382429.7790	-0.0161	0.030	-0.0162	0.50	Melli+22
1505:132	13119	1132	131	13118	1131	382429.7790	-0.0161	0.030	-0.0162	0.50	Melli+22
1506:132	14118	1132	131	14117	1131	382521.8630	0.0519	0.030	0.0520	0.50	Melli+22
1507:132	14119	1132	131	14118	1131	382521.8630	0.0519	0.030	0.0520	0.50	Melli+22
1508:133	2132	1133	132	2131	1132	382683.9960	-0.0610	0.030			Melli+22
1509:132	17116	1132	131	17115	1131	382853.0950	-0.0329	0.030	-0.0329	0.25	Melli+22
1510:132	17115	1132	131	17114	1131	382853.0950	-0.0329	0.030	-0.0329	0.25	Melli+22
1511:132	17115	1132	131	17114	1131	382853.0950	-0.0329	0.030	-0.0329	0.25	Melli+22

1512:132	17116	1132	131	17115	1131	382853.0950	-0.0329	0.030	-0.0329	0.25	Melli+22
1513:134	0134	1134	133	0133	1133	382889.2690	-0.0341	0.030			Melli+22
1514:132	4128	1132	131	4127	1131	383040.6160	-0.0156	0.030			Melli+22
1515:133	1132	1133	132	1131	1132	383246.5300	-0.0361	0.030			Melli+22
1516:132	2130	1132	131	2129	1131	383626.3700	0.0275	0.030			Melli+22
1517:132	3129	1132	131	3128	1131	384320.9400	-0.0026	0.030			Melli+22
1518:133	8125	1133	132	8124	1132	385043.2240	-0.0228	0.030	-0.0209	0.50	Melli+22
1519:133	8126	1133	132	8125	1132	385043.2240	-0.0189	0.030	-0.0209	0.50	Melli+22
1520:133	9124	1133	132	9123	1132	385063.6160	0.0346	0.030	0.0347	0.50	Melli+22
1521:133	9125	1133	132	9124	1132	385063.6160	0.0347	0.030	0.0347	0.50	Melli+22
1522:133	6128	1133	132	6127	1132	385105.7680	0.0316	0.030			Melli+22
1523:133	6127	1133	132	6126	1132	385109.8230	0.0278	0.030			Melli+22
1524:133	5129	1133	132	5128	1132	385214.5300	0.0289	0.030			Melli+22
1525:133	5128	1133	132	5127	1132	385287.1830	-0.0042	0.030			Melli+22
1526:133	13121	1133	132	13120	1132	385314.5710	0.0297	0.030	0.0297	0.50	Melli+22
1527:133	13120	1133	132	13119	1132	385314.5710	0.0297	0.030	0.0297	0.50	Melli+22
1528:133	14120	1133	132	14119	1132	385407.0810	0.0131	0.030	0.0132	0.50	Melli+22
1529:133	14119	1133	132	14118	1132	385407.0810	0.0131	0.030	0.0132	0.50	Melli+22
1530:134	2133	1134	133	2132	1133	385526.8340	-0.0126	0.030			Melli+22
1531:135	1135	1135	134	1134	1134	385692.1940	-0.0564	0.030			Melli+22
1532:135	0135	1135	134	0134	1134	385722.8990	-0.0513	0.030			Melli+22

(Z)-cyanovinylacetylene Wed Jul 13 14:42:07 2022

/ instead of : below denotes (o-c)>3*err

											obs	o-c	error	blends o-c	wt	Notes
1:	1	1	0	0	0	1	0	1	0	1	5153.4986	-0.0015	0.002			Halter+01
2:	1	1	0	0	2	1	0	1	0	1	5154.4806	0.0019	0.002			Halter+01
3:	1	1	0	0	2	1	0	1	0	2	5154.5535	0.0001	0.002			Halter+01
4:	1	1	0	0	1	1	0	1	0	1	5155.1309	-0.0001	0.002			Halter+01
5:	1	1	0	0	1	1	0	1	0	2	5155.2044	-0.0013	0.002			Halter+01
6:	1	1	0	0	1	1	0	1	0	0	5155.3178	0.0000	0.002			Halter+01
7:	2	1	1	0	1	2	0	2	0	1	5978.3287	-0.0001	0.002			Halter+01
8:	2	1	1	0	1	2	0	2	0	2	5978.3791	-0.0023	0.002			Halter+01
9:	2	1	1	0	3	2	0	2	0	3	5978.7805	0.0000	0.002			Halter+01
10:	2	1	1	0	2	2	0	2	0	1	5979.5418	0.0011	0.002			Halter+01
11:	2	1	1	0	2	2	0	2	0	2	5979.5926	-0.0006	0.002			Halter+01
12:	3	1	2	0	2	3	0	3	0	2	7363.8024	0.0006	0.002			Halter+01
13:	3	1	2	0	2	3	0	3	0	3	7364.0769	0.0021	0.002			Halter+01
14:	3	1	2	0	4	3	0	3	0	4	7364.1485	0.0002	0.002			Halter+01
15:	3	1	2	0	4	3	0	3	0	3	7364.3508	0.0002	0.002			Halter+01
16:	3	1	2	0	3	3	0	3	0	2	7364.8667	0.0011	0.002			Halter+01
17:	3	1	2	0	3	3	0	3	0	4	7364.9356	-0.0007	0.002			Halter+01
18:	3	1	2	0	3	3	0	3	0	3	7365.1383	-0.0002	0.002			Halter+01
19:	2	1	2	0	3	1	1	1	0	2	8513.2772	0.0007	0.002			Halter+01
20:	2	1	2	0	2	1	1	1	0	1	8513.3073	0.0027	0.002			Halter+01
21:	2	1	2	0	1	1	1	1	0	1	8514.3903	-0.0016	0.002			Halter+01
22:	2	1	2	0	1	1	1	1	0	0	8512.5760	0.0018	0.002	0.0007	0.64	Halter+01
23:	2	1	2	0	2	1	1	1	0	2	8512.5760	-0.0014	0.002	0.0007	0.36	Halter+01
24:	1	1	1	0	0	0	0	0	0	1	9042.9459	-0.0004	0.002			Halter+01
25:	1	1	1	0	1	0	0	0	0	1	9041.1281	-0.0004	0.002			Halter+01
26:	1	1	1	0	2	0	0	0	0	1	9041.8566	0.0009	0.002			Halter+01
27:	2	0	2	0	1	1	0	1	0	0	9167.0929	0.0006	0.002			Halter+01
28:	2	0	2	0	2	1	0	1	0	1	9166.8522	-0.0007	0.002			Halter+01
29:	2	0	2	0	3	1	0	1	0	2	9166.9609	-0.0005	0.002			Halter+01
30:	4	1	3	0	3	4	0	4	0	3	9443.0133	0.0009	0.002			Halter+01
31:	4	1	3	0	5	4	0	4	0	5	9443.3082	0.0003	0.002			Halter+01
32:	4	1	3	0	3	4	0	4	0	4	9443.5171	-0.0028	0.003			Halter+01
33:	4	1	3	0	5	4	0	4	0	4	9443.7171	0.0054	0.003			Halter+01
34:	4	1	3	0	4	4	0	4	0	3	9443.9511	0.0012	0.003			Halter+01
35:	4	1	3	0	4	4	0	4	0	5	9444.0517	-0.0018	0.003			Halter+01
36:	4	1	3	0	4	4	0	4	0	4	9444.4574	0.0000	0.002			Halter+01
37:	3	0	3	0	2	2	1	2	0	2	9783.2255	0.0006	0.002			Halter+01
38:	3	0	3	0	2	2	1	2	0	1	9782.1375	0.0000	0.002			Halter+01
39:	3	0	3	0	3	2	1	2	0	3	9782.2551	0.0022	0.004			Halter+01
40:	3	0	3	0	4	2	1	2	0	3	9782.4547	-0.0003	0.002			Halter+01
41:	3	0	3	0	3	2	1	2	0	2	9782.9508	-0.0010	0.002			Halter+01
42:	2	1	1	0	2	1	1	0	0	2	9991.9675	-0.0000	0.002			Halter+01
43:	2	1	1	0	1	1	1	0	0	1	9990.1033	-0.0001	0.002			Halter+01
44:	2	1	1	0	3	1	1	0	0	2	9991.1896	0.0010	0.002			Halter+01
45:	2	1	1	0	2	1	1	0	0	1	9991.3172	0.0020	0.002			Halter+01
46:	2	1	1	0	1	1	1	0	0	0	9991.7365	0.0021	0.002			Halter+01
47:	5	2	3	0	4	5	1	4	0	4	11895.9307	0.0054	0.002	0.0045	0.22	Halter+01
48:	5	2	3	0	6	5	1	4	0	6	11895.9307	0.0050	0.002	0.0045	0.47	Halter+01
49:	5	2	3	0	5	5	1	4	0	5	11895.9307	0.0029	0.002	0.0045	0.31	Halter+01
50:	4	2	2	0	4	4	1	3	0	4	12051.3742	0.0013	0.002			Halter+01
51:	4	2	2	0	5	4	1	3	0	5	12051.6273	0.0026	0.002			Halter+01
52:	4	2	2	0	3	4	1	3	0	3	12051.6871	-0.0023	0.003			Halter+01
53:	5	1	4	0	4	5	0	5	0	4	12292.5839	-0.0002	0.002			Halter+01
54:	5	1	4	0	6	5	0	5	0	6	12292.8407	-0.0011	0.002			Halter+01
55:	5	1	4	0	5	5	0	5	0	5	12294.1064	0.0004	0.002			Halter+01
56:	3	2	1	0	3	3	1	2	0	3	12613.6487	-0.0013	0.002			Halter+01
57:	3	2	1	0	4	3	1	2	0	4	12614.1525	-0.0003	0.002			Halter+01
58:	3	2	1	0	2	3	1	2	0	2	12614.3255	-0.0033	0.002			Halter+01
59:	3	2	1	0	3	3	1	2	0	4	12614.4395	0.0013	0.004			Halter+01
60:	3	2	1	0	3	3	1	2	0	2	12614.7138	-0.0001	0.002			Halter+01
61:	3	2	1	0	2	3	1	2	0	3	12613.2619	-0.0031	0.002			Halter+01
62:	3	2	1	0	4	3	1	2	0	3	12613.3657	0.0008	0.004			Halter+01

63:	3	1	3	0	2	2	1	2	0	1	12719.1336	-0.0026	0.002			Halter+01
64:	3	1	3	0	3	2	1	2	0	2	12719.2242	-0.0027	0.002			Halter+01
65:	3	1	3	0	4	2	1	2	0	3	12719.2643	-0.0019	0.002			Halter+01
66:	2	1	2	0	1	1	0	1	0	1	12929.3360	-0.0014	0.002			Halter+01
67:	2	1	2	0	1	1	0	1	0	0	12929.5246	0.0004	0.002			Halter+01
68:	2	1	2	0	2	1	0	1	0	1	12928.2511	0.0010	0.002			Halter+01
69:	2	1	2	0	2	1	0	1	0	2	12928.3236	-0.0011	0.002			Halter+01
70:	2	1	2	0	3	1	0	1	0	2	12929.0251	0.0014	0.002			Halter+01
71:	2	2	0	0	1	2	1	1	0	1	13332.3906	-0.0034	0.002			Halter+01
72:	2	2	0	0	2	2	1	1	0	1	13332.4398	-0.0067	0.004			Halter+01
73:	2	2	0	0	2	2	1	1	0	2	13331.2316	-0.0031	0.002			Halter+01
74:	2	2	0	0	3	2	1	1	0	3	13331.9779	-0.0020	0.002			Halter+01
75:	3	0	3	0	3	2	0	2	0	2	13544.3487	-0.0001	0.002			Halter+01
76:	3	0	3	0	4	2	0	2	0	3	13544.5176	0.0002	0.002			Halter+01
77:	3	0	3	0	2	2	0	2	0	1	13544.5646	-0.0046	0.004			Halter+01
78:	5	1	4	0	4	4	2	3	0	3	13693.9858	-0.0026	0.002			Halter+01
79:	5	1	4	0	6	4	2	3	0	5	13694.1193	-0.0027	0.002			Halter+01
80:	5	1	4	0	5	4	2	3	0	4	13694.7754	-0.0033	0.002			Halter+01
81:	3	2	2	0	2	2	2	1	0	1	13878.5028	0.0031	0.002			Halter+01
82:	3	2	2	0	4	2	2	1	0	3	13878.5452	0.0009	0.002			Halter+01
83:	3	2	2	0	3	2	2	1	0	2	13878.6242	-0.0000	0.002			Halter+01
84:	3	2	1	0	2	2	2	0	0	1	14211.9834	0.0064	0.004			Halter+01
85:	3	2	1	0	4	2	2	0	0	3	14212.0609	0.0028	0.002			Halter+01
86:	3	2	1	0	3	2	2	0	0	2	14212.3131	0.0036	0.002			Halter+01
87:	4	0	4	0	3	3	1	3	0	3	14776.3011	-0.0052	0.003			Halter+01
88:	4	0	4	0	4	3	1	3	0	4	14775.0512	-0.0093	0.004			Halter+01
89:	4	0	4	0	3	3	1	3	0	2	14775.3086	-0.0010	0.002			Halter+01
90:	4	0	4	0	5	3	1	3	0	4	14775.4641	-0.0002	0.002			Halter+01
91:	4	0	4	0	4	3	1	3	0	3	14775.7982	-0.0006	0.002			Halter+01
92:	3	1	2	0	4	2	1	1	0	3	14929.8859	0.0007	0.002			Halter+01
93:	3	1	2	0	2	2	1	1	0	1	14930.0449	0.0027	0.002			Halter+01
94:	21	0	21	1	21	20	0	20	1	20	83547.7090	-0.0186	0.030	0.0030	0.25	Melli+22
95:	21	1	21	1	21	20	1	20	1	20	83547.7090	0.0247	0.030	0.0030	0.25	Melli+22
96:	21	1	21	1	21	20	0	20	1	20	83547.7090	-0.0600	0.030	0.0030	0.25	Melli+22
97:	21	0	21	1	21	20	1	20	1	20	83547.7090	0.0661	0.030	0.0030	0.25	Melli+22
98:	20	2	19	1	20	19	2	18	1	19	83580.8300	0.0048	0.030			Melli+22
99:	20	1	19	1	20	19	1	18	1	19	83585.4020	0.0307	0.030			Melli+22
100:	20	2	19	1	20	19	1	18	1	19	83590.3180	-0.0146	0.030			Melli+22
101:	19	3	17	1	19	18	3	16	1	18	83628.5210	0.0530	0.030			Melli+22
102:	19	2	17	1	19	18	2	16	1	18	83791.7550	0.0319	0.030			Melli+22
103:	19	3	17	1	19	18	2	16	1	18	84009.8750	0.0177	0.030			Melli+22
104:	18	5	14	1	18	17	5	13	1	17	85212.2530	0.0103	0.030			Melli+22
105:	18	7	12	1	18	17	7	11	1	17	85226.2610	0.0020	0.030			Melli+22
106:	18	6	13	1	18	17	6	12	1	17	85599.5520	0.0099	0.030			Melli+22
107:	22	1	22	1	22	21	0	21	1	21	87429.5710	-0.0478	0.030	-0.0170	0.25	Melli+22
108:	22	0	22	1	22	21	1	21	1	21	87429.5710	0.0138	0.030	-0.0170	0.25	Melli+22
109:	22	0	22	1	22	21	0	21	1	21	87429.5710	-0.0276	0.030	-0.0170	0.25	Melli+22
110:	22	1	22	1	22	21	1	21	1	21	87429.5710	-0.0064	0.030	-0.0170	0.25	Melli+22
111:	21	1	20	1	21	20	2	19	1	20	87458.6120	-0.0492	0.030			Melli+22
112:	21	2	20	1	21	20	2	19	1	20	87461.2200	-0.0119	0.030			Melli+22
113:	21	1	20	1	21	20	1	19	1	20	87463.6200	-0.0027	0.030			Melli+22
114:	21	2	20	1	21	20	1	19	1	20	87466.1700	-0.0234	0.030			Melli+22
115:	20	2	18	1	20	19	2	17	1	19	87618.9420	-0.0093	0.030			Melli+22
116:	19	3	16	1	19	18	3	15	1	18	88659.2030	-0.0104	0.030			Melli+22
117:	19	12	7	1	19	18	12	6	1	18	88872.0960	0.0478	0.030	0.0478	0.50	Melli+22
118:	19	12	8	1	19	18	12	7	1	18	88872.0960	0.0478	0.030	0.0478	0.50	Melli+22
119:	19	11	8	1	19	18	11	7	1	18	88985.0000	0.0400	0.030	0.0405	0.50	Melli+22
120:	19	11	9	1	19	18	11	8	1	18	88985.0000	0.0409	0.030	0.0405	0.50	Melli+22
121:	19	10	10	1	19	18	10	9	1	18	89143.1430	0.0579	0.030	0.0423	0.50	Melli+22
122:	19	10	9	1	19	18	10	8	1	18	89143.1430	0.0267	0.030	0.0423	0.50	Melli+22
123:	19	7	12	1	19	18	7	11	1	18	90343.4390	0.0062	0.030			Melli+22
124:	19	6	14	1	19	18	6	13	1	18	90454.6490	0.0032	0.030			Melli+22
125:	23	0	23	1	23	22	0	22	1	22	91311.0640	0.0271	0.030	0.0323	0.25	Melli+22
126:	23	1	23	1	23	22	1	22	1	22	91311.0640	0.0375	0.030	0.0323	0.25	Melli+22
127:	23	1	23	1	23	22	0	22	1	22	91311.0640	0.0173	0.030	0.0323	0.25	Melli+22
128:	23	0	23	1	23	22	1	22	1	22	91311.0640	0.0473	0.030	0.0323	0.25	Melli+22
129:	22	1	21	1	22	21	2	20	1	21	91339.8000	0.0033	0.030			Melli+22
130:	22	2	21	1	22	21	2	20	1	21	91341.1420	0.0217	0.030			Melli+22
131:	22	1	21	1	22	21	1	20	1	21	91342.3640	-0.0033	0.030			Melli+22

132:	22	2	21	1	22	21	1	20	1	21	91343.6880	-0.0030	0.030		Melli+22
133:	21	3	19	1	21	20	3	18	1	20	91409.2060	0.0031	0.030		Melli+22
134:	21	2	19	1	21	20	2	18	1	20	91463.7830	0.0199	0.030		Melli+22
135:	20	3	17	1	20	19	3	16	1	19	92259.1530	0.0263	0.030		Melli+22
136:	20	12	9	1	20	19	12	8	1	19	93615.9920	-0.0062	0.030	-0.0063 0.50	Melli+22
137:	20	12	8	1	20	19	12	7	1	19	93615.9920	-0.0062	0.030	-0.0063 0.50	Melli+22
138:	19	4	15	1	19	18	4	14	1	18	93682.5420	-0.0051	0.030		Melli+22
139:	20	11	10	1	20	19	11	9	1	19	93750.3300	-0.0022	0.030	-0.0037 0.50	Melli+22
140:	20	11	9	1	20	19	11	8	1	19	93750.3300	-0.0051	0.030	-0.0037 0.50	Melli+22
141:	20	4	17	1	20	19	3	16	1	19	93864.9660	0.0550	0.030		Melli+22
142:	20	10	11	1	20	19	10	10	1	19	93937.9060	0.0694	0.030	0.0252 0.50	Melli+22
143:	20	10	10	1	20	19	10	9	1	19	93937.9060	-0.0190	0.030	0.0252 0.50	Melli+22
144:	20	5	16	1	20	19	5	15	1	19	94147.0370	-0.0008	0.030		Melli+22
145:	20	9	12	1	20	19	9	11	1	19	94205.3370	0.0769	0.030		Melli+22
146:	20	9	11	1	20	19	9	10	1	19	94207.2810	0.0022	0.030		Melli+22
147:	21	3	18	1	21	20	4	17	1	20	94305.0410	-0.0115	0.030		Melli+22
148:	20	8	13	1	20	19	8	12	1	19	94591.4260	0.0102	0.030		Melli+22
149:	20	8	12	1	20	19	8	11	1	19	94624.5610	0.0133	0.030		Melli+22
150:	24	1	24	1	24	23	0	23	1	23	95192.0160	-0.0028	0.030	0.0044 0.25	Melli+22
151:	24	0	24	1	24	23	1	23	1	23	95192.0160	0.0117	0.030	0.0044 0.25	Melli+22
152:	24	0	24	1	24	23	0	23	1	23	95192.0160	0.0018	0.030	0.0044 0.25	Melli+22
153:	24	1	24	1	24	23	1	23	1	23	95192.0160	0.0069	0.030	0.0044 0.25	Melli+22
154:	23	1	22	1	23	22	2	21	1	22	95219.9330	0.0052	0.030		Melli+22
155:	23	2	22	1	23	22	2	21	1	22	95220.6060	0.0005	0.030		Melli+22
156:	23	2	22	1	23	22	1	21	1	22	95221.9190	-0.0100	0.030		Melli+22
157:	23	1	22	1	23	22	1	21	1	22	95221.2650	0.0136	0.030		Melli+22
158:	22	2	20	1	22	21	3	19	1	21	95251.5180	-0.0003	0.030		Melli+22
159:	20	6	15	1	20	19	6	14	1	19	95260.5410	0.0101	0.030		Melli+22
160:	21	4	18	1	21	20	4	17	1	20	95287.1390	0.0145	0.030		Melli+22
161:	22	3	20	1	22	21	3	19	1	21	95289.0880	0.0244	0.030		Melli+22
162:	22	2	20	1	22	21	2	19	1	21	95319.8100	-0.0126	0.030		Melli+22
163:	22	3	20	1	22	21	2	19	1	21	95357.3680	0.0000	0.030		Melli+22
164:	21	3	18	1	21	20	3	17	1	20	95910.8450	0.0081	0.030		Melli+22
165:	21	4	18	1	21	20	3	17	1	20	96892.9080	-0.0007	0.030		Melli+22
166:	20	4	16	1	20	19	4	15	1	19	97695.6770	0.0350	0.030		Melli+22
167:	21	13	9	1	21	20	13	8	1	20	98254.9540	-0.0578	0.030	-0.0578 0.50	Melli+22
168:	21	13	8	1	21	20	13	7	1	20	98254.9540	-0.0578	0.030	-0.0578 0.50	Melli+22
169:	21	12	10	1	21	20	12	9	1	20	98370.2740	-0.0327	0.030	-0.0328 0.50	Melli+22
170:	21	12	9	1	21	20	12	8	1	20	98370.2740	-0.0329	0.030	-0.0328 0.50	Melli+22
171:	21	5	17	1	21	20	5	16	1	20	98457.1990	0.0192	0.030		Melli+22
172:	21	11	10	1	21	20	11	9	1	20	98528.6600	-0.0127	0.030	-0.0083 0.50	Melli+22
173:	21	11	11	1	21	20	11	10	1	20	98528.6600	-0.0039	0.030	-0.0083 0.50	Melli+22
174:	25	0	25	1	25	24	1	24	1	24	99072.5030	0.0030	0.030	-0.0005 0.25	Melli+22
175:	25	0	25	1	25	24	0	24	1	24	99072.5030	-0.0017	0.030	-0.0005 0.25	Melli+22
176:	25	1	25	1	25	24	1	24	1	24	99072.5030	0.0007	0.030	-0.0005 0.25	Melli+22
177:	25	1	25	1	25	24	0	24	1	24	99072.5030	-0.0040	0.030	-0.0005 0.25	Melli+22
178:	24	1	23	1	24	23	2	22	1	23	99099.3810	0.0047	0.030		Melli+22
179:	24	2	23	1	24	23	2	22	1	23	99099.7270	0.0054	0.030		Melli+22
180:	24	1	23	1	24	23	1	22	1	23	99100.0560	0.0019	0.030		Melli+22
181:	24	2	23	1	24	23	1	22	1	23	99100.4210	0.0217	0.030		Melli+22
182:	23	2	21	1	23	22	3	20	1	22	99145.3560	0.0050	0.030		Melli+22
183:	23	3	21	1	23	22	3	20	1	22	99165.7830	-0.0025	0.030		Melli+22
184:	23	2	21	1	23	22	2	20	1	22	99182.9380	0.0417	0.030		Melli+22
185:	23	3	21	1	23	22	2	20	1	22	99203.3210	-0.0097	0.030		Melli+22
186:	22	4	19	1	22	21	4	18	1	21	99225.7390	-0.0343	0.030		Melli+22
187:	21	8	14	1	21	20	8	13	1	20	99509.4750	-0.0132	0.030		Melli+22
188:	21	8	13	1	21	20	8	12	1	20	99577.4840	0.0519	0.030		Melli+22
189:	22	3	19	1	22	21	3	18	1	21	99620.0270	-0.0126	0.030		Melli+22
190:	21	6	16	1	21	20	6	15	1	20	99996.9300	0.0201	0.030		Melli+22
191:	21	4	17	1	21	20	4	16	1	20	101443.9730	0.0293	0.030		Melli+22
192:	22	5	18	1	22	21	5	17	1	21	102667.4810	0.0104	0.030		Melli+22
193:	23	3	20	1	23	22	4	19	1	22	102790.1210	0.0205	0.030		Melli+22
194:	26	0	26	1	26	25	0	25	1	25	102952.5020	0.0173	0.030	0.0180 0.25	Melli+22
195:	26	1	26	1	26	25	1	25	1	25	102952.5020	0.0185	0.030	0.0180 0.25	Melli+22
196:	26	1	26	1	26	25	0	25	1	25	102952.5020	0.0162	0.030	0.0180 0.25	Melli+22
197:	26	0	26	1	26	25	1	25	1	25	102952.5020	0.0196	0.030	0.0180 0.25	Melli+22
198:	24	2	22	1	24	23	3	21	1	23	103029.8580	0.0272	0.030		Melli+22
199:	24	3	22	1	24	23	3	21	1	23	103040.8830	0.0270	0.030		Melli+22
200:	24	2	22	1	24	23	2	21	1	23	103050.2680	0.0027	0.030		Melli+22

201:	24	3	22	1	24	23	2	21	1	23	103061.2940	0.0035	0.030			Melli+22
202:	22	12	10	1	22	21	12	9	1	21	103135.5510	-0.0239	0.030	-0.0235	0.50	Melli+22
203:	22	12	11	1	22	21	12	10	1	21	103135.5510	-0.0231	0.030	-0.0235	0.50	Melli+22
204:	22	11	12	1	22	21	11	11	1	21	103320.7450	-0.0328	0.030	-0.0453	0.50	Melli+22
205:	22	11	11	1	22	21	11	10	1	21	103320.7450	-0.0578	0.030	-0.0453	0.50	Melli+22
206:	23	3	20	1	23	22	3	19	1	22	103377.9020	-0.0040	0.030			Melli+22
207:	23	4	20	1	23	22	3	19	1	22	103723.4690	-0.0000	0.030			Melli+22
208:	22	9	14	1	22	21	9	13	1	21	103944.6570	0.0019	0.030			Melli+22
209:	22	9	13	1	22	21	9	12	1	21	103955.1300	-0.0135	0.030			Melli+22
210:	22	8	15	1	22	21	8	14	1	21	104450.4050	0.0123	0.030			Melli+22
211:	22	4	18	1	22	21	4	17	1	21	105018.9750	0.0421	0.030			Melli+22
212:	24	3	21	1	24	23	4	20	1	23	106826.4580	0.0189	0.030			Melli+22
213:	27	1	27	1	27	26	1	26	1	26	106831.9130	-0.0169	0.030	-0.0173	0.25	Melli+22
214:	27	1	27	1	27	26	0	26	1	26	106831.9130	-0.0180	0.030	-0.0173	0.25	Melli+22
215:	27	0	27	1	27	26	1	26	1	26	106831.9130	-0.0164	0.030	-0.0173	0.25	Melli+22
216:	27	0	27	1	27	26	0	26	1	26	106831.9130	-0.0175	0.030	-0.0173	0.25	Melli+22
217:	25	2	23	1	25	24	3	22	1	24	106909.1990	0.0051	0.030			Melli+22
218:	25	3	23	1	25	24	3	22	1	24	106915.0960	-0.0005	0.030			Melli+22
219:	25	2	23	1	25	24	2	22	1	24	106920.2200	0.0009	0.030			Melli+22
220:	25	3	23	1	25	24	2	22	1	24	106926.1280	0.0062	0.030			Melli+22
221:	24	4	21	1	24	23	4	20	1	23	107026.5220	-0.0022	0.030			Melli+22
222:	24	3	21	1	24	23	3	20	1	23	107172.0050	0.0029	0.030			Melli+22
223:	23	10	14	1	23	22	10	13	1	22	108426.6880	-0.0390	0.030			Melli+22
224:	23	10	13	1	23	22	10	12	1	22	108428.0890	-0.0510	0.030			Melli+22
225:	23	4	19	1	23	22	4	18	1	22	108526.7040	0.0242	0.030			Melli+22
226:	23	6	18	1	23	22	6	17	1	22	109194.5900	0.0120	0.030			Melli+22
227:	28	1	28	1	28	27	1	27	1	27	110710.8090	-0.0107	0.030	-0.0109	0.25	Melli+22
228:	28	1	28	1	28	27	0	27	1	27	110710.8090	-0.0112	0.030	-0.0109	0.25	Melli+22
229:	28	0	28	1	28	27	1	27	1	27	110710.8090	-0.0104	0.030	-0.0109	0.25	Melli+22
230:	28	0	28	1	28	27	0	27	1	27	110710.8090	-0.0110	0.030	-0.0109	0.25	Melli+22
231:	27	2	26	1	27	26	2	25	1	26	110734.7180	0.0153	0.030	-0.0066	0.25	Melli+22
232:	27	2	26	1	27	26	1	25	1	26	110734.7180	-0.0731	0.030	-0.0066	0.25	Melli+22
233:	27	1	26	1	27	26	2	25	1	26	110734.7180	0.0598	0.030	-0.0066	0.25	Melli+22
234:	27	1	26	1	27	26	1	25	1	26	110734.7180	-0.0286	0.030	-0.0066	0.25	Melli+22
235:	26	2	24	1	26	25	3	23	1	25	110785.7730	-0.0117	0.030			Melli+22
236:	26	3	24	1	26	25	3	23	1	25	110788.9220	-0.0011	0.030			Melli+22
237:	25	3	22	1	25	24	4	21	1	24	110791.1560	-0.0036	0.030			Melli+22
238:	26	3	24	1	26	25	2	23	1	25	110794.8070	-0.0188	0.030			Melli+22
239:	30	1	30	1	30	29	1	29	1	29	118466.8410	-0.0004	0.030	-0.0005	0.25	Melli+22
240:	30	1	30	1	30	29	0	29	1	29	118466.8410	-0.0006	0.030	-0.0005	0.25	Melli+22
241:	30	0	30	1	30	29	1	29	1	29	118466.8410	-0.0004	0.030	-0.0005	0.25	Melli+22
242:	30	0	30	1	30	29	0	29	1	29	118466.8410	-0.0005	0.030	-0.0005	0.25	Melli+22
243:	29	2	28	1	29	28	2	27	1	28	118489.0590	0.0021	0.030	-0.0034	0.25	Melli+22
244:	29	2	28	1	29	28	1	27	1	28	118489.0590	-0.0201	0.030	-0.0034	0.25	Melli+22
245:	29	1	28	1	29	28	2	27	1	28	118489.0590	0.0133	0.030	-0.0034	0.25	Melli+22
246:	29	1	28	1	29	28	1	27	1	28	118489.0590	-0.0089	0.030	-0.0034	0.25	Melli+22
247:	28	3	26	1	28	27	2	25	1	27	118537.5980	0.0132	0.030			Melli+22
248:	28	2	26	1	28	27	3	25	1	27	118535.0550	0.0002	0.030			Melli+22
249:	28	3	26	1	28	27	3	25	1	27	118535.9230	-0.0033	0.030			Melli+22
250:	28	2	26	1	28	27	2	25	1	27	118536.7210	0.0077	0.030			Melli+22
251:	63	0	63	1	63	62	1	62	1	62	245959.1500	-0.0057	0.030	-0.0058	0.25	Melli+22
252:	63	1	63	1	63	62	0	62	1	62	245959.1500	-0.0057	0.030	-0.0058	0.25	Melli+22
253:	63	1	63	1	63	62	1	62	1	62	245959.1500	-0.0057	0.030	-0.0058	0.25	Melli+22
254:	63	0	63	1	63	62	0	62	1	62	245959.1500	-0.0057	0.030	-0.0058	0.25	Melli+22
255:	62	2	61	1	62	61	1	60	1	61	245972.2260	-0.0185	0.030	-0.0185	0.25	Melli+22
256:	62	1	61	1	62	61	2	60	1	61	245972.2260	-0.0185	0.030	-0.0185	0.25	Melli+22
257:	62	2	61	1	62	61	2	60	1	61	245972.2260	-0.0185	0.030	-0.0185	0.25	Melli+22
258:	62	1	61	1	62	61	1	60	1	61	245972.2260	-0.0185	0.030	-0.0185	0.25	Melli+22
259:	61	3	59	1	61	60	2	58	1	60	245983.5170	-0.0043	0.030	-0.0043	0.25	Melli+22
260:	61	2	59	1	61	60	3	58	1	60	245983.5170	-0.0043	0.030	-0.0043	0.25	Melli+22
261:	61	2	59	1	61	60	2	58	1	60	245983.5170	-0.0043	0.030	-0.0043	0.25	Melli+22
262:	61	3	59	1	61	60	3	58	1	60	245983.5170	-0.0043	0.030	-0.0043	0.25	Melli+22
263:	60	4	57	1	60	59	3	56	1	59	245997.5410	0.0073	0.030	0.0073	0.25	Melli+22
264:	60	3	57	1	60	59	4	56	1	59	245997.5410	0.0073	0.030	0.0073	0.25	Melli+22
265:	60	3	57	1	60	59	3	56	1	59	245997.5410	0.0073	0.030	0.0073	0.25	Melli+22
266:	60	4	57	1	60	59	4	56	1	59	245997.5410	0.0073	0.030	0.0073	0.25	Melli+22
267:	59	5	55	1	59	58	4	54	1	58	246020.7310	-0.0251	0.030	-0.0251	0.25	Melli+22
268:	59	4	55	1	59	58	5	54	1	58	246020.7310	-0.0251	0.030	-0.0251	0.25	Melli+22
269:	59	4	55	1	59	58	4	54	1	58	246020.7310	-0.0251	0.030	-0.0251	0.25	Melli+22

270:	59	5	55	1	59	58	5	54	1	58	246020.7310	-0.0251	0.030	-0.0251	0.25	Melli+22
271:	58	6	53	1	58	57	5	52	1	57	246062.7300	-0.0055	0.030	-0.0054	0.25	Melli+22
272:	58	5	53	1	58	57	6	52	1	57	246062.7300	-0.0053	0.030	-0.0054	0.25	Melli+22
273:	58	5	53	1	58	57	5	52	1	57	246062.7300	-0.0054	0.030	-0.0054	0.25	Melli+22
274:	58	6	53	1	58	57	6	52	1	57	246062.7300	-0.0054	0.030	-0.0054	0.25	Melli+22
275:	57	7	51	1	57	56	6	50	1	56	246138.1290	-0.0302	0.030	-0.0247	0.25	Melli+22
276:	57	6	51	1	57	56	7	50	1	56	246138.1290	-0.0191	0.030	-0.0247	0.25	Melli+22
277:	57	6	51	1	57	56	6	50	1	56	246138.1290	-0.0263	0.030	-0.0247	0.25	Melli+22
278:	57	7	51	1	57	56	7	50	1	56	246138.1290	-0.0230	0.030	-0.0247	0.25	Melli+22
279:	64	1	64	1	64	63	0	63	1	63	249804.2380	-0.0156	0.030	-0.0156	0.25	Melli+22
280:	64	0	64	1	64	63	1	63	1	63	249804.2380	-0.0156	0.030	-0.0156	0.25	Melli+22
281:	64	1	64	1	64	63	1	63	1	63	249804.2380	-0.0156	0.030	-0.0156	0.25	Melli+22
282:	64	0	64	1	64	63	0	63	1	63	249804.2380	-0.0156	0.030	-0.0156	0.25	Melli+22
283:	63	1	62	1	63	62	2	61	1	62	249817.2290	0.0044	0.030	0.0044	0.25	Melli+22
284:	63	2	62	1	63	62	1	61	1	62	249817.2290	0.0044	0.030	0.0044	0.25	Melli+22
285:	63	1	62	1	63	62	1	61	1	62	249817.2290	0.0044	0.030	0.0044	0.25	Melli+22
286:	63	2	62	1	63	62	2	61	1	62	249817.2290	0.0044	0.030	0.0044	0.25	Melli+22
287:	62	2	60	1	62	61	3	59	1	61	249828.1480	0.0157	0.030	0.0157	0.25	Melli+22
288:	62	3	60	1	62	61	2	59	1	61	249828.1480	0.0157	0.030	0.0157	0.25	Melli+22
289:	62	2	60	1	62	61	2	59	1	61	249828.1480	0.0157	0.030	0.0157	0.25	Melli+22
290:	62	3	60	1	62	61	3	59	1	61	249828.1480	0.0157	0.030	0.0157	0.25	Melli+22
291:	61	4	58	1	61	60	3	57	1	60	249841.3380	-0.0149	0.030	-0.0149	0.25	Melli+22
292:	61	3	58	1	61	60	4	57	1	60	249841.3380	-0.0149	0.030	-0.0149	0.25	Melli+22
293:	61	3	58	1	61	60	3	57	1	60	249841.3380	-0.0149	0.030	-0.0149	0.25	Melli+22
294:	61	4	58	1	61	60	4	57	1	60	249841.3380	-0.0149	0.030	-0.0149	0.25	Melli+22
295:	60	5	56	1	60	59	4	55	1	59	249863.0710	0.0053	0.030	0.0053	0.25	Melli+22
296:	60	4	56	1	60	59	5	55	1	59	249863.0710	0.0053	0.030	0.0053	0.25	Melli+22
297:	60	4	56	1	60	59	4	55	1	59	249863.0710	0.0053	0.030	0.0053	0.25	Melli+22
298:	60	5	56	1	60	59	5	55	1	59	249863.0710	0.0053	0.030	0.0053	0.25	Melli+22
299:	59	6	54	1	59	58	5	53	1	58	249902.2850	-0.0138	0.030	-0.0138	0.25	Melli+22
300:	59	5	54	1	59	58	6	53	1	58	249902.2850	-0.0137	0.030	-0.0138	0.25	Melli+22
301:	59	5	54	1	59	58	5	53	1	58	249902.2850	-0.0138	0.030	-0.0138	0.25	Melli+22
302:	59	6	54	1	59	58	6	53	1	58	249902.2850	-0.0138	0.030	-0.0138	0.25	Melli+22
303:	58	7	52	1	58	57	6	51	1	57	249972.7440	-0.0471	0.030	-0.0440	0.25	Melli+22
304:	58	6	52	1	58	57	7	51	1	57	249972.7440	-0.0409	0.030	-0.0440	0.25	Melli+22
305:	58	6	52	1	58	57	6	51	1	57	249972.7440	-0.0449	0.030	-0.0440	0.25	Melli+22
306:	58	7	52	1	58	57	7	51	1	57	249972.7440	-0.0431	0.030	-0.0440	0.25	Melli+22
307:	65	1	65	1	65	64	0	64	1	64	253648.0220	-0.0218	0.030	-0.0219	0.25	Melli+22
308:	65	0	65	1	65	64	1	64	1	64	253648.0220	-0.0218	0.030	-0.0219	0.25	Melli+22
309:	65	0	65	1	65	64	0	64	1	64	253648.0220	-0.0218	0.030	-0.0219	0.25	Melli+22
310:	65	1	65	1	65	64	1	64	1	64	253648.0220	-0.0218	0.030	-0.0219	0.25	Melli+22
311:	64	1	63	1	64	63	2	62	1	63	253660.8770	-0.0228	0.030	-0.0228	0.25	Melli+22
312:	64	2	63	1	64	63	1	62	1	63	253660.8770	-0.0228	0.030	-0.0228	0.25	Melli+22
313:	64	2	63	1	64	63	2	62	1	63	253660.8770	-0.0228	0.030	-0.0228	0.25	Melli+22
314:	64	1	63	1	64	63	1	62	1	63	253660.8770	-0.0228	0.030	-0.0228	0.25	Melli+22
315:	63	2	61	1	63	62	3	60	1	62	253671.4860	0.0372	0.030	0.0372	0.25	Melli+22
316:	63	3	61	1	63	62	2	60	1	62	253671.4860	0.0372	0.030	0.0372	0.25	Melli+22
317:	63	3	61	1	63	62	3	60	1	62	253671.4860	0.0372	0.030	0.0372	0.25	Melli+22
318:	63	2	61	1	63	62	2	60	1	62	253671.4860	0.0372	0.030	0.0372	0.25	Melli+22
319:	62	4	59	1	62	61	3	58	1	61	253683.9370	0.0305	0.030	0.0306	0.25	Melli+22
320:	62	3	59	1	62	61	4	58	1	61	253683.9370	0.0305	0.030	0.0306	0.25	Melli+22
321:	62	3	59	1	62	61	3	58	1	61	253683.9370	0.0305	0.030	0.0306	0.25	Melli+22
322:	62	4	59	1	62	61	4	58	1	61	253683.9370	0.0305	0.030	0.0306	0.25	Melli+22
323:	61	5	57	1	61	60	4	56	1	60	253704.1730	-0.0049	0.030	-0.0049	0.25	Melli+22
324:	61	4	57	1	61	60	5	56	1	60	253704.1730	-0.0049	0.030	-0.0049	0.25	Melli+22
325:	61	4	57	1	61	60	4	56	1	60	253704.1730	-0.0049	0.030	-0.0049	0.25	Melli+22
326:	61	5	57	1	61	60	5	56	1	60	253704.1730	-0.0049	0.030	-0.0049	0.25	Melli+22
327:	60	6	55	1	60	59	5	54	1	59	253740.7820	-0.0331	0.030	-0.0331	0.25	Melli+22
328:	60	5	55	1	60	59	6	54	1	59	253740.7820	-0.0331	0.030	-0.0331	0.25	Melli+22
329:	60	5	55	1	60	59	5	54	1	59	253740.7820	-0.0331	0.030	-0.0331	0.25	Melli+22
330:	60	6	55	1	60	59	6	54	1	59	253740.7820	-0.0331	0.030	-0.0331	0.25	Melli+22
331:	59	7	53	1	59	58	6	52	1	58	253806.6980	0.0009	0.030	0.0026	0.25	Melli+22
332:	59	6	53	1	59	58	7	52	1	58	253806.6980	0.0042	0.030	0.0026	0.25	Melli+22
333:	59	6	53	1	59	58	6	52	1	58	253806.6980	0.0020	0.030	0.0026	0.25	Melli+22
334:	59	7	53	1	59	58	7	52	1	58	253806.6980	0.0030	0.030	0.0026	0.25	Melli+22
335:	58	8	51	1	58	57	7	50	1	57	253922.2070	-0.0357	0.030	0.0228	0.25	Melli+22
336:	58	7	51	1	58	57	8	50	1	57	253922.2070	0.0814	0.030	0.0228	0.25	Melli+22
337:	58	7	51	1	58	57	7	50	1	57	253922.2070	0.0063	0.030	0.0228	0.25	Melli+22
338:	58	8	51	1	58	57	8	50	1	57	253922.2070	0.0393	0.030	0.0228	0.25	Melli+22

339:	57	8	49	1	57	56	9	48	1	56	254120.1760	0.0482	0.030			Melli+22
340:	57	9	49	1	57	56	9	48	1	56	254121.2840	-0.0015	0.030			Melli+22
341:	57	8	49	1	57	56	8	48	1	56	254122.1870	0.0583	0.030			Melli+22
342:	57	9	49	1	57	56	8	48	1	56	254123.2920	0.0055	0.030			Melli+22
343:	66	0	66	1	66	65	0	65	1	65	257490.4840	-0.0230	0.030			Melli+22
344:	65	2	64	1	65	64	1	63	1	64	257503.2600	0.0092	0.030	0.0092	0.25	Melli+22
345:	65	1	64	1	65	64	2	63	1	64	257503.2600	0.0092	0.030	0.0092	0.25	Melli+22
346:	65	2	64	1	65	64	2	63	1	64	257503.2600	0.0092	0.030	0.0092	0.25	Melli+22
347:	65	1	64	1	65	64	1	63	1	64	257503.2600	0.0092	0.030	0.0092	0.25	Melli+22
348:	64	2	62	1	64	63	3	61	1	63	257513.4430	-0.0075	0.030	-0.0075	0.25	Melli+22
349:	64	3	62	1	64	63	2	61	1	63	257513.4430	-0.0075	0.030	-0.0075	0.25	Melli+22
350:	64	3	62	1	64	63	3	61	1	63	257513.4430	-0.0075	0.030	-0.0075	0.25	Melli+22
351:	64	2	62	1	64	63	2	61	1	63	257513.4430	-0.0075	0.030	-0.0075	0.25	Melli+22
352:	63	3	60	1	63	62	3	59	1	62	257525.1880	0.0163	0.030	0.0163	0.50	Melli+22
353:	63	4	60	1	63	62	4	59	1	62	257525.1880	0.0163	0.030	0.0163	0.50	Melli+22
354:	61	6	56	1	61	60	5	55	1	60	257578.2060	-0.0371	0.030	-0.0372	0.25	Melli+22
355:	61	5	56	1	61	60	6	55	1	60	257578.2060	-0.0371	0.030	-0.0372	0.25	Melli+22
356:	61	5	56	1	61	60	5	55	1	60	257578.2060	-0.0371	0.030	-0.0372	0.25	Melli+22
357:	61	6	56	1	61	60	6	55	1	60	257578.2060	-0.0371	0.030	-0.0372	0.25	Melli+22
358:	60	7	54	1	60	59	6	53	1	59	257639.7460	-0.0583	0.030	-0.0574	0.25	Melli+22
359:	60	6	54	1	60	59	7	53	1	59	257639.7460	-0.0565	0.030	-0.0574	0.25	Melli+22
360:	60	6	54	1	60	59	6	53	1	59	257639.7460	-0.0576	0.030	-0.0574	0.25	Melli+22
361:	60	7	54	1	60	59	7	53	1	59	257639.7460	-0.0571	0.030	-0.0574	0.25	Melli+22
362:	59	8	52	1	59	58	7	51	1	58	257747.6250	-0.0526	0.030	-0.0198	0.25	Melli+22
363:	59	7	52	1	59	58	8	51	1	58	257747.6250	0.0130	0.030	-0.0198	0.25	Melli+22
364:	59	7	52	1	59	58	7	51	1	58	257747.6250	-0.0290	0.030	-0.0198	0.25	Melli+22
365:	59	8	52	1	59	58	8	51	1	58	257747.6250	-0.0105	0.030	-0.0198	0.25	Melli+22
366:	67	0	67	1	67	66	0	66	1	66	261331.6210	-0.0027	0.030			Melli+22
367:	66	1	65	1	66	65	1	64	1	65	261344.2390	-0.0188	0.030	-0.0188	0.25	Melli+22
368:	66	2	65	1	66	65	2	64	1	65	261344.2390	-0.0188	0.030	-0.0188	0.25	Melli+22
369:	66	2	65	1	66	65	1	64	1	65	261344.2390	-0.0188	0.030	-0.0188	0.25	Melli+22
370:	66	1	65	1	66	65	2	64	1	65	261344.2390	-0.0188	0.030	-0.0188	0.25	Melli+22
371:	65	2	63	1	65	64	2	62	1	64	261354.1030	-0.0142	0.030	-0.0143	0.25	Melli+22
372:	65	3	63	1	65	64	3	62	1	64	261354.1030	-0.0142	0.030	-0.0143	0.25	Melli+22
373:	65	2	63	1	65	64	3	62	1	64	261354.1030	-0.0142	0.030	-0.0143	0.25	Melli+22
374:	65	3	63	1	65	64	2	62	1	64	261354.1030	-0.0142	0.030	-0.0143	0.25	Melli+22
375:	64	4	61	1	64	63	4	60	1	63	261365.1640	0.0374	0.030	0.0374	0.50	Melli+22
376:	64	3	61	1	64	63	3	60	1	63	261365.1640	0.0374	0.030	0.0374	0.50	Melli+22
377:	63	5	59	1	63	62	4	58	1	62	261382.6950	-0.0041	0.030	-0.0042	0.25	Melli+22
378:	63	4	59	1	63	62	5	58	1	62	261382.6950	-0.0041	0.030	-0.0042	0.25	Melli+22
379:	63	4	59	1	63	62	4	58	1	62	261382.6950	-0.0041	0.030	-0.0042	0.25	Melli+22
380:	63	5	59	1	63	62	5	58	1	62	261382.6950	-0.0041	0.030	-0.0042	0.25	Melli+22
381:	62	6	57	1	62	61	5	56	1	61	261414.5200	-0.0239	0.030	-0.0240	0.25	Melli+22
382:	62	5	57	1	62	61	6	56	1	61	261414.5200	-0.0239	0.030	-0.0240	0.25	Melli+22
383:	62	5	57	1	62	61	5	56	1	61	261414.5200	-0.0239	0.030	-0.0240	0.25	Melli+22
384:	62	6	57	1	62	61	6	56	1	61	261414.5200	-0.0239	0.030	-0.0240	0.25	Melli+22
385:	61	7	55	1	61	60	6	54	1	60	261472.0470	0.0008	0.030	0.0014	0.25	Melli+22
386:	61	6	55	1	61	60	7	54	1	60	261472.0470	0.0018	0.030	0.0014	0.25	Melli+22
387:	61	6	55	1	61	60	6	54	1	60	261472.0470	0.0012	0.030	0.0014	0.25	Melli+22
388:	61	7	55	1	61	60	7	54	1	60	261472.0470	0.0015	0.030	0.0014	0.25	Melli+22
389:	60	8	53	1	60	59	7	52	1	59	261572.7550	-0.0500	0.030	-0.0316	0.25	Melli+22
390:	60	7	53	1	60	59	8	52	1	59	261572.7550	-0.0133	0.030	-0.0316	0.25	Melli+22
391:	60	7	53	1	60	59	7	52	1	59	261572.7550	-0.0368	0.030	-0.0316	0.25	Melli+22
392:	60	8	53	1	60	59	8	52	1	59	261572.7550	-0.0264	0.030	-0.0316	0.25	Melli+22
393:	68	0	68	1	68	67	0	67	1	67	265171.3800	0.0053	0.030			Melli+22
394:	67	1	66	1	67	66	2	65	1	66	265183.8930	-0.0085	0.030	-0.0085	0.25	Melli+22
395:	67	2	66	1	67	66	1	65	1	66	265183.8930	-0.0085	0.030	-0.0085	0.25	Melli+22
396:	67	2	66	1	67	66	2	65	1	66	265183.8930	-0.0085	0.030	-0.0085	0.25	Melli+22
397:	67	1	66	1	67	66	1	65	1	66	265183.8930	-0.0085	0.030	-0.0085	0.25	Melli+22
398:	66	2	64	1	66	65	3	63	1	65	265193.4150	-0.0139	0.030	-0.0140	0.25	Melli+22
399:	66	3	64	1	66	65	2	63	1	65	265193.4150	-0.0139	0.030	-0.0140	0.25	Melli+22
400:	66	2	64	1	66	65	2	63	1	65	265193.4150	-0.0139	0.030	-0.0140	0.25	Melli+22
401:	66	3	64	1	66	65	3	63	1	65	265193.4150	-0.0139	0.030	-0.0140	0.25	Melli+22
402:	65	4	62	1	65	64	4	61	1	64	265203.7660	0.0168	0.030	0.0168	0.50	Melli+22
403:	65	3	62	1	65	64	3	61	1	64	265203.7660	0.0168	0.030	0.0168	0.50	Melli+22
404:	63	6	58	1	63	62	5	57	1	62	265249.7010	0.0204	0.030	0.0204	0.25	Melli+22
405:	63	5	58	1	63	62	6	57	1	62	265249.7010	0.0204	0.030	0.0204	0.25	Melli+22
406:	63	5	58	1	63	62	5	57	1	62	265249.7010	0.0204	0.030	0.0204	0.25	Melli+22
407:	63	6	58	1	63	62	6	57	1	62	265249.7010	0.0204	0.030	0.0204	0.25	Melli+22

408:	62	7	56	1	62	61	6	55	1	61	265303.3520	-0.0091	0.030	-0.0089	0.25	Melli+22
409:	62	6	56	1	62	61	7	55	1	61	265303.3520	-0.0086	0.030	-0.0089	0.25	Melli+22
410:	62	6	56	1	62	61	6	55	1	61	265303.3520	-0.0090	0.030	-0.0089	0.25	Melli+22
411:	62	7	56	1	62	61	7	55	1	61	265303.3520	-0.0088	0.030	-0.0089	0.25	Melli+22
412:	61	8	54	1	61	60	7	53	1	60	265397.4710	-0.0307	0.030	-0.0205	0.25	Melli+22
413:	61	7	54	1	61	60	8	53	1	60	265397.4710	-0.0103	0.030	-0.0205	0.25	Melli+22
414:	61	7	54	1	61	60	7	53	1	60	265397.4710	-0.0234	0.030	-0.0205	0.25	Melli+22
415:	61	8	54	1	61	60	8	53	1	60	265397.4710	-0.0176	0.030	-0.0205	0.25	Melli+22
416:	69	0	69	1	69	68	0	68	1	68	269009.7310	-0.0094	0.030			Melli+22
417:	68	1	67	1	68	67	1	66	1	67	269022.1560	-0.0063	0.030	-0.0064	0.25	Melli+22
418:	68	2	67	1	68	67	2	66	1	67	269022.1560	-0.0063	0.030	-0.0064	0.25	Melli+22
419:	68	1	67	1	68	67	2	66	1	67	269022.1560	-0.0063	0.030	-0.0064	0.25	Melli+22
420:	68	2	67	1	68	67	1	66	1	67	269022.1560	-0.0063	0.030	-0.0064	0.25	Melli+22
421:	67	3	65	1	67	66	3	64	1	66	269031.3720	0.0064	0.030	0.0064	0.25	Melli+22
422:	67	2	65	1	67	66	2	64	1	66	269031.3720	0.0064	0.030	0.0064	0.25	Melli+22
423:	67	2	65	1	67	66	3	64	1	66	269031.3720	0.0064	0.030	0.0064	0.25	Melli+22
424:	67	3	65	1	67	66	2	64	1	66	269031.3720	0.0064	0.030	0.0064	0.25	Melli+22
425:	66	3	63	1	66	65	3	62	1	65	269041.0020	-0.0158	0.030	-0.0159	0.50	Melli+22
426:	66	4	63	1	66	65	4	62	1	65	269041.0020	-0.0158	0.030	-0.0159	0.50	Melli+22
427:	65	4	61	1	65	64	4	60	1	64	269056.1260	0.0204	0.030			Melli+22
428:	64	6	59	1	64	63	5	58	1	63	269083.6080	-0.0098	0.030	-0.0098	0.25	Melli+22
429:	64	5	59	1	64	63	6	58	1	63	269083.6080	-0.0098	0.030	-0.0098	0.25	Melli+22
430:	64	5	59	1	64	63	5	58	1	63	269083.6080	-0.0098	0.030	-0.0098	0.25	Melli+22
431:	64	6	59	1	64	63	6	58	1	63	269083.6080	-0.0098	0.030	-0.0098	0.25	Melli+22
432:	63	7	57	1	63	62	6	56	1	62	269133.6970	0.0042	0.030	0.0044	0.25	Melli+22
433:	63	6	57	1	63	62	7	56	1	62	269133.6970	0.0045	0.030	0.0044	0.25	Melli+22
434:	63	6	57	1	63	62	6	56	1	62	269133.6970	0.0043	0.030	0.0044	0.25	Melli+22
435:	63	7	57	1	63	62	7	56	1	62	269133.6970	0.0044	0.030	0.0044	0.25	Melli+22
436:	62	8	55	1	62	61	7	54	1	61	269221.6710	0.0121	0.030	0.0179	0.25	Melli+22
437:	62	7	55	1	62	61	8	54	1	61	269221.6710	0.0235	0.030	0.0179	0.25	Melli+22
438:	62	7	55	1	62	61	7	54	1	61	269221.6710	0.0162	0.030	0.0179	0.25	Melli+22
439:	62	8	55	1	62	61	8	54	1	61	269221.6710	0.0194	0.030	0.0179	0.25	Melli+22
440:	70	0	70	1	70	69	0	69	1	69	272846.6800	-0.0218	0.030			Melli+22
441:	69	1	68	1	69	68	1	67	1	68	272859.0250	0.0039	0.030	0.0039	0.25	Melli+22
442:	69	1	68	1	69	68	2	67	1	68	272859.0250	0.0039	0.030	0.0039	0.25	Melli+22
443:	69	2	68	1	69	68	1	67	1	68	272859.0250	0.0039	0.030	0.0039	0.25	Melli+22
444:	69	2	68	1	69	68	2	67	1	68	272859.0250	0.0039	0.030	0.0039	0.25	Melli+22
445:	68	3	66	1	68	67	3	65	1	67	272867.9300	0.0227	0.030	0.0227	0.25	Melli+22
446:	68	2	66	1	68	67	3	65	1	67	272867.9300	0.0227	0.030	0.0227	0.25	Melli+22
447:	68	3	66	1	68	67	2	65	1	67	272867.9300	0.0227	0.030	0.0227	0.25	Melli+22
448:	68	2	66	1	68	67	2	65	1	67	272867.9300	0.0227	0.030	0.0227	0.25	Melli+22
449:	67	4	64	1	67	66	4	63	1	66	272876.9350	0.0237	0.030	0.0237	0.25	Melli+22
450:	67	3	64	1	67	66	3	63	1	66	272876.9350	0.0237	0.030	0.0237	0.25	Melli+22
451:	67	3	64	1	67	66	4	63	1	66	272876.9350	0.0237	0.030	0.0237	0.25	Melli+22
452:	67	4	64	1	67	66	3	63	1	66	272876.9350	0.0237	0.030	0.0237	0.25	Melli+22
453:	66	4	62	1	66	65	4	61	1	65	272890.8370	0.0101	0.030	0.0102	0.25	Melli+22
454:	66	5	62	1	66	65	5	61	1	65	272890.8370	0.0101	0.030	0.0102	0.25	Melli+22
455:	66	5	62	1	66	65	4	61	1	65	272890.8370	0.0101	0.030	0.0102	0.25	Melli+22
456:	66	4	62	1	66	65	5	61	1	65	272890.8370	0.0101	0.030	0.0102	0.25	Melli+22
457:	65	5	60	1	65	64	5	59	1	64	272916.3400	0.0178	0.030	0.0179	0.25	Melli+22
458:	65	6	60	1	65	64	6	59	1	64	272916.3400	0.0178	0.030	0.0179	0.25	Melli+22
459:	65	6	60	1	65	64	5	59	1	64	272916.3400	0.0178	0.030	0.0179	0.25	Melli+22
460:	65	5	60	1	65	64	6	59	1	64	272916.3400	0.0178	0.030	0.0179	0.25	Melli+22
461:	64	7	58	1	64	63	6	57	1	63	272962.9810	-0.0070	0.030	-0.0070	0.25	Melli+22
462:	64	6	58	1	64	63	7	57	1	63	272962.9810	-0.0069	0.030	-0.0070	0.25	Melli+22
463:	64	6	58	1	64	63	6	57	1	63	272962.9810	-0.0070	0.030	-0.0070	0.25	Melli+22
464:	64	7	58	1	64	63	7	57	1	63	272962.9810	-0.0069	0.030	-0.0070	0.25	Melli+22
465:	63	8	56	1	63	62	7	55	1	62	273045.1870	0.0084	0.030	0.0116	0.25	Melli+22
466:	63	7	56	1	63	62	8	55	1	62	273045.1870	0.0148	0.030	0.0116	0.25	Melli+22
467:	63	7	56	1	63	62	7	55	1	62	273045.1870	0.0107	0.030	0.0116	0.25	Melli+22
468:	63	8	56	1	63	62	8	55	1	62	273045.1870	0.0125	0.030	0.0116	0.25	Melli+22
469:	62	9	54	1	62	61	8	53	1	61	273185.4750	-0.0858	0.030	0.0128	0.25	Melli+22
470:	62	8	54	1	62	61	9	53	1	61	273185.4750	0.1114	0.030	0.0128	0.25	Melli+22
471:	62	8	54	1	62	61	8	53	1	61	273185.4750	-0.0142	0.030	0.0128	0.25	Melli+22
472:	62	9	54	1	62	61	9	53	1	61	273185.4750	0.0398	0.030	0.0128	0.25	Melli+22
473:	61	9	52	1	61	60	10	51	1	60	273419.5020	0.0200	0.030			Melli+22
474:	61	10	52	1	61	60	10	51	1	60	273421.2970	0.0372	0.030			Melli+22
475:	61	9	52	1	61	60	9	51	1	60	273422.5360	0.0280	0.030			Melli+22
476:	61	10	52	1	61	60	9	51	1	60	273424.3130	0.0273	0.030			Melli+22

477:	71	0	71	1	71	70	0	70	1	70	276682.2110	-0.0287	0.030			Melli+22
478:	70	1	69	1	70	69	1	68	1	69	276694.4560	-0.0022	0.030	-0.0022	0.25	Melli+22
479:	70	2	69	1	70	69	2	68	1	69	276694.4560	-0.0022	0.030	-0.0022	0.25	Melli+22
480:	70	2	69	1	70	69	1	68	1	69	276694.4560	-0.0022	0.030	-0.0022	0.25	Melli+22
481:	70	1	69	1	70	69	2	68	1	69	276694.4560	-0.0022	0.030	-0.0022	0.25	Melli+22
482:	69	3	67	1	69	68	3	66	1	68	276703.0700	0.0358	0.030	0.0358	0.25	Melli+22
483:	69	2	67	1	69	68	2	66	1	68	276703.0700	0.0358	0.030	0.0358	0.25	Melli+22
484:	69	2	67	1	69	68	3	66	1	68	276703.0700	0.0358	0.030	0.0358	0.25	Melli+22
485:	69	3	67	1	69	68	2	66	1	68	276703.0700	0.0358	0.030	0.0358	0.25	Melli+22
486:	68	4	65	1	68	67	4	64	1	67	276711.4640	0.0557	0.030	0.0557	0.25	Melli+22
487:	68	3	65	1	68	67	3	64	1	67	276711.4640	0.0557	0.030	0.0557	0.25	Melli+22
488:	68	4	65	1	68	67	3	64	1	67	276711.4640	0.0557	0.030	0.0557	0.25	Melli+22
489:	68	3	65	1	68	67	4	64	1	67	276711.4640	0.0557	0.030	0.0557	0.25	Melli+22
490:	67	5	63	1	67	66	4	62	1	66	276724.1090	-0.0848	0.030	-0.0848	0.25	Melli+22
491:	67	4	63	1	67	66	5	62	1	66	276724.1090	-0.0848	0.030	-0.0848	0.25	Melli+22
492:	67	4	63	1	67	66	4	62	1	66	276724.1090	-0.0848	0.030	-0.0848	0.25	Melli+22
493:	67	5	63	1	67	66	5	62	1	66	276724.1090	-0.0848	0.030	-0.0848	0.25	Melli+22
494:	66	6	61	1	66	65	5	60	1	65	276747.7330	-0.0282	0.030	-0.0282	0.25	Melli+22
495:	66	5	61	1	66	65	6	60	1	65	276747.7330	-0.0282	0.030	-0.0282	0.25	Melli+22
496:	66	5	61	1	66	65	5	60	1	65	276747.7330	-0.0282	0.030	-0.0282	0.25	Melli+22
497:	66	6	61	1	66	65	6	60	1	65	276747.7330	-0.0282	0.030	-0.0282	0.25	Melli+22
498:	65	7	59	1	65	64	6	58	1	64	276791.1790	-0.0188	0.030	-0.0188	0.25	Melli+22
499:	65	6	59	1	65	64	7	58	1	64	276791.1790	-0.0187	0.030	-0.0188	0.25	Melli+22
500:	65	6	59	1	65	64	6	58	1	64	276791.1790	-0.0188	0.030	-0.0188	0.25	Melli+22
501:	65	7	59	1	65	64	7	58	1	64	276791.1790	-0.0188	0.030	-0.0188	0.25	Melli+22
502:	64	8	57	1	64	63	7	56	1	63	276867.9180	-0.0545	0.030	-0.0528	0.25	Melli+22
503:	64	7	57	1	64	63	8	56	1	63	276867.9180	-0.0510	0.030	-0.0528	0.25	Melli+22
504:	64	7	57	1	64	63	7	56	1	63	276867.9180	-0.0533	0.030	-0.0528	0.25	Melli+22
505:	64	8	57	1	64	63	8	56	1	63	276867.9180	-0.0523	0.030	-0.0528	0.25	Melli+22
506:	63	9	55	1	63	62	8	54	1	62	276999.0180	-0.0525	0.030	0.0036	0.25	Melli+22
507:	63	8	55	1	63	62	9	54	1	62	276999.0180	0.0597	0.030	0.0036	0.25	Melli+22
508:	63	8	55	1	63	62	8	54	1	62	276999.0180	-0.0118	0.030	0.0036	0.25	Melli+22
509:	63	9	55	1	63	62	9	54	1	62	276999.0180	0.0190	0.030	0.0036	0.25	Melli+22
510:	72	0	72	1	72	71	0	71	1	71	280516.3150	-0.0197	0.030			Melli+22
511:	71	2	70	1	71	70	2	69	1	70	280528.4470	-0.0075	0.030	-0.0076	0.25	Melli+22
512:	71	1	70	1	71	70	1	69	1	70	280528.4470	-0.0075	0.030	-0.0076	0.25	Melli+22
513:	71	2	70	1	71	70	1	69	1	70	280528.4470	-0.0075	0.030	-0.0076	0.25	Melli+22
514:	71	1	70	1	71	70	2	69	1	70	280528.4470	-0.0075	0.030	-0.0076	0.25	Melli+22
515:	70	3	68	1	70	69	3	67	1	69	280536.7410	0.0143	0.030	0.0144	0.25	Melli+22
516:	70	2	68	1	70	69	2	67	1	69	280536.7410	0.0143	0.030	0.0144	0.25	Melli+22
517:	70	2	68	1	70	69	3	67	1	69	280536.7410	0.0143	0.030	0.0144	0.25	Melli+22
518:	70	3	68	1	70	69	2	67	1	69	280536.7410	0.0143	0.030	0.0144	0.25	Melli+22
519:	69	3	66	1	69	68	3	65	1	68	280544.5170	0.0291	0.030	0.0292	0.25	Melli+22
520:	69	4	66	1	69	68	4	65	1	68	280544.5170	0.0291	0.030	0.0292	0.25	Melli+22
521:	69	3	66	1	69	68	4	65	1	68	280544.5170	0.0291	0.030	0.0292	0.25	Melli+22
522:	69	4	66	1	69	68	3	65	1	68	280544.5170	0.0291	0.030	0.0292	0.25	Melli+22
523:	68	4	64	1	68	67	4	63	1	67	280556.1960	0.0134	0.030	0.0135	0.25	Melli+22
524:	68	5	64	1	68	67	5	63	1	67	280556.1960	0.0134	0.030	0.0135	0.25	Melli+22
525:	68	5	64	1	68	67	4	63	1	67	280556.1960	0.0134	0.030	0.0135	0.25	Melli+22
526:	68	4	64	1	68	67	5	63	1	67	280556.1960	0.0134	0.030	0.0135	0.25	Melli+22
527:	67	5	62	1	67	66	5	61	1	66	280577.9050	0.0008	0.030	0.0009	0.25	Melli+22
528:	67	6	62	1	67	66	6	61	1	66	280577.9050	0.0008	0.030	0.0009	0.25	Melli+22
529:	67	6	62	1	67	66	5	61	1	66	280577.9050	0.0008	0.030	0.0009	0.25	Melli+22
530:	67	5	62	1	67	66	6	61	1	66	280577.9050	0.0008	0.030	0.0009	0.25	Melli+22
531:	66	6	60	1	66	65	6	59	1	65	280618.2680	-0.0079	0.030	-0.0080	0.25	Melli+22
532:	66	7	60	1	66	65	7	59	1	65	280618.2680	-0.0079	0.030	-0.0080	0.25	Melli+22
533:	66	7	60	1	66	65	6	59	1	65	280618.2680	-0.0080	0.030	-0.0080	0.25	Melli+22
534:	66	6	60	1	66	65	7	59	1	65	280618.2680	-0.0079	0.030	-0.0080	0.25	Melli+22
535:	65	7	58	1	65	64	7	57	1	64	280689.9480	-0.0120	0.030	-0.0118	0.25	Melli+22
536:	65	8	58	1	65	64	8	57	1	64	280689.9480	-0.0115	0.030	-0.0118	0.25	Melli+22
537:	65	8	58	1	65	64	7	57	1	64	280689.9480	-0.0127	0.030	-0.0118	0.25	Melli+22
538:	65	7	58	1	65	64	8	57	1	64	280689.9480	-0.0108	0.030	-0.0118	0.25	Melli+22
539:	64	9	56	1	64	63	8	55	1	63	280812.3670	-0.0791	0.030	-0.0472	0.25	Melli+22
540:	64	8	56	1	64	63	9	55	1	63	280812.3670	-0.0153	0.030	-0.0472	0.25	Melli+22
541:	64	8	56	1	64	63	8	55	1	63	280812.3670	-0.0560	0.030	-0.0472	0.25	Melli+22
542:	64	9	56	1	64	63	9	55	1	63	280812.3670	-0.0384	0.030	-0.0472	0.25	Melli+22
543:	73	0	73	1	73	72	0	72	1	72	284348.9690	0.0011	0.030			Melli+22
544:	71	2	69	1	71	70	2	68	1	70	284368.9740	0.0090	0.030	0.0090	0.50	Melli+22
545:	71	3	69	1	71	70	3	68	1	70	284368.9740	0.0090	0.030	0.0090	0.50	Melli+22

546:	72	2	71	1 72	71	1	70	1 71	284361.0030	0.0121	0.030	0.0121	0.25	Melli+22
547:	72	1	71	1 72	71	2	70	1 71	284361.0030	0.0121	0.030	0.0121	0.25	Melli+22
548:	72	1	71	1 72	71	1	70	1 71	284361.0030	0.0121	0.030	0.0121	0.25	Melli+22
549:	72	2	71	1 72	71	2	70	1 71	284361.0030	0.0121	0.030	0.0121	0.25	Melli+22
550:	71	3	69	1 71	70	2	68	1 70	284368.9740	0.0090	0.030	0.0090	0.50	Melli+22
551:	71	2	69	1 71	70	3	68	1 70	284368.9740	0.0090	0.030	0.0090	0.50	Melli+22
552:	70	3	67	1 70	69	3	66	1 69	284376.1330	0.0038	0.030	0.0039	0.25	Melli+22
553:	70	4	67	1 70	69	4	66	1 69	284376.1330	0.0038	0.030	0.0039	0.25	Melli+22
554:	70	4	67	1 70	69	3	66	1 69	284376.1330	0.0038	0.030	0.0039	0.25	Melli+22
555:	70	3	67	1 70	69	4	66	1 69	284376.1330	0.0038	0.030	0.0039	0.25	Melli+22
556:	69	4	65	1 69	68	4	64	1 68	284386.7980	0.0286	0.030	0.0286	0.25	Melli+22
557:	69	5	65	1 69	68	5	64	1 68	284386.7980	0.0286	0.030	0.0286	0.25	Melli+22
558:	69	5	65	1 69	68	4	64	1 68	284386.7980	0.0286	0.030	0.0286	0.25	Melli+22
559:	69	4	65	1 69	68	5	64	1 68	284386.7980	0.0286	0.030	0.0286	0.25	Melli+22
560:	68	5	63	1 68	67	5	62	1 67	284406.7170	-0.0040	0.030	-0.0040	0.25	Melli+22
561:	68	6	63	1 68	67	6	62	1 67	284406.7170	-0.0040	0.030	-0.0040	0.25	Melli+22
562:	68	6	63	1 68	67	5	62	1 67	284406.7170	-0.0040	0.030	-0.0040	0.25	Melli+22
563:	68	5	63	1 68	67	6	62	1 67	284406.7170	-0.0040	0.030	-0.0040	0.25	Melli+22
564:	67	6	61	1 67	66	6	60	1 66	284444.1820	0.0030	0.030	0.0031	0.25	Melli+22
565:	67	7	61	1 67	66	7	60	1 66	284444.1820	0.0030	0.030	0.0031	0.25	Melli+22
566:	67	7	61	1 67	66	6	60	1 66	284444.1820	0.0030	0.030	0.0031	0.25	Melli+22
567:	67	6	61	1 67	66	7	60	1 66	284444.1820	0.0031	0.030	0.0031	0.25	Melli+22
568:	66	8	59	1 66	65	7	58	1 65	284511.0560	-0.0135	0.030	-0.0130	0.25	Melli+22
569:	66	7	59	1 66	65	8	58	1 65	284511.0560	-0.0124	0.030	-0.0130	0.25	Melli+22
570:	66	7	59	1 66	65	7	58	1 65	284511.0560	-0.0131	0.030	-0.0130	0.25	Melli+22
571:	66	8	59	1 66	65	8	58	1 65	284511.0560	-0.0128	0.030	-0.0130	0.25	Melli+22
572:	65	9	57	1 65	64	8	56	1 64	284625.5310	-0.0082	0.030	0.0099	0.25	Melli+22
573:	65	8	57	1 65	64	9	56	1 64	284625.5310	0.0279	0.030	0.0099	0.25	Melli+22
574:	65	8	57	1 65	64	8	56	1 64	284625.5310	0.0048	0.030	0.0099	0.25	Melli+22
575:	65	9	57	1 65	64	9	56	1 64	284625.5310	0.0148	0.030	0.0099	0.25	Melli+22
576:	74	0	74	1 74	73	0	73	1 73	288180.1360	0.0160	0.030			Melli+22
577:	72	3	70	1 72	71	3	69	1 71	288199.7530	0.0232	0.030			Melli+22
578:	73	2	72	1 73	72	1	71	1 72	288192.0520	0.0039	0.030	0.0040	0.25	Melli+22
579:	73	1	72	1 73	72	2	71	1 72	288192.0520	0.0039	0.030	0.0040	0.25	Melli+22
580:	73	1	72	1 73	72	1	71	1 72	288192.0520	0.0039	0.030	0.0040	0.25	Melli+22
581:	73	2	72	1 73	72	2	71	1 72	288192.0520	0.0039	0.030	0.0040	0.25	Melli+22
582:	72	3	70	1 72	71	2	69	1 71	288199.7530	0.0232	0.030	0.0233	0.33	Melli+22
583:	72	2	70	1 72	71	3	69	1 71	288199.7530	0.0232	0.030	0.0233	0.33	Melli+22
584:	72	2	70	1 72	71	2	69	1 71	288199.7530	0.0232	0.030	0.0233	0.33	Melli+22
585:	71	4	68	1 71	70	4	67	1 70	288206.3300	0.0183	0.030	0.0183	0.25	Melli+22
586:	71	4	68	1 71	70	3	67	1 70	288206.3300	0.0183	0.030	0.0183	0.25	Melli+22
587:	71	3	68	1 71	70	4	67	1 70	288206.3300	0.0183	0.030	0.0183	0.25	Melli+22
588:	71	3	68	1 71	70	3	67	1 70	288206.3300	0.0183	0.030	0.0183	0.25	Melli+22
589:	70	5	66	1 70	69	5	65	1 69	288215.9360	0.0047	0.030	0.0048	0.25	Melli+22
590:	70	4	66	1 70	69	5	65	1 69	288215.9360	0.0047	0.030	0.0048	0.25	Melli+22
591:	70	5	66	1 70	69	4	65	1 69	288215.9360	0.0047	0.030	0.0048	0.25	Melli+22
592:	70	4	66	1 70	69	4	65	1 69	288215.9360	0.0047	0.030	0.0048	0.25	Melli+22
593:	69	6	64	1 69	68	5	63	1 68	288234.1070	-0.0760	0.030	-0.0760	0.25	Melli+22
594:	69	5	64	1 69	68	6	63	1 68	288234.1070	-0.0760	0.030	-0.0760	0.25	Melli+22
595:	69	5	64	1 69	68	5	63	1 68	288234.1070	-0.0760	0.030	-0.0760	0.25	Melli+22
596:	69	6	64	1 69	68	6	63	1 68	288234.1070	-0.0760	0.030	-0.0760	0.25	Melli+22
597:	68	7	62	1 68	67	6	61	1 67	288268.7960	-0.0695	0.030	-0.0695	0.25	Melli+22
598:	68	6	62	1 68	67	7	61	1 67	288268.7960	-0.0695	0.030	-0.0695	0.25	Melli+22
599:	68	6	62	1 68	67	6	61	1 67	288268.7960	-0.0695	0.030	-0.0695	0.25	Melli+22
600:	68	7	62	1 68	67	7	61	1 67	288268.7960	-0.0695	0.030	-0.0695	0.25	Melli+22
601:	67	8	60	1 67	66	7	59	1 66	288331.2010	-0.0303	0.030	-0.0300	0.25	Melli+22
602:	67	7	60	1 67	66	8	59	1 66	288331.2010	-0.0297	0.030	-0.0300	0.25	Melli+22
603:	67	7	60	1 67	66	7	59	1 66	288331.2010	-0.0301	0.030	-0.0300	0.25	Melli+22
604:	67	8	60	1 67	66	8	59	1 66	288331.2010	-0.0299	0.030	-0.0300	0.25	Melli+22
605:	66	9	58	1 66	65	8	57	1 65	288438.1990	-0.0200	0.030	-0.0098	0.25	Melli+22
606:	66	8	58	1 66	65	9	57	1 65	288438.1990	0.0004	0.030	-0.0098	0.25	Melli+22
607:	66	8	58	1 66	65	8	57	1 65	288438.1990	-0.0126	0.030	-0.0098	0.25	Melli+22
608:	66	9	58	1 66	65	9	57	1 65	288438.1990	-0.0070	0.030	-0.0098	0.25	Melli+22
609:	75	1	75	1 75	74	0	74	1 74	292009.7840	0.0119	0.030	0.0120	0.25	Melli+22
610:	75	0	75	1 75	74	1	74	1 74	292009.7840	0.0119	0.030	0.0120	0.25	Melli+22
611:	75	0	75	1 75	74	0	74	1 74	292009.7840	0.0119	0.030	0.0120	0.25	Melli+22
612:	75	1	75	1 75	74	1	74	1 74	292009.7840	0.0119	0.030	0.0120	0.25	Melli+22
613:	74	1	73	1 74	73	2	72	1 73	292021.6470	0.0401	0.030	0.0402	0.25	Melli+22
614:	74	2	73	1 74	73	1	72	1 73	292021.6470	0.0401	0.030	0.0402	0.25	Melli+22

615:	74	2	73	1	74	73	2	72	1	73	292021.6470	0.0401	0.030	0.0402	0.25	Melli+22
616:	74	1	73	1	74	73	1	72	1	73	292021.6470	0.0401	0.030	0.0402	0.25	Melli+22
617:	73	2	71	1	73	72	3	70	1	72	292029.0750	0.0736	0.030	0.0737	0.25	Melli+22
618:	73	3	71	1	73	72	2	70	1	72	292029.0750	0.0736	0.030	0.0737	0.25	Melli+22
619:	73	2	71	1	73	72	2	70	1	72	292029.0750	0.0736	0.030	0.0737	0.25	Melli+22
620:	73	3	71	1	73	72	3	70	1	72	292029.0750	0.0736	0.030	0.0737	0.25	Melli+22
621:	71	5	67	1	71	70	4	66	1	70	292043.6880	0.0427	0.030	0.0428	0.25	Melli+22
622:	71	4	67	1	71	70	5	66	1	70	292043.6880	0.0427	0.030	0.0428	0.25	Melli+22
623:	71	4	67	1	71	70	4	66	1	70	292043.6880	0.0427	0.030	0.0428	0.25	Melli+22
624:	71	5	67	1	71	70	5	66	1	70	292043.6880	0.0427	0.030	0.0428	0.25	Melli+22
625:	70	6	65	1	70	69	5	64	1	69	292060.2920	0.0298	0.030	0.0298	0.25	Melli+22
626:	70	5	65	1	70	69	6	64	1	69	292060.2920	0.0298	0.030	0.0298	0.25	Melli+22
627:	70	5	65	1	70	69	5	64	1	69	292060.2920	0.0298	0.030	0.0298	0.25	Melli+22
628:	70	6	65	1	70	69	6	64	1	69	292060.2920	0.0298	0.030	0.0298	0.25	Melli+22
629:	69	7	63	1	69	68	6	62	1	68	292092.3250	0.0281	0.030	0.0282	0.25	Melli+22
630:	69	6	63	1	69	68	7	62	1	68	292092.3250	0.0281	0.030	0.0282	0.25	Melli+22
631:	69	6	63	1	69	68	6	62	1	68	292092.3250	0.0281	0.030	0.0282	0.25	Melli+22
632:	69	7	63	1	69	68	7	62	1	68	292092.3250	0.0281	0.030	0.0282	0.25	Melli+22
633:	68	8	61	1	68	67	7	60	1	67	292150.4380	0.0544	0.030	0.0546	0.25	Melli+22
634:	68	7	61	1	68	67	8	60	1	67	292150.4380	0.0547	0.030	0.0546	0.25	Melli+22
635:	68	7	61	1	68	67	7	60	1	67	292150.4380	0.0545	0.030	0.0546	0.25	Melli+22
636:	68	8	61	1	68	67	8	60	1	67	292150.4380	0.0546	0.030	0.0546	0.25	Melli+22
637:	67	9	59	1	67	66	8	58	1	66	292250.3960	0.0267	0.030	0.0325	0.25	Melli+22
638:	67	8	59	1	67	66	9	58	1	66	292250.3960	0.0383	0.030	0.0325	0.25	Melli+22
639:	67	8	59	1	67	66	8	58	1	66	292250.3960	0.0309	0.030	0.0325	0.25	Melli+22
640:	67	9	59	1	67	66	9	58	1	66	292250.3960	0.0341	0.030	0.0325	0.25	Melli+22
641:	76	0	76	1	76	75	1	75	1	75	295837.8950	-0.0100	0.030	-0.0100	0.25	Melli+22
642:	76	1	76	1	76	75	0	75	1	75	295837.8950	-0.0100	0.030	-0.0100	0.25	Melli+22
643:	76	1	76	1	76	75	1	75	1	75	295837.8950	-0.0100	0.030	-0.0100	0.25	Melli+22
644:	76	0	76	1	76	75	0	75	1	75	295837.8950	-0.0100	0.030	-0.0100	0.25	Melli+22
645:	75	1	74	1	75	74	2	73	1	74	295849.6520	0.0037	0.030	0.0037	0.25	Melli+22
646:	75	2	74	1	75	74	1	73	1	74	295849.6520	0.0037	0.030	0.0037	0.25	Melli+22
647:	75	1	74	1	75	74	1	73	1	74	295849.6520	0.0037	0.030	0.0037	0.25	Melli+22
648:	75	2	74	1	75	74	2	73	1	74	295849.6520	0.0037	0.030	0.0037	0.25	Melli+22
649:	74	2	72	1	74	73	3	71	1	73	295856.7610	0.0005	0.030	0.0005	0.25	Melli+22
650:	74	3	72	1	74	73	2	71	1	73	295856.7610	0.0005	0.030	0.0005	0.25	Melli+22
651:	74	3	72	1	74	73	3	71	1	73	295856.7610	0.0005	0.030	0.0005	0.25	Melli+22
652:	74	2	72	1	74	73	2	71	1	73	295856.7610	0.0005	0.030	0.0005	0.25	Melli+22
653:	73	3	70	1	73	72	4	69	1	72	295862.2260	0.0072	0.030	0.0073	0.25	Melli+22
654:	73	4	70	1	73	72	3	69	1	72	295862.2260	0.0072	0.030	0.0073	0.25	Melli+22
655:	73	4	70	1	73	72	4	69	1	72	295862.2260	0.0072	0.030	0.0073	0.25	Melli+22
656:	73	3	70	1	73	72	3	69	1	72	295862.2260	0.0072	0.030	0.0073	0.25	Melli+22
657:	72	5	68	1	72	71	4	67	1	71	295869.9020	0.0129	0.030	0.0130	0.25	Melli+22
658:	72	4	68	1	72	71	5	67	1	71	295869.9020	0.0129	0.030	0.0130	0.25	Melli+22
659:	72	4	68	1	72	71	4	67	1	71	295869.9020	0.0129	0.030	0.0130	0.25	Melli+22
660:	72	5	68	1	72	71	5	67	1	71	295869.9020	0.0129	0.030	0.0130	0.25	Melli+22
661:	71	6	66	1	71	70	5	65	1	70	295884.9260	-0.0052	0.030	-0.0053	0.25	Melli+22
662:	71	5	66	1	71	70	6	65	1	70	295884.9260	-0.0052	0.030	-0.0053	0.25	Melli+22
663:	71	5	66	1	71	70	5	65	1	70	295884.9260	-0.0052	0.030	-0.0053	0.25	Melli+22
664:	71	6	66	1	71	70	6	65	1	70	295884.9260	-0.0052	0.030	-0.0053	0.25	Melli+22
665:	70	7	64	1	70	69	6	63	1	69	295914.4330	-0.0026	0.030	-0.0027	0.25	Melli+22
666:	70	6	64	1	70	69	7	63	1	69	295914.4330	-0.0026	0.030	-0.0027	0.25	Melli+22
667:	70	6	64	1	70	69	6	63	1	69	295914.4330	-0.0026	0.030	-0.0027	0.25	Melli+22
668:	70	7	64	1	70	69	7	63	1	69	295914.4330	-0.0026	0.030	-0.0027	0.25	Melli+22
669:	69	8	62	1	69	68	7	61	1	68	295968.3820	-0.0860	0.030	-0.0860	0.25	Melli+22
670:	69	7	62	1	69	68	8	61	1	68	295968.3820	-0.0859	0.030	-0.0860	0.25	Melli+22
671:	69	7	62	1	69	68	7	61	1	68	295968.3820	-0.0860	0.030	-0.0860	0.25	Melli+22
672:	69	8	62	1	69	68	8	61	1	68	295968.3820	-0.0859	0.030	-0.0860	0.25	Melli+22
673:	68	9	60	1	68	67	8	59	1	67	296061.8440	-0.0409	0.030	-0.0377	0.25	Melli+22
674:	68	8	60	1	68	67	9	59	1	67	296061.8440	-0.0344	0.030	-0.0377	0.25	Melli+22
675:	68	8	60	1	68	67	8	59	1	67	296061.8440	-0.0386	0.030	-0.0377	0.25	Melli+22
676:	68	9	60	1	68	67	9	59	1	67	296061.8440	-0.0367	0.030	-0.0377	0.25	Melli+22
677:	77	0	77	1	77	76	1	76	1	76	299664.4550	-0.0450	0.030	-0.0450	0.25	Melli+22
678:	77	1	77	1	77	76	0	76	1	76	299664.4550	-0.0450	0.030	-0.0450	0.25	Melli+22
679:	77	1	77	1	77	76	1	76	1	76	299664.4550	-0.0450	0.030	-0.0450	0.25	Melli+22
680:	77	0	77	1	77	76	0	76	1	76	299664.4550	-0.0450	0.030	-0.0450	0.25	Melli+22
681:	76	2	75	1	76	75	1	74	1	75	299676.1690	0.0157	0.030	0.0158	0.25	Melli+22
682:	76	1	75	1	76	75	2	74	1	75	299676.1690	0.0157	0.030	0.0158	0.25	Melli+22
683:	76	1	75	1	76	75	1	74	1	75	299676.1690	0.0157	0.030	0.0158	0.25	Melli+22

684:	76	2	75	1	76	75	2	74	1	75	299676.1690	0.0157	0.030	0.0158	0.25	Melli+22
685:	74	4	71	1	74	73	3	70	1	73	299687.9230	0.0202	0.030	0.0202	0.25	Melli+22
686:	74	3	71	1	74	73	4	70	1	73	299687.9230	0.0202	0.030	0.0202	0.25	Melli+22
687:	74	3	71	1	74	73	3	70	1	73	299687.9230	0.0202	0.030	0.0202	0.25	Melli+22
688:	74	4	71	1	74	73	4	70	1	73	299687.9230	0.0202	0.030	0.0202	0.25	Melli+22
689:	75	3	73	1	75	74	2	72	1	74	299683.0140	0.0261	0.030	0.0262	0.25	Melli+22
690:	75	2	73	1	75	74	3	72	1	74	299683.0140	0.0261	0.030	0.0262	0.25	Melli+22
691:	75	3	73	1	75	74	3	72	1	74	299683.0140	0.0261	0.030	0.0262	0.25	Melli+22
692:	75	2	73	1	75	74	2	72	1	74	299683.0140	0.0261	0.030	0.0262	0.25	Melli+22
693:	72	6	67	1	72	71	5	66	1	71	299708.1660	0.0021	0.030	0.0021	0.25	Melli+22
694:	72	5	67	1	72	71	6	66	1	71	299708.1660	0.0021	0.030	0.0021	0.25	Melli+22
695:	72	5	67	1	72	71	5	66	1	71	299708.1660	0.0021	0.030	0.0021	0.25	Melli+22
696:	72	6	67	1	72	71	6	66	1	71	299708.1660	0.0021	0.030	0.0021	0.25	Melli+22
697:	71	7	65	1	71	70	6	64	1	70	299735.2290	-0.0176	0.030	-0.0176	0.25	Melli+22
698:	71	6	65	1	71	70	7	64	1	70	299735.2290	-0.0176	0.030	-0.0176	0.25	Melli+22
699:	71	6	65	1	71	70	6	64	1	70	299735.2290	-0.0176	0.030	-0.0176	0.25	Melli+22
700:	71	7	65	1	71	70	7	64	1	70	299735.2290	-0.0176	0.030	-0.0176	0.25	Melli+22
701:	70	8	63	1	70	69	7	62	1	69	299785.3970	-0.0337	0.030	-0.0337	0.25	Melli+22
702:	70	7	63	1	70	69	8	62	1	69	299785.3970	-0.0336	0.030	-0.0337	0.25	Melli+22
703:	70	7	63	1	70	69	7	62	1	69	299785.3970	-0.0337	0.030	-0.0337	0.25	Melli+22
704:	70	8	63	1	70	69	8	62	1	69	299785.3970	-0.0336	0.030	-0.0337	0.25	Melli+22
705:	68	10	59	1	68	67	9	58	1	67	300018.3260	-0.0659	0.030	-0.0119	0.25	Melli+22
706:	68	9	59	1	68	67	10	58	1	67	300018.3260	0.0420	0.030	-0.0119	0.25	Melli+22
707:	68	9	59	1	68	67	9	58	1	67	300018.3260	-0.0264	0.030	-0.0119	0.25	Melli+22
708:	68	10	59	1	68	67	10	58	1	67	300018.3260	0.0025	0.030	-0.0119	0.25	Melli+22
709:	78	1	78	1	78	77	0	77	1	77	303489.5070	-0.0311	0.030	-0.0312	0.25	Melli+22
710:	78	0	78	1	78	77	1	77	1	77	303489.5070	-0.0311	0.030	-0.0312	0.25	Melli+22
711:	78	0	78	1	78	77	0	77	1	77	303489.5070	-0.0311	0.030	-0.0312	0.25	Melli+22
712:	78	1	78	1	78	77	1	77	1	77	303489.5070	-0.0311	0.030	-0.0312	0.25	Melli+22
713:	76	2	74	1	76	75	3	73	1	75	303507.6960	0.0318	0.030	0.0319	0.25	Melli+22
714:	76	3	74	1	76	75	2	73	1	75	303507.6960	0.0318	0.030	0.0319	0.25	Melli+22
715:	76	3	74	1	76	75	3	73	1	75	303507.6960	0.0318	0.030	0.0319	0.25	Melli+22
716:	76	2	74	1	76	75	2	73	1	75	303507.6960	0.0318	0.030	0.0319	0.25	Melli+22
717:	77	2	76	1	77	76	1	75	1	76	303501.0960	-0.0068	0.030	-0.0069	0.25	Melli+22
718:	77	1	76	1	77	76	2	75	1	76	303501.0960	-0.0068	0.030	-0.0069	0.25	Melli+22
719:	77	1	76	1	77	76	1	75	1	76	303501.0960	-0.0068	0.030	-0.0069	0.25	Melli+22
720:	77	2	76	1	77	76	2	75	1	76	303501.0960	-0.0068	0.030	-0.0069	0.25	Melli+22
721:	74	5	70	1	74	73	4	69	1	73	303517.8970	0.0194	0.030	0.0195	0.25	Melli+22
722:	74	4	70	1	74	73	5	69	1	73	303517.8970	0.0194	0.030	0.0195	0.25	Melli+22
723:	74	4	70	1	74	73	4	69	1	73	303517.8970	0.0194	0.030	0.0195	0.25	Melli+22
724:	74	5	70	1	74	73	5	69	1	73	303517.8970	0.0194	0.030	0.0195	0.25	Melli+22
725:	75	3	72	1	75	74	4	71	1	74	303512.0770	0.0298	0.030	0.0299	0.25	Melli+22
726:	75	4	72	1	75	74	3	71	1	74	303512.0770	0.0298	0.030	0.0299	0.25	Melli+22
727:	75	3	72	1	75	74	3	71	1	74	303512.0770	0.0298	0.030	0.0299	0.25	Melli+22
728:	75	4	72	1	75	74	4	71	1	74	303512.0770	0.0298	0.030	0.0299	0.25	Melli+22
729:	72	7	66	1	72	71	6	65	1	71	303554.7030	0.0072	0.030	0.0073	0.25	Melli+22
730:	72	6	66	1	72	71	7	65	1	71	303554.7030	0.0072	0.030	0.0073	0.25	Melli+22
731:	72	6	66	1	72	71	6	65	1	71	303554.7030	0.0072	0.030	0.0073	0.25	Melli+22
732:	72	7	66	1	72	71	7	65	1	71	303554.7030	0.0072	0.030	0.0073	0.25	Melli+22
733:	71	8	64	1	71	70	7	63	1	70	303601.2280	0.0072	0.030	0.0073	0.25	Melli+22
734:	71	7	64	1	71	70	8	63	1	70	303601.2280	0.0073	0.030	0.0073	0.25	Melli+22
735:	71	7	64	1	71	70	7	63	1	70	303601.2280	0.0072	0.030	0.0073	0.25	Melli+22
736:	71	8	64	1	71	70	8	63	1	70	303601.2280	0.0073	0.030	0.0073	0.25	Melli+22
737:	69	10	60	1	69	68	9	59	1	68	303818.8610	-0.0257	0.030	0.0053	0.25	Melli+22
738:	69	9	60	1	69	68	10	59	1	68	303818.8610	0.0364	0.030	0.0053	0.25	Melli+22
739:	69	9	60	1	69	68	9	59	1	68	303818.8610	-0.0030	0.030	0.0053	0.25	Melli+22
740:	69	10	60	1	69	68	10	59	1	68	303818.8610	0.0137	0.030	0.0053	0.25	Melli+22
741:	79	1	79	1	79	78	0	78	1	78	307312.9950	-0.0055	0.030	-0.0055	0.25	Melli+22
742:	79	0	79	1	79	78	1	78	1	78	307312.9950	-0.0055	0.030	-0.0055	0.25	Melli+22
743:	79	1	79	1	79	78	1	78	1	78	307312.9950	-0.0055	0.030	-0.0055	0.25	Melli+22
744:	79	0	79	1	79	78	0	78	1	78	307312.9950	-0.0055	0.030	-0.0055	0.25	Melli+22
745:	78	2	77	1	78	77	1	76	1	77	307324.4820	0.0037	0.030	0.0038	0.25	Melli+22
746:	78	1	77	1	78	77	2	76	1	77	307324.4820	0.0037	0.030	0.0038	0.25	Melli+22
747:	78	2	77	1	78	77	2	76	1	77	307324.4820	0.0037	0.030	0.0038	0.25	Melli+22
748:	78	1	77	1	78	77	1	76	1	77	307324.4820	0.0037	0.030	0.0038	0.25	Melli+22
749:	76	3	73	1	76	75	4	72	1	75	307334.6940	0.0621	0.030	0.0621	0.25	Melli+22
750:	76	4	73	1	76	75	3	72	1	75	307334.6940	0.0621	0.030	0.0621	0.25	Melli+22
751:	76	3	73	1	76	75	3	72	1	75	307334.6940	0.0621	0.030	0.0621	0.25	Melli+22
752:	76	4	73	1	76	75	4	72	1	75	307334.6940	0.0621	0.030	0.0621	0.25	Melli+22

753:	75	4	71	1	75	74	5	70	1	74	307339.6210	0.0421	0.030	0.0421	0.25	Melli+22
754:	75	5	71	1	75	74	4	70	1	74	307339.6210	0.0421	0.030	0.0421	0.25	Melli+22
755:	75	4	71	1	75	74	4	70	1	74	307339.6210	0.0421	0.030	0.0421	0.25	Melli+22
756:	75	5	71	1	75	74	5	70	1	74	307339.6210	0.0421	0.030	0.0421	0.25	Melli+22
757:	77	3	75	1	77	76	2	74	1	76	307330.7770	0.0067	0.030	0.0068	0.25	Melli+22
758:	77	2	75	1	77	76	3	74	1	76	307330.7770	0.0067	0.030	0.0068	0.25	Melli+22
759:	77	2	75	1	77	76	2	74	1	76	307330.7770	0.0067	0.030	0.0068	0.25	Melli+22
760:	77	3	75	1	77	76	3	74	1	76	307330.7770	0.0067	0.030	0.0068	0.25	Melli+22
761:	74	6	69	1	74	73	5	68	1	73	307350.2640	0.0467	0.030	0.0468	0.25	Melli+22
762:	74	5	69	1	74	73	6	68	1	73	307350.2640	0.0467	0.030	0.0468	0.25	Melli+22
763:	74	5	69	1	74	73	5	68	1	73	307350.2640	0.0467	0.030	0.0468	0.25	Melli+22
764:	74	6	69	1	74	73	6	68	1	73	307350.2640	0.0467	0.030	0.0468	0.25	Melli+22
765:	73	7	67	1	73	72	6	66	1	72	307372.7280	-0.0224	0.030	-0.0224	0.25	Melli+22
766:	73	6	67	1	73	72	7	66	1	72	307372.7280	-0.0224	0.030	-0.0224	0.25	Melli+22
767:	73	6	67	1	73	72	6	66	1	72	307372.7280	-0.0224	0.030	-0.0224	0.25	Melli+22
768:	73	7	67	1	73	72	7	66	1	72	307372.7280	-0.0224	0.030	-0.0224	0.25	Melli+22
769:	72	8	65	1	72	71	7	64	1	71	307415.7950	0.0047	0.030	0.0047	0.25	Melli+22
770:	72	7	65	1	72	71	8	64	1	71	307415.7950	0.0047	0.030	0.0047	0.25	Melli+22
771:	72	7	65	1	72	71	7	64	1	71	307415.7950	0.0047	0.030	0.0047	0.25	Melli+22
772:	72	8	65	1	72	71	8	64	1	71	307415.7950	0.0047	0.030	0.0047	0.25	Melli+22
773:	71	9	63	1	71	70	8	62	1	70	307491.7410	0.0243	0.030	0.0249	0.25	Melli+22
774:	71	8	63	1	71	70	9	62	1	70	307491.7410	0.0255	0.030	0.0249	0.25	Melli+22
775:	71	8	63	1	71	70	8	62	1	70	307491.7410	0.0247	0.030	0.0249	0.25	Melli+22
776:	71	9	63	1	71	70	9	62	1	70	307491.7410	0.0250	0.030	0.0249	0.25	Melli+22
777:	70	10	61	1	70	69	9	60	1	69	307619.1170	0.0027	0.030	0.0207	0.25	Melli+22
778:	70	9	61	1	70	69	10	60	1	69	307619.1170	0.0385	0.030	0.0207	0.25	Melli+22
779:	70	9	61	1	70	69	9	60	1	69	307619.1170	0.0158	0.030	0.0207	0.25	Melli+22
780:	70	10	61	1	70	69	10	60	1	69	307619.1170	0.0254	0.030	0.0207	0.25	Melli+22
781:	80	1	80	1	80	79	0	79	1	79	311134.8630	-0.0053	0.030	-0.0053	0.25	Melli+22
782:	80	0	80	1	80	79	1	79	1	79	311134.8630	-0.0053	0.030	-0.0053	0.25	Melli+22
783:	80	0	80	1	80	79	0	79	1	79	311134.8630	-0.0053	0.030	-0.0053	0.25	Melli+22
784:	80	1	80	1	80	79	1	79	1	79	311134.8630	-0.0053	0.030	-0.0053	0.25	Melli+22
785:	79	2	78	1	79	78	1	77	1	78	311146.2590	-0.0014	0.030	-0.0014	0.25	Melli+22
786:	79	1	78	1	79	78	2	77	1	78	311146.2590	-0.0014	0.030	-0.0014	0.25	Melli+22
787:	79	2	78	1	79	78	2	77	1	78	311146.2590	-0.0014	0.030	-0.0014	0.25	Melli+22
788:	79	1	78	1	79	78	1	77	1	78	311146.2590	-0.0014	0.030	-0.0014	0.25	Melli+22
789:	77	3	74	1	77	76	4	73	1	76	311155.6630	0.0256	0.030	0.0257	0.25	Melli+22
790:	77	4	74	1	77	76	3	73	1	76	311155.6630	0.0256	0.030	0.0257	0.25	Melli+22
791:	77	4	74	1	77	76	4	73	1	76	311155.6630	0.0256	0.030	0.0257	0.25	Melli+22
792:	77	3	74	1	77	76	3	73	1	76	311155.6630	0.0256	0.030	0.0257	0.25	Melli+22
793:	76	4	72	1	76	75	5	71	1	75	311159.7290	0.0058	0.030	0.0058	0.25	Melli+22
794:	76	5	72	1	76	75	4	71	1	75	311159.7290	0.0058	0.030	0.0058	0.25	Melli+22
795:	76	4	72	1	76	75	4	71	1	75	311159.7290	0.0058	0.030	0.0058	0.25	Melli+22
796:	76	5	72	1	76	75	5	71	1	75	311159.7290	0.0058	0.030	0.0058	0.25	Melli+22
797:	78	3	76	1	78	77	2	75	1	77	311152.2480	-0.0390	0.030	-0.0390	0.25	Melli+22
798:	78	2	76	1	78	77	3	75	1	77	311152.2480	-0.0390	0.030	-0.0390	0.25	Melli+22
799:	78	3	76	1	78	77	3	75	1	77	311152.2480	-0.0390	0.030	-0.0390	0.25	Melli+22
800:	78	2	76	1	78	77	2	75	1	77	311152.2480	-0.0390	0.030	-0.0390	0.25	Melli+22
801:	75	6	70	1	75	74	5	69	1	74	311169.0170	0.0288	0.030	0.0288	0.25	Melli+22
802:	75	5	70	1	75	74	6	69	1	74	311169.0170	0.0288	0.030	0.0288	0.25	Melli+22
803:	75	5	70	1	75	74	5	69	1	74	311169.0170	0.0288	0.030	0.0288	0.25	Melli+22
804:	75	6	70	1	75	74	6	69	1	74	311169.0170	0.0288	0.030	0.0288	0.25	Melli+22
805:	74	7	68	1	74	73	6	67	1	73	311189.3620	-0.0172	0.030	-0.0173	0.25	Melli+22
806:	74	6	68	1	74	73	7	67	1	73	311189.3620	-0.0172	0.030	-0.0173	0.25	Melli+22
807:	74	6	68	1	74	73	6	67	1	73	311189.3620	-0.0172	0.030	-0.0173	0.25	Melli+22
808:	74	7	68	1	74	73	7	67	1	73	311189.3620	-0.0172	0.030	-0.0173	0.25	Melli+22
809:	73	8	66	1	73	72	7	65	1	72	311229.0650	-0.0294	0.030	-0.0294	0.25	Melli+22
810:	73	7	66	1	73	72	8	65	1	72	311229.0650	-0.0293	0.030	-0.0294	0.25	Melli+22
811:	73	7	66	1	73	72	7	65	1	72	311229.0650	-0.0293	0.030	-0.0294	0.25	Melli+22
812:	73	8	66	1	73	72	8	65	1	72	311229.0650	-0.0293	0.030	-0.0294	0.25	Melli+22
813:	81	0	81	1	81	80	1	80	1	80	314955.0520	-0.0707	0.030	-0.0708	0.25	Melli+22
814:	81	1	81	1	81	80	0	80	1	80	314955.0520	-0.0707	0.030	-0.0708	0.25	Melli+22
815:	81	0	81	1	81	80	0	80	1	80	314955.0520	-0.0707	0.030	-0.0708	0.25	Melli+22
816:	81	1	81	1	81	80	1	80	1	80	314955.0520	-0.0707	0.030	-0.0708	0.25	Melli+22
817:	80	2	79	1	80	79	1	78	1	79	314966.3610	-0.0696	0.030	-0.0697	0.25	Melli+22
818:	80	1	79	1	80	79	2	78	1	79	314966.3610	-0.0696	0.030	-0.0697	0.25	Melli+22
819:	80	1	79	1	80	79	1	78	1	79	314966.3610	-0.0696	0.030	-0.0697	0.25	Melli+22
820:	80	2	79	1	80	79	2	78	1	79	314966.3610	-0.0696	0.030	-0.0697	0.25	Melli+22
821:	78	4	75	1	78	77	3	74	1	77	314975.0550	0.0112	0.030	0.0112	0.25	Melli+22

822:	78	3	75	1	78	77	4	74	1	77	314975.0550	0.0112	0.030	0.0112	0.25 Melli+22
823:	78	4	75	1	78	77	4	74	1	77	314975.0550	0.0112	0.030	0.0112	0.25 Melli+22
824:	78	3	75	1	78	77	3	74	1	77	314975.0550	0.0112	0.030	0.0112	0.25 Melli+22
825:	77	4	73	1	77	76	5	72	1	76	314978.2630	-0.0262	0.030	-0.0263	0.25 Melli+22
826:	77	5	73	1	77	76	4	72	1	76	314978.2630	-0.0262	0.030	-0.0263	0.25 Melli+22
827:	77	4	73	1	77	76	4	72	1	76	314978.2630	-0.0262	0.030	-0.0263	0.25 Melli+22
828:	77	5	73	1	77	76	5	72	1	76	314978.2630	-0.0262	0.030	-0.0263	0.25 Melli+22
829:	79	3	77	1	79	78	2	76	1	78	314972.2280	0.0324	0.030	0.0325	0.25 Melli+22
830:	79	2	77	1	79	78	3	76	1	78	314972.2280	0.0324	0.030	0.0325	0.25 Melli+22
831:	79	3	77	1	79	78	3	76	1	78	314972.2280	0.0324	0.030	0.0325	0.25 Melli+22
832:	79	2	77	1	79	78	2	76	1	78	314972.2280	0.0324	0.030	0.0325	0.25 Melli+22
833:	76	5	71	1	76	75	6	70	1	75	314986.1970	-0.0259	0.030	-0.0260	0.25 Melli+22
834:	76	6	71	1	76	75	5	70	1	75	314986.1970	-0.0259	0.030	-0.0260	0.25 Melli+22
835:	76	5	71	1	76	75	5	70	1	75	314986.1970	-0.0259	0.030	-0.0260	0.25 Melli+22
836:	76	6	71	1	76	75	6	70	1	75	314986.1970	-0.0259	0.030	-0.0260	0.25 Melli+22
837:	75	7	69	1	75	74	6	68	1	74	315004.4890	-0.0630	0.030	-0.0630	0.25 Melli+22
838:	75	6	69	1	75	74	7	68	1	74	315004.4890	-0.0630	0.030	-0.0630	0.25 Melli+22
839:	75	6	69	1	75	74	6	68	1	74	315004.4890	-0.0630	0.030	-0.0630	0.25 Melli+22
840:	75	7	69	1	75	74	7	68	1	74	315004.4890	-0.0630	0.030	-0.0630	0.25 Melli+22
841:	74	8	67	1	74	73	7	66	1	73	315041.0300	-0.0604	0.030	-0.0604	0.25 Melli+22
842:	74	7	67	1	74	73	8	66	1	73	315041.0300	-0.0604	0.030	-0.0604	0.25 Melli+22
843:	74	7	67	1	74	73	7	66	1	73	315041.0300	-0.0604	0.030	-0.0604	0.25 Melli+22
844:	74	8	67	1	74	73	8	66	1	73	315041.0300	-0.0604	0.030	-0.0604	0.25 Melli+22
845:	73	9	65	1	73	72	8	64	1	72	315106.8400	-0.0535	0.030	-0.0533	0.25 Melli+22
846:	73	8	65	1	73	72	9	64	1	72	315106.8400	-0.0531	0.030	-0.0533	0.25 Melli+22
847:	73	8	65	1	73	72	8	64	1	72	315106.8400	-0.0533	0.030	-0.0533	0.25 Melli+22
848:	73	9	65	1	73	72	9	64	1	72	315106.8400	-0.0532	0.030	-0.0533	0.25 Melli+22
849:	72	10	63	1	72	71	9	62	1	71	315218.2540	0.0198	0.030	0.0257	0.25 Melli+22
850:	72	9	63	1	72	71	10	62	1	71	315218.2540	0.0315	0.030	0.0257	0.25 Melli+22
851:	72	9	63	1	72	71	9	62	1	71	315218.2540	0.0241	0.030	0.0257	0.25 Melli+22
852:	72	10	63	1	72	71	10	62	1	71	315218.2540	0.0273	0.030	0.0257	0.25 Melli+22
853:	82	1	82	1	82	81	0	81	1	81	318773.7540	0.0087	0.030	0.0088	0.25 Melli+22
854:	82	0	82	1	82	81	1	81	1	81	318773.7540	0.0087	0.030	0.0088	0.25 Melli+22
855:	82	1	82	1	82	81	1	81	1	81	318773.7540	0.0087	0.030	0.0088	0.25 Melli+22
856:	82	0	82	1	82	81	0	81	1	81	318773.7540	0.0087	0.030	0.0088	0.25 Melli+22
857:	81	2	80	1	81	80	1	79	1	80	318784.9700	-0.0002	0.030	-0.0002	0.25 Melli+22
858:	81	1	80	1	81	80	2	79	1	80	318784.9700	-0.0002	0.030	-0.0002	0.25 Melli+22
859:	81	2	80	1	81	80	2	79	1	80	318784.9700	-0.0002	0.030	-0.0002	0.25 Melli+22
860:	81	1	80	1	81	80	1	79	1	80	318784.9700	-0.0002	0.030	-0.0002	0.25 Melli+22
861:	80	2	78	1	80	79	3	77	1	79	318790.4790	0.0022	0.030	0.0023	0.25 Melli+22
862:	80	3	78	1	80	79	2	77	1	79	318790.4790	0.0022	0.030	0.0023	0.25 Melli+22
863:	80	3	78	1	80	79	3	77	1	79	318790.4790	0.0022	0.030	0.0023	0.25 Melli+22
864:	80	2	78	1	80	79	2	77	1	79	318790.4790	0.0022	0.030	0.0023	0.25 Melli+22
865:	79	3	76	1	79	78	4	75	1	78	318792.8450	0.0133	0.030	0.0133	0.25 Melli+22
866:	79	4	76	1	79	78	3	75	1	78	318792.8450	0.0133	0.030	0.0133	0.25 Melli+22
867:	79	4	76	1	79	78	4	75	1	78	318792.8450	0.0133	0.030	0.0133	0.25 Melli+22
868:	79	3	76	1	79	78	3	75	1	78	318792.8450	0.0133	0.030	0.0133	0.25 Melli+22
869:	78	4	74	1	78	77	5	73	1	77	318795.2660	0.0096	0.030	0.0097	0.25 Melli+22
870:	78	5	74	1	78	77	4	73	1	77	318795.2660	0.0096	0.030	0.0097	0.25 Melli+22
871:	78	4	74	1	78	77	4	73	1	77	318795.2660	0.0096	0.030	0.0097	0.25 Melli+22
872:	78	5	74	1	78	77	5	73	1	77	318795.2660	0.0096	0.030	0.0097	0.25 Melli+22
873:	77	6	72	1	77	76	5	71	1	76	318801.8350	-0.0629	0.030	-0.0630	0.25 Melli+22
874:	77	5	72	1	77	76	6	71	1	76	318801.8350	-0.0629	0.030	-0.0630	0.25 Melli+22
875:	77	5	72	1	77	76	5	71	1	76	318801.8350	-0.0629	0.030	-0.0630	0.25 Melli+22
876:	77	6	72	1	77	76	6	71	1	76	318801.8350	-0.0629	0.030	-0.0630	0.25 Melli+22
877:	76	7	70	1	76	75	6	69	1	75	318818.2290	-0.0104	0.030	-0.0104	0.25 Melli+22
878:	76	6	70	1	76	75	7	69	1	75	318818.2290	-0.0104	0.030	-0.0104	0.25 Melli+22
879:	76	6	70	1	76	75	6	69	1	75	318818.2290	-0.0104	0.030	-0.0104	0.25 Melli+22
880:	76	7	70	1	76	75	7	69	1	75	318818.2290	-0.0104	0.030	-0.0104	0.25 Melli+22
881:	74	9	66	1	74	73	8	65	1	73	318912.8500	-0.0147	0.030	-0.0147	0.25 Melli+22
882:	74	8	66	1	74	73	9	65	1	73	318912.8500	-0.0145	0.030	-0.0147	0.25 Melli+22
883:	74	8	66	1	74	73	8	65	1	73	318912.8500	-0.0147	0.030	-0.0147	0.25 Melli+22
884:	74	9	66	1	74	73	9	65	1	73	318912.8500	-0.0146	0.030	-0.0147	0.25 Melli+22
885:	73	10	64	1	73	72	9	63	1	72	319016.8920	-0.0015	0.030	0.0018	0.25 Melli+22
886:	73	9	64	1	73	72	10	63	1	72	319016.8920	0.0051	0.030	0.0018	0.25 Melli+22
887:	73	9	64	1	73	72	9	63	1	72	319016.8920	0.0008	0.030	0.0018	0.25 Melli+22
888:	73	10	64	1	73	72	10	63	1	72	319016.8920	0.0027	0.030	0.0018	0.25 Melli+22
889:	73	11	63	1	73	72	10	62	1	72	322974.1590	-0.0217	0.030	0.0306	0.25 Melli+22
890:	73	10	63	1	73	72	11	62	1	72	322974.1590	0.0830	0.030	0.0306	0.25 Melli+22

891:	73	10	63	1	73	72	10	62	1	72	322974.1590	0.0168	0.030	0.0306	0.25	Melli+22
892:	73	11	63	1	73	72	11	62	1	72	322974.1590	0.0444	0.030	0.0306	0.25	Melli+22
893:	85	0	85	1	85	84	1	84	1	84	330219.6410	0.0070	0.030	0.0070	0.25	Melli+22
894:	85	1	85	1	85	84	0	84	1	84	330219.6410	0.0070	0.030	0.0070	0.25	Melli+22
895:	85	0	85	1	85	84	0	84	1	84	330219.6410	0.0070	0.030	0.0070	0.25	Melli+22
896:	85	1	85	1	85	84	1	84	1	84	330219.6410	0.0070	0.030	0.0070	0.25	Melli+22
897:	82	3	79	1	82	81	3	78	1	81	330236.3340	0.0433	0.030	0.0098	0.25	Melli+22
898:	82	4	79	1	82	81	4	78	1	81	330236.3340	0.0433	0.030	0.0098	0.25	Melli+22
899:	81	4	77	1	81	80	4	76	1	80	330236.3340	-0.0237	0.030	0.0098	0.25	Melli+22
900:	81	5	77	1	81	80	5	76	1	80	330236.3340	-0.0237	0.030	0.0098	0.25	Melli+22
901:	80	6	75	1	80	79	5	74	1	79	330239.3200	-0.0138	0.030	-0.0138	0.25	Melli+22
902:	80	5	75	1	80	79	6	74	1	79	330239.3200	-0.0138	0.030	-0.0138	0.25	Melli+22
903:	80	6	75	1	80	79	6	74	1	79	330239.3200	-0.0138	0.030	-0.0138	0.25	Melli+22
904:	80	5	75	1	80	79	5	74	1	79	330239.3200	-0.0138	0.030	-0.0138	0.25	Melli+22
905:	84	2	83	1	84	83	1	82	1	83	330230.6200	0.0021	0.030	0.0021	0.25	Melli+22
906:	84	1	83	1	84	83	2	82	1	83	330230.6200	0.0021	0.030	0.0021	0.25	Melli+22
907:	84	2	83	1	84	83	2	82	1	83	330230.6200	0.0021	0.030	0.0021	0.25	Melli+22
908:	84	1	83	1	84	83	1	82	1	83	330230.6200	0.0021	0.030	0.0021	0.25	Melli+22
909:	83	3	81	1	83	82	2	80	1	82	330235.3370	-0.0310	0.030	-0.0310	0.25	Melli+22
910:	83	2	81	1	83	82	3	80	1	82	330235.3370	-0.0310	0.030	-0.0310	0.25	Melli+22
911:	83	2	81	1	83	82	2	80	1	82	330235.3370	-0.0310	0.030	-0.0310	0.25	Melli+22
912:	83	3	81	1	83	82	3	80	1	82	330235.3370	-0.0310	0.030	-0.0310	0.25	Melli+22
913:	82	3	79	1	82	81	4	78	1	81	330236.3340	0.0433	0.030	0.0098	0.25	Melli+22
914:	82	4	79	1	82	81	3	78	1	81	330236.3340	0.0433	0.030	0.0098	0.25	Melli+22
915:	81	5	77	1	81	80	4	76	1	80	330236.3340	-0.0237	0.030	0.0098	0.25	Melli+22
916:	81	4	77	1	81	80	5	76	1	80	330236.3340	-0.0237	0.030	0.0098	0.25	Melli+22
917:	79	7	73	1	79	78	6	72	1	78	330250.1330	0.0234	0.030	0.0235	0.25	Melli+22
918:	79	6	73	1	79	78	7	72	1	78	330250.1330	0.0234	0.030	0.0235	0.25	Melli+22
919:	79	6	73	1	79	78	6	72	1	78	330250.1330	0.0234	0.030	0.0235	0.25	Melli+22
920:	79	7	73	1	79	78	7	72	1	78	330250.1330	0.0234	0.030	0.0235	0.25	Melli+22
921:	78	8	71	1	78	77	7	70	1	77	330275.1850	-0.0272	0.030	-0.0272	0.25	Melli+22
922:	78	7	71	1	78	77	8	70	1	77	330275.1850	-0.0272	0.030	-0.0272	0.25	Melli+22
923:	78	7	71	1	78	77	7	70	1	77	330275.1850	-0.0272	0.030	-0.0272	0.25	Melli+22
924:	78	8	71	1	78	77	8	70	1	77	330275.1850	-0.0272	0.030	-0.0272	0.25	Melli+22
925:	77	9	69	1	77	76	8	68	1	76	330323.6000	-0.0154	0.030	-0.0155	0.25	Melli+22
926:	77	8	69	1	77	76	9	68	1	76	330323.6000	-0.0154	0.030	-0.0155	0.25	Melli+22
927:	77	8	69	1	77	76	8	68	1	76	330323.6000	-0.0154	0.030	-0.0155	0.25	Melli+22
928:	77	9	69	1	77	76	9	68	1	76	330323.6000	-0.0154	0.030	-0.0155	0.25	Melli+22
929:	76	10	67	1	76	75	9	66	1	75	330408.0930	0.0083	0.030	0.0090	0.25	Melli+22
930:	76	9	67	1	76	75	10	66	1	75	330408.0930	0.0096	0.030	0.0090	0.25	Melli+22
931:	76	9	67	1	76	75	9	66	1	75	330408.0930	0.0088	0.030	0.0090	0.25	Melli+22
932:	76	10	67	1	76	75	10	66	1	75	330408.0930	0.0091	0.030	0.0090	0.25	Melli+22
933:	75	11	65	1	75	74	10	64	1	74	330547.5840	0.0197	0.030	0.0376	0.25	Melli+22
934:	75	10	65	1	75	74	11	64	1	74	330547.5840	0.0553	0.030	0.0376	0.25	Melli+22
935:	75	10	65	1	75	74	10	64	1	74	330547.5840	0.0328	0.030	0.0376	0.25	Melli+22
936:	75	11	65	1	75	74	11	64	1	74	330547.5840	0.0422	0.030	0.0376	0.25	Melli+22
937:	88	7	82	1	88	87	6	81	1	87	364456.9950	0.0016	0.030	0.0017	0.25	Melli+22
938:	88	6	82	1	88	87	7	81	1	87	364456.9950	0.0016	0.030	0.0017	0.25	Melli+22
939:	88	7	82	1	88	87	7	81	1	87	364456.9950	0.0016	0.030	0.0017	0.25	Melli+22
940:	88	6	82	1	88	87	6	81	1	87	364456.9950	0.0016	0.030	0.0017	0.25	Melli+22
941:	89	6	84	1	89	88	5	83	1	88	364460.2150	-0.0040	0.030	-0.0040	0.25	Melli+22
942:	89	5	84	1	89	88	6	83	1	88	364460.2150	-0.0040	0.030	-0.0040	0.25	Melli+22
943:	89	6	84	1	89	88	6	83	1	88	364460.2150	-0.0040	0.030	-0.0040	0.25	Melli+22
944:	89	5	84	1	89	88	5	83	1	88	364460.2150	-0.0040	0.030	-0.0040	0.25	Melli+22
945:	87	8	80	1	87	86	7	79	1	86	364461.7350	-0.0071	0.030	-0.0071	0.25	Melli+22
946:	87	7	80	1	87	86	8	79	1	86	364461.7350	-0.0071	0.030	-0.0071	0.25	Melli+22
947:	87	8	80	1	87	86	8	79	1	86	364461.7350	-0.0071	0.030	-0.0071	0.25	Melli+22
948:	87	7	80	1	87	86	7	79	1	86	364461.7350	-0.0071	0.030	-0.0071	0.25	Melli+22
949:	94	1	94	1	94	93	0	93	1	93	364463.1630	-0.0128	0.030	-0.0129	0.25	Melli+22
950:	94	0	94	1	94	93	1	93	1	93	364463.1630	-0.0128	0.030	-0.0129	0.25	Melli+22
951:	94	0	94	1	94	93	0	93	1	93	364463.1630	-0.0128	0.030	-0.0129	0.25	Melli+22
952:	94	1	94	1	94	93	1	93	1	93	364463.1630	-0.0128	0.030	-0.0129	0.25	Melli+22
953:	93	1	92	1	93	92	2	91	1	92	364473.2910	-0.2072	0.030	0.0073	0.12	Melli+22
954:	93	2	92	1	93	92	1	91	1	92	364473.2910	-0.2072	0.030	0.0073	0.12	Melli+22
955:	93	2	92	1	93	92	2	91	1	92	364473.2910	-0.2072	0.030	0.0073	0.12	Melli+22
956:	93	1	92	1	93	92	1	91	1	92	364473.2910	-0.2072	0.030	0.0073	0.12	Melli+22
957:	91	3	88	1	91	90	4	87	1	90	364473.2910	0.2218	0.030	0.0073	0.12	Melli+22
958:	91	4	88	1	91	90	3	87	1	90	364473.2910	0.2218	0.030	0.0073	0.12	Melli+22
959:	91	4	88	1	91	90	4	87	1	90	364473.2910	0.2218	0.030	0.0073	0.12	Melli+22

960:	91	3	88	1	91	90	3	87	1	90	364473.2910	0.2218	0.030	0.0073	0.12	Melli+22
961:	92	2	90	1	92	91	3	89	1	91	364476.0930	-0.0199	0.030	-0.0200	0.25	Melli+22
962:	92	3	90	1	92	91	2	89	1	91	364476.0930	-0.0199	0.030	-0.0200	0.25	Melli+22
963:	92	3	90	1	92	91	3	89	1	91	364476.0930	-0.0199	0.030	-0.0200	0.25	Melli+22
964:	92	2	90	1	92	91	2	89	1	91	364476.0930	-0.0199	0.030	-0.0200	0.25	Melli+22
965:	86	9	78	1	86	85	8	77	1	85	364480.4970	0.0044	0.030	0.0044	0.25	Melli+22
966:	86	8	78	1	86	85	9	77	1	85	364480.4970	0.0044	0.030	0.0044	0.25	Melli+22
967:	86	8	78	1	86	85	8	77	1	85	364480.4970	0.0044	0.030	0.0044	0.25	Melli+22
968:	86	9	78	1	86	85	9	77	1	85	364480.4970	0.0044	0.030	0.0044	0.25	Melli+22
969:	85	10	76	1	85	84	9	75	1	84	364521.2840	-0.0271	0.030	-0.0272	0.25	Melli+22
970:	85	9	76	1	85	84	10	75	1	84	364521.2840	-0.0271	0.030	-0.0272	0.25	Melli+22
971:	85	9	76	1	85	84	9	75	1	84	364521.2840	-0.0271	0.030	-0.0272	0.25	Melli+22
972:	85	10	76	1	85	84	10	75	1	84	364521.2840	-0.0271	0.030	-0.0272	0.25	Melli+22
973:	84	11	74	1	84	83	10	73	1	83	364595.3500	0.0191	0.030	0.0192	0.25	Melli+22
974:	84	10	74	1	84	83	11	73	1	83	364595.3500	0.0193	0.030	0.0192	0.25	Melli+22
975:	84	10	74	1	84	83	10	73	1	83	364595.3500	0.0192	0.030	0.0192	0.25	Melli+22
976:	84	11	74	1	84	83	11	73	1	83	364595.3500	0.0192	0.030	0.0192	0.25	Melli+22
977:	89	6	83	1	89	88	7	82	1	88	368248.9990	-0.0282	0.030	-0.0283	0.25	Melli+22
978:	89	7	83	1	89	88	6	82	1	88	368248.9990	-0.0282	0.030	-0.0283	0.25	Melli+22
979:	89	7	83	1	89	88	7	82	1	88	368248.9990	-0.0282	0.030	-0.0283	0.25	Melli+22
980:	89	6	83	1	89	88	6	82	1	88	368248.9990	-0.0282	0.030	-0.0283	0.25	Melli+22
981:	90	6	85	1	90	89	5	84	1	89	368253.6210	0.0001	0.030	0.0001	0.25	Melli+22
982:	90	5	85	1	90	89	6	84	1	89	368253.6210	0.0001	0.030	0.0001	0.25	Melli+22
983:	90	6	85	1	90	89	6	84	1	89	368253.6210	0.0001	0.030	0.0001	0.25	Melli+22
984:	90	5	85	1	90	89	5	84	1	89	368253.6210	0.0001	0.030	0.0001	0.25	Melli+22
985:	95	1	95	1	95	94	0	94	1	94	368258.9190	0.0160	0.030	0.0160	0.25	Melli+22
986:	95	0	95	1	95	94	1	94	1	94	368258.9190	0.0160	0.030	0.0160	0.25	Melli+22
987:	95	1	95	1	95	94	1	94	1	94	368258.9190	0.0160	0.030	0.0160	0.25	Melli+22
988:	95	0	95	1	95	94	0	94	1	94	368258.9190	0.0160	0.030	0.0160	0.25	Melli+22
989:	88	8	81	1	88	87	7	80	1	87	368251.8280	-0.0182	0.030	-0.0182	0.25	Melli+22
990:	88	7	81	1	88	87	8	80	1	87	368251.8280	-0.0182	0.030	-0.0182	0.25	Melli+22
991:	88	7	81	1	88	87	7	80	1	87	368251.8280	-0.0182	0.030	-0.0182	0.25	Melli+22
992:	88	8	81	1	88	87	8	80	1	87	368251.8280	-0.0182	0.030	-0.0182	0.25	Melli+22
993:	91	4	87	1	91	90	5	86	1	90	368261.1500	-0.0029	0.030	-0.0029	0.25	Melli+22
994:	91	5	87	1	91	90	4	86	1	90	368261.1500	-0.0029	0.030	-0.0029	0.25	Melli+22
995:	91	5	87	1	91	90	5	86	1	90	368261.1500	-0.0029	0.030	-0.0029	0.25	Melli+22
996:	91	4	87	1	91	90	4	86	1	90	368261.1500	-0.0029	0.030	-0.0029	0.25	Melli+22
997:	94	1	93	1	94	93	2	92	1	93	368269.2210	0.0638	0.030	0.0638	0.25	Melli+22
998:	94	2	93	1	94	93	1	92	1	93	368269.2210	0.0638	0.030	0.0638	0.25	Melli+22
999:	94	1	93	1	94	93	1	92	1	93	368269.2210	0.0638	0.030	0.0638	0.25	Melli+22
1000:	94	2	93	1	94	93	2	92	1	93	368269.2210	0.0638	0.030	0.0638	0.25	Melli+22
1001:	93	2	91	1	93	92	3	90	1	92	368271.5490	0.0045	0.030	0.0046	0.25	Melli+22
1002:	93	3	91	1	93	92	2	90	1	92	368271.5490	0.0045	0.030	0.0046	0.25	Melli+22
1003:	93	2	91	1	93	92	2	90	1	92	368271.5490	0.0045	0.030	0.0046	0.25	Melli+22
1004:	93	3	91	1	93	92	3	90	1	92	368271.5490	0.0045	0.030	0.0046	0.25	Melli+22
1005:	86	10	77	1	86	85	9	76	1	85	368304.8210	-0.0031	0.030	-0.0032	0.25	Melli+22
1006:	86	9	77	1	86	85	10	76	1	85	368304.8210	-0.0031	0.030	-0.0032	0.25	Melli+22
1007:	86	9	77	1	86	85	9	76	1	85	368304.8210	-0.0031	0.030	-0.0032	0.25	Melli+22
1008:	86	10	77	1	86	85	10	76	1	85	368304.8210	-0.0031	0.030	-0.0032	0.25	Melli+22
1009:	85	11	75	1	85	84	10	74	1	84	368373.2850	0.0147	0.030	0.0148	0.25	Melli+22
1010:	85	10	75	1	85	84	11	74	1	84	368373.2850	0.0149	0.030	0.0148	0.25	Melli+22
1011:	85	10	75	1	85	84	10	74	1	84	368373.2850	0.0148	0.030	0.0148	0.25	Melli+22
1012:	85	11	75	1	85	84	11	74	1	84	368373.2850	0.0148	0.030	0.0148	0.25	Melli+22
1013:	90	7	84	1	90	89	6	83	1	89	372039.1620	-0.0711	0.030	-0.0712	0.25	Melli+22
1014:	90	6	84	1	90	89	7	83	1	89	372039.1620	-0.0711	0.030	-0.0712	0.25	Melli+22
1015:	90	7	84	1	90	89	7	83	1	89	372039.1620	-0.0711	0.030	-0.0712	0.25	Melli+22
1016:	90	6	84	1	90	89	6	83	1	89	372039.1620	-0.0711	0.030	-0.0712	0.25	Melli+22
1017:	89	8	82	1	89	88	7	81	1	88	372040.2360	0.0620	0.030	0.0620	0.25	Melli+22
1018:	89	7	82	1	89	88	8	81	1	88	372040.2360	0.0620	0.030	0.0620	0.25	Melli+22
1019:	89	7	82	1	89	88	7	81	1	88	372040.2360	0.0620	0.030	0.0620	0.25	Melli+22
1020:	89	8	82	1	89	88	8	81	1	88	372040.2360	0.0620	0.030	0.0620	0.25	Melli+22
1021:	91	5	86	1	91	90	6	85	1	90	372045.1780	0.0129	0.030	0.0130	0.25	Melli+22
1022:	91	6	86	1	91	90	5	85	1	90	372045.1780	0.0129	0.030	0.0130	0.25	Melli+22
1023:	91	6	86	1	91	90	6	85	1	90	372045.1780	0.0129	0.030	0.0130	0.25	Melli+22
1024:	91	5	86	1	91	90	5	85	1	90	372045.1780	0.0129	0.030	0.0130	0.25	Melli+22
1025:	96	1	96	1	96	95	0	95	1	95	372052.7120	-0.0296	0.030	-0.0296	0.25	Melli+22
1026:	96	0	96	1	96	95	1	95	1	95	372052.7120	-0.0296	0.030	-0.0296	0.25	Melli+22
1027:	96	0	96	1	96	95	0	95	1	95	372052.7120	-0.0296	0.030	-0.0296	0.25	Melli+22
1028:	96	1	96	1	96	95	1	95	1	95	372052.7120	-0.0296	0.030	-0.0296	0.25	Melli+22

1029:	93	3	90	1 93	92	4	89	1 92	372061.2230	0.0072	0.030	0.0072	0.25	Melli+22
1030:	93	4	90	1 93	92	3	89	1 92	372061.2230	0.0072	0.030	0.0072	0.25	Melli+22
1031:	93	3	90	1 93	92	3	89	1 92	372061.2230	0.0072	0.030	0.0072	0.25	Melli+22
1032:	93	4	90	1 93	92	4	89	1 92	372061.2230	0.0072	0.030	0.0072	0.25	Melli+22
1033:	95	2	94	1 95	94	1	93	1 94	372062.9570	0.0283	0.030	0.0283	0.25	Melli+22
1034:	95	1	94	1 95	94	2	93	1 94	372062.9570	0.0283	0.030	0.0283	0.25	Melli+22
1035:	95	2	94	1 95	94	2	93	1 94	372062.9570	0.0283	0.030	0.0283	0.25	Melli+22
1036:	95	1	94	1 95	94	1	93	1 94	372062.9570	0.0283	0.030	0.0283	0.25	Melli+22
1037:	94	2	92	1 94	93	3	91	1 93	372065.1040	0.0138	0.030	0.0139	0.25	Melli+22
1038:	94	3	92	1 94	93	2	91	1 93	372065.1040	0.0138	0.030	0.0139	0.25	Melli+22
1039:	94	2	92	1 94	93	2	91	1 93	372065.1040	0.0138	0.030	0.0139	0.25	Melli+22
1040:	94	3	92	1 94	93	3	91	1 93	372065.1040	0.0138	0.030	0.0139	0.25	Melli+22
1041:	87	10	78	1 87	86	9	77	1 86	372086.8210	0.0248	0.030	0.0249	0.25	Melli+22
1042:	87	9	78	1 87	86	10	77	1 86	372086.8210	0.0248	0.030	0.0249	0.25	Melli+22
1043:	87	9	78	1 87	86	9	77	1 86	372086.8210	0.0248	0.030	0.0249	0.25	Melli+22
1044:	87	10	78	1 87	86	10	77	1 86	372086.8210	0.0248	0.030	0.0249	0.25	Melli+22
1045:	84	13	72	1 84	83	12	71	1 83	372429.1490	-0.0492	0.030	-0.0184	0.25	Melli+22
1046:	84	12	72	1 84	83	13	71	1 83	372429.1490	0.0124	0.030	-0.0184	0.25	Melli+22
1047:	84	12	72	1 84	83	12	71	1 83	372429.1490	-0.0261	0.030	-0.0184	0.25	Melli+22
1048:	84	13	72	1 84	83	13	71	1 83	372429.1490	-0.0106	0.030	-0.0184	0.25	Melli+22
1049:	90	7	83	1 90	89	8	82	1 89	375826.6250	-0.0758	0.030	-0.0759	0.25	Melli+22
1050:	90	8	83	1 90	89	7	82	1 89	375826.6250	-0.0758	0.030	-0.0759	0.25	Melli+22
1051:	90	7	83	1 90	89	7	82	1 89	375826.6250	-0.0758	0.030	-0.0759	0.25	Melli+22
1052:	90	8	83	1 90	89	8	82	1 89	375826.6250	-0.0758	0.030	-0.0759	0.25	Melli+22
1053:	91	7	85	1 91	90	6	84	1 90	375827.6270	0.0373	0.030	0.0373	0.25	Melli+22
1054:	91	6	85	1 91	90	7	84	1 90	375827.6270	0.0373	0.030	0.0373	0.25	Melli+22
1055:	91	7	85	1 91	90	7	84	1 90	375827.6270	0.0373	0.030	0.0373	0.25	Melli+22
1056:	91	6	85	1 91	90	6	84	1 90	375827.6270	0.0373	0.030	0.0373	0.25	Melli+22
1057:	92	6	87	1 92	91	5	86	1 91	375834.8560	0.0240	0.030	0.0241	0.25	Melli+22
1058:	92	5	87	1 92	91	6	86	1 91	375834.8560	0.0240	0.030	0.0241	0.25	Melli+22
1059:	92	5	87	1 92	91	5	86	1 91	375834.8560	0.0240	0.030	0.0241	0.25	Melli+22
1060:	92	6	87	1 92	91	6	86	1 91	375834.8560	0.0240	0.030	0.0241	0.25	Melli+22
1061:	89	9	81	1 89	88	8	80	1 88	375837.5740	0.0319	0.030	0.0320	0.25	Melli+22
1062:	89	8	81	1 89	88	9	80	1 88	375837.5740	0.0319	0.030	0.0320	0.25	Melli+22
1063:	89	8	81	1 89	88	8	80	1 88	375837.5740	0.0319	0.030	0.0320	0.25	Melli+22
1064:	89	9	81	1 89	88	9	80	1 88	375837.5740	0.0319	0.030	0.0320	0.25	Melli+22
1065:	94	3	91	1 94	93	4	90	1 93	375852.4360	-0.0118	0.030	-0.0119	0.25	Melli+22
1066:	94	4	91	1 94	93	3	90	1 93	375852.4360	-0.0118	0.030	-0.0119	0.25	Melli+22
1067:	94	4	91	1 94	93	4	90	1 93	375852.4360	-0.0118	0.030	-0.0119	0.25	Melli+22
1068:	94	3	91	1 94	93	3	90	1 93	375852.4360	-0.0118	0.030	-0.0119	0.25	Melli+22
1069:	95	3	93	1 95	94	2	92	1 94	375856.7970	0.0649	0.030	0.0649	0.25	Melli+22
1070:	95	2	93	1 95	94	3	92	1 94	375856.7970	0.0649	0.030	0.0649	0.25	Melli+22
1071:	95	2	93	1 95	94	2	92	1 94	375856.7970	0.0649	0.030	0.0649	0.25	Melli+22
1072:	95	3	93	1 95	94	3	92	1 94	375856.7970	0.0649	0.030	0.0649	0.25	Melli+22
1073:	88	10	79	1 88	87	9	78	1 87	375867.1700	-0.0169	0.030	-0.0170	0.25	Melli+22
1074:	88	9	79	1 88	87	10	78	1 87	375867.1700	-0.0169	0.030	-0.0170	0.25	Melli+22
1075:	88	9	79	1 88	87	9	78	1 87	375867.1700	-0.0169	0.030	-0.0170	0.25	Melli+22
1076:	88	10	79	1 88	87	10	78	1 87	375867.1700	-0.0169	0.030	-0.0170	0.25	Melli+22
1077:	87	11	77	1 87	86	10	76	1 86	375925.2490	0.0372	0.030	0.0373	0.25	Melli+22
1078:	87	10	77	1 87	86	11	76	1 86	375925.2490	0.0373	0.030	0.0373	0.25	Melli+22
1079:	87	10	77	1 87	86	10	76	1 86	375925.2490	0.0372	0.030	0.0373	0.25	Melli+22
1080:	87	11	77	1 87	86	11	76	1 86	375925.2490	0.0372	0.030	0.0373	0.25	Melli+22
1081:	86	12	75	1 86	85	11	74	1 85	376025.0060	-0.0078	0.030	-0.0071	0.25	Melli+22
1082:	86	11	75	1 86	85	12	74	1 85	376025.0060	-0.0063	0.030	-0.0071	0.25	Melli+22
1083:	86	11	75	1 86	85	11	74	1 85	376025.0060	-0.0073	0.030	-0.0071	0.25	Melli+22
1084:	86	12	75	1 86	85	12	74	1 85	376025.0060	-0.0069	0.030	-0.0071	0.25	Melli+22
1085:	91	8	84	1 91	90	7	83	1 90	379611.4130	0.0100	0.030	0.0100	0.25	Melli+22
1086:	91	7	84	1 91	90	8	83	1 90	379611.4130	0.0100	0.030	0.0100	0.25	Melli+22
1087:	91	8	84	1 91	90	8	83	1 90	379611.4130	0.0100	0.030	0.0100	0.25	Melli+22
1088:	91	7	84	1 91	90	7	83	1 90	379611.4130	0.0100	0.030	0.0100	0.25	Melli+22
1089:	92	7	86	1 92	91	6	85	1 91	379614.0720	-0.0037	0.030	-0.0037	0.25	Melli+22
1090:	92	6	86	1 92	91	7	85	1 91	379614.0720	-0.0037	0.030	-0.0037	0.25	Melli+22
1091:	92	7	86	1 92	91	7	85	1 91	379614.0720	-0.0037	0.030	-0.0037	0.25	Melli+22
1092:	92	6	86	1 92	91	6	85	1 91	379614.0720	-0.0037	0.030	-0.0037	0.25	Melli+22
1093:	90	9	82	1 90	89	8	81	1 89	379619.7720	0.0031	0.030	0.0031	0.25	Melli+22
1094:	90	8	82	1 90	89	9	81	1 89	379619.7720	0.0031	0.030	0.0031	0.25	Melli+22
1095:	90	8	82	1 90	89	8	81	1 89	379619.7720	0.0031	0.030	0.0031	0.25	Melli+22
1096:	90	9	82	1 90	89	9	81	1 89	379619.7720	0.0031	0.030	0.0031	0.25	Melli+22
1097:	93	6	88	1 93	92	5	87	1 92	379622.5760	-0.0261	0.030	-0.0261	0.25	Melli+22

1098:	93	5	88	1	93	92	6	87	1	92	379622.5760	-0.0261	0.030	-0.0261	0.25	Melli+22
1099:	93	5	88	1	93	92	5	87	1	92	379622.5760	-0.0261	0.030	-0.0261	0.25	Melli+22
1100:	93	6	88	1	93	92	6	87	1	92	379622.5760	-0.0261	0.030	-0.0261	0.25	Melli+22
1101:	94	5	90	1	94	93	4	89	1	93	379632.9110	-0.0072	0.030	-0.0072	0.25	Melli+22
1102:	94	4	90	1	94	93	5	89	1	93	379632.9110	-0.0072	0.030	-0.0072	0.25	Melli+22
1103:	94	4	90	1	94	93	4	89	1	93	379632.9110	-0.0072	0.030	-0.0072	0.25	Melli+22
1104:	94	5	90	1	94	93	5	89	1	93	379632.9110	-0.0072	0.030	-0.0072	0.25	Melli+22
1105:	98	1	98	1	98	97	0	97	1	97	379634.6880	0.0059	0.030	0.0059	0.25	Melli+22
1106:	98	0	98	1	98	97	1	97	1	97	379634.6880	0.0059	0.030	0.0059	0.25	Melli+22
1107:	98	0	98	1	98	97	0	97	1	97	379634.6880	0.0059	0.030	0.0059	0.25	Melli+22
1108:	98	1	98	1	98	97	1	97	1	97	379634.6880	0.0059	0.030	0.0059	0.25	Melli+22
1109:	95	4	92	1	95	94	3	91	1	94	379641.7210	-0.0406	0.030	-0.0406	0.25	Melli+22
1110:	95	3	92	1	95	94	4	91	1	94	379641.7210	-0.0406	0.030	-0.0406	0.25	Melli+22
1111:	95	4	92	1	95	94	4	91	1	94	379641.7210	-0.0406	0.030	-0.0406	0.25	Melli+22
1112:	95	3	92	1	95	94	3	91	1	94	379641.7210	-0.0406	0.030	-0.0406	0.25	Melli+22
1113:	97	1	96	1	97	96	2	95	1	96	379644.7110	-0.0269	0.030	-0.0269	0.25	Melli+22
1114:	97	2	96	1	97	96	1	95	1	96	379644.7110	-0.0269	0.030	-0.0269	0.25	Melli+22
1115:	97	1	96	1	97	96	1	95	1	96	379644.7110	-0.0269	0.030	-0.0269	0.25	Melli+22
1116:	97	2	96	1	97	96	2	95	1	96	379644.7110	-0.0269	0.030	-0.0269	0.25	Melli+22
1117:	88	11	78	1	88	87	10	77	1	87	379699.0990	-0.0019	0.030	-0.0019	0.25	Melli+22
1118:	88	10	78	1	88	87	11	77	1	87	379699.0990	-0.0018	0.030	-0.0019	0.25	Melli+22
1119:	88	10	78	1	88	87	10	77	1	87	379699.0990	-0.0019	0.030	-0.0019	0.25	Melli+22
1120:	88	11	78	1	88	87	11	77	1	87	379699.0990	-0.0019	0.030	-0.0019	0.25	Melli+22
1121:	87	12	76	1	87	86	11	75	1	86	379791.8850	0.0058	0.030	0.0063	0.25	Melli+22
1122:	87	11	76	1	87	86	12	75	1	86	379791.8850	0.0066	0.030	0.0063	0.25	Melli+22
1123:	87	11	76	1	87	86	11	75	1	86	379791.8850	0.0061	0.030	0.0063	0.25	Melli+22
1124:	87	12	76	1	87	86	12	75	1	86	379791.8850	0.0063	0.030	0.0063	0.25	Melli+22
1125:	86	13	74	1	86	85	12	73	1	85	379942.6560	0.0457	0.030	0.0568	0.25	Melli+22
1126:	86	12	74	1	86	85	13	73	1	85	379942.6560	0.0677	0.030	0.0568	0.25	Melli+22
1127:	86	12	74	1	86	85	12	73	1	85	379942.6560	0.0539	0.030	0.0568	0.25	Melli+22
1128:	86	13	74	1	86	85	13	73	1	85	379942.6560	0.0595	0.030	0.0568	0.25	Melli+22
1129:	92	7	85	1	92	91	8	84	1	91	383394.2640	0.0073	0.030	0.0073	0.25	Melli+22
1130:	92	8	85	1	92	91	7	84	1	91	383394.2640	0.0073	0.030	0.0073	0.25	Melli+22
1131:	92	7	85	1	92	91	7	84	1	91	383394.2640	0.0073	0.030	0.0073	0.25	Melli+22
1132:	92	8	85	1	92	91	8	84	1	91	383394.2640	0.0073	0.030	0.0073	0.25	Melli+22
1133:	93	7	87	1	93	92	6	86	1	92	383398.6520	-0.0184	0.030	-0.0185	0.25	Melli+22
1134:	93	6	87	1	93	92	7	86	1	92	383398.6520	-0.0184	0.030	-0.0185	0.25	Melli+22
1135:	93	6	87	1	93	92	6	86	1	92	383398.6520	-0.0184	0.030	-0.0185	0.25	Melli+22
1136:	93	7	87	1	93	92	7	86	1	92	383398.6520	-0.0184	0.030	-0.0185	0.25	Melli+22
1137:	91	9	83	1	91	90	8	82	1	90	383400.2010	-0.0187	0.030	-0.0187	0.25	Melli+22
1138:	91	8	83	1	91	90	9	82	1	90	383400.2010	-0.0187	0.030	-0.0187	0.25	Melli+22
1139:	91	8	83	1	91	90	8	82	1	90	383400.2010	-0.0187	0.030	-0.0187	0.25	Melli+22
1140:	91	9	83	1	91	90	9	82	1	90	383400.2010	-0.0187	0.030	-0.0187	0.25	Melli+22
1141:	94	5	89	1	94	93	6	88	1	93	383408.4850	0.0286	0.030	0.0287	0.25	Melli+22
1142:	94	6	89	1	94	93	5	88	1	93	383408.4850	0.0286	0.030	0.0287	0.25	Melli+22
1143:	94	5	89	1	94	93	5	88	1	93	383408.4850	0.0286	0.030	0.0287	0.25	Melli+22
1144:	94	6	89	1	94	93	6	88	1	93	383408.4850	0.0286	0.030	0.0287	0.25	Melli+22
1145:	95	4	91	1	95	94	5	90	1	94	383419.6550	-0.0172	0.030	-0.0173	0.33	Melli+22
1146:	95	5	91	1	95	94	4	90	1	94	383419.6550	-0.0172	0.030	-0.0173	0.33	Melli+22
1147:	95	5	91	1	95	94	5	90	1	94	383419.6550	-0.0172	0.030	-0.0173	0.33	Melli+22
1148:	96	4	93	1	96	95	3	92	1	95	383429.0880	-0.0510	0.030	-0.0511	0.25	Melli+22
1149:	96	3	93	1	96	95	4	92	1	95	383429.0880	-0.0510	0.030	-0.0511	0.25	Melli+22
1150:	96	4	93	1	96	95	4	92	1	95	383429.0880	-0.0510	0.030	-0.0511	0.25	Melli+22
1151:	96	3	93	1	96	95	3	92	1	95	383429.0880	-0.0510	0.030	-0.0511	0.25	Melli+22
1152:	98	2	97	1	98	97	1	96	1	97	383432.7260	-0.0140	0.030	-0.0141	0.25	Melli+22
1153:	98	1	97	1	98	97	2	96	1	97	383432.7260	-0.0140	0.030	-0.0141	0.25	Melli+22
1154:	98	1	97	1	98	97	1	96	1	97	383432.7260	-0.0140	0.030	-0.0141	0.25	Melli+22
1155:	98	2	97	1	98	97	2	96	1	97	383432.7260	-0.0140	0.030	-0.0141	0.25	Melli+22
1156:	97	3	95	1	97	96	2	94	1	96	383434.2250	-0.0081	0.030	-0.0081	0.25	Melli+22
1157:	97	2	95	1	97	96	3	94	1	96	383434.2250	-0.0081	0.030	-0.0081	0.25	Melli+22
1158:	97	2	95	1	97	96	2	94	1	96	383434.2250	-0.0081	0.030	-0.0081	0.25	Melli+22
1159:	97	3	95	1	97	96	3	94	1	96	383434.2250	-0.0081	0.030	-0.0081	0.25	Melli+22
1160:	88	12	77	1	88	87	11	76	1	87	383557.6160	-0.0025	0.030	-0.0023	0.25	Melli+22
1161:	88	11	77	1	88	87	12	76	1	87	383557.6160	-0.0020	0.030	-0.0023	0.25	Melli+22
1162:	88	11	77	1	88	87	11	76	1	87	383557.6160	-0.0023	0.030	-0.0023	0.25	Melli+22
1163:	88	12	77	1	88	87	12	76	1	87	383557.6160	-0.0022	0.030	-0.0023	0.25	Melli+22
1164:	87	13	75	1	87	86	12	74	1	86	383698.5450	-0.0065	0.030	0.0000	0.25	Melli+22
1165:	87	12	75	1	87	86	13	74	1	86	383698.5450	0.0065	0.030	0.0000	0.25	Melli+22
1166:	87	12	75	1	87	86	12	74	1	86	383698.5450	-0.0016	0.030	0.0000	0.25	Melli+22

1167:	87	13	75	1	87	86	13	74	1	86	383698.5450	0.0016	0.030	0.0000	0.25	Melli+22
1168:	93	7	86	1	93	92	8	85	1	92	387175.2610	0.0219	0.030	0.0220	0.25	Melli+22
1169:	93	8	86	1	93	92	7	85	1	92	387175.2610	0.0219	0.030	0.0220	0.25	Melli+22
1170:	93	8	86	1	93	92	8	85	1	92	387175.2610	0.0219	0.030	0.0220	0.25	Melli+22
1171:	93	7	86	1	93	92	7	85	1	92	387175.2610	0.0219	0.030	0.0220	0.25	Melli+22
1172:	92	9	84	1	92	91	8	83	1	91	387178.8980	0.0309	0.030	0.0310	0.25	Melli+22
1173:	92	8	84	1	92	91	9	83	1	91	387178.8980	0.0309	0.030	0.0310	0.25	Melli+22
1174:	92	8	84	1	92	91	8	83	1	91	387178.8980	0.0309	0.030	0.0310	0.25	Melli+22
1175:	92	9	84	1	92	91	9	83	1	91	387178.8980	0.0309	0.030	0.0310	0.25	Melli+22
1176:	94	7	88	1	94	93	6	87	1	93	387181.3760	0.0225	0.030	0.0226	0.25	Melli+22
1177:	94	6	88	1	94	93	7	87	1	93	387181.3760	0.0225	0.030	0.0226	0.25	Melli+22
1178:	94	6	88	1	94	93	6	87	1	93	387181.3760	0.0225	0.030	0.0226	0.25	Melli+22
1179:	94	7	88	1	94	93	7	87	1	93	387181.3760	0.0225	0.030	0.0226	0.25	Melli+22
1180:	95	5	90	1	95	94	6	89	1	94	387192.3490	-0.0264	0.030	-0.0265	0.25	Melli+22
1181:	95	6	90	1	95	94	5	89	1	94	387192.3490	-0.0264	0.030	-0.0265	0.25	Melli+22
1182:	95	5	90	1	95	94	5	89	1	94	387192.3490	-0.0264	0.030	-0.0265	0.25	Melli+22
1183:	95	6	90	1	95	94	6	89	1	94	387192.3490	-0.0264	0.030	-0.0265	0.25	Melli+22
1184:	91	10	82	1	91	90	9	81	1	90	387198.5100	0.0122	0.030	0.0123	0.25	Melli+22
1185:	91	9	82	1	91	90	10	81	1	90	387198.5100	0.0122	0.030	0.0123	0.25	Melli+22
1186:	91	9	82	1	91	90	9	81	1	90	387198.5100	0.0122	0.030	0.0123	0.25	Melli+22
1187:	91	10	82	1	91	90	10	81	1	90	387198.5100	0.0122	0.030	0.0123	0.25	Melli+22
1188:	97	4	94	1	97	96	3	93	1	96	387214.5840	0.0218	0.030	0.0218	0.25	Melli+22
1189:	97	3	94	1	97	96	4	93	1	96	387214.5840	0.0218	0.030	0.0218	0.25	Melli+22
1190:	97	4	94	1	97	96	4	93	1	96	387214.5840	0.0218	0.030	0.0218	0.25	Melli+22
1191:	97	3	94	1	97	96	3	93	1	96	387214.5840	0.0218	0.030	0.0218	0.25	Melli+22
1192:	99	2	98	1	99	98	1	97	1	98	387218.7840	0.0003	0.030	0.0004	0.25	Melli+22
1193:	99	1	98	1	99	98	2	97	1	98	387218.7840	0.0003	0.030	0.0004	0.25	Melli+22
1194:	99	2	98	1	99	98	2	97	1	98	387218.7840	0.0003	0.030	0.0004	0.25	Melli+22
1195:	99	1	98	1	99	98	1	97	1	98	387218.7840	0.0003	0.030	0.0004	0.25	Melli+22
1196:	98	2	96	1	98	97	3	95	1	97	387220.1140	0.0573	0.030	0.0573	0.25	Melli+22
1197:	98	3	96	1	98	97	2	95	1	97	387220.1140	0.0573	0.030	0.0573	0.25	Melli+22
1198:	98	3	96	1	98	97	3	95	1	97	387220.1140	0.0573	0.030	0.0573	0.25	Melli+22
1199:	98	2	96	1	98	97	2	95	1	97	387220.1140	0.0573	0.030	0.0573	0.25	Melli+22
1200:	90	11	80	1	90	89	10	79	1	89	387242.4650	0.0078	0.030	0.0078	0.25	Melli+22
1201:	90	10	80	1	90	89	11	79	1	89	387242.4650	0.0078	0.030	0.0078	0.25	Melli+22
1202:	90	10	80	1	90	89	10	79	1	89	387242.4650	0.0078	0.030	0.0078	0.25	Melli+22
1203:	90	11	80	1	90	89	11	79	1	89	387242.4650	0.0078	0.030	0.0078	0.25	Melli+22
1204:	89	12	78	1	89	88	11	77	1	88	387322.1580	-0.0000	0.030	0.0001	0.25	Melli+22
1205:	89	11	78	1	89	88	12	77	1	88	387322.1580	0.0002	0.030	0.0001	0.25	Melli+22
1206:	89	11	78	1	89	88	11	77	1	88	387322.1580	0.0000	0.030	0.0001	0.25	Melli+22
1207:	89	12	78	1	89	88	12	77	1	88	387322.1580	0.0001	0.030	0.0001	0.25	Melli+22
1208:	88	13	76	1	88	87	12	75	1	87	387453.8050	-0.0106	0.030	-0.0067	0.25	Melli+22
1209:	88	12	76	1	88	87	13	75	1	87	387453.8050	-0.0028	0.030	-0.0067	0.25	Melli+22
1210:	88	12	76	1	88	87	12	75	1	87	387453.8050	-0.0077	0.030	-0.0067	0.25	Melli+22
1211:	88	13	76	1	88	87	13	75	1	87	387453.8050	-0.0057	0.030	-0.0067	0.25	Melli+22
1212:	87	13	74	1	87	86	14	73	1	86	387661.4100	0.0046	0.030	-0.0824	0.25	Melli+22
1213:	87	14	74	1	87	86	14	73	1	86	387661.4100	-0.0610	0.030	-0.0824	0.25	Melli+22
1214:	87	14	74	1	87	86	13	73	1	86	387661.4100	-0.1693	0.030	-0.0824	0.25	Melli+22
1215:	87	13	74	1	87	86	13	73	1	86	387661.4100	-0.1036	0.030	-0.0824	0.25	Melli+22
1216:	94	7	87	1	94	93	8	86	1	93	390954.2930	-0.0343	0.030	-0.0344	0.25	Melli+22
1217:	94	8	87	1	94	93	7	86	1	93	390954.2930	-0.0343	0.030	-0.0344	0.25	Melli+22
1218:	94	8	87	1	94	93	8	86	1	93	390954.2930	-0.0343	0.030	-0.0344	0.25	Melli+22
1219:	94	7	87	1	94	93	7	86	1	93	390954.2930	-0.0343	0.030	-0.0344	0.25	Melli+22
1220:	93	8	85	1	93	92	9	84	1	92	390955.7100	0.0259	0.030	0.0259	0.25	Melli+22
1221:	93	9	85	1	93	92	8	84	1	92	390955.7100	0.0259	0.030	0.0259	0.25	Melli+22
1222:	93	9	85	1	93	92	9	84	1	92	390955.7100	0.0259	0.030	0.0259	0.25	Melli+22
1223:	93	8	85	1	93	92	8	84	1	92	390955.7100	0.0259	0.030	0.0259	0.25	Melli+22
1224:	95	6	89	1	95	94	7	88	1	94	390962.0970	-0.0072	0.030	-0.0073	0.25	Melli+22
1225:	95	7	89	1	95	94	6	88	1	94	390962.0970	-0.0072	0.030	-0.0073	0.25	Melli+22
1226:	95	6	89	1	95	94	6	88	1	94	390962.0970	-0.0072	0.030	-0.0073	0.25	Melli+22
1227:	95	7	89	1	95	94	7	88	1	94	390962.0970	-0.0072	0.030	-0.0073	0.25	Melli+22
1228:	92	10	83	1	92	91	9	82	1	91	390972.2530	0.0564	0.030	0.0565	0.25	Melli+22
1229:	92	9	83	1	92	91	10	82	1	91	390972.2530	0.0564	0.030	0.0565	0.25	Melli+22
1230:	92	9	83	1	92	91	9	82	1	91	390972.2530	0.0564	0.030	0.0565	0.25	Melli+22
1231:	92	10	83	1	92	91	10	82	1	91	390972.2530	0.0564	0.030	0.0565	0.25	Melli+22
1232:	96	5	91	1	96	95	6	90	1	95	390974.3530	0.0125	0.030	0.0125	0.25	Melli+22
1233:	96	6	91	1	96	95	5	90	1	95	390974.3530	0.0125	0.030	0.0125	0.25	Melli+22
1234:	96	6	91	1	96	95	6	90	1	95	390974.3530	0.0125	0.030	0.0125	0.25	Melli+22
1235:	96	5	91	1	96	95	5	90	1	95	390974.3530	0.0125	0.030	0.0125	0.25	Melli+22

1236:	97	4	93	1	97	96	5	92	1	96	390987.2650	-0.0536	0.030	-0.0536	0.25	Melli+22
1237:	97	5	93	1	97	96	4	92	1	96	390987.2650	-0.0536	0.030	-0.0536	0.25	Melli+22
1238:	97	4	93	1	97	96	4	92	1	96	390987.2650	-0.0536	0.030	-0.0536	0.25	Melli+22
1239:	97	5	93	1	97	96	5	92	1	96	390987.2650	-0.0536	0.030	-0.0536	0.25	Melli+22
1240:	98	4	95	1	98	97	3	94	1	97	390998.0110	-0.0021	0.030	-0.0021	0.25	Melli+22
1241:	98	3	95	1	98	97	4	94	1	97	390998.0110	-0.0021	0.030	-0.0021	0.25	Melli+22
1242:	98	4	95	1	98	97	4	94	1	97	390998.0110	-0.0021	0.030	-0.0021	0.25	Melli+22
1243:	98	3	95	1	98	97	3	94	1	97	390998.0110	-0.0021	0.030	-0.0021	0.25	Melli+22
1244:	100	2	99	1	100	99	1	98	1	99	391002.8110	-0.0398	0.030	-0.0399	0.25	Melli+22
1245:	100	1	99	1	100	99	2	98	1	99	391002.8110	-0.0398	0.030	-0.0399	0.25	Melli+22
1246:	100	2	99	1	100	99	2	98	1	99	391002.8110	-0.0398	0.030	-0.0399	0.25	Melli+22
1247:	100	1	99	1	100	99	1	98	1	99	391002.8110	-0.0398	0.030	-0.0399	0.25	Melli+22
1248:	99	2	97	1	99	98	3	96	1	98	391003.9490	0.0437	0.030	0.0438	0.25	Melli+22
1249:	99	3	97	1	99	98	2	96	1	98	391003.9490	0.0437	0.030	0.0438	0.25	Melli+22
1250:	99	2	97	1	99	98	2	96	1	98	391003.9490	0.0437	0.030	0.0438	0.25	Melli+22
1251:	99	3	97	1	99	98	3	96	1	98	391003.9490	0.0437	0.030	0.0438	0.25	Melli+22
1252:	91	11	81	1	91	90	10	80	1	90	391011.8450	0.0161	0.030	0.0162	0.25	Melli+22
1253:	91	10	81	1	91	90	11	80	1	90	391011.8450	0.0161	0.030	0.0162	0.25	Melli+22
1254:	91	10	81	1	91	90	10	80	1	90	391011.8450	0.0161	0.030	0.0162	0.25	Melli+22
1255:	91	11	81	1	91	90	11	80	1	90	391011.8450	0.0161	0.030	0.0162	0.25	Melli+22
1256:	94	9	86	1	94	93	8	85	1	93	394730.6540	0.0091	0.030	0.0092	0.25	Melli+22
1257:	94	8	86	1	94	93	9	85	1	93	394730.6540	0.0091	0.030	0.0092	0.25	Melli+22
1258:	94	9	86	1	94	93	9	85	1	93	394730.6540	0.0091	0.030	0.0092	0.25	Melli+22
1259:	94	8	86	1	94	93	8	85	1	93	394730.6540	0.0091	0.030	0.0092	0.25	Melli+22
1260:	95	7	88	1	95	94	8	87	1	94	394731.5280	0.0286	0.030	0.0287	0.25	Melli+22
1261:	95	8	88	1	95	94	7	87	1	94	394731.5280	0.0286	0.030	0.0287	0.25	Melli+22
1262:	95	7	88	1	95	94	7	87	1	94	394731.5280	0.0286	0.030	0.0287	0.25	Melli+22
1263:	95	8	88	1	95	94	8	87	1	94	394731.5280	0.0286	0.030	0.0287	0.25	Melli+22
1264:	96	7	90	1	96	95	6	89	1	95	394740.9140	0.0110	0.030	0.0111	0.25	Melli+22
1265:	96	6	90	1	96	95	7	89	1	95	394740.9140	0.0110	0.030	0.0111	0.25	Melli+22
1266:	96	7	90	1	96	95	7	89	1	95	394740.9140	0.0110	0.030	0.0111	0.25	Melli+22
1267:	96	6	90	1	96	95	6	89	1	95	394740.9140	0.0110	0.030	0.0111	0.25	Melli+22
1268:	93	10	84	1	93	92	9	83	1	92	394744.1580	0.0206	0.030	0.0206	0.25	Melli+22
1269:	93	9	84	1	93	92	10	83	1	92	394744.1580	0.0206	0.030	0.0206	0.25	Melli+22
1270:	93	9	84	1	93	92	9	83	1	92	394744.1580	0.0206	0.030	0.0206	0.25	Melli+22
1271:	93	10	84	1	93	92	10	83	1	92	394744.1580	0.0206	0.030	0.0206	0.25	Melli+22
1272:	97	6	92	1	97	96	5	91	1	96	394754.3370	0.0043	0.030	0.0044	0.25	Melli+22
1273:	97	5	92	1	97	96	6	91	1	96	394754.3370	0.0043	0.030	0.0044	0.25	Melli+22
1274:	97	5	92	1	97	96	5	91	1	96	394754.3370	0.0043	0.030	0.0044	0.25	Melli+22
1275:	97	6	92	1	97	96	6	91	1	96	394754.3370	0.0043	0.030	0.0044	0.25	Melli+22
1276:	98	4	94	1	98	97	5	93	1	97	394768.1600	-0.0145	0.030	-0.0145	0.25	Melli+22
1277:	98	5	94	1	98	97	4	93	1	97	394768.1600	-0.0145	0.030	-0.0145	0.25	Melli+22
1278:	98	5	94	1	98	97	5	93	1	97	394768.1600	-0.0145	0.030	-0.0145	0.25	Melli+22
1279:	98	4	94	1	98	97	4	93	1	97	394768.1600	-0.0145	0.030	-0.0145	0.25	Melli+22
1280:	100	2	98	1	100	99	3	97	1	99	394785.8360	0.0748	0.030	0.0748	0.25	Melli+22
1281:	100	3	98	1	100	99	2	97	1	99	394785.8360	0.0748	0.030	0.0748	0.25	Melli+22
1282:	100	3	98	1	100	99	3	97	1	99	394785.8360	0.0748	0.030	0.0748	0.25	Melli+22
1283:	100	2	98	1	100	99	2	97	1	99	394785.8360	0.0748	0.030	0.0748	0.25	Melli+22
1284:	101	2	100	1	101	100	1	99	1	100	394784.9250	0.0007	0.030	0.0007	0.25	Melli+22
1285:	101	1	100	1	101	100	2	99	1	100	394784.9250	0.0007	0.030	0.0007	0.25	Melli+22
1286:	101	2	100	1	101	100	2	99	1	100	394784.9250	0.0007	0.030	0.0007	0.25	Melli+22
1287:	101	1	100	1	101	100	1	99	1	100	394784.9250	0.0007	0.030	0.0007	0.25	Melli+22
1288:	91	12	80	1	91	90	11	79	1	90	394847.3740	0.0090	0.030	0.0091	0.25	Melli+22
1289:	91	11	80	1	91	90	12	79	1	90	394847.3740	0.0091	0.030	0.0091	0.25	Melli+22
1290:	91	11	80	1	91	90	11	79	1	90	394847.3740	0.0091	0.030	0.0091	0.25	Melli+22
1291:	91	12	80	1	91	90	12	79	1	90	394847.3740	0.0091	0.030	0.0091	0.25	Melli+22
1292:	95	9	87	1	95	94	8	86	1	94	398503.7410	0.0172	0.030	0.0173	0.25	Melli+22
1293:	95	8	87	1	95	94	9	86	1	94	398503.7410	0.0172	0.030	0.0173	0.25	Melli+22
1294:	95	8	87	1	95	94	8	86	1	94	398503.7410	0.0172	0.030	0.0173	0.25	Melli+22
1295:	95	9	87	1	95	94	9	86	1	94	398503.7410	0.0172	0.030	0.0173	0.25	Melli+22
1296:	96	7	89	1	96	95	8	88	1	95	398506.7260	-0.0070	0.030	-0.0071	0.25	Melli+22
1297:	96	8	89	1	96	95	7	88	1	95	398506.7260	-0.0070	0.030	-0.0071	0.25	Melli+22
1298:	96	8	89	1	96	95	8	88	1	95	398506.7260	-0.0070	0.030	-0.0071	0.25	Melli+22
1299:	96	7	89	1	96	95	7	88	1	95	398506.7260	-0.0070	0.030	-0.0071	0.25	Melli+22
1300:	94	10	85	1	94	93	9	84	1	93	398514.2770	-0.0117	0.030	-0.0117	0.25	Melli+22
1301:	94	9	85	1	94	93	10	84	1	93	398514.2770	-0.0117	0.030	-0.0117	0.25	Melli+22
1302:	94	9	85	1	94	93	9	84	1	93	398514.2770	-0.0117	0.030	-0.0117	0.25	Melli+22
1303:	94	10	85	1	94	93	10	84	1	93	398514.2770	-0.0117	0.030	-0.0117	0.25	Melli+22
1304:	97	6	91	1	97	96	7	90	1	96	398517.6830	-0.0465	0.030	-0.0466	0.25	Melli+22

```
1305: 97  7 91  1 97   96  6 90  1 96       398517.6830  -0.0465  0.030  -0.0466 0.25 Melli+22
1306: 97  6 91  1 97   96  6 90  1 96       398517.6830  -0.0465  0.030  -0.0466 0.25 Melli+22
1307: 97  7 91  1 97   96  7 90  1 96       398517.6830  -0.0465  0.030  -0.0466 0.25 Melli+22
1308: 98  6 93  1 98   97  5 92  1 97       398532.2950  -0.0381  0.030  -0.0382 0.25 Melli+22
1309: 98  5 93  1 98   97  6 92  1 97       398532.2950  -0.0381  0.030  -0.0382 0.25 Melli+22
1310: 98  5 93  1 98   97  5 92  1 97       398532.2950  -0.0381  0.030  -0.0382 0.25 Melli+22
1311: 98  6 93  1 98   97  6 92  1 97       398532.2950  -0.0381  0.030  -0.0382 0.25 Melli+22
1312: 99  4 95  1 99   98  5 94  1 98       398547.0180  -0.0101  0.030  -0.0101 0.25 Melli+22
1313: 99  5 95  1 99   98  4 94  1 98       398547.0180  -0.0101  0.030  -0.0101 0.25 Melli+22
1314: 99  4 95  1 99   98  4 94  1 98       398547.0180  -0.0101  0.030  -0.0101 0.25 Melli+22
1315: 99  5 95  1 99   98  5 94  1 98       398547.0180  -0.0101  0.030  -0.0101 0.25 Melli+22
1316:100  4 97  1100   99  3 96  1 99       398558.8650  -0.0623  0.030  -0.0624 0.25 Melli+22
1317:100  3 97  1100   99  4 96  1 99       398558.8650  -0.0623  0.030  -0.0624 0.25 Melli+22
1318:100  4 97  1100   99  4 96  1 99       398558.8650  -0.0623  0.030  -0.0624 0.25 Melli+22
1319:100  3 97  1100   99  3 96  1 99       398558.8650  -0.0623  0.030  -0.0624 0.25 Melli+22
1320:103  1103  1103  102  0102  1102       398555.2670   0.0264  0.030   0.0264 0.25 Melli+22
1321:103  0103  1103  102  1102  1102       398555.2670   0.0264  0.030   0.0264 0.25 Melli+22
1322:103  0103  1103  102  0102  1102       398555.2670   0.0264  0.030   0.0264 0.25 Melli+22
1323:103  1103  1103  102  1102  1102       398555.2670   0.0264  0.030   0.0264 0.25 Melli+22
1324: 92 12 81  1 92   91 11 80  1 91       398607.8580  -0.0486  0.030  -0.0486 0.25 Melli+22
1325: 92 11 81  1 92   91 12 80  1 91       398607.8580  -0.0486  0.030  -0.0486 0.25 Melli+22
1326: 92 11 81  1 92   91 11 80  1 91       398607.8580  -0.0486  0.030  -0.0486 0.25 Melli+22
1327: 92 12 81  1 92   91 12 80  1 91       398607.8580  -0.0486  0.030  -0.0486 0.25 Melli+22
1328: 92  5 88  1 92   91  4 87  1 91       372053.6670   0.0269  0.030   0.0633 0.12 Melli+22
1329: 92  4 88  1 92   91  5 87  1 91       372053.6670   0.0269  0.030   0.0633 0.12 Melli+22
1330: 88  9 80  1 88   87  8 79  1 87       372053.6670   0.0995  0.030   0.0633 0.12 Melli+22
1331: 88  8 80  1 88   87  9 79  1 87       372053.6670   0.0995  0.030   0.0633 0.12 Melli+22
1332: 92  5 88  1 92   91  5 87  1 91       372053.6670   0.0269  0.030   0.0633 0.12 Melli+22
1333: 92  4 88  1 92   91  4 87  1 91       372053.6670   0.0269  0.030   0.0633 0.12 Melli+22
1334: 88  8 80  1 88   87  8 79  1 87       372053.6670   0.0995  0.030   0.0633 0.12 Melli+22
1335: 88  9 80  1 88   87  9 79  1 87       372053.6670   0.0995  0.030   0.0633 0.12 Melli+22
1336: 99  1 99  1 99   98  0 98  1 98       383422.9200   0.1716  0.030   0.0090 0.12 Melli+22
1337: 99  0 99  1 99   98  1 98  1 98       383422.9200   0.1716  0.030   0.0090 0.12 Melli+22
1338: 99  0 99  1 99   98  0 98  1 98       383422.9200   0.1716  0.030   0.0090 0.12 Melli+22
1339: 99  1 99  1 99   98  1 98  1 98       383422.9200   0.1716  0.030   0.0090 0.12 Melli+22
1340: 90 10 81  1 90   89  9 80  1 89       383422.9200  -0.1537  0.030   0.0090 0.12 Melli+22
1341: 90  9 81  1 90   89 10 80  1 89       383422.9200  -0.1537  0.030   0.0090 0.12 Melli+22
1342: 90  9 81  1 90   89  9 80  1 89       383422.9200  -0.1537  0.030   0.0090 0.12 Melli+22
1343: 90 10 81  1 90   89 10 80  1 89       383422.9200  -0.1537  0.030   0.0090 0.12 Melli+22
1344: 99  4 96  1 99   98  3 95  1 98       394779.5480   0.0739  0.030   0.0094 0.12 Melli+22
1345: 99  3 96  1 99   98  4 95  1 98       394779.5480   0.0739  0.030   0.0094 0.12 Melli+22
1346: 92 11 82  1 92   91 10 81  1 91       394779.5480  -0.0551  0.030   0.0094 0.12 Melli+22
1347: 92 10 82  1 92   91 11 81  1 91       394779.5480  -0.0551  0.030   0.0094 0.12 Melli+22
1348: 99  3 96  1 99   98  3 95  1 98       394779.5480   0.0739  0.030   0.0094 0.12 Melli+22
1349: 99  4 96  1 99   98  4 95  1 98       394779.5480   0.0739  0.030   0.0094 0.12 Melli+22
1350: 92 10 82  1 92   91 10 81  1 91       394779.5480  -0.0551  0.030   0.0094 0.12 Melli+22
1351: 92 11 82  1 92   91 11 81  1 91       394779.5480  -0.0551  0.030   0.0094 0.12 Melli+22
1352:102  2101  1102  101  1100  1101       398565.2520   0.2657  0.030  -0.0446 0.12 Melli+22
1353:102  1101  1102  101  2100  1101       398565.2520   0.2657  0.030  -0.0446 0.12 Melli+22
1354:102  1101  1102  101  1100  1101       398565.2520   0.2657  0.030  -0.0446 0.12 Melli+22
1355:102  2101  1102  101  2100  1101       398565.2520   0.2657  0.030  -0.0446 0.12 Melli+22
1356:101  2 99  1101  100  3 98  1100       398565.2520  -0.3549  0.030  -0.0446 0.12 Melli+22
1357:101  3 99  1101  100  2 98  1100       398565.2520  -0.3549  0.030  -0.0446 0.12 Melli+22
1358:101  3 99  1101  100  3 98  1100       398565.2520  -0.3549  0.030  -0.0446 0.12 Melli+22
1359:101  2 99  1101  100  2 98  1100       398565.2520  -0.3549  0.030  -0.0446 0.12 Melli+22
-------------------------------------------------------------------------------------
```

allenylacetylene Wed Jul 13 14:33:38 2022

/ instead of : below denotes (o-c)>3*err

							obs	o-c	error	blends o-c	wt	Notes
1:	2	1	2	1	1	1	9854.4090	-0.0012	0.001			McCarthy+20
2:	2	0	2	1	0	1	10056.5320	-0.0015	0.001			McCarthy+20
3:	2	1	1	1	1	0	10261.9960	-0.0020	0.001			McCarthy+20
4:	3	1	3	2	1	2	14780.7360	0.0004	0.001			McCarthy+20
5:	3	0	3	2	0	2	15081.4110	0.0006	0.001			McCarthy+20
6:	3	2	2	2	2	1	15088.7750	-0.0002	0.001			McCarthy+20
7:	3	1	2	2	1	1	15392.0770	-0.0003	0.001			McCarthy+20
8:	4	1	4	3	1	3	19706.0130	0.0000	0.001			McCarthy+20
9:	4	0	4	3	0	3	20102.2240	0.0009	0.001			McCarthy+20
10:	4	2	3	3	2	2	20117.2060	0.0016	0.001			McCarthy+20
11:	4	2	2	3	2	1	20130.4810	-0.0008	0.001			McCarthy+20
12:	4	1	3	3	1	2	20521.0460	0.0001	0.001			McCarthy+20
13:	5	1	5	4	1	4	24629.9060	-0.0004	0.001			McCarthy+20
14:	5	2	4	4	2	3	25144.6380	0.0002	0.001			McCarthy+20
15:	7	1	7	6	1	6	34472.2620	0.0054	0.010			Cernicharo+21
16:	7	0	7	6	0	6	35126.9230	0.0054	0.010			Cernicharo+21
17:	7	2	6	6	2	5	35195.5320	0.0067	0.010			Cernicharo+21
18:	7	3	5	6	3	4	35222.5310	0.0200	0.010			Cernicharo+21
19:	7	3	4	6	3	3	35223.1950	0.0029	0.010			Cernicharo+21
20:	7	2	5	6	2	4	35269.6640	0.0076	0.010			Cernicharo+21
21:	7	1	6	6	1	5	35897.4180	0.0085	0.010			Cernicharo+21
22:	8	1	8	7	1	7	39390.1160	0.0081	0.010			Cernicharo+21
23:	8	0	8	7	0	7	40118.2520	0.0066	0.010			Cernicharo+21
24:	8	2	7	7	2	6	40218.5110	0.0256	0.020			Cernicharo+21
25:	8	2	6	7	2	5	40329.4480	-0.0000	0.010			Cernicharo+21
26:	8	1	7	7	1	6	41018.0020	0.0062	0.010			Cernicharo+21
27:	9	1	9	8	1	8	44305.3690	-0.0004	0.010			Cernicharo+21
28:	9	0	9	8	0	8	45099.0940	0.0069	0.020			Cernicharo+21
29:	9	2	8	8	2	7	45239.4520	-0.0102	0.010			Cernicharo+21
30:	9	3	6	8	3	5	45294.3820	-0.0091	0.020			Cernicharo+21
31:	9	2	7	8	2	6	45397.5220	0.0072	0.020			Cernicharo+21
32:	9	1	8	8	1	7	46135.4310	0.0005	0.010			Cernicharo+21
33:	10	1	10	9	1	9	49217.7840	-0.0029	0.010			Cernicharo+21
34:	16	2	15	15	2	14	80310.5630	-0.0014	0.035			Melli+22
35:	16	4	13	15	4	12	80551.1340	-0.0005	0.035			Melli+22
36:	16	4	12	15	4	11	80552.0600	0.0143	0.035			Melli+22
37:	16	5	11	15	5	10	80553.4260	-0.0082	0.035	-0.0036	0.50	Melli+22
38:	16	5	12	15	5	11	80553.4260	0.0010	0.035	-0.0036	0.50	Melli+22
39:	16	3	14	15	3	13	80562.9140	-0.0159	0.035			Melli+22
40:	16	7	9	15	7	8	80599.4650	0.0107	0.035	0.0108	0.50	Melli+22
41:	16	7	10	15	7	9	80599.4650	0.0107	0.035	0.0108	0.50	Melli+22
42:	16	3	13	15	3	12	80608.4950	0.0083	0.035			Melli+22
43:	16	8	8	15	8	7	80635.2460	0.0053	0.035	0.0053	0.50	Melli+22
44:	16	8	9	15	8	8	80635.2460	0.0053	0.035	0.0053	0.50	Melli+22
45:	16	9	7	15	9	6	80677.6130	0.0183	0.035	0.0183	0.50	Melli+22
46:	16	9	8	15	9	7	80677.6130	0.0183	0.035	0.0183	0.50	Melli+22
47:	16	2	14	15	2	13	81153.0330	-0.0075	0.035			Melli+22
48:	16	1	15	15	1	14	81827.3200	0.0034	0.035			Melli+22
49:	17	1	17	16	1	16	83508.9440	0.0146	0.035			Melli+22
50:	17	0	17	16	0	16	84473.8600	-0.0100	0.035			Melli+22
51:	17	5	12	16	5	11	85590.5240	-0.0195	0.035	-0.0114	0.50	Melli+22
52:	17	5	13	16	5	12	85590.5240	-0.0033	0.035	-0.0114	0.50	Melli+22
53:	17	4	13	16	4	12	85592.3710	-0.0072	0.035			Melli+22
54:	17	3	15	16	3	14	85604.5440	-0.0039	0.035			Melli+22
55:	17	6	11	16	6	10	85607.9870	-0.0096	0.035	-0.0096	0.50	Melli+22
56:	17	6	12	16	6	11	85607.9870	-0.0095	0.035	-0.0096	0.50	Melli+22
57:	17	7	10	16	7	9	85636.8720	0.0068	0.035	0.0069	0.50	Melli+22
58:	17	7	11	16	7	10	85636.8720	0.0068	0.035	0.0069	0.50	Melli+22
59:	17	3	14	16	3	13	85666.1400	0.0050	0.035			Melli+22
60:	17	8	9	16	8	8	85674.2870	0.0132	0.035	0.0133	0.50	Melli+22
61:	17	8	10	16	8	9	85674.2870	0.0132	0.035	0.0133	0.50	Melli+22
62:	17	9	8	16	9	7	85718.8770	0.0223	0.035	0.0223	0.50	Melli+22

63:	17	9	9	16	9	8	85718.8770	0.0223	0.035	0.0223	0.50	Melli+22
64:	17	2	15	16	2	14	86301.1460	0.0010	0.035			Melli+22
65:	17	1	16	16	1	15	86900.7090	-0.0104	0.035			Melli+22
66:	18	1	18	17	1	17	88392.4940	-0.0010	0.035			Melli+22
67:	18	0	18	17	0	17	89334.1100	-0.0030	0.035			Melli+22
68:	18	2	17	17	2	16	90299.5800	-0.0036	0.035			Melli+22
69:	18	5	13	17	5	12	90628.0760	-0.0309	0.035	-0.0173	0.50	Melli+22
70:	18	5	14	17	5	13	90628.0760	-0.0035	0.035	-0.0173	0.50	Melli+22
71:	18	4	15	17	4	14	90631.7150	-0.0243	0.035			Melli+22
72:	18	4	14	17	4	13	90633.8110	-0.0212	0.035			Melli+22
73:	18	6	12	17	6	11	90644.7670	-0.0012	0.035	-0.0012	0.50	Melli+22
74:	18	6	13	17	6	12	90644.7670	-0.0010	0.035	-0.0012	0.50	Melli+22
75:	18	3	16	17	3	15	90646.5080	-0.0084	0.035			Melli+22
76:	18	7	11	17	7	10	90674.2740	0.0110	0.035	0.0110	0.50	Melli+22
77:	18	7	12	17	7	11	90674.2740	0.0110	0.035	0.0110	0.50	Melli+22
78:	18	8	10	17	8	9	90713.1930	0.0101	0.035	0.0101	0.50	Melli+22
79:	18	8	11	17	8	10	90713.1930	0.0101	0.035	0.0101	0.50	Melli+22
80:	18	3	15	17	3	14	90728.2740	0.0118	0.035			Melli+22
81:	18	9	9	17	9	8	90759.9340	0.0190	0.035	0.0190	0.50	Melli+22
82:	18	9	10	17	9	9	90759.9340	0.0190	0.035	0.0190	0.50	Melli+22
83:	18	2	16	17	2	15	91457.3490	-0.0169	0.035			Melli+22
84:	18	1	17	17	1	16	91965.8000	-0.0159	0.035			Melli+22
85:	19	1	19	18	1	18	93272.0950	-0.0130	0.035			Melli+22
86:	19	0	19	18	0	18	94182.5000	0.0009	0.035			Melli+22
87:	19	2	18	18	2	17	95287.8000	0.0150	0.035			Melli+22
88:	19	5	14	18	5	13	95666.1260	-0.0247	0.035	-0.0023	0.50	Melli+22
89:	19	5	15	18	5	14	95666.1260	0.0202	0.035	-0.0023	0.50	Melli+22
90:	19	4	16	18	4	15	95673.4360	-0.0047	0.035			Melli+22
91:	19	4	15	18	4	14	95676.5040	0.0021	0.035			Melli+22
92:	19	6	13	18	6	12	95681.7130	0.0054	0.035	0.0056	0.50	Melli+22
93:	19	6	14	18	6	13	95681.7130	0.0058	0.035	0.0056	0.50	Melli+22
94:	19	3	17	18	3	16	95688.6350	-0.0088	0.035			Melli+22
95:	19	3	16	18	3	15	95795.3630	-0.0129	0.035			Melli+22
96:	19	2	17	18	2	16	96620.3800	-0.0156	0.035			Melli+22
97:	19	1	18	18	1	17	97021.8920	0.0120	0.035			Melli+22
98:	20	1	20	19	1	19	98147.7700	-0.0128	0.035			Melli+22
99:	20	0	20	19	0	19	99020.2170	-0.0068	0.035			Melli+22
100:	20	2	19	19	2	18	100271.5350	0.0205	0.035			Melli+22
101:	20	5	15	19	5	14	100704.6820	-0.0195	0.035	0.0163	0.50	Melli+22
102:	20	5	16	19	5	15	100704.6820	0.0522	0.035	0.0163	0.50	Melli+22
103:	20	4	17	19	4	16	100716.0950	-0.0083	0.035			Melli+22
104:	20	6	14	19	6	13	100718.8370	0.0144	0.035	0.0148	0.50	Melli+22
105:	20	6	15	19	6	14	100718.8370	0.0151	0.035	0.0148	0.50	Melli+22
106:	20	4	16	19	4	15	100720.5080	0.0175	0.035			Melli+22
107:	20	3	18	19	3	17	100730.7180	0.0040	0.035			Melli+22
108:	20	7	13	19	7	12	100749.0260	0.0133	0.035	0.0133	0.50	Melli+22
109:	20	7	14	19	7	13	100749.0260	0.0133	0.035	0.0133	0.50	Melli+22
110:	20	8	12	19	8	11	100790.6340	0.0363	0.035	0.0364	0.50	Melli+22
111:	20	8	13	19	8	12	100790.6340	0.0363	0.035	0.0364	0.50	Melli+22
112:	20	9	11	19	9	10	100841.4180	0.0301	0.035	0.0302	0.50	Melli+22
113:	20	9	12	19	9	11	100841.4180	0.0301	0.035	0.0302	0.50	Melli+22
114:	20	2	18	19	2	17	101788.7200	-0.0094	0.035			Melli+22
115:	20	1	19	19	1	18	102068.1600	-0.0002	0.035			Melli+22
116:	21	1	21	20	1	20	103019.5480	-0.0104	0.035			Melli+22
117:	21	0	21	20	0	20	103848.5820	-0.0307	0.035			Melli+22
118:	21	2	20	20	2	19	105250.5850	0.0010	0.035			Melli+22
119:	21	6	15	20	6	14	105756.1150	-0.0062	0.035	-0.0056	0.50	Melli+22
120:	21	6	16	20	6	15	105756.1150	-0.0050	0.035	-0.0056	0.50	Melli+22
121:	21	4	18	20	4	17	105759.7400	0.0015	0.035			Melli+22
122:	21	4	17	20	4	16	105765.9000	-0.0113	0.035			Melli+22
123:	21	3	19	20	3	18	105772.4680	-0.0196	0.035			Melli+22
124:	21	7	14	20	7	13	105786.3680	0.0071	0.035	0.0071	0.50	Melli+22
125:	21	7	15	20	7	14	105786.3680	0.0071	0.035	0.0071	0.50	Melli+22
126:	21	8	13	20	8	12	105829.0980	0.0108	0.035	0.0109	0.50	Melli+22
127:	21	8	14	20	8	13	105829.0980	0.0108	0.035	0.0109	0.50	Melli+22
128:	21	3	18	20	3	17	105946.7060	-0.0089	0.035			Melli+22
129:	21	2	19	20	2	18	106960.7050	-0.0195	0.035			Melli+22
130:	21	1	20	20	1	19	107103.8820	-0.0052	0.035			Melli+22
131:	22	1	22	21	1	21	107887.5000	0.0036	0.035			Melli+22

132:	22	0	22	21	0	21	108669.0470	-0.0078	0.035			Melli+22
133:	22	2	21	21	2	20	110224.8120	-0.0038	0.035			Melli+22
134:	22	5	17	21	5	16	110783.3430	-0.0901	0.035	-0.0049	0.50	Melli+22
135:	22	5	18	21	5	17	110783.3430	0.0803	0.035	-0.0049	0.50	Melli+22
136:	22	6	16	21	6	15	110793.6020	-0.0093	0.035	-0.0083	0.50	Melli+22
137:	22	6	17	21	6	16	110793.6020	-0.0073	0.035	-0.0083	0.50	Melli+22
138:	22	3	20	21	3	19	110813.6960	-0.0091	0.035			Melli+22
139:	22	7	15	21	7	14	110823.6830	-0.0056	0.035	-0.0056	0.50	Melli+22
140:	22	7	16	21	7	15	110823.6830	-0.0055	0.035	-0.0056	0.50	Melli+22
141:	22	8	14	21	8	13	110867.4260	0.0055	0.035	0.0055	0.50	Melli+22
142:	22	8	15	21	8	14	110867.4260	0.0055	0.035	0.0055	0.50	Melli+22
143:	22	9	13	21	9	12	110921.9360	0.0210	0.035	0.0211	0.50	Melli+22
144:	22	9	14	21	9	13	110921.9360	0.0210	0.035	0.0211	0.50	Melli+22
145:	22	1	21	21	1	20	112128.2140	-0.0677	0.035			Melli+22
146:	23	1	23	22	1	22	112751.6240	-0.0544	0.035			Melli+22
147:	23	0	23	22	0	22	113482.9330	-0.0068	0.035			Melli+22
148:	23	1	22	22	1	21	117140.5590	-0.0086	0.035			Melli+22
149:	23	2	21	22	2	20	117308.8100	-0.0044	0.035			Melli+22
150:	24	1	24	23	1	23	117612.2010	-0.0036	0.035			Melli+22
151:	24	0	24	23	0	23	118291.5950	-0.0061	0.035			Melli+22
152:	24	2	23	23	2	22	120158.1110	-0.0048	0.035			Melli+22
153:	24	1	23	23	1	22	122139.9600	-0.0265	0.035			Melli+22
154:	45	7	38	44	7	37	226669.8730	-0.1399	0.035	-0.0494	0.50	Melli+22
155:	45	7	39	44	7	38	226669.8730	0.0410	0.035	-0.0494	0.50	Melli+22
156:	45	8	37	44	8	36	226685.4090	-0.0083	0.035	-0.0061	0.50	Melli+22
157:	45	8	38	44	8	37	226685.4090	-0.0038	0.035	-0.0061	0.50	Melli+22
158:	45	11	34	44	11	33	226961.7010	0.0159	0.035	0.0160	0.50	Melli+22
159:	45	11	35	44	11	34	226961.7010	0.0159	0.035	0.0160	0.50	Melli+22
160:	45	12	33	44	12	32	227104.1100	-0.0667	0.035	-0.0668	0.50	Melli+22
161:	45	12	34	44	12	33	227104.1100	-0.0667	0.035	-0.0668	0.50	Melli+22
162:	47	1	47	46	1	46	228704.4820	0.0272	0.035			Melli+22
163:	47	0	47	46	0	46	228747.4480	0.0232	0.035			Melli+22
164:	46	1	45	45	1	44	228804.0660	-0.0216	0.035			Melli+22
165:	45	2	43	44	2	42	228865.1540	-0.0341	0.035			Melli+22
166:	46	7	39	45	7	38	231705.7510	-0.1945	0.035	-0.0749	0.50	Melli+22
167:	46	7	40	45	7	39	231705.7510	0.0447	0.035	-0.0749	0.50	Melli+22
168:	46	8	38	45	8	37	231717.2390	-0.0117	0.035	-0.0086	0.50	Melli+22
169:	46	8	39	45	8	38	231717.2390	-0.0054	0.035	-0.0086	0.50	Melli+22
170:	46	5	42	45	5	41	231914.5020	0.0463	0.035			Melli+22
171:	46	4	43	45	4	42	231920.9630	0.0107	0.035			Melli+22
172:	46	5	41	45	5	40	232029.8350	-0.0259	0.035			Melli+22
173:	47	2	46	46	2	45	232812.4500	-0.0280	0.035			Melli+22
174:	46	4	42	45	4	41	233056.6080	-0.0109	0.035			Melli+22
175:	46	2	44	45	2	43	233769.1470	-0.0281	0.035			Melli+22
176:	46	3	43	45	3	42	234984.9880	-0.0132	0.035			Melli+22
177:	47	3	45	46	3	44	235792.2990	-0.0159	0.035			Melli+22
178:	47	8	39	46	8	38	236748.6670	-0.0210	0.035	-0.0168	0.50	Melli+22
179:	47	8	40	46	8	39	236748.6670	-0.0124	0.035	-0.0168	0.50	Melli+22
180:	47	9	38	46	9	37	236806.5560	0.0250	0.035	0.0252	0.50	Melli+22
181:	47	9	39	46	9	38	236806.5560	0.0252	0.035	0.0252	0.50	Melli+22
182:	47	6	42	46	6	41	236810.0810	-0.0133	0.035			Melli+22
183:	47	6	41	46	6	40	236818.2240	-0.0219	0.035			Melli+22
184:	47	4	44	46	4	43	236947.7530	-0.0046	0.035			Melli+22
185:	47	5	43	46	5	42	236966.3000	-0.0065	0.035			Melli+22
186:	47	11	36	46	11	35	237023.5520	0.0173	0.035	0.0173	0.50	Melli+22
187:	47	11	37	46	11	36	237023.5520	0.0173	0.035	0.0173	0.50	Melli+22
188:	47	12	35	46	12	34	237169.8580	-0.0242	0.035	-0.0242	0.50	Melli+22
189:	47	12	36	46	12	35	237169.8580	-0.0242	0.035	-0.0242	0.50	Melli+22
190:	48	2	47	47	2	46	237651.4750	-0.0430	0.035			Melli+22
191:	49	1	49	48	1	48	238325.5660	-0.0366	0.035			Melli+22
192:	48	1	47	47	1	46	238341.0540	-0.0917	0.035			Melli+22
193:	49	0	49	48	0	48	238357.9260	0.0550	0.035			Melli+22
194:	47	2	45	46	2	44	238655.9310	-0.0069	0.035			Melli+22
195:	48	3	46	47	3	45	240721.0200	0.0873	0.035			Melli+22
196:	48	8	40	47	8	39	241779.7030	-0.0157	0.035	-0.0099	0.50	Melli+22
197:	48	8	41	47	8	40	241779.7030	-0.0040	0.035	-0.0099	0.50	Melli+22
198:	48	9	39	47	9	38	241835.5660	-0.0055	0.035	-0.0055	0.50	Melli+22
199:	48	9	40	47	9	39	241835.5660	-0.0053	0.035	-0.0055	0.50	Melli+22
200:	48	6	43	47	6	42	241854.1490	-0.0066	0.035	-0.0066	0.50	Melli+22

201: 48 6 43	47 6 42	241854.1490	-0.0066	0.035	-0.0066 0.50	Melli+22
202: 48 10 38	47 10 37	241929.6090	0.0368	0.035	0.0368 0.50	Melli+22
203: 48 10 39	47 10 38	241929.6090	0.0368	0.035	0.0368 0.50	Melli+22
204: 48 5 44	47 5 43	242017.8340	0.0115	0.035		Melli+22
205: 48 12 36	47 12 35	242201.3900	0.0133	0.035	0.0134 0.50	Melli+22
206: 48 12 37	47 12 36	242201.3900	0.0133	0.035	0.0134 0.50	Melli+22
207: 49 2 48	48 2 47	242486.9280	0.0012	0.035		Melli+22
208: 49 1 48	48 1 47	243110.9000	0.0070	0.035		Melli+22
209: 50 1 50	49 1 49	243134.7420	0.0369	0.035		Melli+22
210: 50 0 50	49 0 49	243162.5640	-0.0700	0.035		Melli+22
211: 48 2 46	47 2 45	243525.7600	0.0361	0.035		Melli+22
212: 48 3 45	47 3 44	245263.3050	-0.0004	0.035		Melli+22
213: 49 7 43	48 7 42	246812.5840	-0.0364	0.035		Melli+22
214: 49 7 42	48 7 41	246813.1040	-0.0486	0.035		Melli+22
215: 49 9 40	48 9 39	246863.9850	-0.0120	0.035	-0.0119 0.50	Melli+22
216: 49 9 41	48 9 40	246863.9850	-0.0116	0.035	-0.0119 0.50	Melli+22
217: 49 6 44	48 6 43	246898.4370	0.0188	0.035		Melli+22
218: 49 6 43	48 6 42	246911.0440	-0.0307	0.035		Melli+22
219: 49 4 46	48 4 45	246989.1050	-0.0088	0.035		Melli+22
220: 49 5 45	48 5 44	247068.8270	-0.0152	0.035		Melli+22
221: 49 11 38	48 11 37	247081.9860	-0.0546	0.035	-0.0546 0.50	Melli+22
222: 49 11 39	48 11 38	247081.9860	-0.0546	0.035	-0.0546 0.50	Melli+22
223: 49 12 37	48 12 36	247232.0240	0.0849	0.035	0.0849 0.50	Melli+22
224: 49 12 38	48 12 37	247232.0240	0.0849	0.035	0.0849 0.50	Melli+22
225: 49 5 44	48 5 43	247264.6260	-0.0005	0.035		Melli+22
226: 50 1 49	49 1 48	247881.8450	0.0100	0.035		Melli+22
227: 51 1 51	50 1 50	247942.8790	0.0206	0.035		Melli+22
228: 51 0 51	50 0 50	247967.0260	0.0118	0.035		Melli+22
229: 49 2 47	48 2 46	248378.9550	0.0028	0.035		Melli+22
230: 49 4 45	48 4 44	248604.3260	0.0349	0.035		Melli+22
231: 49 3 46	48 3 45	250382.8450	0.0190	0.035		Melli+22
232: 50 3 48	49 3 47	250558.4300	-0.0278	0.035		Melli+22
233: 50 8 42	49 8 41	251840.5030	-0.0152	0.035	-0.0046 0.50	Melli+22
234: 50 8 43	49 8 42	251840.5030	0.0060	0.035	-0.0046 0.50	Melli+22
235: 50 7 44	49 7 43	251847.9040	-0.0983	0.035		Melli+22
236: 50 7 43	49 7 42	251848.7670	0.0780	0.035		Melli+22
237: 50 9 41	49 9 40	251891.7920	-0.0007	0.035	-0.0005 0.50	Melli+22
238: 50 9 42	49 9 41	251891.7920	-0.0002	0.035	-0.0005 0.50	Melli+22
239: 50 6 44	49 6 43	251958.4900	-0.0178	0.035		Melli+22
240: 50 4 47	49 4 46	252003.1350	0.0546	0.035		Melli+22
241: 50 5 46	49 5 45	252119.2740	0.0830	0.035		Melli+22
242: 51 2 50	50 2 49	252147.6250	0.0390	0.035		Melli+22
243: 51 1 50	50 1 49	252654.1460	0.0099	0.035		Melli+22
244: 52 0 52	51 0 51	252770.9630	0.0033	0.035		Melli+22
245: 50 4 46	49 4 45	253800.7480	0.0941	0.035		Melli+22
246: 50 3 47	49 3 46	255488.2330	-0.0205	0.035		Melli+22
247: 51 7 44	50 7 43	256884.1650	0.0389	0.035		Melli+22
248: 52 2 51	51 2 50	256973.2300	0.0369	0.035		Melli+22
249: 51 6 46	50 6 45	256987.4760	0.0314	0.035		Melli+22
250: 51 6 45	50 6 44	257006.7150	0.0124	0.035		Melli+22
251: 51 4 48	50 4 47	257012.2250	0.0259	0.035		Melli+22
252: 51 5 47	50 5 46	257168.6700	-0.0102	0.035		Melli+22
253: 52 1 51	51 1 50	257427.8700	-0.0110	0.035		Melli+22
254: 51 5 46	50 5 45	257440.7820	-0.0090	0.035		Melli+22
255: 53 1 53	52 1 52	257556.4000	0.0103	0.035		Melli+22
256: 53 0 53	52 0 52	257574.4110	-0.0125	0.035		Melli+22
257: 52 3 50	51 3 49	260369.8940	-0.0122	0.035		Melli+22
258: 51 3 48	50 3 47	260578.9270	-0.0105	0.035		Melli+22
259: 53 2 52	52 2 51	261795.8980	0.0186	0.035		Melli+22
260: 52 8 44	51 8 43	261899.5670	0.0019	0.035	0.0208 0.50	Melli+22
261: 52 8 45	51 8 44	261899.5670	0.0397	0.035	0.0208 0.50	Melli+22
262: 52 4 49	51 4 48	262016.1720	-0.0327	0.035		Melli+22
263: 52 6 47	51 6 46	262032.1440	-0.0014	0.035		Melli+22
264: 52 6 46	51 6 45	262055.7310	0.0000	0.035		Melli+22
265: 53 1 52	52 1 51	262203.1460	0.0554	0.035		Melli+22
266: 52 5 48	51 5 47	262217.0750	-0.0334	0.035		Melli+22
267: 54 0 54	53 0 53	262377.3100	-0.0524	0.035		Melli+22
268: 52 5 47	51 5 46	262535.7680	-0.0127	0.035		Melli+22
269: 53 3 51	52 3 50	265265.9970	-0.0044	0.035		Melli+22

270:	52	3	49	51	3	48	265654.3280	0.0340	0.035			Melli+22
271:	53	7	46	52	7	45	266954.7090	-0.0026	0.035			Melli+22
272:	54	1	53	53	1	52	266979.7690	0.0348	0.035			Melli+22
273:	55	1	55	54	1	54	267166.2840	-0.0202	0.035			Melli+22
274:	55	0	55	54	0	54	267179.7880	0.0514	0.035			Melli+22
275:	53	5	49	52	5	48	267264.2860	0.0247	0.035			Melli+22
276:	53	5	48	52	5	47	267635.8420	-0.0114	0.035			Melli+22
277:	53	4	49	52	4	48	269418.1530	-0.0109	0.035			Melli+22
278:	53	3	50	52	3	49	270713.8380	0.0430	0.035			Melli+22
279:	54	8	46	53	8	45	271956.7340	-0.0422	0.035	-0.0095	0.50	Melli+22
280:	54	8	47	53	8	46	271956.7340	0.0232	0.035	-0.0095	0.50	Melli+22
281:	56	0	56	55	0	55	271981.5080	-0.0011	0.035			Melli+22
282:	54	7	47	53	7	46	271989.9210	0.0535	0.035			Melli+22
283:	54	6	49	53	6	48	272121.7920	0.0680	0.035			Melli+22
284:	54	2	52	53	2	51	272423.6350	0.0481	0.035			Melli+22
285:	54	4	50	53	4	49	274629.2370	-0.0119	0.035			Melli+22
286:	56	1	55	55	1	54	276536.9800	-0.0396	0.035			Melli+22
287:	57	1	57	56	1	56	276772.7030	0.0412	0.035			Melli+22
288:	57	0	57	56	0	56	276782.6640	0.0173	0.035			Melli+22
289:	55	8	47	54	8	46	276984.5720	-0.0959	0.035	-0.0532	0.50	Melli+22
290:	55	8	48	54	8	47	276984.5720	-0.0104	0.035	-0.0532	0.50	Melli+22
291:	55	4	52	54	4	51	276995.0130	-0.0926	0.035			Melli+22
292:	55	7	49	54	7	48	277022.7400	0.0635	0.035			Melli+22
293:	55	7	48	54	7	47	277024.9450	0.0055	0.035			Melli+22
294:	55	10	45	54	10	44	277108.8670	0.0006	0.035	0.0007	0.50	Melli+22
295:	55	10	46	54	10	45	277108.8670	0.0007	0.035	0.0007	0.50	Melli+22
296:	55	6	50	54	6	49	277166.5450	0.0414	0.035			Melli+22
297:	55	2	53	54	2	52	277195.7030	-0.0336	0.035			Melli+22
298:	55	6	49	54	6	48	277208.6570	-0.0233	0.035			Melli+22
299:	55	5	51	54	5	50	277353.7630	-0.0625	0.035			Melli+22
300:	55	12	43	54	12	42	277394.5820	-0.0530	0.035	-0.0531	0.50	Melli+22
301:	55	12	44	54	12	43	277394.5820	-0.0530	0.035	-0.0531	0.50	Melli+22
302:	55	5	50	54	5	49	277852.6770	-0.0246	0.035			Melli+22
303:	56	3	54	55	3	53	279917.0050	0.0856	0.035			Melli+22
304:	56	9	47	55	9	46	282044.4640	-0.0564	0.035	-0.0549	0.50	Melli+22
305:	56	9	48	55	9	47	282044.4640	-0.0532	0.035	-0.0549	0.50	Melli+22
306:	56	5	51	55	5	50	282970.2110	0.0246	0.035			Melli+22
307:	56	4	52	55	4	51	285050.4170	0.0429	0.035			Melli+22
308:	56	3	53	55	3	52	285792.6280	0.0519	0.035			Melli+22
309:	58	1	57	57	1	56	286098.8470	-0.0494	0.035			Melli+22
310:	59	1	59	58	1	58	286375.3860	-0.0976	0.035			Melli+22
311:	59	0	59	58	0	58	286382.8490	-0.0443	0.035			Melli+22
312:	57	2	55	56	2	54	286713.7460	0.0161	0.035			Melli+22
313:	57	4	54	56	4	53	286951.2810	-0.0105	0.035			Melli+22
314:	57	8	50	56	8	49	287038.8240	-0.0067	0.035			Melli+22
315:	57	8	49	56	8	48	287038.9670	-0.0070	0.035			Melli+22
316:	57	9	48	56	9	47	287067.5570	0.0601	0.035	0.0623	0.50	Melli+22
317:	57	9	49	56	9	48	287067.5570	0.0645	0.035	0.0623	0.50	Melli+22
318:	57	7	51	56	7	50	287091.3860	0.0457	0.035			Melli+22
319:	57	7	50	56	7	49	287094.9140	0.0492	0.035			Melli+22
320:	57	6	52	56	6	51	287255.7280	-0.0175	0.035			Melli+22
321:	57	11	46	56	11	45	287279.6180	-0.0307	0.035	-0.0307	0.50	Melli+22
322:	57	11	47	56	11	46	287279.6180	-0.0307	0.035	-0.0307	0.50	Melli+22
323:	57	12	45	56	12	44	287440.4670	-0.0478	0.035	-0.0478	0.50	Melli+22
324:	57	12	46	56	12	45	287440.4670	-0.0478	0.035	-0.0478	0.50	Melli+22
325:	57	5	52	56	5	51	288094.1090	-0.0443	0.035			Melli+22
326:	58	3	56	57	3	55	289654.3220	-0.0054	0.035			Melli+22
327:	58	2	56	57	2	55	291462.3860	0.0343	0.035			Melli+22
328:	58	12	46	57	12	45	292461.8020	0.0022	0.035	0.0022	0.50	Melli+22
329:	58	12	47	57	12	46	292461.8020	0.0022	0.035	0.0022	0.50	Melli+22
330:	59	3	57	58	3	56	294514.6200	0.0999	0.035			Melli+22
331:	60	2	59	59	2	58	295485.4650	0.0076	0.035			Melli+22
332:	60	1	59	59	1	58	295664.2890	-0.0215	0.035			Melli+22
333:	61	0	61	60	0	60	295980.3380	0.0855	0.035			Melli+22
334:	59	4	56	58	4	55	296881.9670	-0.0425	0.035			Melli+22
335:	59	9	50	58	9	49	297111.1230	-0.0698	0.035	-0.0660	0.50	Melli+22
336:	59	9	51	58	9	50	297111.1230	-0.0622	0.035	-0.0660	0.50	Melli+22
337:	59	6	54	58	6	53	297344.0590	-0.0165	0.035			Melli+22
338:	59	6	53	58	6	52	297430.4430	-0.0112	0.035			Melli+22

339:	59 5 55	58 5 54	297506.9790	-0.0478	0.035			Melli+22
340:	61 1 60	60 1 59	300448.0060	0.0074	0.035			Melli+22
341:	59 3 56	58 3 55	300713.9060	-0.0208	0.035			Melli+22
342:	60 8 52	59 8 51	302116.4670	-0.1309	0.035	0.0190	0.50	Melli+22
343:	60 8 53	59 8 52	302116.4670	0.1689	0.035	0.0190	0.50	Melli+22
344:	60 9 51	59 9 50	302131.8840	0.0008	0.035	0.0059	0.50	Melli+22
345:	60 9 52	59 9 51	302131.8840	0.0108	0.035	0.0059	0.50	Melli+22
346:	60 7 54	59 7 53	302192.7480	-0.0121	0.035			Melli+22
347:	60 7 53	59 7 52	302199.3590	-0.0313	0.035			Melli+22
348:	60 10 50	59 10 49	302211.0740	-0.0221	0.035	-0.0220	0.50	Melli+22
349:	60 10 51	59 10 50	302211.0740	-0.0218	0.035	-0.0220	0.50	Melli+22
350:	60 11 49	59 11 48	302337.4220	0.0259	0.035	0.0259	0.50	Melli+22
351:	60 11 50	59 11 49	302337.4220	0.0259	0.035	0.0259	0.50	Melli+22
352:	60 6 55	59 6 54	302387.6650	-0.0177	0.035			Melli+22
353:	60 6 54	59 6 53	302490.0490	0.0283	0.035			Melli+22
354:	60 12 48	59 12 47	302500.9510	-0.0104	0.035	-0.0104	0.50	Melli+22
355:	60 12 49	59 12 48	302500.9510	-0.0104	0.035	-0.0104	0.50	Melli+22
356:	60 5 56	59 5 55	302538.3750	-0.0119	0.035			Melli+22
357:	60 13 47	59 13 46	302695.6380	0.0369	0.035	0.0370	0.50	Melli+22
358:	60 13 48	59 13 47	302695.6380	0.0369	0.035	0.0370	0.50	Melli+22
359:	62 2 61	61 2 60	305092.5580	-0.0020	0.035			Melli+22
360:	62 1 61	61 1 60	305232.1580	-0.0015	0.035			Melli+22
361:	60 3 57	59 3 56	305651.4080	-0.0323	0.035			Melli+22
362:	60 4 56	59 4 55	305843.3280	-0.0175	0.035			Melli+22
363:	61 8 53	60 8 52	307141.2540	-0.1601	0.035	0.0298	0.50	Melli+22
364:	61 8 54	60 8 53	307141.2540	0.2197	0.035	0.0298	0.50	Melli+22
365:	61 9 52	60 9 51	307151.7550	-0.0272	0.035	-0.0206	0.50	Melli+22
366:	61 9 53	60 9 52	307151.7550	-0.0140	0.035	-0.0206	0.50	Melli+22
367:	61 10 51	60 10 50	307228.8100	0.0745	0.035	0.0748	0.50	Melli+22
368:	61 10 52	60 10 51	307228.8100	0.0749	0.035	0.0748	0.50	Melli+22
369:	61 11 50	60 11 49	307354.5340	0.0206	0.035	0.0207	0.50	Melli+22
370:	61 11 51	60 11 50	307354.5340	0.0206	0.035	0.0207	0.50	Melli+22
371:	61 6 55	60 6 54	307551.5700	-0.0130	0.035			Melli+22
372:	61 5 57	60 5 56	307566.3690	-0.0547	0.035			Melli+22
373:	62 8 54	61 8 53	312165.3740	-0.3135	0.035	-0.0739	0.50	Melli+22
374:	62 8 55	61 8 54	312165.3740	0.1657	0.035	-0.0739	0.50	Melli+22
375:	62 9 53	61 9 52	312170.9110	0.0356	0.035	0.0442	0.50	Melli+22
376:	62 9 54	61 9 53	312170.9110	0.0528	0.035	0.0442	0.50	Melli+22
377:	62 10 52	61 10 51	312245.4130	0.0139	0.035	0.0142	0.50	Melli+22
378:	62 10 53	61 10 52	312245.4130	0.0144	0.035	0.0142	0.50	Melli+22
379:	62 7 56	61 7 55	312259.1730	0.0310	0.035			Melli+22
380:	62 7 55	61 7 54	312269.0490	0.0073	0.035			Melli+22
381:	62 11 51	61 11 50	312370.5450	0.0114	0.035	0.0115	0.50	Melli+22
382:	62 11 52	61 11 51	312370.5450	0.0114	0.035	0.0115	0.50	Melli+22
383:	62 6 57	61 6 56	312473.2430	-0.0252	0.035			Melli+22
384:	62 12 50	61 12 49	312535.4430	-0.0045	0.035	-0.0045	0.50	Melli+22
385:	62 12 51	61 12 50	312535.4430	-0.0045	0.035	-0.0045	0.50	Melli+22
386:	62 6 56	61 6 55	312615.3740	0.0316	0.035			Melli+22
387:	64 2 63	63 2 62	314692.8670	-0.0249	0.035			Melli+22
388:	64 1 63	63 1 62	314801.3740	-0.0140	0.035			Melli+22
